# Global trends and risk factors of aortic aneurysm mortality from 1990 to 2021: An analysis of the Global Burden of Disease Study 2021

**DOI:** 10.1097/ah9.0000000000000008

**Published:** 2025-06-30

**Authors:** Raluca-Maria Câsu, Loic Metz, David Freiholtz, Xiaofeng Zheng, Hanna M. Björck, Xiaowei Zheng

**Affiliations:** a Department of Molecular Medicine and Surgery, Karolinska Institutet, Stockholm, Sweden; b Division of Thoracic and Endocrine Surgery, Department of Surgery, University Hospital of Geneva, Geneva, Switzerland; c Department of Cell Physiology and Metabolism, Faculty of Medicine, University of Geneva, Geneva, Switzerland; d Division of Cardiology, Center for Molecular Medicine, Karolinska University Hospital, Solna, Sweden; e Department of Medicine, Karolinska Institutet, Stockholm, Sweden; f Department of Endocrinology and Metabolism, Research Center for Islet Transplantation, West China Hospital, Sichuan University, Chengdu, Sichuan Province, China

**Keywords:** Aortic aneurysm, Death rates, Epidemiology, Global burden of disease, Mortality, Risk factors

## Abstract

**Objectives:**

Aortic aneurysm (AA) is a life-threatening disease with significant global burden. However, population-based analyses of trends in AA mortality and its risk factors across sexes and world regions over time, based on up-to-date data, remain limited. This study aimed to conduct a comprehensive analysis of temporal and geographical trends in AA mortality and its risk factors over the past 3 decades, integrating age, sex, healthcare system, and socioeconomic factors using the latest data from the Global Burden of Disease (GBD) Study 2021.

**Methods:**

Using the GBD Study 2021 data, we analyzed AA-related deaths, death rates, and the age-standardized death rates (ASDRs) per 100,000, along with risk factors. Trends from 1990 to 2021 were compared across global regions and countries by sociodemographic index, health systems, and income. We also examined the impact of age, sex, and risk factors over time.

**Results:**

In 2021, global AA-related deaths reached 153,927, a 74.2% increase from 1990. However, ASDR declined from 2.54 to 1.86 deaths per 100,000 people. AA mortality increased with age and varied across global regions, influenced by socioeconomic factors. ASDR declined by 24.8% in Europe and 47.4% in America, while Asia saw a 38.6% increase. Higher mortality persisted in regions with high income, advanced health system, and high sociodemographic index. In 2021, Japan had the highest total AA-related deaths (23,815), and Armenia had the highest ASDR (9.16 per 100,000). AA-related mortality demonstrated notable sex disparities. Men had nearly twice the ASDR of women, but the gap narrowed over time. The sex disparity also varied by age and region. Risk factors were also differed by sex and region. Smoking was the primary risk factor for men, while high systolic blood pressure was more significant for women. High body-mass index was one of the emerging risk factors. Notably, the relative contribution of these risk factors has shifted over time, reflecting changes in lifestyle, public health policies, and healthcare access.

**Conclusion:**

AA-related mortality remains a global burden with regional and sex disparities, and is affected by socioeconomic factors. Smoking, hypertension, and obesity are key contributors, emphasizing the need for targeted prevention, screening, and healthcare access.

## 1. Introduction

Aortic aneurysm (AA) is a life-threatening vascular disease defined as the focal and permanent enlargement of the aorta, exceeding 50% of its normal diameter^[1,2]^. AA generally progresses asymptomatically and is often discovered incidentally during routine physical examinations or on imaging^[1]^, except for the United Kingdom (UK), United States of America (USA), and Sweden where screening programs for men above 65 have been implemented^[3–5]^. Based on the location of the aneurysm, they are classified either as thoracic aortic aneurysm (TAA) or abdominal aortic aneurysm (AAA), with the latter being the most prevalent^[6]^. The prevalence of AAA ranges from 0.4% to 7.6% depending on the population^[7]^, while the global prevalence of TAA is 0.16%^[8]^.

The main risk factors for AAA include advanced age, smoking, male sex, family history of AAA, atherosclerosis, and hypertension^[9]^. TAA shares some (e.g., age, smoking, and to some extent hypertension) but not all risk factors with AAA, and is in ~20% of cases associated with genetic syndromes affecting the connective tissue, such as Marfan syndrome and Ehlers-Danlos syndrome, or familial where sporadic mutations segregate in families^[1,10]^. The vast majority of TAAs, however, are sporadic or occur in association with a bicuspid aortic valve, denoted bicuspid aortic valve-aortopathy^[11]^. Rupture of an AA is associated with high mortality^[9]^. The primary approach for AA prevention has focused on smoking cessation and hypertension control. Surgical intervention is indicated for large aneurysms or those that are at high risk of rupture. As for now, no pharmacological therapies have been approved for the treatment of AA^[1]^.

With the restriction of risk factors such as smoking and better blood pressure control, along with technological and medical advances in the treatment of AA, a decline in AA mortality over time would be expected. However, previous epidemiological studies primarily relied on national datasets and focused on specific populations or inhospital mortality. Therefore, comprehensive population-based analysis of AA mortality and population-level risk factors is warranted to better understand the mechanisms underlying the increased AA mortality^[12]^.

The Global Burden of Diseases, Injuries, and Risk Factors Study (GBD) is a comprehensive research initiative that quantifies the impact of diseases, injuries, and risk factors on global health in more than 200 countries and at the subnational level in more than 20 countries^[13,14]^. It was initiated more than 30 years ago and has been conducted by the Institute for Health Metrics and Evaluation (IHME). It provides dynamic data since 1990 for various diseases, injuries, and risk factors, broken down by region, age, and sex, and tracks changes over time. Its standardized metrics, such as age-standardized death rates (ASDRs), allow for accurate comparisons across populations and time periods while minimizing biases from demographic changes. The goal is to help inform public health policies and priorities by highlighting major health challenges and guiding resource allocation for interventions^[13,14]^.

Recent studies using GBD 2019 data have explored the global burden of AA and its attributable factors, or focused on specific countries such as Iran and China to provide localized perspective^[15–18]^. However, updated data are needed to better understand the regional and sex-specific variations, especially in light of emerging evidence of poorer outcomes for women^[19]^. Therefore, this study aimed to conduct a comprehensive analysis of temporal and geographical trends in AA mortality and its risk factors over the past 3 decades, integrating age, sex, healthcare system, and socioeconomic factors using the latest data from GBD 2021.

## 2. Methods

### 2.1. Data source and extraction

The data for this analysis were sourced from GBD 2021 through the online query tool https://vizhub.healthdata.org/gbd-results/. The GBD study was conducted by the Institute for IHME at the University of Washington. The GBD 2021 provides a robust and comprehensive framework for understanding the burden of 288 causes of death and the contribution of 88 risk factors for both men and women worldwide, covering 204 countries and 811 subnational locations, for each year from 1990 until 2021^[13,14]^.

The GBD 2021 provides data on burden of AA, corresponding to one of the International Classification of Disease-10 codes I71 to I71.9, combining both AAA and TAA at global, regional, national, and subnational levels. We have extracted data of absolute death number, death rate per 100,000 persons, ASDR per 100,000, and percentage changes from 1990 to 2021. ASDR is a measure that adjusts for differences in age distribution within a population. Older populations naturally have higher death rates due to aging-related health issues. By standardizing death rates across age groups, ASDR allows for fair comparisons between different countries or time periods, ensuring that differences in mortality are not simply due to variations in age structure.

The formula for ASDR per 100,000 is as follows:


$$\begin{array}{l} ASDR=\left(\frac{\Sigma_{i}\left(D_{i}\times S_{i}\right)}{\Sigma_{i}P_{i}}\right)\times 100000 \end{array}$$

Where *D*_i_ = number of deaths in age group *i*, *S*_i_ = standard population for age group *i* (from a reference population), *P*_*i*_ = total population in age group *i*, 100,000 = scaling factor to express the rate per 100,000 people.

We did not evaluate the data of percentage of total deaths, trying to avoid the influence of deaths from COVID-19 pandemic since late 2019. Disability-adjusted life years (DALYs) combine the impact of both premature death and disability caused by the disease^[13]^. However, since a number of AA cases are undiagnosed until aneurysm rupture or dissections, DALYs may be influenced a lot by screening policy and health care access. Therefore, we did not evaluate DALY data but rather focused on ASDR for the comparison of different populations and over time.

### 2.2. Risk factors

To evaluate the contribution of risk factors to AA-related death, we extracted the percentage contribution of risk factors for AA-related mortality, and also the ASDR attributable to individual risk factors. GBD 2021 study utilized the comparative risk assessment framework to quantify the burden attributable to individual risk factors^[20,21]^. Briefly, this approach involves a hierarchical organization of 88 risk factors and quantifies their contributions to specific health outcomes. Relative risks for each risk-outcome pair were derived from systematic reviews and meta-analyses. Estimates were standardized for age, sex, and geographical location, ensuring comparability across regions and over time. The GBD framework thus assigns attributable fractions to outcomes, based on the prevalence of exposure and relative risk in literature. In total, 7 risk factors were associated with AA mortality in the GBD 2021 study, and relative risks employed in the present study were not adjusted for mediation to capture the direct effect of a risk factor on the outcome. Each association is considered separately; therefore, the combined height of multiple bar segments of the graphs may not be 100%.

### 2.3. Studied regions, countries, and populations

Following analysis of global changes in AA-related mortality and its risk factors, we have evaluated data from 4 regions that GBD Study 2021 provides (Europe, America, Asia, and Africa). We also assessed data from regions defined according to the health system levels: advanced, basic, limited, and minimal health system regions; regions defined by income including commonwealth high-, middle-, and low-income regions; and regions with different sociodemographic index (SDI). The SDI categorizes populations based on income, education, and fertility rates^[22]^.

We found that Europe and Asia had opposite trends of AA mortality changes over the years, so we have then evaluated the ASDR for AA in different regions and countries in Europe and Asia. The inclusion of countries in different parts of Europe and Asia was based on the classification in the GBD study. The data were further stratified by sex and age groups.

To ensure data reliability, we set an inclusion criterion for intercountry comparison, based on country population size, using the same cutoff as applied in a previous World Health Organization study^[23]^. Consequently, countries with populations less than 90,000, including Monaco, Nauru, Andorra, and San Marino, were excluded from comparison.

### 2.4. Data analysis

The differences in global AA-caused death number, death rate, ASDR per 100,000 between men and women were computed as mean of men − mean of women. The lower and upper bounds of the 95% uncertainty intervals (UI) of each sex were back transformed to their corresponding standard error (SE) to estimate mathematically correct UIs as follows: ΔASDR/Death rate = ASDRmen − ASDRwomen; SE_lower_ = $\sqrt{SElowerMEN{}^{2}\;+\;SElowerWOMEN{}^{2}}$; SE_upper_ = $\sqrt{SEupperMEN{}^{2}\;+\;SEupperWOMEN{}^{2}}$; UI_upper_ = Δmean + 1.96*SE_upper_; UI_lower_ = Δmean − 1.96*SE_lower_.

A linear regression model was used to assess the statistical significance of temporal trends of AA mortality across the analyzed regions and countries. To identify differential evolution in temporal trends between sexes, we tested for an interaction in the linear model between sex and time using 2-way analysis of variance. A *P*-value of less than 0.05 was considered statistically significant. As GBD data provide only summary statistics without raw individual-level data or sample size, we only did descriptive statistics for the rest of the study.

### 2.5. Data visualization

Data were imported into R version 4.4.2 (https://cran.r-project.org/) for visualization through graphs and descriptive analysis, providing a clear depiction of patterns and trends in the population of interest. Initial graphs were generated using ggplot2 package^[24]^ (https://ggplot2.tidyverse.org/) or GraphPad Prism version 10 (Dotmatics, Boston, MA, USA). All panels were assembled together using Adobe Illustrator 2025.

Data are presented as mean with 95% UI, which represent the range between the upper and lower bounds of the aggregated data from the GBD study. All data together with their 95% UI upper and lower bounds are presented in Supplemental Digital Content, https://links.lww.com/AHJ/A4.

## 3. Results

### 3.1. Global mortality and age-standardized death rate of aortic aneurysm

**Figure 1.**
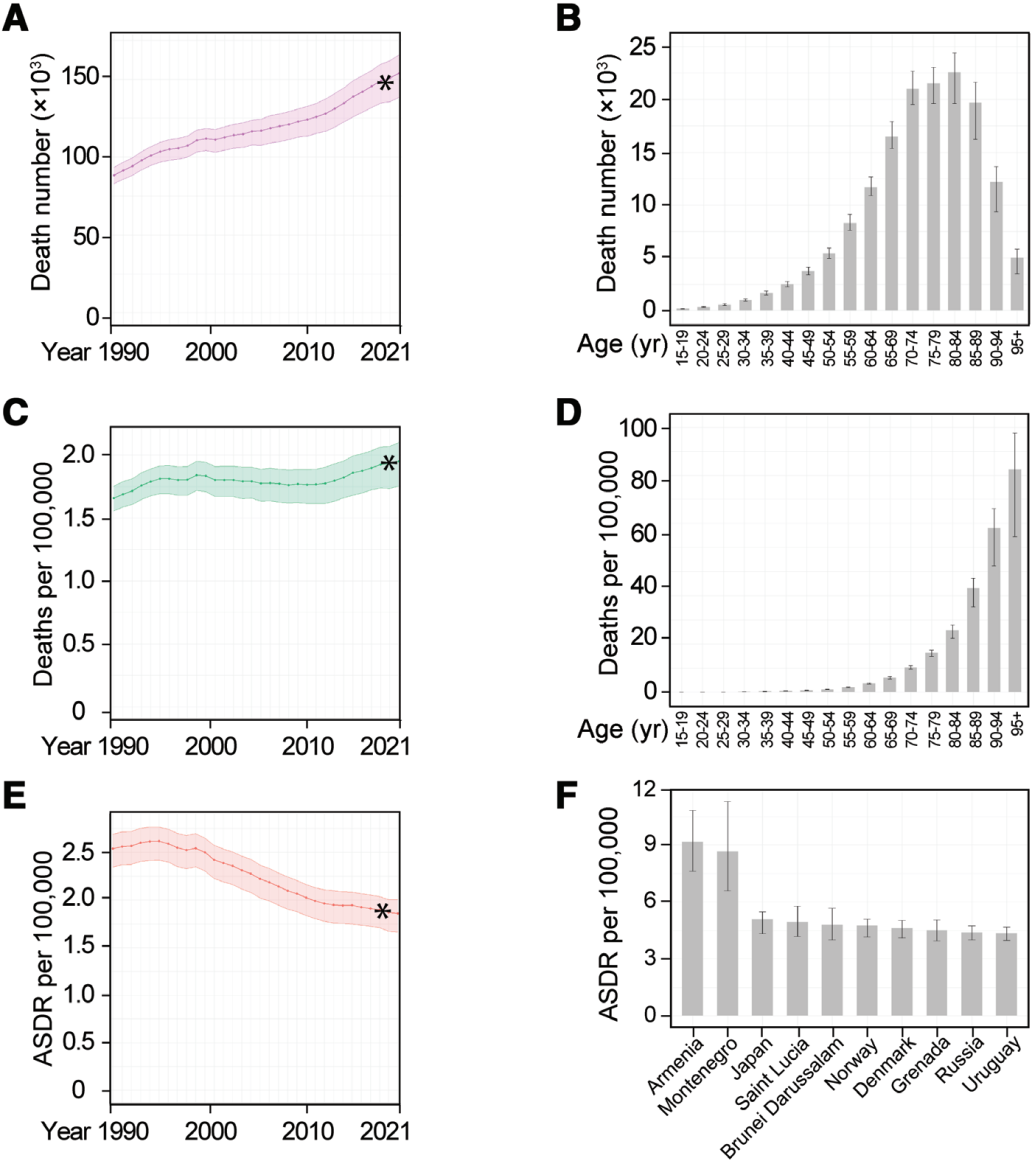
Global trends in the mortality of AA. (A) Global number of AA deaths in all ages from 1990 to 2021. (B) AA deaths per age group in 2021. (C) Global AA death rate per 100,000 in all ages from 1990 to 2021. (D) Global AA death rates per 100,000 per age group in 2021. (E) Global AA-caused ASDR per 100,000 from 1990 to 2021. (F) AA-caused ASDR per 100,000 in top 10 countries. Data are shown as means with 95% UIs. Linear trends were tested by linear regression analysis. *P < 0.001. AA, aortic aneurysm; ASDR, age-standardized death rate; UIs, uncertainty intervals.

In 2021, the global number of AA-related deaths was 153,927 (95% UI: 138,413–165,739), with an average increase of 74.2% from 88,353 (95% UI: 83,090–93,492) in 1990 (*P* < 0.001; Fig. 1A and Table 1). Japan, India, the USA, Russia, and Brazil reported the highest number of AA-related deaths in 2021 (Table 2). AA-related death number increased with age, peaking at the age of 70 to 84 years old (Fig. 1B). However, these data are affected by population numbers in each country and in each age group.

**Table 1:** Death number, death rates and age-standardized death rates in world regions in 1990 and 2021 along with the percent changes.

Location	Death number 1990	Death number 2021	% change in death number 1990–2021	Death rate per 100,000 1990	Death rate per 100,000 2021	% change in death rate per 100,000 1990 - 2021	ASDR per 100,000 1990	ASDR per 100,000 2021	% change in ASDR 1990, 2021
Global	88353 (83090, 93492)	153927 (138413, 165739)	74.2*** (63.0, 83.6)	1.66 (1.56, 1.75)	1.95 (1.75, 2.10)	17.8*** (10.2, 24.1)	2.54 (2.35, 2.69)	1.87 (1.67, 2.00)	−26.7*** (−31.1, −23.1)
Four world regions									
Europe	40891 (38817, 41959)	50082 (45990, 53067)	22.5*** (16.9, 28.3)	5.10 (4.84, 5.23)	5.90 (5.42, 6.25)	15.8 (10.5, 21.3)	3.95 (3.74, 4.06)	2.97 (2.76, 3.15)	−24.8*** (−27.9, −21.2)
America	26779 (25150, 27685)	31596 (28699, 33296)	18.0 (13.2, 22.3)	3.74 (3.51, 3.87)	3.08 (2.79, 3.24)	−17.7*** (−21.1, −14.7)	4.43 (4.14, 4.59)	2.33 (2.12, 2.45)	−47.4*** (−49.3, −45.6)
Africa	4214 (2648, 6608)	9125 (5666, 13820)	116.6*** (78.1, 158.1)	0.67 (0.42, 1.05)	0.66 (0.41, 1.00)	−1.2*** (−18.8, 17.7)	1.82 (1.14, 2.82)	1.65 (1.04, 2.47)	−9.1*** (−24.2, 6.3)
Asia	16240 (13949, 19497)	62840 (54678, 71295)	287.0*** (228.1, 349.3)	0.51 (0.44, 0.61)	1.36 (1.18, 1.54)	165.8*** (125.3, 208.6)	1.01 (0.87, 1.18)	1.40 (1.20, 1.59)	39.5*** (20.4, 59.6)
Health system grouping									
Advanced	68082 (64194, 70005)	92496 (81937, 98972)	35.9*** (26.9, 42.5)	5.16 (4.87, 5.31)	6.05 (5.36, 6.47)	17.1** (9.4, 22.9)	4.15 (3.90, 4.28)	2.98 (2.68, 3.16)	−28.1*** (−31.3, −25.1)
Basic	12800 (11864, 13937)	36566 (33367, 40060)	185.7*** (146.7, 224.8)	0.55 (0.51, 0.60)	1.16 (1.06, 1.27)	109.4*** (80.8, 138.1)	1.02 (0.94, 1.11)	1.05 (0.95, 1.15)	2.7 (−10.3, 15.1)
Minimal	857 (471, 1580)	1745 (856, 3183)	103.6*** (60.7, 149.1)	0.63 (0.35, 1.17)	0.52 (0.25, 0.95)	−18.1*** (−35.4, 0.1)	1.71 (0.93, 3.14)	1.70 (0.84, 3.09)	−0.9* (−21.0, 20.2)
Limited	6481 (4793, 9600)	22930 (18425, 31894)	253.8*** (178.0, 345.1)	0.41 (0.31, 0.61)	0.80 (0.64, 1.11)	93.0*** (51.6, 142.8)	0.99 (0.73, 1.45)	1.32 (1.06, 1.83)	33.6*** (6.4, 66.0)
Commonwealth									
High income	13799 (13065, 14230)	9778 (8609, 10352)	−29.1*** (−33.7, −26.5)	12.41 (11.75, 12.80)	6.67 (5.87, 7.06)	−46.2*** (−49.6, −44.2)	8.66 (8.18, 8.94)	3.22 (2.86, 3.40)	−62.8*** (−64.8, −61.6)
Middle income	5365 (4150, 7408)	19117 (15474, 25646)	256.3*** (190.5, 332.8)	0.46 (0.35, 0.63)	0.91 (0.74, 1.22)	98.9*** (62.1, 141.6)	1.06 (0.83, 1.45)	1.39 (1.13, 1.85)	30.6*** (8.1, 56.9)
Low income	975 (615, 1603)	3075 (1941, 4829)	215.4*** (131.2, 332.9)	0.46 (0.29, 0.76)	0.79 (0.50, 1.24)	70.1*** (24.6, 133.5)	1.27 (0.80, 2.11)	1.53 (0.98, 2.38)	19.9*** (−10.8, 63.5)
SDI									
High	53929 (50582, 55553)	67202 (57735, 72287)	24.6*** (14.2, 31.1)	6.13 (5.75, 6.32)	6.14 (5.28, 6.61)	0.1*** (−8.1, 5.4)	4.76 (4.46, 4.91)	2.87 (2.51, 3.06)	−39.8*** (−43.5, −37.2)
High-middle	18321 (17508, 19197)	34827 (32309, 37274)	90.1*** (74.2, 104.6)	1.72 (1.65, 1.80)	2.67 (2.48, 2.86)	55.0*** (42.0, 66.8)	1.99 (1.88, 2.08)	1.79 (1.66, 1.92)	−10.1*** (−17.3, −3.6)
Middle	8804 (8110, 9844)	28528 (25797, 30959)	224.0*** (182.5, 262.6)	0.51 (0.47, 0.57)	1.17 (1.05, 1.26)	128*** (98.7, 155.1)	1.03 (0.94, 1.14)	1.15 (1.04, 1.25)	12.5* (−1.4, 25.3)
Low-middle	4608 (3664, 6272)	16808 (13956, 22468)	264.7*** (199.5, 331.6)	0.40 (0.32, 0.54)	0.87 (0.73, 1.17)	120.5*** (81.0, 160.8)	0.89 (0.71, 1.20)	1.31 (1.09, 1.76)	47.9*** (22.9, 73.0)
Low	2557 (1568, 4437)	6371 (3932, 10434)	149.2*** (101.5, 207.7)	0.51 (0.31, 0.89)	0.57 (0.35, 0.93)	11.7 (−9.6, 38.0)	1.37 (0.83, 2.37)	1.48 (0.91, 2.44)	8.4 (−12.0, 31.9)

Data are presented as mean with their 95% UIs with upper and lower bounds. Linear trends were tested by linear regression analysis.

ASDR, age-standardized death rates; SDI, sociodemographic index; UI, uncertainty interval.

* *P* < 0.05.

** *P* < 0.01.

*** *P* < 0.001.

**Table 2:** Death number, death rates and age-standardized death rates in 1990 and 2021 in countries with highest burden of aortic aneurysm along with the percent changes.

Location	Death number 1990	Death number 2021	% change in death number 1990, 2021	Death rate per 100,000 1990	Death rate per 100,000 2021	% change in death rate per 100.000 1990, 2021	ASDR per 100,000 1990	ASDR per 100,000 2021	% change in ASDR 1990, 2021
Armenia	117 (96, 142)	397 (330, 467)	238.3*** (155.2, 351.6)	3.43 (2.81, 4.16)	13.24 (11.02, 15.60)	286.4*** (191.4, 415.7)	4.55 (3.71, 5.57)	9.16 (7.61, 10.81)	101.4*** (51.4, 170.2)
Montenegro	40 (32, 52)	81 (61, 105)	100.1*** (44.4, 189.4)	6.45 (5.16, 8.31)	13.09 (9.91, 17.03)	102.8*** (46.3, 193.2)	6.70 (5.35, 8.64)	8.65 (6.59, 11.28)	29.2*** (−7.8, 89.2)
Japan	4797 (4448, 4982)	23815 (19180, 26463)	396.4*** (327.8. 435.2)	3.81 (3.53, 3.96)	18.65 (15.02, 20.72)	389.2*** (321.6, 427.4)	2.92 (2.68, 3.04)	5.07 (4.33, 5.47)	73.6*** (60.4, 81.6)
Saint Lucia	8 (7, 9)	11 (10, 13)	45.1** (19.3, 73.9)	5.75 (5.24, 6.42)	6.42 (5.40, 7.49)	11.6 (−8.1, 33.8)	10.31 (9.41, 11.56)	4.95 (4.17, 5.77)	−52.0*** (−60.5, −42.7)
Brunei Darussalam	5 (4, 6)	13 (11, 15)	162.6*** (98.4, 263.1)	2.86 (2.41, 3.39)	1.89 (1.46, 2.36)	50.9*** (14.0, 108.7)	5.61 (4.47, 6.89)	4.79 (4.00, 5.67)	−14.7 (−35.6, 15.0)
Norway	573 (539, 598)	541 (466, 581)	−5.5*** (−12.6, −0.1)	9.98 (8.61, 10.72)	13.49 (12.68, 14.08)	−26.0*** (−31.5, −21.7)	7.58 (7.16, 7.90)	4.75 (4.15, 5.08)	−37.4*** (−41.8, −34.0)
Denmark	617 (547, 673)	575 (530, 616)	7.2* (−3.8, 19.1)	11.18 (10.30, 11.98)	10.54 (9.35, 11.49)	−5.7*** (−15.5, 4.7)	6.71 (6.21, 7.17)	4.61 (4.10, 5.02)	−31.2*** (−38.2, −23.9)
Grenada	4 (3, 4)	5 (4, 5)	26.0 (0.7, 51.8)	4.30 (3.75, 5.17)	4.59 (4.01, 5.16)	6.8** (−14.6, 28.7)	4.77 (4.15, 5.70)	4.51 (3.94, 5.04)	−5.4* (−24.0, 13.4)
Russia	4699 (4559, 4800)	10445 (9555, 11307)	122.3*** (104.4, 139.8)	7.21 (6.60, 7.81)	3.11 (3.02, 3.18)	131.6*** (113, 149.9)	2.72 (2.62, 2.78)	4.38 (4.01, 4.74)	61.3*** (48.2, 73.9)
Uruguay	228 (213, 247)	256 (233, 276)	12 (0, 23.5)	7.28 (6.79, 7.86)	7.52 (6.85, 8.09)	3.3* (−7.8, 13.9)	5.81 (5.42, 6.24)	4.35 (3.98, 4.67)	−25.2*** (−33.3, −17.5)
Brazil	2857 (2739, 2952)	10010 (9186, 10566)	250.4*** (229.5, 269.1)	1.92 (1.84, 1.99)	4.54 (4.17, 4.80)	136.1*** (122.0, 148.7)	3.39 (3.23, 3.52)	4.06 (3.72, 4.29)	19.7* (13.3, 26.02)
Malaysia	971 (812, 1159)	268 (217, 323)	262.8*** (167.4, 386.3)	3.05 (2.55, 3.64)	1.52 (1.23, 1.83)	101.5*** (48.5, 170.1)	3.30 (2.66, 4.00)	4.04 (3.38, 4.88)	22.4 (−9.2, 65.7)
Greece	574 (536, 611)	1066 (955, 1149)	85.6*** (68.9, 102.9)	5.53 (5.16, 5.88)	10.47 (9.39, 11.29)	89.6*** (72.4, 107.2)	3.81 (3.55, 4.03)	3.99 (3.65, 4.26)	4.7* (−3.7, 13.4)
Georgia	37 (31, 44)	173 (146, 200)	369.4*** (257.2, 500.7)	0.67 (0.56, 0.79)	4.80 (4.06, 5.56)	618.6*** (446.8, 819.7)	0.61 (0.51, 0.72)	2.88 (2.43, 3.35)	374.2*** (260.2, 510)
Kazakhstan	180 (147, 231)	377 (302, 462)	109.4*** (48.8, 187.9)	1.10 (0.90, 1.41)	1.99 (1.59, 2.44)	81.1*** (28.7, 149)	1.46 (1.19, 1.87)	2.23 (1.80, 2.71)	52.8** (8.1, 112.2)
USA	17399 (16107, 18106)	12195 (10940, 12944)	−29.9*** (−32.6, −27.6)	6.85 (6.34, 7.13)	3.67 (3.29, 3.89)	−46.4*** (−48.5, −44.6)	5.17 (4.80, 5.38)	2.06 (1.87, 2.18)	−60.1*** (−61.5, −59.0)
India	2652 (1578, 4344)	12805 (9107, 18763)	382.9*** (258.3, 576.7)	0.31 (0.18, 0.51)	0.91 (0.64, 1.33)	191.2*** (116.0, 308.1)	0.68 (0.40, 1.08)	1.20 (0.86, 1.74)	77.4*** (30.0, 149.5)

Data are presented as mean with their 95% UIs with upper and lower bounds. Linear trends were tested by linear regression analysis.

ASDR, age-standardized death rate; UIs, uncertainty intervals.

* *P* < 0.05.

** *P* < 0.01.

*** *P* < 0.001.

We, therefore, explored the global death rate of AA per 100,000 population. It increased from an average of 1.66 (95% UI: 1.56–1.75) deaths in 1990 to 1.95 (95% UI: 1.75–2.10) deaths in 2021 in all ages per 100,000 population (17.8% increase, *P* < 0.001; Fig. 1C and Table 1). Japan, Armenia, Montenegro, Denmark, and Greece ranked high in the death rate of AA in 2021 (Table 2). The global death rate also increased with age, peaking at the highest age (Fig. 1D), indicating that the death rate can be affected by the age of the population.

To remove the confounding effects caused by age, we explored data of ASDR. As shown in Figure 1E and Table 1, the global ASDR decreased from an average of 2.54 (95% UI: 2.35–2.69) deaths in 1990 to 1.87 (95% UI: 1.67–2.00) deaths in 2021 per 100,000 population (26.7% decrease, *P* < 0.001). Armenia, Montenegro, Japan, Saint Lucia, Brunei Darussalam, Norway, Denmark, Grenada, Russia, and Uruguay are the top 10 countries with highest burden of AA-caused death after age standardization in 2021 (Fig. 1F and Table 2).

These data indicate that the global burden of AA-related mortality has been increasing over the time from 1990 to 2021. However, the increase in global population and life span have contributed to the increased mortality of AA. After age standardization, the global ASDR of AA decreased. We, therefore, decided to use ASDR to evaluate the difference in AA-related mortality among different populations in the world across time and sex.

### 3.2. Mortality of aortic aneurysm in world regions

**Figure 2.**
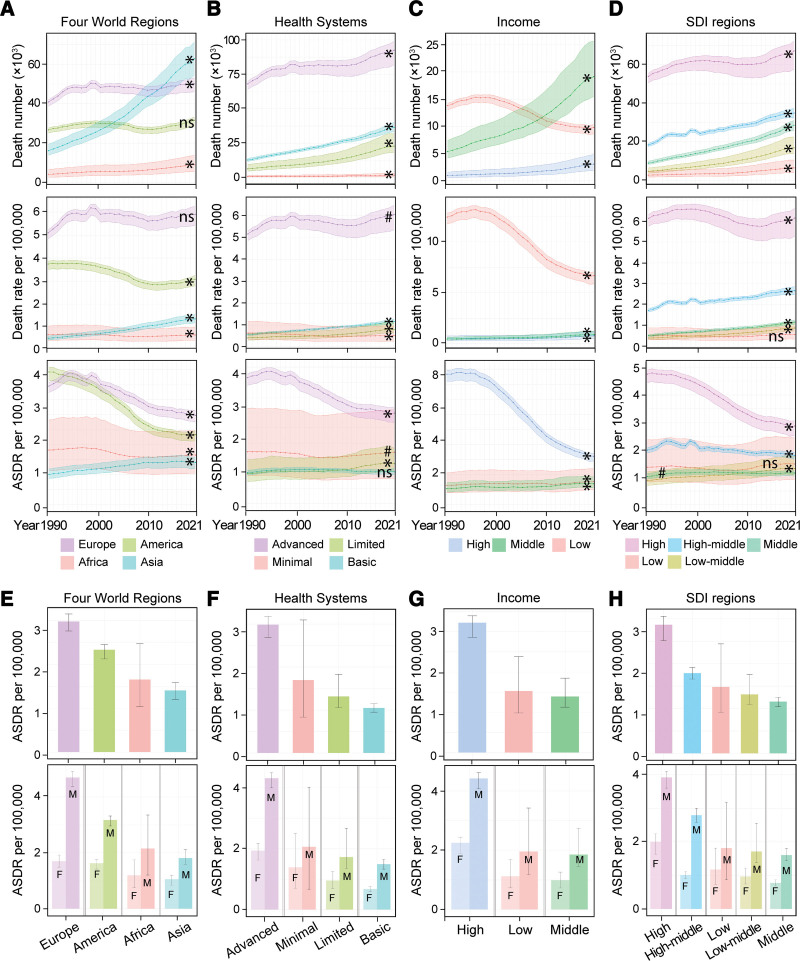
Mortality of aortic aneurysm among world regions. (A–D) The death number, death rate, and ASDR per 100,000 of AA analyzed by Four World Regions, Health System Grouping, Commonwealth (Income), and SDI regions from 1990 to 2021. (E–H) ASDR of AA per 100,000 in 2021 analyzed by Four World Regions, Health System Grouping, Commonwealth (Income), and SDI and their subgroups. Data for both sexes are shown in the upper panel, and stratified data by sex are shown in the lower panel. Data are shown as mean with 95% UI. Linear trends were tested by linear regression analysis. *P < 0.001; #*P* < 0.05; ns: not significant. *P* values are also shown in Table 2. AA, aortic aneurysm; ASDR, age-standardized death rate; F, women; M, Men; SDI, sociodemographic index.

We next analyzed AA mortality among different regions in the world. From 1990 to 2021, AA mortality increased dramatically in Asia, with a 287% (95% UI: 228.1%–349.3%) increase in death number (*P* < 0.001), a 165.8% (95% UI: 125.3%–208.6%) rise in the death rate per 100,000 (*P* < 0.001), and a 39.5% (95% UI: 20.4%–59.6%) increase in ASDR (*P* < 0.001). AA-caused death number also increased in Europe by 22.5% (95% UI: 16.9%–28.3%, *P* < 0.001), whereas in America, the increase was 18% (95% UI: 13.2%–22.3%) but not statistically significant (*P* = 0.413). However, after adjusting for population growth and age distribution, ASDR decreased 24.8% in Europe and 47.4% in America (*P* < 0.001). In Africa, both the death rate and ASDR showed relatively small change, but the number of deaths increased by 116.6% (95% UI: 78.1%–158.1%, *P* < 0.001; Fig. 2A and Table 1).

Although it decreased, Europe still had the highest ASDR caused by AA (2.97 deaths per 100,000 [95% UI: 2.76–3.15]) in 2021, which was followed by America (2.33 deaths per 100,000 [95% UI: 2.12–2.45]). Despite a continuous increase, the ASDR of AA remained at lower levels in Asia (1.4 deaths per 100,000 [95% UI: 1.20–1.59]), similar as in Africa (1.65 deaths per 100,000 [95% UI: 1.04–2.47]). However, due to the large population, Asia reported the highest number of AA-related deaths in 2021, followed by Europe and America (Fig. 2A, 2E and Table 1).

Interestingly, ASDR of AA declined from 1990 to 2021 in world regions, which had advanced health systems (Fig. 2B), high income (Fig. 2C), and high SDI (*P* < 0.001) (Fig. 2D). However, these regions still had the highest AA-caused ASDR in 2021 (Fig. 2B–2D). The other regions, with basic to limited health systems, middle and low income, and middle to low SDI, had constantly lower AA-caused ASDR but it increased over time (*P* < 0.001; Fig. 2 and Table 1).

Since ASDR of AA decreased in Europe but increased in Asia, we will explore European and Asian countries in detail to understand the burden of AA and its underlying reasons.

### 3.3. Mortality of aortic aneurysm in Europe

To understand further ASDR of AA in Europe, we analyzed 3 GBD European regions, Western, Central, and Eastern Europe. The countries with the highest observed ASDRs that belong to each of the European regions are listed in Table 3.

**Table 3:** Death number, death rates and age-standardized death rates in studied European countries in 1990 and 2021 along with the percent changes.

Location	Death number 1990	Death number 2021	% change in death number 1990, 2021	Death rate per 100,000 1990	Death rate per 100,000 2021	% change in death rate per 100,000 1990, 2021	ASDR per 100,000 2019	ASDR per 100,000 2021	% change in ASDR 1990, 2021
Western Europe	29016 (27281, 29890)	27511 (24098, 29189)	−5.2*** (−11.9, −1.1)	7.55 (7.10, 7.78)	6.29 (5.51, 6.67)	−16,7*** (−22,6, −13,1)	4.78 (4.50, 4.92)	2.57 (2.30, 2.71)	−46.2*** (−48.9, −44.2)
Norway	573 (539, 598)	541 (466, 581)	−5.6*** (−12.7, −0.1)	13.49 (12.68, 14.08)	9.98 (8.61, 10.72)	−26*** (−31.6, −21.7)	7.58 (7.16, 7.90)	4.75 (4.15, 5.08)	−37.4*** (−41.8, −34.0)
Denmark	575 (530, 616)	617 (547, 673)	7.3* (−3.9, 19.1)	11.18 (10.30, 11.98)	10.54 (9.35, 11.49)	−5.7*** (−15.5, 4.7)	6.71 (6.21, 7.17)	4.61 (4.10, 5.02)	−31.2*** (−38.2, −23.9)
United Kingdom	9552 (9013, 9824)	6071 (5374, 6435)	−36.4*** (−40.7, −34.2)	16.67 (15.73, 17.15)	8.95 (7.92, 9.48)	−46.3*** (−49.9, −44.4)	9.72 (9.16, 10.00)	4.03 (3.61, 4.25)	−58.6*** (−60.9, −57.3)
Sweden	1226 (1141, 1295)	985 (846, 1098)	−19.7*** (−28, −11.4)	14.28 (13.29, 15.08)	9.49 (8.15, 10.58)	−33.5*** (−40.4, −26.6)	7.41 (6.91, 7.79)	3.88 (3.38, 4.33)	−47.6*** (−52.8, −42.2)
Italy	2816 (2646, 2922)	3655 (3187, 3939)	29.8 (19.3, 37.6)	4.96 (4.66, 5.14)	6.11 (5.33, 6.59)	23.2 (13.3, 30.7)	3.10 (2.91, 3.21)	2.18 (1.95, 2.34)	−29.6*** (−33.6, −25.7)
Spain	1402 (1313, 1480)	2256 (1979, 2434)	60.9*** (47, 73.6)	3.61 (3.39, 3.82)	4.95 (4.34, 5.34)	37 (25.1, 47.8)	2.54 (2.37, 2.68)	2.08 (1.87, 2.23)	−18.3*** (−24.2, −12.4)
Portugal	238 (223, 253)	405 (361, 437)	70.4*** (54.4, 86.2)	2.35 (2.20, 2.49)	3.82 (3.41, 4.12)	62.8*** (47.6, 78)	1.75 (1.64, 1.86)	1.51 (1.37, 1.62)	−14.1*** (−21.1, −7.0)
Malta	11 (10, 12)	14 (12, 16)	28.6 (12.9, 46.1)	2.94 (2.73, 3.17)	3.17 (2.78, 3.55)	7.8* (−5.4, 22.4)	2.60 (2.41, 2.80)	1.32 (1.17, 1.47)	−49.4*** (−55.2, −43.0)
Central Europe	4379 (4217, 4522)	6682 (6141, 7318)	52.6*** (40.9, 67.4)	3.50 (3.37, 3.62)	5.80 (5.33, 6.35)	65.6*** (52.9, 81.6)	3.07 (2.95, 3.18)	2.93 (2.69, 3.21)	−4.8*** (−12.2, 4.6)
Montenegro	40 (32, 52)	81 (61, 105)	100.1*** (44.4, 189.4)	6.45 (5.16, 8.31)	13.09 (9.91, 17.03)	102.8*** (46.3, 193.2)	6.70 (5.35, 8.64)	8.65 (6.59, 11.28)	29.2*** (−7.8, 89.2)
Serbia	397 (330, 474)	692 (549, 872)	74.4*** (32.5, 130.8)	4.12 (3.43, 4.92)	7.75 (6.15, 9.77)	88.2*** (43.1, 149.1)	4.22 (3.50, 5.14)	4.05 (3.21, 5.10)	−4.2* (−28.5, 24.7)
Poland	1943 (1876, 1997)	2576 (2307, 2831)	32.6*** (20.6, 46)	5.09 (4.92, 5.23)	6.74 (6.03, 7.40)	32.4*** (20.4, 45.7)	4.54 (4.36, 4.66)	3.44 (3.09, 3.79)	−24.2*** (−31.1, −16.4)
Croatia	157 (137, 180)	317 (271, 365)	102.2*** (62.4, 148)	3.22 (2.82, 3.70)	7.53 (6.44, 8.67)	133.6*** (87.6, 186.4)	2.79 (2.45, 3.19)	3.34 (2.87, 3.86)	19.7*** (−3.4, 46.5)
Bosnia and Herzegovina	79 (58, 109)	189 (135, 252)	139.5*** (48.1, 286.6)	1.76 (1.29, 2.42)	5.73 (4.09, 7.64)	226.2*** (101.7, 426.6)	2.12 (1.55, 2.89)	3.00 (2.15, 3.99)	41.6*** (−12.1, 127.1)
Hungary	460 (426, 496)	574 (507, 647)	24.7*** (9, 41.2)	4.43 (4.10, 4.77)	5.98 (5.29, 6.74)	35.1*** (18, 52.9)	3.23 (3.00, 3.48)	2.87 (2.54, 3.23)	−11.2*** (−22.0, −0.1)
Czech Republic	405 (377, 436)	637 (554, 722)	57.3*** (35, 80.2)	3.94 (3.67, 4.24)	6.00 (5.21, 6.79)	52.3*** (30.7, 74.4)	2.96 (2.76, 3.18)	2.83 (2.46, 3.19)	−4.2 (−17.6, 10.1)
Eastern Europe	6812 (6583, 7085)	13406 (12354, 14430)	96.8*** (80.4, 112.4)	3.01 (2.91, 3.13)	6.48 (5.97, 6.98)	115.6*** (97.6, 132.7)	2.52 (2.43, 2.62)	3.82 (3.52, 4.12)	51.8*** (38.9, 64.0)
Russia	4699 (4559, 4800)	10445 (9555, 11307)	122.3*** (104.4, 139.8)	3.11 (3.02, 3.18)	7.21 (6.60, 7.81)	131.7*** (113, 149.9)	2.72 (2.62, 2.78)	4.38 (4.01, 4.74)	61.3*** (48.2, 74.0)
Belarus	338 (294, 411)	596 (486, 715)	76.2*** (37.7, 126.7)	3.24 (2.82, 3.93)	6.39 (5.21, 7.66)	97.3*** (54.3, 153.9)	2.67 (2.31, 3.23)	3.75 (3.05, 4.49)	40.5*** (10.1, 81.4)
Estonia	58 (52, 63)	98 (85, 110)	69.6*** (45, 100.7)	3.67 (3.34, 4.03)	7.45 (6.49, 8.42)	102.9*** (73.5, 140.1)	2.86 (2.60, 3.13)	3.34 (2.90, 3.78)	16.9 (0.01, 37.9)
Lithuania	97 (88, 106)	187 (162, 211)	93.2*** (67.1, 123.1)	2.63 (2.40, 2.89)	6.84 (5.95, 7.73)	160.2*** (125.1, 200.5)	2.16 (1.97, 2.36)	3.15 (2.73, 3.56)	46.0*** (26.2, 69.3)
Latvia	79 (72, 86)	125 (107, 142)	58.7*** (36.4, 84.4)	2.96 (2.72, 3.23)	6.68 (5.73, 7.57)	125.5*** (93.8, 162)	2.21 (2.03, 2.40)	3.05 (2.63, 3.47)	38.2** (18.9, 60.5)
Ukraine	1503 (1356, 1672)	1867 (1385, 2442)	24.3 (−11.9, 66.3)	2.85 (2.57, 3.17)	4.33 (3.21, 5.67)	52.1*** (7.8, 103.4)	2.13 (1.92, 2.37)	2.47 (1.82, 3.25)	15.9 (−18.5, 56.0)
Republic of Moldova	39 (35, 43)	89 (79, 100)	129.1*** (96.6, 169.9)	0.87 (0.80, 0.96)	2.47 (2.19, 2.77)	183.5*** (143.3, 234)	0.96 (0.89, 1.06)	1.49 (1.32, 1.67)	54.9*** (33.6, 81.1)

Data are presented as mean with their 95% UIs with upper and lower bounds. Linear trends were tested by linear regression analysis.

ASDR, age-standardized death rate; UIs, uncertainty intervals.

* *P* < 0.05.

** *P* < 0.01.

*** *P* < 0.001.

**Figure 3.**
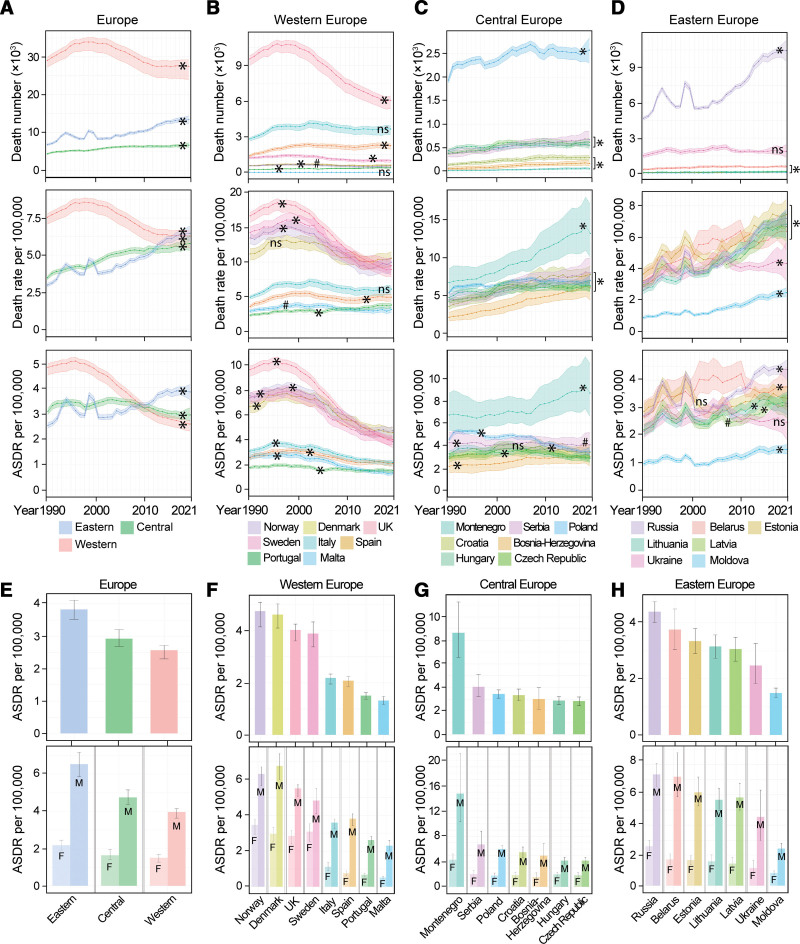
Mortality of aortic aneurysm in Europe. (A–D) AA-caused death number, death rate, and ASDR per 100,000 in Europe, Western Europe, Central Europe, and Eastern Europe from 1990 to 2021 (*P < 0.001). (E–H) AA-caused ASDR per 100,000 in European regions and representative countries in 2021. Data for both sexes are shown in the upper panel, and stratified data by sex are shown in the lower panel. Data are shown as mean with 95% UI. Linear trends were tested by linear regression analysis. *P < 0.001; #*P* < 0.05; ns, not significant. *P* values are also shown in Table 3. Data of other European countries are shown in Supplemental Digital Content, https://links.lww.com/AHJ/A4. AA, aortic aneurysm; ASDR, age-standardized death rate; F, women; M, men.

Western Europe reported the highest number of AA-related deaths in Europe from 1990 to 2021. However, the death rate per 100,000 population and ASDR decreased over time (*P* < 0.001; Fig. 3A and Table 3). The UK consistently had the highest number of AA-related deaths among Western European countries, but it decreased significantly in recent years (*P* < 0.001). Although the total death number remained relatively stable, almost all the Western European countries had decreased ASDR of AA from 1990 to 2021 (Fig. 3B). A significant decrease was especially observed in the UK, Norway, Sweden, Netherlands, Denmark, Finland, France, and Iceland (*P* < 0.001). However, in 2021, the ASDR of AA was still higher in these countries compared with other Western European countries, such as Portugal, Italy, Spain, and Malta (Fig. 3B, 3F and Table 3, and Supplemental Digital Content, https://links.lww.com/AHJ/A4).

Most Central European countries had relatively stable ASDR of AA from 1990 to 2021. Montenegro, where ASDR has remained high and continued to rise (*P* < 0.001), had the highest ASDR in Europe in 2021. In contrast, Poland experienced a significant decline in ASDR (*P* < 0.001), but both its death rate and death number increased during the same period (*P* < 0.001), likely reflecting the impact of its large and aging population. Indeed, Poland recorded the highest number of AA-related deaths in Central Europe (Fig. 3C, 3G, and Table 3).

The ASDR of AA increased constantly in most Eastern European countries (*P* < 0.001), except Ukraine and Estonia. In 2021, Eastern Europe had a higher ASDR of AA than Western Europe and Central Europe. Russia recorded the highest number of AA-related deaths in all ages in Central Europe (Fig. 3D, 3E, and Table 3).

### 3.4. Mortality of aortic aneurysm in Asia

We also explored data among Asian countries using existing GBD regions including Central Asia, South Asia, East Asia, and Southeast Asia, along with data from Japan, the Republic of Korea, and Singapore—originally categorized under the High-Income Asia Pacific GBD region (Table 4).

**Table 4:** Death number, death rates and age-standardized death rates in studied Asian countries in 1990 and 2021 along with the percent changes.

Location	Death number 1990	Death number 2021	% change in death number 1990, 2021	Death rate per 100,000 1990	Death rate per 100,000 2021	% change in death rate per 100,000 1990, 2021	ASDR per 100,000 1990	ASDR per 100,000 2021	% change in ASDR 1990, 2021
South Asia	3457 (2168, 5573)	15979 (11379, 23410)	362.2** (246, 536.3)	0.32 (0.20, 0.51)	0.87 (0.62, 1.27)	173.7** (104.9, 276.8)	0.71 (0.45, 1.13)	1.22 (0.88, 1.78)	71.6** (27.9, 136.1)
Bhutan	1 (1, 2)	8 (5, 12)	478.2** (211.7, 834.3)	0.23 (0.15, 0.34)	1.09 (0.68, 1.61)	381.3** (159.5, 677.8)	0.78 (0.51, 1.16)	1.49 (0.93, 2.16)	91.6** (5.3, 201.2)
Pakistan	423 (285, 647)	1472 (1081, 2066)	248.2** (142.9, 386.1)	0.38 (0.26, 0.58)	0.62 (0.46, 0.88)	64.3** (14.6, 129.4)	0.84 (0.57, 1.29)	1.48 (1.06, 2.04)	75.8** (21.9, 148.4)
Bangladesh	334 (196, 596)	1458 (952, 2422)	336.9** (192.4, 595.5)	0.31 (0.18, 0.55)	0.89 (0.58, 1.47)	189.6** (93.8, 361)	0.82 (0.49, 1.42)	1.21 (0.80, 1.99)	48.6** (2.2, 131.6)
India	2652 (1578, 4344)	12805 (9107, 18763)	382.9** (258.3, 576.7)	0.31 (0.18, 0.51)	0.91 (0.64, 1.33)	191.2** (116.1, 308.1)	0.68 (0.40, 1.08)	1.20 (0.86, 1.74)	77.5** (30.0, 149.6)
Nepal	48 (27, 86)	236 (162, 368)	394.2** (221.5, 668.4)	0.25 (0.14, 0.44)	0.76 (0.52, 1.18)	209.1** (101.1, 380.6)	0.64 (0.37, 1.13)	1.19 (0.82, 1.85)	84.6** (24.4, 177.4)
Central Asia	430 (374, 513)	1443 (1280, 1615)	235.7** (173.5, 299.1)	0.62 (0.54, 0.74)	1.51 (1.34, 1.69)	142.9** (97.8, 188.7)	0.94 (0.81, 1.13)	1.98 (1.77, 2.21)	110.1** (71.5, 150.9)
Armenia	117 (96, 142)	397 (330, 467)	238.4** (155.2, 351.6)	3.43 (2.81, 4.16)	13.24 (11.02, 15.60)	286.4** (191.4, 415.7)	4.55 (3.71, 5.57)	9.16 (7.61, 10.81)	101.4** (51.4, 170.2)
Georgia	37 (31, 44)	173 (146, 200)	369.4** (257.2, 500.7)	0.67 (0.56, 0.79)	4.80 (4.06, 5.56)	618.6** (446.8, 819.7)	0.61 (0.51, 0.72)	2.88 (2.43, 3.35)	374.2** (260.2, 510)
Kazakhstan	180 (147, 231)	377 (302, 462)	109.4** (48.8, 187.9)	1.10 (0.90, 1.41)	1.99 (1.59, 2.44)	81.1** (28.7, 149)	1.46 (1.19, 1.87)	2.23 (1.80, 2.71)	52.8* (8.1, 112.2)
Turkmenistan	19 (15, 23)	72 (53, 103)	285.1** (154.3, 468.3)	0.50 (0.42, 0.61)	1.39 (1.03, 1.99)	176.2** (82.4, 307.5)	1.03 (0.84, 1.26)	1.88 (1.42, 2.67)	83.0** (21.2, 165.9)
Azerbaijan	35 (26, 46)	130 (81, 212)	276.6** (116.8, 521)	0.47 (0.36, 0.63)	1.24 (0.77, 2.01)	162.7** (51.3, 333.3)	0.73 (0.55, 0.96)	1.37 (0.93, 2.08)	87.7** (15.1, 189.9)
Uzbekistan	24 (18, 34)	231 (186, 285)	852.5** (540.8, 1241.2)	0.12 (0.09, 0.16)	0.68 (0.54, 0.83)	483.2** (292.3, 721.1)	0.21 (0.16, 0.30)	1.04 (0.85, 1.28)	386.0** (225.1, 595)
Kyrgyzstan	8 (7, 9)	36 (29, 44)	350.3** (236, 488.1)	0.18 (0.16, 0.21)	0.53 (0.42, 0.64)	192.9** (118.5, 282.5)	0.28 (0.24, 0.33)	0.79 (0.63, 0.96)	185.5** (114, 272.3)
Southeast Asia	2069 (1670, 2586)	7391 (6476, 8513)	257.3** (178.3, 354.1)	0.44 (0.36, 0.56)	1.06 (0.93, 1.22)	138.2** (85.5, 202.7)	1.03 (0.83, 1.29)	1.39 (1.21, 1.60)	34.6** (5.7, 70.2)
Malaysia	268 (217, 323)	971 (812, 1159)	262.8** (167.4, 386.3)	1.52 (1.23, 1.83)	3.05 (2.55, 3.64)	101.5** (48.5, 170.1)	3.30 (2.66, 4.00)	4.04 (3.38, 4.88)	22.4 (−9.2, 65.7)
Thailand	546 (418, 723)	2134 (1640, 2755)	290.5** (165.6, 469.3)	0.96 (0.74, 1.27)	3.20 (2.46, 4.13)	232.4** (126.1, 384.6)	2.02 (1.53, 2.71)	2.01 (1.55, 2.58)	−0.4** (−32.8, 44.4)
Philippines	292 (251, 337)	950 (779, 1119)	225.8** (162.9, 310)	0.46 (0.40, 0.53)	0.84 (0.69, 0.99)	81.3** (46.3, 128.1)	1.22 (1.07, 1.39)	1.35 (1.12, 1.58)	10.6** (−11.1, 36.1)
Viet Nam	238 (172, 324)	895 (656, 1193)	275.6** (158.5, 483.6)	0.35 (0.25, 0.47)	0.89 (0.65, 1.19)	155.6** (75.9, 297.1)	0.67 (0.48, 0.90)	1.07 (0.78, 1.42)	60.4** (12.1, 146.4)
Seychelles	1 (1, 1)	1 (1, 2)	76.5** (25.5, 146.7)	0.84 (0.69, 1.00)	1.03 (0.70, 1.44)	22** (−13.3, 70.5)	1.08 (0.88, 1.29)	1.07 (0.74, 1.51)	−0.8 (−28.8, 37.9)
Indonesia	497 (328, 674)	1820 (1246, 2446)	266** (135.2, 430.8)	0.27 (0.18, 0.36)	0.65 (0.45, 0.88)	142.8** (56, 252)	0.63 (0.41, 0.87)	1.03 (0.71, 1.36)	62.2** (4.4, 130.4)
Myanmar	150 (88, 225)	392 (296, 514)	160.8** (73.2, 314.3)	0.37 (0.22, 0.56)	0.70 (0.53, 0.91)	86.9** (24.1, 196.9)	0.80 (0.47, 1.16)	0.96 (0.73, 1.26)	20.8** (−18.5, 84.7)
GBD East Asia	2936 (2374, 3719)	10199 (8229, 12817)	247.4** (139, 387.6)	0.24 (0.20, 0.31)	0.69 (0.56, 0.87)	187.1** (97.5, 303.1)	0.36 (0.29, 0.44)	0.50 (0.41, 0.63)	40.0** (−1.6, 92.0)
Japan	4797 (4448, 4982)	23815 (19180, 26463)	396.4** (327.8, 435.2)	3.81 (3.53, 3.96)	18.65 (15.02, 20.72)	389.2** (321.6, 427.4)	2.92 (2.68, 3.04)	5.07 (4.33, 5.47)	73.6** (60.4, 81.6)
Republic of Korea	422 (280, 603)	1767 (1441, 2093)	318.7** (157.3, 604.3)	0.95 (0.63, 1.36)	3.43 (2.79, 4.06)	259.3** (120.8, 504.3)	1.84 (1.25, 2.57)	1.92 (1.56, 2.27)	4.1 (−34.2, 68.9)
North Korea	87 (63, 118)	175 (131, 232)	99.9** (40.3, 187.6)	0.42 (0.30, 0.58)	0.66 (0.50, 0.88)	55.9** (9.5, 124.4)	0.55 (0.41, 0.73)	0.55 (0.41, 0.72)	−1.3* (−29.0, 42.0)
China	2648 (2088, 3416)	9033 (7053, 11644)	241.2** (125.1, 398.1)	0.23 (0.18, 0.29)	0.63 (0.50, 0.82)	182.1** (86.2, 311.9)	0.33 (0.27, 0.42)	0.46 (0.36, 0.59)	37.2** (−7.5, 95.7)
Singapore	53 (50, 55)	177 (158, 191)	237.1** (202, 267.7)	1.73 (1.63, 1.82)	3.10 (2.75, 3.34)	79.3** (60.7, 95.6)	2.65 (2.49, 2.79)	2.15 (1.91, 2.32)	−18.9** (−26.7, −11.6)

Data are presented as mean with their 95% UIs with upper and lower bounds. Linear trends were tested by linear regression analysis.

ASDR, age-standardized death rate; UIs, uncertainty intervals.

* *P* < 0.01.

** *P* < 0.001.

**Figure 4.**
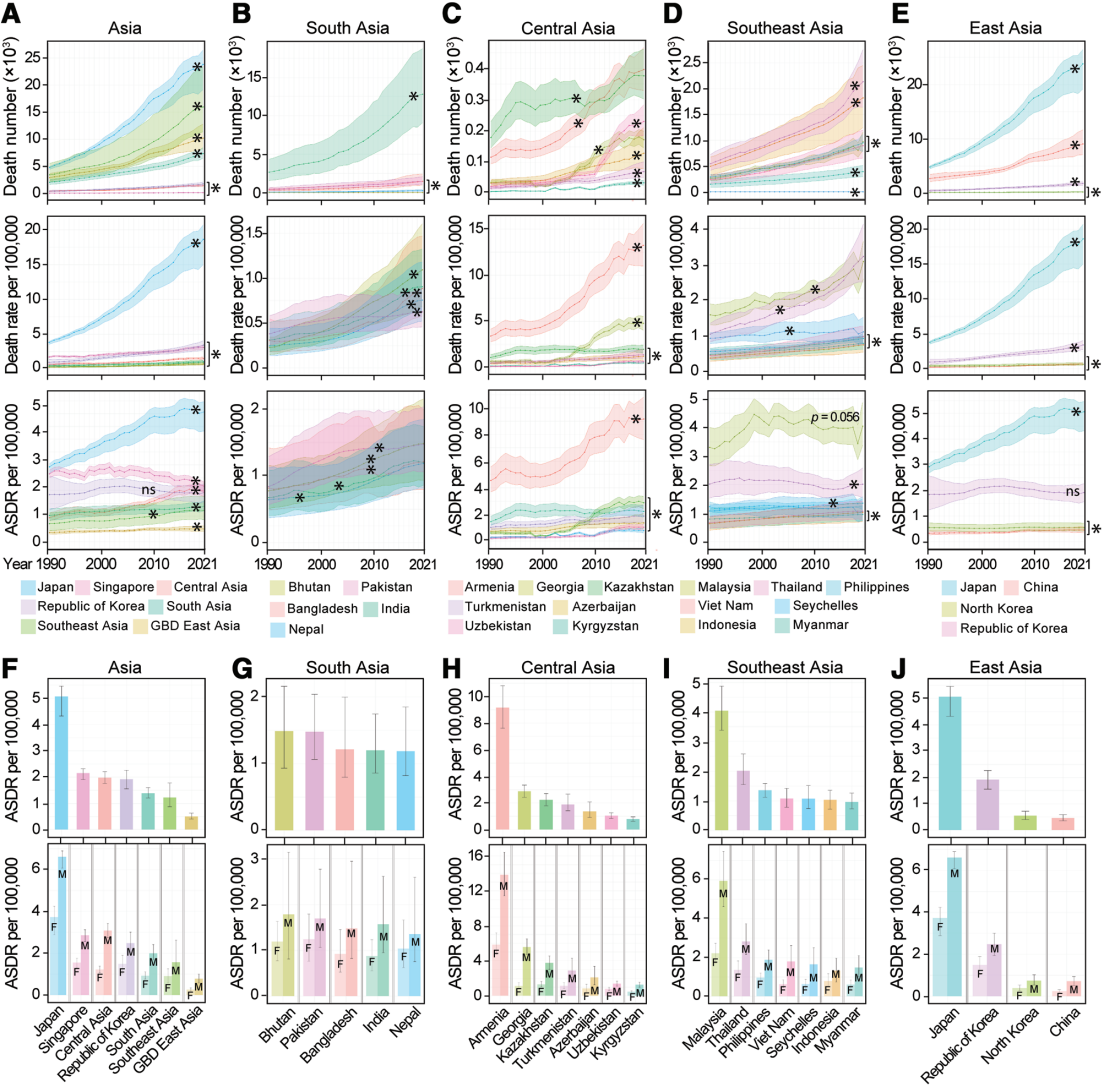
Mortality of aortic aneurysm in Asia. (A–E) AA-related death number, death rate and ASDR per 100,000 in Asia, South Asia, Central Asia, Southeast Asia and East Asia from 1990 to 2021. (F–J) AA-caused ASDR per 100,000 in Asian regions and representative countries in 2021. Data for both sexes are shown in the upper panel, and stratified data by sex are shown in the lower panel. East Asia in GBD 2021 study (GBD East Asia) contains China and North Korea. Data are shown as mean with 95% UI. Linear trends were tested by linear regression analysis. *P <0.001; ns: not significant. *P* values are also shown in Table 4. Data of other Asian countries are shown in Supplemental Digital Content, https://links.lww.com/AHJ/A4. AA, aortic aneurysm; ASDR, age-standardized death rate; F, women; GBD, Global Burden of Disease; M, men.

The AA mortality increased constantly in Japan from 1990 to 2021 (*P* < 0.001), making it the country with the highest AA-related death number, death rate per 100,000 population in all ages in the world and ASDR (5.07 deaths per 100,000 (95% UI: 4.33–5.47)) in Asia in 2021, almost twice as much as the other Asian countries (Figs. 1 and 4). Singapore had similar ASDR of AA as in Japan in 1990, but it decreased during the years (*P* < 0.001), ending in 2.15 (95% UI: 1.91–2.32) ASDR per 100,000 in 2021 (Fig. 4A, 4F, and Table 4).

Although the AA-related ASDR value had been kept at low levels in most of the Asian countries, it increased significantly from 1990 to 2021, by 71.6% in South Asia, 110.1% in Central Asia, 34.6% in Southeast Asia, and 40% in GBD East Asia (all *P* < 0.001). Armenia had constantly much higher ASDR of AA throughout the years. In 2021, Armenia had the highest ASDR of AA in the world, with 9.16 (95% UI: 7.61–10.81) deaths per 100,000. India, the second biggest population in the world in 2021, recorded the second most AA-related death number worldwide, with 382.9% (95% UI: 258.3%–576.7%) increase from 1990 (*P* < 0.001; Fig. 4B, 4C, 4G, 4H, and Table 4).

China and Democratic People’s Republic of Korea (North Korea) from GBD East Asia and most countries in Southeast Asia had constantly lower levels of ASDR of AA. Republic of Korea in East Asia and Malaysia in Southeast Asia had much higher ASDR, but it remained stable over the time (*P* > 0.05). China, with the biggest population in the world in 2021, reported 9033 (95% UI: 7053–11,644) AA-related deaths in 2021, ranked number 6 globally. Although the ASDR in China increased 37.2% from 1990 to 2021 (*P* < 0.001), it was still at lower level with 0.46 (95% UI: 0.36–0.59) deaths per 100,000 in 2021 (Fig. 4D, 4E, 4I, 4J, and Table 4).

### 3.5. Sex disparity in mortality of aortic aneurysm

**Figure 5.**
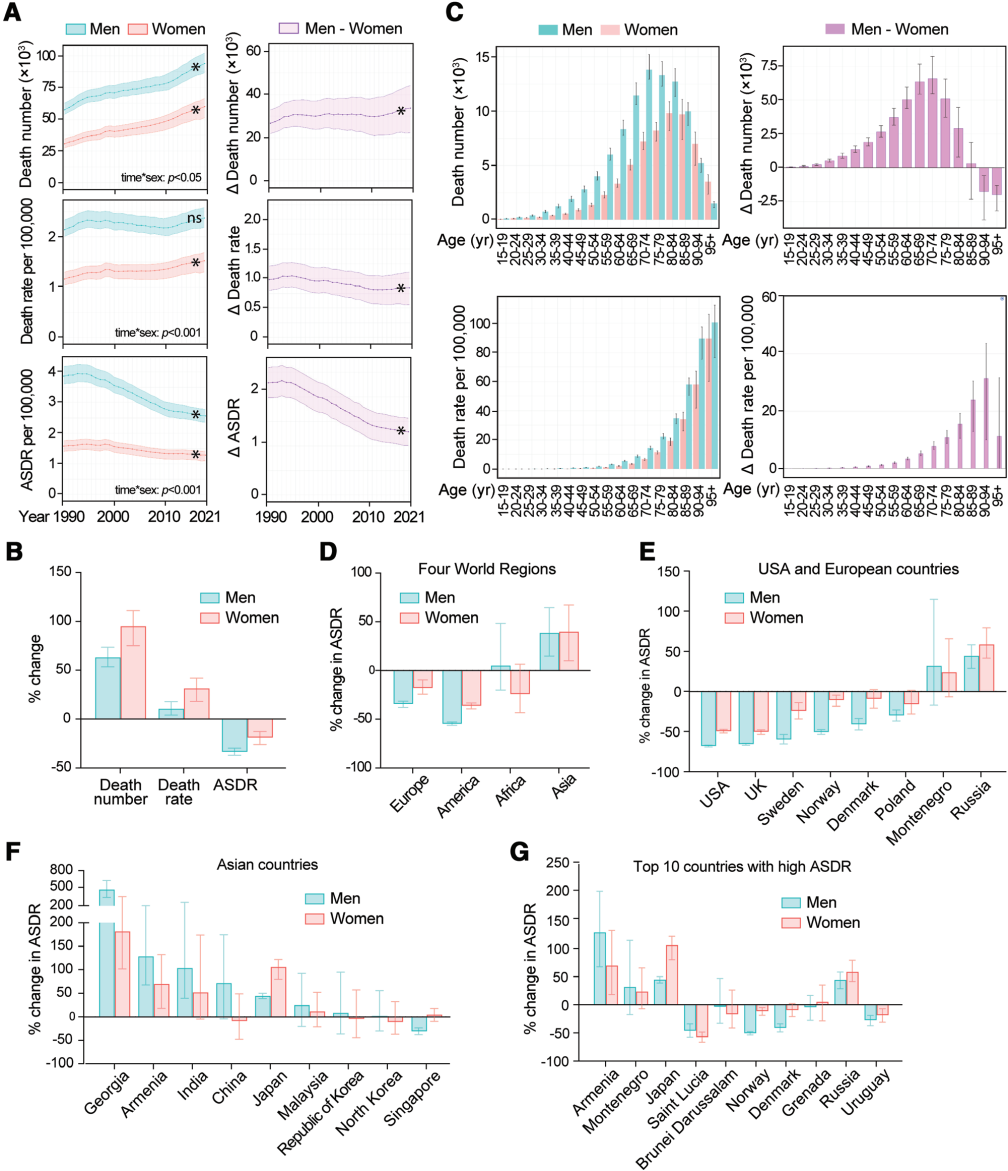
Sex differences in the mortality of aortic aneurysm. (A) Global AA-caused death number, death rate, and ASDR per 100,000 in men (blue) and women (red), as well as the difference between men and women (pink, right panel) from 1990 to 2021. (B) Percentage changes in global death number, death rate, and ASDR of AA in men and women in 2021 compared with 1990. (C) Global death number (upper panels) and death rates per 100,000 (lower panels) in men (blue) and women (red), as well as the difference between men and women (pink, right panel), stratified by age groups in 2021. (D–G) Percentage changes in AA-caused ASDR of men and women in 2021 compared with 1990, in the indicated world regions and countries. Data are shown as mean with 95% UI. Linear trends were tested by linear regression analysis. Interactions between time and sex were analyzed by 2-way analysis of variance. *P <0.001; ns, not significant. *P* values are also shown in Table 5. AA, aortic aneurysm; ASDR, age-standardized death rate; UI, uncertainty interval.

We next examined sex disparity in the global burden of AA. From 1990 to 2021, men consistently had higher numbers of AA-related deaths, death rates per 100,000, and ASDR compared with women (Fig. 5A). Although both sexes experienced increases in AA-related deaths and death rates over time, the trends differed significantly by sex, as indicated by a significant interaction between time and sex in the trend analysis (Fig. 5A). Between 1990 and 2021, the sex gap in the number of deaths widened by 26.3%, while the gaps in death rate and ASDR narrowed by 14% and 43.8%, respectively (both *P* < 0.001; Fig. 5A and Table 5).

**Table 5:** Death number, death rates and age-standardized death rates of men and women in 1990 and 2021 along with the percentage changes.

Location	Death number 1990	Death number 2021	% change in death number 1990 - 2021	Death rate per 100,000 1990	Death rate per 100,000 2021	% change in death rate per 100,000 1990 - 2021	ASDR per 100,000 1990	ASDR per 100,000 2021	% change in ASDR 1990, 2021
Global									
Men	57557 (53978, 62641)	93864 (86610, 102153)	63.1*** (53.4, 73.7)	2.14 (2.01, 2.33)	2.37 (2.19, 2.58)	10.6 (4.1, 17.9)	3.87 (3.61, 4.18)	2.57 (2.36, 2.79)	−33.6*** (−37.1, −29.8)
Women	30796 (27622, 34388)	60063 (51303, 66298)	95.0*** (75.2, 110.9)	1.16 (1.04, 1.30)	1.53 (1.30, 1.69)	31.3*** (18.0, 42.0)	1.58 (1.41, 1.76)	1.28 (1.10, 1.42)	−18.8*** (−26.0, −12.8)
Four world regions									
** **Europe									
Men	27265 (26285, 27901)	32117 (29948, 33666)	17.8 (11.4, 23.1)	7.03 (6.78, 7.20)	7.80 (7.27, 8.17)	10.9 (4.8, 15.9)	7.16 (6.85, 7.34)	4.69 (4.38, 4.92)	−34.5*** (−37.8, −31.5)
Women	13626 (12498, 14236)	17966 (15447, 20122)	31.8*** (21.4, 44.2)	3.29 (3.01, 3.43)	4.11 (3.54, 4.61)	25.2*** (15.3, 37)	2.07 (1.91, 2.17)	1.70 (1.50, 1.92)	−17.9*** (−24.3, −9.5)
** **America									
** **Men	17791 (17052, 18268)	18999 (17807, 19882)	6.8** (2.6, 11.2)	5.05 (4.84, 5.19)	3.77 (3.54, 3.95)	−25.3*** (−28.3, −22.2)	6.99 (6.64, 7.21)	3.17 (2.97, 3.32)	−54.6*** (−56.4, −52.8)
** **Women	8988 (8042, 9467)	12597 (10919, 13638)	40.1*** (32.6, 46.2)	2.47 (2.21, 2.60)	2.41 (2.09, 2.61)	−2.5*** (−7.8, 1.7)	2.55 (2.28, 2.69)	1.63 (1.42, 1.76)	−36.2*** (−39.3, −33.4)
** **Africa									
Men	2381 (998, 3936)	5779 (3187, 9075)	142.7*** (81.6, 247.7)	0.76 (0.32, 1.25)	0.84 (0.46, 1.32)	11.2 (−16.8, 59.3)	2.06 (0.88, 3.37)	2.16 (1.21, 3.36)	4.9* (−20, 48.4)
Women	1832 (1135, 2870)	3346 (2128, 4907)	82.6*** (32.9, 167.7)	0.58 (0.36, 0.91)	0.48 (0.30, 0.70)	−17.1*** (−39.6, 21.6)	1.58 (1.01, 2.48)	1.20 (0.76, 1.75)	−24.2*** (−43.3, 6.6)
** **Asia									
Men	9963 (8304, 13672)	36786 (32545, 43569)	269.2*** (199.6, 347.4)	0.61 (0.51, 0.84)	1.56 (1.38, 1.85)	155.3*** (107.2, 209.3)	1.31 (1.11, 1.75)	1.81 (1.60, 2.11)	38.6*** (14.9, 64.4)
Women	6277 (4967, 8481)	26055 (21132, 29692)	315.1*** (214.9, 410.8)	0.41 (0.32, 0.55)	1.15 (0.93, 1.31)	183.1*** (114.8, 248.4)	0.75 (0.61, 0.98)	1.05 (0.85, 1.21)	39.8*** (10, 67.1)

Data are presented as mean with their 95% UIs with upper and lower bounds. Linear trends were tested by linear regression analysis.

ASDR, age-standardized death rate; UIs, uncertainty intervals.

* *P* < 0.05.

** *P* < 0.01.

*** *P* < 0.001.

Globally, women experienced a greater increase in AA-related death number (95% versus 63.1%) and death rate (31.3% versus 10.6%) compared with men, along with a much smaller decline in ASDR (−18.8% versus −33.6%; Fig. 5B and Table 5). These trends suggest that the worsened outcome in women has contributed to the narrowing sex gap in AA-related mortality.

Sex differences in AA-related mortality varied with age, with deaths peaking at 70 to 79 years old in men and 80 to 89 years old in women. Men had more deaths up to age 80 to 84 years, with the largest gap at 70 to 74 years old, while women surpassed men after age 90 years (Fig. 5C). As expected, these numbers reflected the age-group population size. Death rate per 100,000 increased steadily with age in both sexes and remained higher in men (Fig. 5C). However, the sex difference in death rates narrowed in those aged 95^+^ years, likely due to improved outcomes in elderly men following adherence to the treatment guidelines of aortic disease^[25]^.

Sex disparity in AA-related mortality differs across world regions. In Europe, AA-related deaths increased more in women (31.8%; *P* < 0.001) than in men (17.8%; *P* > 0.05). Similarly, in America, deaths rose by 40.1% in women compared with just 6.8% in men (both *P* < 0.01). In Asia, deaths increased dramatically in both men (269.2%) and women (315.1%) (both *P* < 0.001), while in Africa, it grew by 142.7% in men and 82.6% in women (both *P* < 0.001; Table 5).

In Europe and America, ASDR generally declined more in men than in women (Fig. 5D). Notably, the differences were particularly pronounced in Sweden, Norway, and Denmark, where men experienced percentage changes of −59.7%, −50.6% and −40.9%, respectively, compared with smaller declines in women (−24.2%, −10.8%, and −9.0%; Fig. 5E). However, in Japan and Russia, women had a greater increase than men, with 106.1% versus 44.7% increase in Japan and 58.5% versus 44.5% increase in Russia. In China, ASDR in men increased 71.6%, but it decreased 8.8% in women. Conversely, in Singapore, men had a 31.0% decrease while women experienced a slight increase of 4.7% (Fig. 5E, 5F). Among the countries with the highest ASDR, Armenia increased 128.6% in men and 69.9% in women; however, Grenada maintained consistently high levels without significant change throughout the study period (Fig. 5G, Table 5, and Supplemental Digital Content, https://links.lww.com/AHJ/A4).

These data underscore the significant role of sex differences in AA-caused mortality. The impact of sex difference varies with age, being lower in younger and oldest age groups. It fluctuates across different countries over time, influencing the overall AA-caused mortality.

### 3.6. Risk factors contributing to aortic aneurysm-related deaths

**Figure 6.**
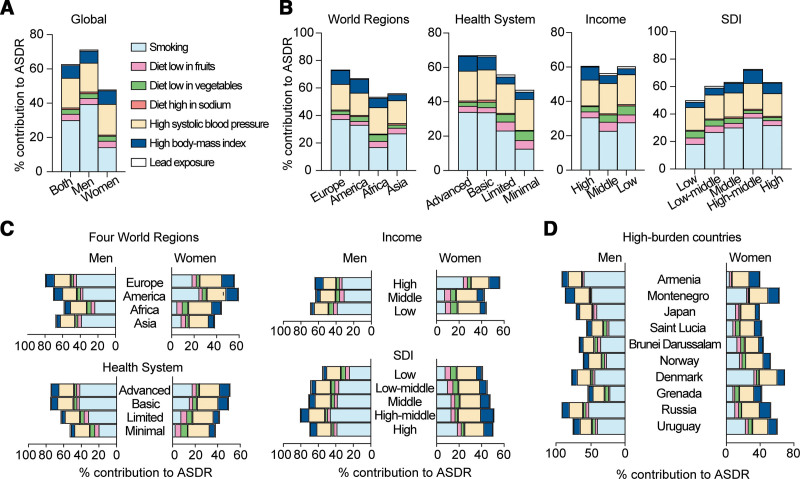
Percentage contribution of risk factors to AA-caused ASDR at global level in 2021. (A) Percentage contribution of various risk factors to global AA-ASDR, stratified by sex. (B) Percentage contribution of risk factors to AA-related ASDR among world regions, including Four World Regions, Health System Grouping, Commonwealth (Income), and SDI. (C) Percentage contribution of risk factors to AA-related ASDR analyzed by Four World Regions, Health System Grouping, Commonwealth (Income) and SDI, stratified by sex. (D) Percentage contribution of risk factors to AA-caused ASDR in the top 10 countries with the highest ASDR in the world. AA, aortic aneurysm; ASDR, age-standardized death rate; SDI, social demographic index.

Using the comparative risk assessment framework in GBD 2021, smoking, high systolic blood pressure, high body-mass index, dietary factors, and lead exposure were identified as significant contributors to AA-related mortality. The risk factors that contributed to the ASDR of AA in 2021 were smoking (30%, 95% UI: 25.5%–34.9%), high systolic blood pressure (17.3%, 95% UI: 13.0%–21.9%), high body-mass index (7.4%, 95% UI: 4.0%–12.7%), low consumption of fruits (3.6%, 95% UI: 2.5%–4.8%) and vegetables (2.9%, 95% UI: 1.9%–4.0%), high sodium intake (0.9%, 95% UI: 0.1%–2.7%) and lead exposure (0.7%, 95% UI: −0.1% to 1.7%; Fig. 6A and Supplemental Digital Content, https://links.lww.com/AHJ/A4). We analyzed the percentage contribution of these risk factors across different regions of the world and the top 10 countries with the highest ASDR of AA for both men and women (Fig. 6B–6D).

#### 3.6.1. Smoking

The biggest risk factor for AA-caused death was smoking. The percentage contribution of smoking to ASDR of AA was higher in regions with high disease burden of AA. For example, smoking contributed 37.2% (95% UI: 31.8%–42.9%) and 33.1% (95% UI: 27.5%–39.0%) of ASDR in Europe and America while as 16.9% (95% UI: 13.7–20.4%) in Africa, and it contributed 33.9% (95% UI: 28.6%–39.3%) of ASDR in regions having Advanced Health System, but only 12.5% (95% UI: 7.2%–17.5%) in regions with Minimal Health System (Fig. 6B). The contribution of smoking to AA-caused death was particularly high in the following countries such as Lebanon (48.5%), Georgia (48.1%), Belarus (47.8%), Greece (46.8%), Albania (46.3%), Jordan (46.3%), Bosnia and Herzegovina (45.5%), and China (44.7%; Supplemental Digital Content, https://links.lww.com/AHJ/A4).

The percentage contribution of smoking to AA-caused ASDR was much higher in men (39.3%, 95% UI: 33.6%–45.5%) than in women (14.2%, 95% UI: 11.2%–17.4%) globally (Fig. 6A and Supplemental Digital Content, https://links.lww.com/AHJ/A4), a trend which was maintained among all the world regions and countries with high AA burden (Fig. 6C, 6D). Smoking was the leading risk factor for women in many countries with high burden of AA-caused ASDR, such as in Denmark (33.4%), Montenegro (24.8%), and Uruguay (23.2%; Fig. 6D and Supplemental Digital Content, https://links.lww.com/AHJ/A4).

#### 3.6.2. High systolic blood pressure

The second risk factor for AA-caused death was high systolic blood pressure, accounting for 17.3% (95% UI: 13.0%–21.9%) of AA-caused ASDR globally (Fig. 6A). It contributed the most in Indonesia (26.3%), Hungary (25.9%), Republic of Moldova (24.7%), Sierra Leone (24.4%), Kazakhstan (23.9%), Lithuania (23.4%), Malaysia (23.4%), and Georgia (22.8%; Supplemental Digital Content, https://links.lww.com/AHJ/A4).

High systolic blood pressure was the biggest risk factor for AA-caused death in women globally and in most of the regions that we have explored, except America and Commonwealth High Income region where smoking had the highest contribution (Fig. 6C). It was also the biggest risk factor for women in many countries with a high burden of AA-caused ASDR, such as in Armenia, Japan, Saint Lucia, Brunei Darussalam, Norway, Grenada, and Russia (Fig. 6D).

#### 3.6.3. High body-mass index

High body-mass index contributed to 7.4% (95% UI: 4.0%–12.7%) of AA-caused ASDR globally. Its contribution to AA-caused ASDR was higher in regions with high disease burden of AA (Fig. 6B). For example, it contributed 10% (95% UI: 5.3%–17.2%) and 9.9% (95% UI: 5.3%–16.9%) of the AA-caused ASDR in Europe and America while 4.4% (95% UI: 2.4%–7.0%) in Asia, and it contributed 8.6% (95% UI: 4.6%–14.7%) of the AA-caused ASDR in regions having advanced health system, but only 4.3% (95% UI: 2.4%–7.1%) in regions with minimal health system (Fig. 6B and Supplemental Digital Content, https://links.lww.com/AHJ/A4). The contribution of high body-mass index to AA-caused death was particularly high in Middle East, Central, and Eastern Europe, and North Africa, such as in Qatar, Kuwait, Hungary, Saudi Arabia, the Republic of Moldova, the United Arab Emirates, Libya, and the Syrian Arab Republic. In general, it played more important roles in women than in men for AA-caused ASDR.

#### 3.6.4. Diet with low fruits and vegetables but high salt

Diet with low consumption of fruits (3.6%, 95% UI: 2.5%–4.8%) and vegetables (2.9%, 95% UI: 1.9%–4.0%), but high sodium intake (0.9%, 95% UI: 0.1%–2.7%) were also important risk factors for AA-caused ASDR globally (Fig. 6A). Their contributions were much lower than smoking, high blood pressure and high body-mass index. It was similar between men and women, and it did not vary a lot among the world regions with different burdens of AA (Fig. 6B). However, they are important risk factors for particular countries.

Diet with low fruits or vegetables contributed the most to African countries, such as Zimbabwe, Togo, and Sierra Leone; Asian countries such as Mongolia and countries in Oceania such as Vanuatu. Diet with high salt played important roles in the burden of AA-caused death in China, the Republic of Korea, North Korea, and Singapore in Asia, and in Central and Eastern (Balkans) European countries such as the Czech Republic, Bosnia and Herzegovina, Bulgaria, North Macedonia, Slovakia, and Montenegro.

#### 3.6.5. Lead exposure

Lead exposure contributed to 0.7% (95% UI: −0.1% to 1.7%) of AA-caused ASDR globally. Its contribution was particularly high in the Middle East and Southern Asian countries including Nepal, Yemen, Afghanistan, Bhutan, Bangladesh, and Iran; in African countries such as Somalia, Egypt, and Ethiopia; and in Central American countries such as Guatemala, Haiti, and Honduras.

### 3.7. Risk factors contributing to aortic aneurysm-related deaths in Europe

**Figure 7.**
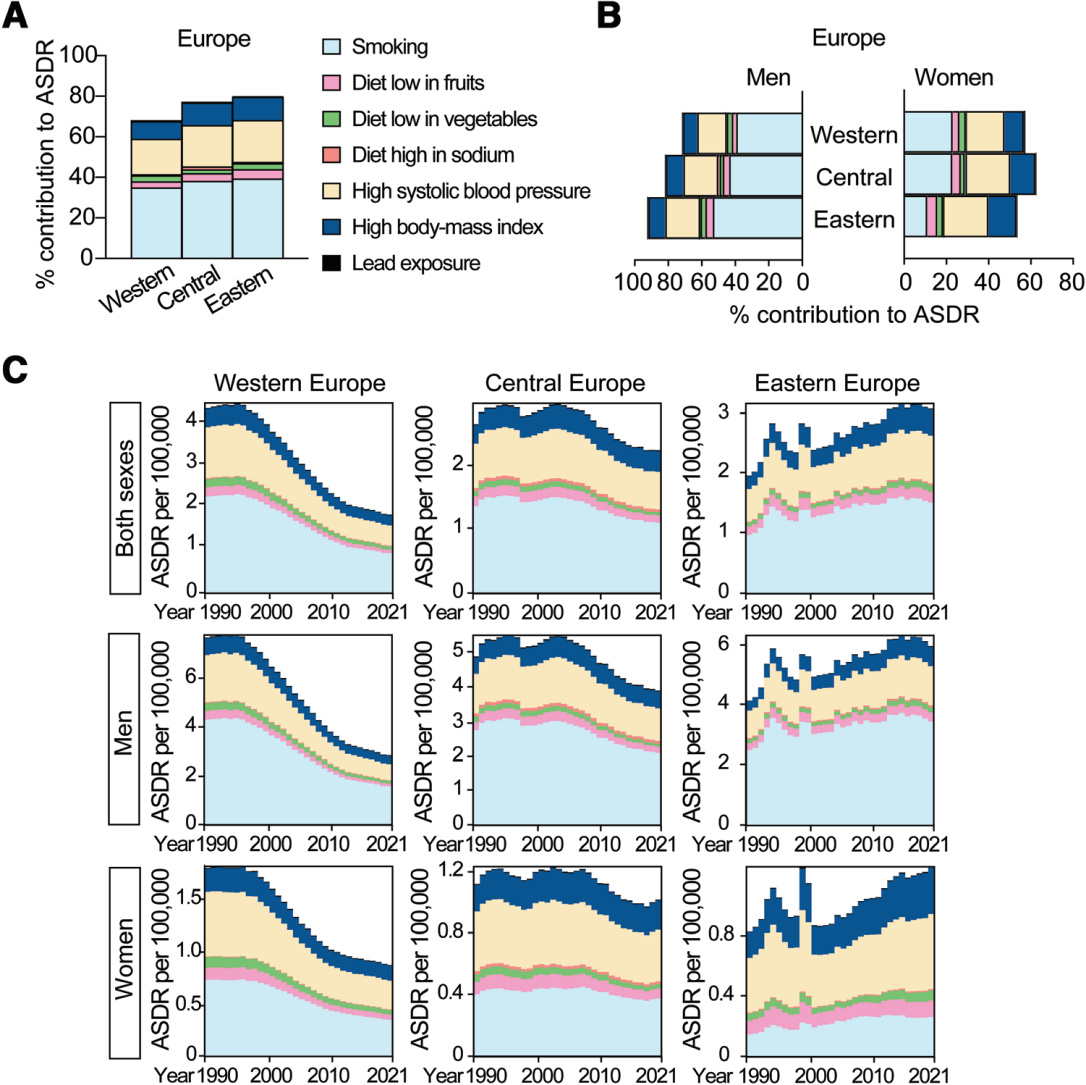
Percentage of AA-caused ASDR attributable to risk factors in Europe. (A) Percentage contribution of risk factors to AA-caused ASDR in Western Europe, Central Europe, and Eastern Europe in 2021. (B) Percentage contribution of risk factors to AA-related ASDR in Western Europe, Central Europe, and Eastern Europe, stratified by sex in 2021. (C) AA-caused ASDR attributable to risk factors in Western Europe, Central Europe, and Eastern Europe, stratified by sex from 1990 to 2021. AA, aortic aneurysm; ASDR, age-standardized death rate.

In Europe in 2021, smoking was the leading risk factor for AA-caused death in all the regions for both men and women, except for women in Eastern Europe where high systolic blood pressure was the biggest risk factor (Fig. 7A and 7B). High body-mass index contributed more to AA-caused death in women than in men in all the European regions, and high salt diet played a particular role in Central Europe, especially in men (Fig. 7B).

To understand the reasons for the differential changes in ASDR of AA in Western Europe (46.2% decrease) and in Eastern Europe (51.6% increase) from 1990 to 2021, we explored the ASDR attributable to different risk factors. All the known risk factors decreased in Western Europe in both men and women, and increased in Eastern Europe over the time. Smoking contributed the most to the change of AA-caused ASDR in men population, also in women from Western Europe. High systolic blood pressure played the second important role. In women from Eastern Europe, the increases in AA-caused ASDR were particularly attributable to high systolic blood pressure and followed by smoking and high body-mass index (Fig. 7C).

### 3.8. Risk factors contributing to aortic aneurysm-related deaths in Asia

**Figure 8.**
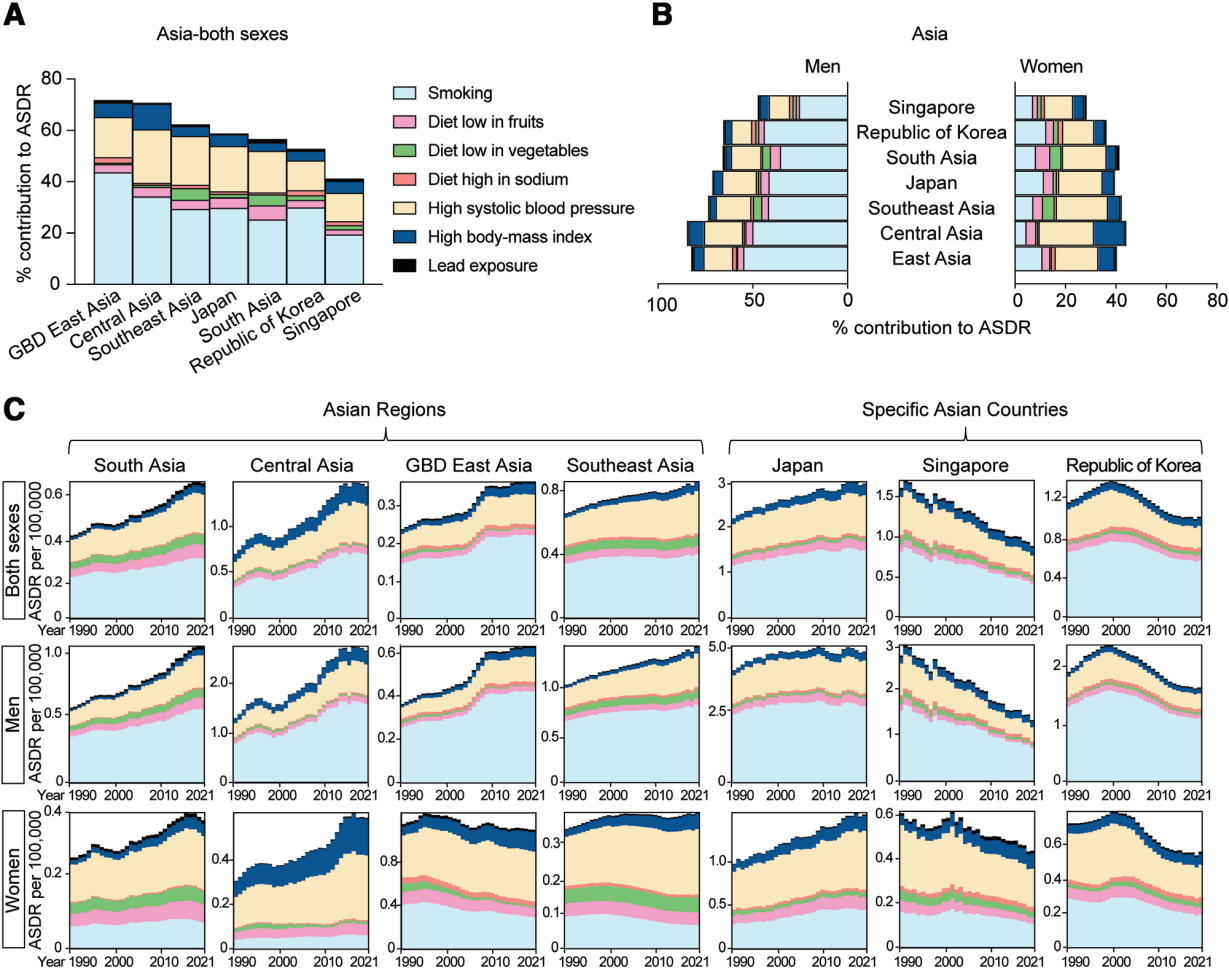
Percentage of AA-caused ASDR attributable to risk factors in Asia. (A) Percentage contribution of risk factors to AA-caused ASDR in South Asia, Central Asia, Southeast Asia, GBD East Asia including China and North Korea, Japan, the Republic of Korea, and Singapore in 2021. (B) Percentage contribution of risk factors to AA-related deaths in the above regions, stratified by sex, in 2021. (C) AA-caused ASDR attributable to risk factors in the above regions, stratified by sex, from 1990 to 2021. AA, aortic aneurysm; ASDR, age-standardized death rate; GBD, Global Burden of Disease.

Smoking was the biggest risk factor for AA-caused death in all Asian regions in men, but high systolic blood pressure was the biggest risk factor for AA-caused death in Asian women. This was observed in both Japan, the country with the continuously increasing AA-related death and the highest ASDR in Asia, and in Singapore, where the AA-caused ASDR was decreasing (Figs. 4A, 8A, and 8B).

The trend in AA-related ASDR attributable to all the risk factors across different regions of Asia from 1990 to 2021 is shown in Figure 8C. Over the 3 decades, the contribution of smoking steadily increased among men in most Asian regions, except for Singapore. In women, the contribution of smoking decreased in many regions, although it saw a dramatic increase in Japan. In Japan, AA-related ASDR attributable to smoking rose 53.6% among women, from 0.28 (95% UI: 0.22–0.36) per 100,000 in 1990 to 0.43 (95% UI: 0.31–0.59) in 2021. In contrast, the increase in men was only 12.6% (Supplemental Digital Content, https://links.lww.com/AHJ/A4). The contribution of high blood pressure and high body-mass index grew in both men and women across most regions in Asia, except Singapore. In addition, the increased contribution of high body-mass index was particularly prominent in women, especially in Central Asia and in Japan (Fig. 8C and Supplemental Digital Content, https://links.lww.com/AHJ/A4).

Interestingly, in contrast to the trends observed in most other Asian countries, the ASDR attributable to all the risk factors decreased in Singapore, with a more significant reduction in men than in women. In Japan, which reported the highest AA-related deaths and highest death rate per 100,000 globally, the ASDR attributable to all the risk factors increased, with a more pronounced rise observed in women (Fig. 8C).

## 4. Discussion

AA remains a significant global health burden. Despite the trend of decrease in ASDR, AA-related death numbers have risen over the past 3 decades worldwide, driven by population growth and increased life expectancy. A comprehensive temporal and geographical analysis of these trends is essential for identifying high-risk populations, guiding healthcare policies, and improving prevention strategies. In this study, we explored AA-related mortality data from the GBD Study 2021, assessing epidemiological trends and risk factors for AA-related mortality from 1990 to 2021 across regions, with a focus on age, sex, and socioeconomic factors. The findings aim to guide strategies for reducing AA mortality, even amidst a growing and aging population.

### 4.1. Global trends in aortic aneurysm-related mortality

From 1990 to 2021, the total AA-related deaths increased by 74.2%, reaching 153,927 deaths in 2021. The increase is influenced by population growth and aging demographics. Our findings indicate that AA-related death rates increase significantly with age, highlighting the vulnerability of older populations. This is in agreement with previous studies^[26]^. Consequently, countries with large populations and longer life expectancies bear a greater burden of AA-related deaths. Japan, India, and the USA reported the highest absolute numbers of AA-related deaths, while Japan, Armenia, Montenegro, Denmark, and Greece ranked highest in death rates per 100,000 people.

The ASDR, which accounts for population aging, shows a global decline, from 2.54 deaths per 100,000 in 1990 to 1.86 in 2021. This suggests that advancements in healthcare and preventive measures can help mitigate AA-related mortality, despite the growing absolute number of cases. After adjusting for age, Armenia, Montenegro, Japan, Saint Lucia, Brunei Darussalam, Norway, Denmark, Grenada, Russia, and Uruguay emerged as the top 10 countries with highest AA death rates. To better understand and address the high AA mortality in these nations, it is crucial to analyze the underlying risk factors. In addition, using ASDR allows for more accurate comparisons of death rates across different populations and time periods by eliminating the confounding effects of population growth and aging.

### 4.2. Regional variations in aortic aneurysm-related mortality

AA-related death rates varied significantly across world regions. While Europe and America have experienced a decline in ASDR, Asia has seen an increase. Africa shows high variability among countries, with no clear overall trend. Similar geographical disparity has been reported in a recent study analyzing AA mortality trends between 2000 and 2019 using WHO mortality database. The study found that the ASDR from aortic dissection and rupture combined showed decreasing trends in the UK, USA, and Canada, but an increasing trend in Japan^[27]^.

Interestingly, regions with advanced healthcare systems, high incomes, and high SDI, such as Western Europe, showed a decline in ASDR. However, they still bear a significant burden of AA-related deaths, suggesting that despite healthcare advancements reducing AA death rates, these regions continue to experience a high prevalence of lifestyle-related risk factors, such as smoking, hypertension, and obesity, significantly contributing to AA-related deaths. Moreover, many AA cases remain fatal due to their sudden onset and the challenge of timely intervention, particularly in cases of ruptured aneurysms or in clinically frail patients. This underscores that even in well-developed healthcare systems, AA remains a serious cause of mortality. In addition, countries with advanced healthcare systems may have better diagnostic capabilities, leading to better detection and reporting of AA-related deaths.

In contrast, ASDR of AA has increased in some world territories, including Japan, Eastern Europe, South Asia, and Central Asia. Our analysis indicates that many risk factors have contributed to the increase in AA-related deaths in both sexes. Among men, smoking is the most significant contributor, whereas hypertension and obesity (high body-mass index) play a larger role in women. Furthermore, limited access to healthcare, screening, and delays in emergency surgical intervention may have led to higher fatality rates in these regions. For example, Eastern Europe has faced ongoing challenges in healthcare transformation since the collapse of the Soviet Union in 1991, resulting in underfunded healthcare infrastructure, outdated equipment, emigration of medical professionals, and financial barriers^[28]^. These issues have limited timely and effective access to healthcare, contributing not only to poorer general health and increased AA risk, but also to delayed diagnosis and treatment, which may have exacerbated AA-related mortality^[28,29]^. Besides, rapid aging in Eastern European countries also contributed to the increased AA mortality^[30]^.

Japan bears the highest burden of AA-related death globally, both in absolute death numbers and death rate per 100,000 population. The rapidly aging population is no doubt the major contributor. ASDR may still be indirectly influenced by aging through increased vascular degeneration and comorbidities, which not only increase the incidence of AA but may also complicate the treatment^[31,32]^. Rising rates of smoking, hypertension, and obesity, particularly among women, also contribute to the growing AA burden, as shown by our study and others^[33]^. The high ASDR in Japan can also reflect improved detection and diagnosis due to Japan’s advanced healthcare infrastructure^[34]^. Easy and affordable access to advanced diagnosis technology including postmortem imaging, and a well-developed reporting system likely results in more accurate attribution of AA as a cause of death^[34,35]^. However, this also triggers other problems such as overburdened hospitals and healthcare workforce shortage, which may delay the treatments and worsen the outcomes of AA in clinical care^[34]^. Further research is needed to clarify the multifactorial drivers of the AA burden in Japan.

The rise in AA-related deaths highlights the urgent need for stronger public health measures, including smoking cessation programs, better hypertension management, and improved screening and surgical interventions. Without these measures, AA mortality may continue to increase, particularly as life expectancy rises in many middle-income countries.

### 4.3. Sex differences in aortic aneurysm-related mortality

Sex significantly influences the burden, progression, and risk factors associated with AA-related mortality^[26]^. Our findings show that the ASDR for AA was consistently higher in men than in women, likely due to a 4-fold higher prevalence of subclinical AA compared with women^[26]^. However, men exhibited only twice the AA-related ASDR across major global regions, suggesting worse outcomes in women. AA in women tends to rupture at smaller sizes and is more likely to experience rapid expansion and rupture, leading to higher death rates despite lower incidence^[36–38]^.

From 1990 to 2021, AA-related deaths and death rates in women have increased more rapidly than in men, contributing significantly to the global burden of AA, particularly in countries like Japan. We observed a narrowing gap in AA-related ASDR between men and women, likely attributable to both poorer outcomes in women and improved outcomes in men, at least partly due to the implementation of screening programs for abdominal AA for men aged 65 or above^[39,40]^. To date, screening programs are generally not recommended for women^[41]^, despite evidence that AA tends to progress more aggressively in women with more adverse outcomes.

The sex disparity in AA mortality has been shown in recent studies using the WHO mortality database, with men having approximately twice the ASDR of women in the UK, USA, Canada, and Japan. Although both sexes exhibited similar trends over time, the decline in ASDR from aortic rupture was more pronounced in men than in women across these 4 countries between 2000 and 2019^[27]^. In addition, another study using CDC WONDER (Centers for Disease Control and Prevention Wide‐Ranging Online Data for Epidemiologic Research) reported a more pronounced increase in ASDR from aortic dissection among women than men in the USA between 2012 and 2019^[42]^.

Taken together, these findings emphasize the need of implementing preventive measures and screening for undiagnosed AA in at-risk women to reduce mortality, particularly in light of the global trends of higher AA-related mortality in older women.

### 4.4. Risk factors contributing to aortic aneurysm-related deaths

Consistent with findings from the GBD 2019 study^[18]^, our study confirms that smoking continues to be a leading attributable risk factor for AA in men, while hypertension poses a greater risk for women across most of the studied countries. Although risk factors are known to differ between AAA and TAA, smoking has been reported to increase the risk of AAA by about 4 times^[43]^ and nearly doubles the risk of TAA^[44]^. Likewise, patients with hypertension have a 66% higher risk of developing AA^[45]^ and more than double the risk of developing TAA^[44,46]^. Moreover, both smoking and hypertension are well known to induce endothelial dysfunction, an emerging contributing factor to AA^[47,48]^.

Smoking is also the most significant risk factor for AA-related deaths, responsible for nearly 30% of cases globally^[43,44]^. Effective tobacco control in countries such as Sweden, Norway, and Denmark^[49]^ may have contributed to a decline in AA-related mortality. In contrast, Japan, which has the highest AA-related mortality in Asia, has weaker tobacco control policies^[50]^. The impact of smoking is particularly evident in regions with a high burden of AA, such as in Europe and America. Countries such as Lebanon, Georgia, and Belarus show alarmingly high smoking-related AA death rates, suggesting the urgent need for targeted smoking cessation programs.

Hypertension accounts for 17.3% of AA-related deaths, with a stronger impact on women, particularly in Eastern Europe and Asia. Countries such as Indonesia and Hungary underscore the need for better hypertension management.

As a growing pandemic^[51]^, obesity can induce chronic inflammation in aorta and contribute to AA development^[52]^. Positive association between body mass index and AA mortality has been found among Japanese men and smokers^[53]^. Our research shows that obesity is a smaller but significant risk factor for AA mortality particularly in regions with rising obesity rates, such as the Middle East and Eastern Europe, especially among women.

Our findings indicate that poor diet, including low fruit/vegetable intake and high sodium consumption, as well as lead exposure, also contribute to AA-related mortality. The underlying mechanism may be increased oxidative stress in aorta^[54,55]^. Their impact is more pronounced in specific countries, such as Zimbabwe and Mongolia for low fruit/vegetable intake, and China and the Republic of Korea for high salt consumption. Lead exposure, while minor globally (0.65%), is a significant concern in specific regions, including South and East Asia, Africa, and Central America. However, further longitudinal studies are needed for these emerging risk factors for AA.

### 4.5. Recommendations for reducing aortic aneurysm mortality

Taken together, our findings underscore the need for coordinated action between policymakers and healthcare professionals to strengthen tobacco control, improve hypertension screening and treatment, and address lifestyle and environmental risk factors for AA. Prevention strategies, particularly for women, should be reevaluated in light of rising sex-specific mortality. As population aging intensifies globally, healthcare systems must adapt to the growing burden of age-related diseases, including AA. In under-resourced regions such as Central and Eastern Europe and parts of Asia, targeted policies are needed to improve access to timely diagnosis and surgical care. Strengthening primary care, investing in workforce capacity, improving resource allocation, and ensuring universal health coverage are critical steps. Standardized mortality reporting frameworks are also essential to enhance surveillance and enable accurate international comparisons. These actions align with the UN Sustainable Development Goals, including SDG 3 (Good Health and Well-Being), SDG 5 (Gender Equality), and SDG 10 (Reduced Inequalities), supporting equitable and effective strategies to reduce AA mortality worldwide.

## 5. Limitations

The current study has several limitations. First, the data retrieved from the GBD 2021 study do not distinguish AAA from TAA, making it difficult to identify which regions are most affected by specific types of AA. Also, risk factors, as well as the underlying pathogenic mechanism differ between AAA and TAA, meaning that the attributable effects of modifiable risk factors presented here may be over- or underestimated for each type^[43,44,46]^. Thus, future global epidemiological studies should aim to separately quantify the burden of TAA and AAA to facilitate more precise public health strategies and resource allocation. Moreover, the GBD 2021 study does not include data on the prevalence and incidence of AA, which may impact the interpretation of the disease burden in this analysis.

The limitation for the estimation of risk-outcome association in GBD study has been summarized recently^[21]^. We relied on UIs provided by the GBD estimates, indeed highlighting the uncertainty of attributable risk factors with small mean contributions. UIs incorporate inherent uncertainty in the modeled exposure levels and relative risks. A hidden-bias sensitivity analysis was beyond the scope of this study due to the aggregated nature of available GBD data. Future studies employing individual-level or detailed registry-based data should incorporate such analyses to address hidden biases to these risk factors.

Our study may be influenced by socioeconomic factors, including disparities in healthcare access. Underreporting and limited data from certain regions, particularly in low- and middle-income societies, may affect the accuracy of our findings. In these settings, weak health infrastructure, incomplete vital registration, limited diagnostic tools and gaps in healthcare worker training may lead to undiagnosed, unreported or misclassified AA cases, thereby influencing the estimates^[56–58]^. In contrast, in society with advanced healthcare system, such as Japan^[34]^, widespread, easy, and affordable access to medical care, along with well-developed diagnostic and reporting system, may contribute to higher reported cases of AA-related mortality. These limitations should be considered when interpreting our findings.

## 6. Conclusion

Despite global efforts and decreasing trend of AA-related ASDR, the rising total number of AA-related deaths remains a significant burden, with notable regional variations and sex disparities. The decline of AA-related ASDR in Western Europe and the America suggests effective prevention and treatment strategies, while the increase in Eastern Europe, Central and South Asia, and Japan, particularly in women highlights emerging public health challenges. Smoking, hypertension, and obesity remain the top contributors to AA mortality, underscoring the need for targeted interventions, particularly in high-risk populations. Addressing these modifiable risk factors through public health campaigns, smoking cessation programs, and improved healthcare access could help reduce the global burden of AA-related deaths in the coming years.

## Acknowledgments

This research has been conducted as part of the Global Burden of Diseases, Injuries, and Risk Factors Study (GBD), coordinated by the Institute for Health Metrics and Evaluation. The GBD was partially funded by the Bill & Melinda Gates Foundation; the funders had no role in the study design, data analysis, data interpretation, or writing of the report.

## Ethics committee approval

This study was performed utilizing publicly available estimates provided by the Institute for Health Metrics and Evaluation (IHME) in the Global Burden of Disease (GBD) Study. The study did not involve direct interaction with human participants, and no sensitive individual-level data were collected or analyzed. Therefore, ethical approval and informed consent were not required for this research.

## Patient consent

Not required.

## Conflicts of interest

The authors declare that they have no conflicts of interest with regard to the content of this report.

## Funding source

This work was supported by grants from Karolinska Institute’s Research Foundations, Berth von Kantzows Foundation, Erik Mattssons Foundation, and Rolf Luft Foundation (to XZ); the Chengdu Science and Technology Program (2023-GH02-00083-HZ), the Sichuan Science and Technology Program (2025HJRC0028), The Ministry of Human Resources and Social Security (MOHRSS) of the People’s Republic of China foreign expert project (H20240709), and the Center of Excellence-International Collaboration Initiative Grant of West China Hospital (139220062; to XZ).

## Data availability statement

The data used in this study were sourced from the Global Burden of Disease (GBD) Study 2021, conducted by the Institute for Health Metrics and Evaluation (IHME). The data were accessed via the GBD Results tool, available at https://vizhub.healthdata.org/gbd-results/, accessed in March 2025. All raw data used to make the figures are presented in Supplemental Digital Content.

## Author contributions

XFZ and XWZ: Conceptualization. RMC, LM, DF, and XWZ: Methodology and investigation. RMC, LM, DF, XFZ, HMB, and XWZ: Validation. XFZ, HMB, and XWZ: Resources. RMC, LM, DF, XFZ, HMB, and XWZ: Writing, review & editing. RMC, LM, and XWZ: Visualization. All authors intellectually commented on and edited the manuscript and approved the final version.

## Supplementary Material
