# Supplementary material for: Global trends and risk factors of aortic aneurysm mortality from 1990 to 2021: An analysis of the Global Burden of Disease Study 2021
Source: Asian Heart J. 2025 Jun 30;1(1):18–37. doi: 10.1097/ah9.0000000000000008 (PMC12898288; doi:10.1097/ah9.0000000000000008)
Supplement: Supplementary file 1 [file ah9-1-18-s001.pdf]

Supplemental Digital Content: Raw data

Data are presented in Tables as mean with their 95% uncertainty intervals (UIs) with upper and lower bounds.

Data for Figure 1 ..... **4**
Fig 1A. Global death cases from 1990 to 2021 4
Fig 1B. World map of death cases in 2021 5
Fig 1C. Global number of deaths per age group in 2021 10
Fig 1D. Global death rates per 100,000 from 1990 to 2021 11
Fig 1E. World map of death rates per 100,000 in 2021 12
Fig 1F. Global death rate per 100,000 per age group in 2021 17
Fig 1G. Global age-standardized death rate (ASDR) from 1990 to 2021 18
Fig 1H and I. Global age-standardized death rate (ASDR) in 2021 19
Data for Figure 2 ..... **24**
Fig 2A. ASDR across Four World Regions from 1990 to 2021 24
Fig 2B. ASDR across Health System Groupings from 1990 to 2021 28
Fig 2C. ASDR across different Commonwealth Income regions from 1990 to 2021 32
Fig 2D. ASDR across SDI regions from 1990 to 2021 35
Fig 2E. ASDR in men and women across the Four World Regions in 2021 39
Fig 2F. ASDR in men and women across Health System Groupings in 2021 39
Fig 2G. ASDR in men and women across different Commonwealth Income regions in 2021 39
Fig 2H. ASDR in men and women across SDI regions in 2021 39
Data for Figure 3 ..... **41**
Fig 3A. ASDR in Europe from 1990 to 2021 41
Fig 3B. ASDR in Western Europe from 1990 to 2021 44
Fig 3C. ASDR in Central Europe from 1990 to 2021 63
Fig 3D. ASDR in Eastern Europe from 1990 to 2021 73
Fig 3E. ASDR in men and women from Europe in 2021 78
Fig 3F. ASDR in men and women from Western Europe in 2021 78
Fig 3G. ASDR in men and women from Central Europe 79
Fig 3H. ASDR in men and women from Eastern Europe in 2021 80
Data for Figure 4 ..... **81**
Fig 4A. ASDR in Asia from 1990 to 2021 81

Fig 4B. ASDR in South Asia from 1990 to 2021 87
Fig 4C. ASDR in Central Asia from 1990 to 2021 91
Fig 4D. ASDR in Southeast Asia from 1990 to 2021 98
Fig 4E. ASDR in East Asia from 1990 to 2021 108
Fig 4F. ASDR in men and women from Asia in 2021 110
Fig 4G. ASDR in men and women from South Asia in 2021 110
Fig 4H. ASDR in men and women from Central Asia in 2021 110
Fig 4I. ASDR in men and women from Southeast Asia in 2021 111
Fig 4J. ASDR in men and women from GBD East Asia in 2021 112
Data for Figure 5 .... **113**
Fig 5A. Global death number, death rate and ASDR in men and women and the difference between men and women in these metrics from 1990 to 2021 113
Fig 5B. Global percentage changes in death number, death rate and ASDR in men and women from 1990 to 2021 120
Fig 5C. Global death number and death rate per 100,000 in men and women and the difference between men and women in these metrics per age groups in 2021 121
Fig 5D. Percentage changes in Four World Regions in aortic aneurysm-caused ASDR of men and women in 2021 compared to 1990 124
Fig 5E. Percentage changes in USA and some European countries in aortic aneurysm-caused ASDR of men and women in 2021 compared to 1990 124
Fig 5F. Percentage changes in selected Asian countries in aortic aneurysm-caused ASDR of men and women in 2021 compared to 1990 124
Fig 5G. Percentage changes in top 10 countries with highest ASDR in aortic aneurysm-caused ASDR of men and women in 2021 compared to 1990 1255
Data for Figure 6 .... **126**
Fig 6A. Percentage contribution of risk factors to global AA-ASDR grouped by sex in 2021 126
Fig 6B. Percentage contribution of risk factors to AA-ASDR in world regions 127
Fig 6C. Percentage contribution of risk factors to AA-ASDR in world regions grouped by sex in 2021 130
Fig 6D. Percentage contribution of risk factors to AA-ASDR in high-burden countries in 2021 136
Fig 6E. World maps of percentage contribution of risk factors to AA-caused ASDR in 2021 139
Data for Figure 7 .... **173**

Fig 7A. Percentage contribution of risk factors to AA- ASDR in Europe in 2021 173
Fig 7B. Percentage contribution of risk factors to AA-ASDR in Europe grouped by sex in 2021 174
Fig 7C. AA-ASDR attributable to risk factors in Europe grouped by sex from 1990 to 2021.. 175
Data for Figure 8.. **179**
Fig 8A. Percentage contribution of risk factors to AA-ASDR in Asia in 2021 179
Fig 8B. Percentage contribution of risk factors to AA-ASDR in Asia grouped by sex in 2021.. 181
Fig 8C. AA-ASDR attributable to risk factors in Asia grouped by sex from 1990 to 2021... 184

Data for Figure 1

Fig 1A. Global death cases from 1990 to 2021

Year	Death cases
1990	88352.86 (83090.19 to 93491.94)
1991	91522.72 (85859.47 to 96845.37)
1992	94248.00 (88305.42 to 99617.79)
1993	98058.00 (91826.12 to 103627.38)
1994	101050.53 (94666.70 to 106801.81)
1995	103641.19 (96905.05 to 109549.50)
1996	105056.73 (98157.74 to 111170.91)
1997	105764.31 (98749.31 to 112013.17)
1998	107337.56 (100033.84 to 113578.21)
1999	110871.56 (103396.86 to 117443.96)
2000	111891.54 (104222.15 to 118437.53)
2001	111196.78 (103141.02 to 117903.18)
2002	112630.89 (104423.55 to 119485.70)
2003	114167.51 (105754.73 to 121059.80)
2004	114917.81 (106062.37 to 122119.45)
2005	116486.99 (107763.83 to 123827.27)
2006	117016.96 (107646.94 to 124342.87)
2007	118750.48 (109187.78 to 126633.60)
2008	119917.96 (109966.00 to 127769.06)
2009	120956.04 (111232.56 to 129205.74)
2010	122965.64 (112821.62 to 131022.72)
2011	124205.27 (113610.57 to 132604.13)
2012	125898.18 (115172.16 to 134509.96)
2013	128059.28 (116587.81 to 137271.82)
2014	131234.19 (119315.88 to 141021.07)
2015	134722.78 (122689.22 to 144516.99)
2016	139107.71 (125857.15 to 149208.95)
2017	141983.11 (128792.10 to 152396.51)
2018	145512.68 (131906.71 to 155795.32)
2019	149012.25 (134741.06 to 159764.58)
2020	150848.23 (135574.08 to 161878.18)
2021	153927.20 (138413.36 to 165738.65)

Fig 1B. World map of death cases in 2021

Location	Death cases
American Samoa	1.08 (0.90 to 1.31)
Antigua and Barbuda	1.85 (1.69 to 2.09)
Arab Republic of Egypt	250.50 (200.05 to 307.61)
Argentine Republic	1533.23 (1398.22 to 1665.05)
Australia	1186.44 (1032.66 to 1292.39)
Barbados	11.50 (9.33 to 14.14)
Belize	2.30 (2.00 to 2.60)
Bermuda	6.29 (5.36 to 7.54)
Bolivarian Republic of Venezuela	536.31 (419.48 to 670.89)
Bosnia and Herzegovina	189.32 (135.02 to 252.42)
Brunei Darussalam	12.88 (10.87 to 15.31)
Burkina Faso	155.28 (74.97 to 299.16)
Canada	1773.60 (1541.12 to 1928.66)
Central African Republic	37.08 (19.11 to 68.17)
Commonwealth of Dominica	3.08 (2.48 to 3.82)
Commonwealth of the Bahamas	10.31 (8.51 to 12.39)
Cook Islands	0.73 (0.42 to 1.24)
Czech Republic	637.49 (554.46 to 721.95)
Democratic People's Republic of Korea	174.70 (131.40 to 232.47)
Democratic Republic of Sao Tome and Principe	2.16 (1.07 to 3.79)
Democratic Republic of the Congo	626.42 (309.20 to 1114.51)
Democratic Republic of Timor-Leste	5.39 (3.56 to 8.27)
Democratic Socialist Republic of Sri Lanka	87.53 (59.23 to 122.43)
Dominican Republic	157.96 (115.35 to 208.30)
Eastern Republic of Uruguay	255.95 (233.21 to 275.61)
Federal Democratic Republic of Ethiopia	424.03 (218.94 to 719.77)
Federal Democratic Republic of Nepal	235.84 (162.42 to 367.86)
Federal Republic of Germany	4734.70 (4138.78 to 5130.86)
Federal Republic of Nigeria	1691.03 (768.67 to 3087.52)
Federal Republic of Somalia	43.90 (19.89 to 106.22)
Federated States of Micronesia	1.95 (1.40 to 2.61)
Federative Republic of Brazil	10010.25 (9185.86 to 10566.45)
French Republic	3144.53 (2713.06 to 3400.73)
Gabonese Republic	33.10 (20.12 to 47.97)
Georgia	173.00 (146.35 to 200.39)
Grand Duchy of Luxembourg	25.53 (22.49 to 28.28)
Greenland	1.07 (0.87 to 1.35)
Grenada	4.71 (4.11 to 5.29)
Guam	3.65 (3.08 to 4.27)
Hashemite Kingdom of Jordan	68.64 (52.50 to 88.67)

Hellenic Republic	1065.64 (955.05 to 1148.99)
Hungary	574.37 (507.39 to 647.34)
Independent State of Papua New Guinea	65.80 (43.83 to 96.42)
Independent State of Samoa	3.52 (2.66 to 4.55)
Ireland	247.58 (207.38 to 274.90)
Islamic Republic of Afghanistan	29.10 (18.46 to 43.66)
Islamic Republic of Iran	383.79 (340.23 to 432.35)
Islamic Republic of Mauritania	44.45 (16.20 to 78.07)
Islamic Republic of Pakistan	1472.16 (1080.82 to 2066.44)
Jamaica	55.71 (43.49 to 70.23)
Japan	23815.49 (19179.92 to 26463.45)
Kingdom of Bahrain	5.18 (3.91 to 7.01)
Kingdom of Belgium	606.22 (516.67 to 658.71)
Kingdom of Bhutan	8.29 (5.16 to 12.17)
Kingdom of Cambodia	73.62 (46.97 to 118.67)
Kingdom of Denmark	617.04 (547.43 to 672.54)
Kingdom of Eswatini	10.46 (6.84 to 14.86)
Kingdom of Lesotho	17.39 (9.21 to 27.46)
Kingdom of Morocco	139.56 (97.97 to 181.93)
Kingdom of Norway	540.60 (466.25 to 580.59)
Kingdom of Saudi Arabia	40.89 (28.58 to 54.80)
Kingdom of Spain	2255.90 (1978.97 to 2434.46)
Kingdom of Sweden	984.67 (845.59 to 1097.85)
Kingdom of Thailand	2133.88 (1640.10 to 2754.61)
Kingdom of the Netherlands	1465.59 (1262.87 to 1605.02)
Kingdom of Tonga	2.33 (1.66 to 3.05)
Kyrgyz Republic	36.04 (28.70 to 44.02)
Lao People's Democratic Republic	34.13 (24.87 to 46.86)
Lebanese Republic	139.61 (113.94 to 174.64)
Malaysia	971.33 (811.87 to 1158.65)
Mongolia	14.32 (10.89 to 18.32)
Montenegro	80.89 (61.25 to 105.23)
New Zealand	362.23 (317.81 to 392.13)
North Macedonia	79.51 (51.28 to 119.78)
Northern Mariana Islands	1.36 (1.07 to 1.70)
Palestine	17.39 (13.55 to 21.81)
People's Democratic Republic of Algeria	127.69 (95.98 to 169.99)
People's Republic of Bangladesh	1457.82 (952.13 to 2421.76)
People's Republic of China	9033.46 (7052.89 to 11643.53)
Plurinational State of Bolivia	111.14 (83.88 to 149.99)
Portuguese Republic	404.98 (361.46 to 436.78)
Principality of Andorra	6.44 (4.09 to 9.69)

Principality of Monaco	5.85 (4.42 to 7.84)
Puerto Rico	77.01 (63.32 to 90.36)
Republic of Albania	46.83 (32.41 to 65.26)
Republic of Angola	272.60 (162.44 to 408.84)
Republic of Armenia	396.67 (330.04 to 467.31)
Republic of Austria	364.71 (321.35 to 394.95)
Republic of Azerbaijan	130.31 (81.38 to 211.56)
Republic of Belarus	595.76 (485.73 to 714.72)
Republic of Benin	70.18 (27.97 to 125.91)
Republic of Botswana	25.48 (15.55 to 35.03)
Republic of Bulgaria	362.88 (294.50 to 448.66)
Republic of Burundi	57.45 (25.50 to 105.88)
Republic of Cabo Verde	10.21 (4.96 to 17.83)
Republic of Cameroon	246.56 (146.45 to 429.80)
Republic of Chad	72.50 (32.53 to 142.95)
Republic of Chile	563.14 (514.26 to 603.97)
Republic of Colombia	1635.74 (1357.03 to 1937.00)
Republic of Costa Rica	131.80 (114.59 to 148.00)
Republic of Côte d'Ivoire	225.61 (100.21 to 392.95)
Republic of Croatia	316.78 (271.04 to 364.68)
Republic of Cuba	770.49 (655.31 to 868.02)
Republic of Cyprus	80.41 (60.20 to 101.42)
Republic of Djibouti	9.74 (4.15 to 15.81)
Republic of Ecuador	207.72 (164.69 to 262.06)
Republic of El Salvador	33.78 (26.66 to 43.15)
Republic of Equatorial Guinea	14.57 (6.84 to 25.64)
Republic of Estonia	97.61 (85.07 to 110.32)
Republic of Fiji	21.40 (16.02 to 27.44)
Republic of Finland	519.49 (449.99 to 566.43)
Republic of Ghana	403.84 (184.09 to 682.71)
Republic of Guatemala	44.55 (38.06 to 52.15)
Republic of Guinea	93.21 (34.23 to 180.58)
Republic of Guinea-Bissau	13.36 (7.22 to 23.88)
Republic of Guyana	19.82 (15.30 to 25.13)
Republic of Haiti	131.10 (81.08 to 204.92)
Republic of Honduras	66.40 (48.19 to 89.77)
Republic of Iceland	16.74 (14.12 to 18.73)
Republic of India	12805.03 (9106.77 to 18763.13)
Republic of Indonesia	1819.52 (1246.16 to 2445.73)
Republic of Iraq	88.75 (64.87 to 117.52)
Republic of Italy	3654.92 (3186.88 to 3938.73)
Republic of Kazakhstan	376.69 (302.21 to 461.90)

Republic of Kenya	336.58 (198.11 to 468.32)
Republic of Kiribati	0.28 (0.20 to 0.36)
Republic of Korea	1767.00 (1440.81 to 2092.51)
Republic of Latvia	124.94 (107.23 to 141.58)
Republic of Liberia	33.77 (14.92 to 66.01)
Republic of Lithuania	186.51 (162.26 to 210.89)
Republic of Madagascar	243.96 (118.60 to 402.66)
Republic of Malawi	122.48 (59.16 to 208.90)
Republic of Maldives	1.79 (0.81 to 3.03)
Republic of Mali	87.73 (38.44 to 168.99)
Republic of Malta	14.03 (12.32 to 15.72)
Republic of Mauritius	14.88 (13.60 to 15.78)
Republic of Moldova	88.90 (78.62 to 99.68)
Republic of Mozambique	232.76 (94.07 to 453.84)
Republic of Namibia	27.48 (19.85 to 37.90)
Republic of Nauru	0.24 (0.17 to 0.32)
Republic of Nicaragua	15.82 (12.73 to 19.42)
Republic of Niue	0.07 (0.05 to 0.08)
Republic of Palau	0.49 (0.38 to 0.65)
Republic of Panama	78.60 (60.13 to 95.56)
Republic of Paraguay	162.90 (125.81 to 206.75)
Republic of Peru	219.23 (163.28 to 289.57)
Republic of Poland	2575.97 (2306.76 to 2830.65)
Republic of Rwanda	95.95 (56.15 to 156.76)
Republic of San Marino	1.46 (0.95 to 2.27)
Republic of Senegal	142.59 (60.36 to 263.44)
Republic of Serbia	691.68 (548.90 to 871.74)
Republic of Seychelles	1.08 (0.74 to 1.52)
Republic of Sierra Leone	62.72 (27.61 to 121.49)
Republic of Singapore	177.36 (157.51 to 191.33)
Republic of Slovenia	105.28 (88.59 to 126.29)
Republic of South Africa	935.35 (819.86 to 1052.21)
Republic of South Sudan	44.88 (19.54 to 89.43)
Republic of Sudan	66.77 (41.21 to 97.68)
Republic of Suriname	12.11 (8.48 to 16.72)
Republic of Tajikistan	12.75 (9.10 to 17.32)
Republic of the Congo	73.53 (43.54 to 110.81)
Republic of the Gambia	21.22 (9.90 to 36.82)
Republic of the Marshall Islands	0.77 (0.50 to 1.11)
Republic of the Niger	75.55 (28.59 to 175.18)
Republic of the Philippines	950.08 (779.01 to 1119.23)
Republic of the Union of Myanmar	392.21 (296.46 to 513.63)

Republic of Trinidad and Tobago	74.21 (57.40 to 93.69)
Republic of Tunisia	55.57 (35.84 to 80.82)
Republic of Turkey	2075.39 (1640.49 to 2605.72)
Republic of Uganda	204.85 (99.15 to 337.63)
Republic of Uzbekistan	231.37 (186.39 to 284.82)
Republic of Vanuatu	3.08 (2.28 to 4.16)
Republic of Yemen	45.53 (26.24 to 71.28)
Republic of Zambia	204.57 (87.46 to 371.74)
Republic of Zimbabwe	220.59 (164.47 to 297.48)
Romania	705.79 (613.50 to 810.36)
Russian Federation	10444.90 (9555.03 to 11307.25)
Saint Kitts and Nevis	1.37 (1.12 to 1.67)
Saint Lucia	11.40 (9.59 to 13.30)
Saint Vincent and the Grenadines	2.78 (2.46 to 3.15)
Slovak Republic	217.54 (170.03 to 275.07)
Socialist Republic of Viet Nam	894.89 (656.44 to 1192.65)
Solomon Islands	4.97 (3.37 to 7.18)
State of Eritrea	42.47 (19.38 to 81.65)
State of Israel	171.17 (150.01 to 186.67)
State of Kuwait	24.22 (20.03 to 29.77)
State of Libya	18.10 (10.87 to 28.95)
State of Qatar	8.11 (4.86 to 12.96)
Sultanate of Oman	11.30 (6.51 to 19.20)
Swiss Confederation	487.50 (411.15 to 539.94)
Syrian Arab Republic	50.96 (36.72 to 68.44)
Taiwan (Province of China)	990.85 (888.32 to 1071.28)
Togolese Republic	70.95 (29.93 to 126.48)
Tokelau	0.05 (0.03 to 0.07)
Turkmenistan	71.57 (53.10 to 102.83)
Tuvalu	0.26 (0.21 to 0.33)
Ukraine	1867.34 (1384.72 to 2442.11)
Union of the Comoros	8.42 (3.15 to 15.34)
United Arab Emirates	43.16 (34.04 to 54.00)
United Kingdom of Great Britain and Northern Ireland	6071.17 (5374.04 to 6434.62)
United Mexican States	749.75 (662.46 to 845.06)
United Republic of Tanzania	561.82 (271.21 to 957.26)
United States of America	12194.98 (10939.91 to 12943.79)
United States Virgin Islands	3.62 (2.75 to 4.58)

Fig 1C. Global number of deaths per age group in 2021

Age	Death cases
15-19 years	204.04 (172.69 to 238.52)
20-24 years	372.37 (324.39 to 427.38)
25-29 years	589.06 (520.56 to 666.70)
30-34 years	1015.38 (911.52 to 1150.28)
35-39 years	1663.93 (1484.60 to 1893.23)
40-44 years	2499.70 (2253.07 to 2793.16)
45-49 years	3727.72 (3421.01 to 4117.56)
50-54 years	5404.79 (4983.78 to 5935.50)
55-59 years	8290.38 (7597.86 to 9113.56)
60-64 years	11669.14 (10885.96 to 12673.19)
65-69 years	16484.09 (15355.40 to 17909.55)
70-74 years	21017.46 (19538.19 to 22682.30)
75-79 years	21508.22 (19620.13 to 23035.32)
80-84 years	22556.80 (19647.35 to 24401.09)
85-89 years	19701.47 (16222.10 to 21637.05)
90-94 years	12188.40 (9386.06 to 13647.46)
95+ years	5034.26 (3520.79 to 5857.42)

Fig 1D. Global death rates per 100,000 from 1990 to 2021

Year	Death rate per 100,000
1990	1.66 (1.56 to 1.75)
1991	1.69 (1.59 to 1.79)
1992	1.71 (1.61 to 1.81)
1993	1.76 (1.65 to 1.86)
1994	1.79 (1.68 to 1.89)
1995	1.81 (1.69 to 1.91)
1996	1.81 (1.69 to 1.92)
1997	1.80 (1.68 to 1.91)
1998	1.80 (1.68 to 1.91)
1999	1.84 (1.72 to 1.95)
2000	1.83 (1.71 to 1.94)
2001	1.80 (1.67 to 1.91)
2002	1.80 (1.67 to 1.91)
2003	1.80 (1.67 to 1.91)
2004	1.79 (1.65 to 1.90)
2005	1.79 (1.66 to 1.90)
2006	1.78 (1.63 to 1.89)
2007	1.78 (1.64 to 1.90)
2008	1.77 (1.62 to 1.89)
2009	1.76 (1.62 to 1.88)
2010	1.77 (1.62 to 1.89)
2011	1.76 (1.61 to 1.88)
2012	1.77 (1.62 to 1.89)
2013	1.77 (1.62 to 1.90)
2014	1.80 (1.63 to 1.93)
2015	1.82 (1.66 to 1.95)
2016	1.86 (1.68 to 1.99)
2017	1.87 (1.70 to 2.01)
2018	1.90 (1.72 to 2.03)
2019	1.92 (1.74 to 2.06)
2020	1.93 (1.73 to 2.07)
2021	1.95 (1.75 to 2.10)

Fig 1E. World map of death rates per 100,000 in 2021

Location	Death rate per 100,000
China	0.63 (0.50 to 0.82)
Tajikistan	0.13 (0.09 to 0.17)
Cabo Verde	1.83 (0.89 to 3.19)
Monaco	15.46 (11.68 to 20.70)
Fiji	2.32 (1.73 to 2.97)
Kiribati	0.23 (0.16 to 0.30)
New Zealand	7.01 (6.15 to 7.59)
Democratic People's Republic of Korea	0.66 (0.50 to 0.88)
Syrian Arab Republic	0.36 (0.26 to 0.49)
Seychelles	1.03 (0.70 to 1.44)
Malta	3.17 (2.78 to 3.55)
Bolivia (Plurinational State of)	0.94 (0.71 to 1.27)
Slovenia	5.09 (4.28 to 6.10)
Antigua and Barbuda	2.07 (1.89 to 2.34)
Congo	1.36 (0.81 to 2.06)
Chad	0.41 (0.18 to 0.81)
Turkmenistan	1.39 (1.03 to 1.99)
Nauru	2.21 (1.58 to 2.94)
Democratic Republic of the Congo	0.70 (0.34 to 1.24)
Algeria	0.29 (0.22 to 0.38)
Bahamas	2.66 (2.19 to 3.19)
Marshall Islands	1.37 (0.90 to 1.97)
Uzbekistan	0.68 (0.54 to 0.83)
Netherlands	8.52 (7.34 to 9.33)
Belarus	6.39 (5.21 to 7.66)
United Republic of Tanzania	0.96 (0.46 to 1.64)
Norway	9.98 (8.61 to 10.72)
Equatorial Guinea	0.96 (0.45 to 1.70)
Tunisia	0.47 (0.30 to 0.68)
Gambia	0.89 (0.41 to 1.54)
Taiwan (Province of China)	4.19 (3.76 to 4.53)
Peru	0.60 (0.45 to 0.80)
Somalia	0.20 (0.09 to 0.49)
Côte d'Ivoire	0.81 (0.36 to 1.41)
Micronesia (Federated States of)	1.90 (1.37 to 2.54)
Niue	3.95 (3.10 to 4.81)
Estonia	7.45 (6.49 to 8.42)
Uganda	0.47 (0.23 to 0.78)
United Arab Emirates	0.45 (0.35 to 0.56)
Portugal	3.82 (3.41 to 4.12)
Albania	1.76 (1.21 to 2.45)
Ecuador	1.15 (0.91 to 1.45)

Türkiye	2.48 (1.96 to 3.12)
Papua New Guinea	0.63 (0.42 to 0.92)
Cambodia	0.43 (0.28 to 0.70)
Andorra	7.53 (4.78 to 11.32)
Belgium	5.29 (4.50 to 5.74)
Yemen	0.14 (0.08 to 0.21)
Guinea	0.69 (0.25 to 1.34)
Northern Mariana Islands	2.80 (2.20 to 3.50)
Spain	4.95 (4.34 to 5.34)
Gabon	1.82 (1.11 to 2.64)
Bahrain	0.34 (0.26 to 0.46)
Samoa	1.65 (1.25 to 2.13)
Barbados	3.85 (3.12 to 4.73)
Ghana	1.18 (0.54 to 1.99)
Colombia	3.33 (2.77 to 3.95)
Indonesia	0.65 (0.45 to 0.88)
Burundi	0.43 (0.19 to 0.80)
Sweden	9.49 (8.15 to 10.58)
Cyprus	5.92 (4.43 to 7.47)
Solomon Islands	0.73 (0.49 to 1.05)
Bosnia and Herzegovina	5.73 (4.09 to 7.64)
Dominica	4.59 (3.70 to 5.69)
Palau	2.72 (2.09 to 3.60)
Belize	0.54 (0.47 to 0.61)
Switzerland	5.46 (4.61 to 6.05)
Austria	4.06 (3.58 to 4.40)
Zambia	1.05 (0.45 to 1.90)
Guinea-Bissau	0.65 (0.35 to 1.16)
Puerto Rico	2.34 (1.92 to 2.74)
Saint Kitts and Nevis	2.33 (1.91 to 2.85)
Bulgaria	5.35 (4.34 to 6.61)
Cuba	6.84 (5.81 to 7.70)
Egypt	0.24 (0.19 to 0.29)
Comoros	1.13 (0.42 to 2.06)
Latvia	6.68 (5.73 to 7.57)
Lao People's Democratic Republic	0.46 (0.34 to 0.64)
Croatia	7.53 (6.44 to 8.67)
Botswana	1.06 (0.65 to 1.46)
Jordan	0.56 (0.43 to 0.72)
Republic of Moldova	2.47 (2.19 to 2.77)
Ukraine	4.33 (3.21 to 5.67)
Tonga	2.19 (1.56 to 2.87)
Iran (Islamic Republic of)	0.45 (0.40 to 0.51)
Lithuania	6.84 (5.95 to 7.73)

Dominican Republic	1.43 (1.05 to 1.89)
Russian Federation	7.21 (6.60 to 7.81)
Liberia	0.62 (0.27 to 1.21)
Lesotho	0.93 (0.49 to 1.47)
United Kingdom	8.95 (7.92 to 9.48)
Iraq	0.22 (0.16 to 0.29)
Costa Rica	2.78 (2.41 to 3.12)
Sudan	0.15 (0.09 to 0.22)
Czechia	6.00 (5.21 to 6.79)
Bangladesh	0.89 (0.58 to 1.47)
Djibouti	0.77 (0.33 to 1.26)
Malaysia	3.05 (2.55 to 3.64)
Honduras	0.66 (0.48 to 0.89)
Vanuatu	0.99 (0.73 to 1.33)
El Salvador	0.52 (0.41 to 0.67)
Maldives	0.35 (0.16 to 0.59)
Eritrea	0.64 (0.29 to 1.24)
Tokelau	3.71 (2.40 to 5.21)
Kuwait	0.52 (0.43 to 0.64)
San Marino	4.47 (2.91 to 6.94)
Bhutan	1.09 (0.68 to 1.61)
Haiti	1.02 (0.63 to 1.59)
Mauritania	1.01 (0.37 to 1.78)
Chile	3.00 (2.74 to 3.21)
South Africa	1.65 (1.44 to 1.85)
Argentina	3.37 (3.07 to 3.66)
Armenia	13.24 (11.02 to 15.60)
Greece	10.47 (9.39 to 11.29)
Kenya	0.67 (0.40 to 0.94)
Hungary	5.98 (5.29 to 6.74)
Afghanistan	0.09 (0.06 to 0.14)
Mexico	0.58 (0.51 to 0.65)
Guatemala	0.28 (0.24 to 0.33)
Mali	0.36 (0.16 to 0.70)
Finland	9.38 (8.13 to 10.23)
Jamaica	1.99 (1.55 to 2.51)
Niger	0.30 (0.11 to 0.70)
Lebanon	2.52 (2.06 to 3.15)
France	4.74 (4.09 to 5.12)
Myanmar	0.70 (0.53 to 0.91)
United States Virgin Islands	4.21 (3.20 to 5.33)
Guyana	2.59 (2.00 to 3.29)
Ethiopia	0.39 (0.20 to 0.66)
Brunei Darussalam	2.86 (2.41 to 3.39)

Nigeria	0.73 (0.33 to 1.34)
Bermuda	9.90 (8.44 to 11.87)
Azerbaijan	1.24 (0.77 to 2.01)
Germany	5.55 (4.85 to 6.01)
Morocco	0.38 (0.26 to 0.49)
Japan	18.65 (15.02 to 20.72)
Namibia	1.13 (0.82 to 1.56)
Greenland	1.91 (1.54 to 2.41)
Philippines	0.84 (0.69 to 0.99)
Eswatini	0.91 (0.59 to 1.29)
Denmark	10.54 (9.35 to 11.49)
American Samoa	2.18 (1.81 to 2.64)
Uruguay	7.52 (6.85 to 8.09)
Republic of Korea	3.43 (2.79 to 4.06)
India	0.91 (0.64 to 1.33)
Grenada	4.59 (4.01 to 5.16)
Cook Islands	4.12 (2.37 to 6.99)
Tuvalu	2.10 (1.67 to 2.64)
Saint Lucia	6.42 (5.40 to 7.49)
Palestine	0.34 (0.26 to 0.42)
Nicaragua	0.24 (0.19 to 0.29)
Madagascar	0.85 (0.42 to 1.41)
Panama	1.83 (1.40 to 2.23)
Thailand	3.20 (2.46 to 4.13)
Montenegro	13.09 (9.91 to 17.03)
Kyrgyzstan	0.53 (0.42 to 0.64)
Timor-Leste	0.39 (0.25 to 0.59)
Sri Lanka	0.39 (0.27 to 0.55)
Pakistan	0.62 (0.46 to 0.88)
Romania	3.73 (3.24 to 4.28)
Venezuela (Bolivarian Republic of)	2.01 (1.58 to 2.52)
Kazakhstan	1.99 (1.59 to 2.44)
Mauritius	1.17 (1.07 to 1.24)
Libya	0.26 (0.16 to 0.42)
Canada	4.73 (4.11 to 5.15)
Malawi	0.63 (0.30 to 1.07)
Oman	0.24 (0.14 to 0.41)
Senegal	0.90 (0.38 to 1.66)
Guam	2.29 (1.93 to 2.68)
Italy	6.11 (5.33 to 6.59)
Trinidad and Tobago	5.33 (4.12 to 6.73)
North Macedonia	3.65 (2.36 to 5.50)
Mozambique	0.75 (0.30 to 1.46)
Togo	0.85 (0.36 to 1.51)

Mongolia	0.43 (0.33 to 0.55)
Sao Tome and Principe	1.00 (0.50 to 1.75)
Australia	4.60 (4.00 to 5.01)
Suriname	2.09 (1.46 to 2.89)
Viet Nam	0.89 (0.65 to 1.19)
Israel	1.78 (1.56 to 1.95)
Ireland	5.01 (4.20 to 5.56)
Poland	6.74 (6.03 to 7.40)
Zimbabwe	1.41 (1.05 to 1.91)
Sierra Leone	0.71 (0.31 to 1.37)
Nepal	0.76 (0.52 to 1.18)
United States of America	3.67 (3.29 to 3.89)
Qatar	0.27 (0.16 to 0.44)
Paraguay	2.27 (1.75 to 2.88)
Rwanda	0.72 (0.42 to 1.18)
Luxembourg	3.96 (3.49 to 4.39)
Saint Vincent and the Grenadines	2.44 (2.16 to 2.76)
Georgia	4.80 (4.06 to 5.56)
Saudi Arabia	0.11 (0.08 to 0.15)
Benin	0.52 (0.21 to 0.93)
Burkina Faso	0.68 (0.33 to 1.31)
South Sudan	0.46 (0.20 to 0.92)
Singapore	3.10 (2.75 to 3.34)
Brazil	4.54 (4.17 to 4.80)
Cameroon	0.78 (0.46 to 1.35)
Iceland	4.78 (4.03 to 5.35)
Serbia	7.75 (6.15 to 9.77)
Angola	0.83 (0.50 to 1.25)
Slovakia	4.01 (3.13 to 5.07)
Central African Republic	0.68 (0.35 to 1.24)

Fig 1F. Global death rate per 100,000 per age group in 2021

Age	Death rate per 100,000
15-19 years	0.03 (0.03 to 0.04)
20-24 years	0.06 (0.05 to 0.07)
25-29 years	0.10 (0.09 to 0.11)
30-34 years	0.17 (0.15 to 0.19)
35-39 years	0.30 (0.26 to 0.34)
40-44 years	0.50 (0.45 to 0.56)
45-49 years	0.79 (0.72 to 0.87)
50-54 years	1.21 (1.12 to 1.33)
55-59 years	2.09 (1.92 to 2.30)
60-64 years	3.65 (3.40 to 3.96)
65-69 years	5.98 (5.57 to 6.49)
70-74 years	10.21 (9.49 to 11.02)
75-79 years	16.31 (14.88 to 17.47)
80-84 years	25.75 (22.43 to 27.86)
85-89 years	43.09 (35.48 to 47.32)
90-94 years	68.13 (52.47 to 76.29)
95+ years	92.37 (64.60 to 107.47)

Fig 1G. Global age-standardized death rate (ASDR) from 1990 to 2021

Year	ASDR per 100,000
1990	2.54 (2.35 to 2.69)
1992	2.57 (2.37 to 2.72)
1991	2.56 (2.37 to 2.72)
1994	2.61 (2.42 to 2.77)
1997	2.55 (2.35 to 2.70)
1995	2.62 (2.42 to 2.77)
1998	2.53 (2.33 to 2.68)
2000	2.50 (2.31 to 2.65)
2003	2.36 (2.16 to 2.51)
1999	2.54 (2.35 to 2.70)
2001	2.42 (2.22 to 2.57)
2004	2.31 (2.11 to 2.46)
2006	2.22 (2.02 to 2.36)
2008	2.14 (1.94 to 2.28)
2005	2.28 (2.08 to 2.43)
2010	2.07 (1.88 to 2.20)
2007	2.19 (1.99 to 2.33)
2009	2.10 (1.91 to 2.24)
2011	2.02 (1.84 to 2.16)
2012	1.99 (1.80 to 2.13)
2013	1.96 (1.78 to 2.11)
2015	1.94 (1.76 to 2.09)
2014	1.95 (1.76 to 2.10)
2016	1.95 (1.75 to 2.08)
2017	1.92 (1.74 to 2.07)
2018	1.91 (1.72 to 2.05)
2019	1.90 (1.71 to 2.04)
1996	2.59 (2.40 to 2.74)
2021	1.86 (1.67 to 2.00)
2002	2.39 (2.19 to 2.54)
1993	2.60 (2.40 to 2.75)
2020	1.87 (1.67 to 2.00)

Fig 1H and I. Global age-standardized death rate (ASDR) in 2021

Location	ASDR per 100,000
Republic of Armenia	9.16 (7.61 to 10.81)
Montenegro	8.65 (6.59 to 11.28)
Republic of Nauru	6.01 (3.50 to 9.06)
Principality of Monaco	5.15 (3.88 to 6.95)
Japan	5.07 (4.33 to 5.47)
Saint Lucia	4.95 (4.17 to 5.77)
Brunei Darussalam	4.79 (4.00 to 5.67)
Kingdom of Norway	4.75 (4.15 to 5.08)
Kingdom of Denmark	4.61 (4.10 to 5.02)
Grenada	4.51 (3.94 to 5.04)
Russian Federation	4.38 (4.01 to 4.74)
Eastern Republic of Uruguay	4.35 (3.98 to 4.67)
Bermuda	4.15 (3.55 to 4.99)
Federative Republic of Brazil	4.06 (3.72 to 4.29)
Republic of Serbia	4.05 (3.21 to 5.10)
Malaysia	4.04 (3.38 to 4.88)
United Kingdom of Great Britain and Northern Ireland	4.03 (3.61 to 4.25)
Hellenic Republic	3.99 (3.65 to 4.26)
New Zealand	3.99 (3.52 to 4.30)
Republic of Cyprus	3.96 (3.04 to 4.88)
Principality of Andorra	3.96 (2.50 to 5.97)
Republic of Zimbabwe	3.96 (3.06 to 5.20)
Commonwealth of Dominica	3.94 (3.17 to 4.87)
Republic of Trinidad and Tobago	3.94 (3.05 to 4.96)
Kingdom of Sweden	3.88 (3.38 to 4.33)
Gabonese Republic	3.79 (2.35 to 5.45)
Republic of Belarus	3.75 (3.05 to 4.49)
Republic of Cuba	3.74 (3.18 to 4.22)
Kingdom of the Netherlands	3.74 (3.24 to 4.08)
Republic of Fiji	3.65 (2.86 to 4.58)
Republic of Finland	3.63 (3.20 to 3.93)
Federated States of Micronesia	3.51 (2.73 to 4.51)
Republic of Guyana	3.45 (2.70 to 4.33)
Republic of Poland	3.44 (3.09 to 3.79)
Tokelau	3.41 (2.24 to 4.78)
Republic of Equatorial Guinea	3.39 (1.69 to 5.75)
Republic of the Congo	3.38 (2.01 to 5.05)
Northern Mariana Islands	3.38 (2.76 to 4.11)
Republic of Zambia	3.36 (1.53 to 5.94)
Republic of Croatia	3.34 (2.87 to 3.86)
Republic of Estonia	3.34 (2.90 to 3.78)
Republic of Niue	3.27 (2.58 to 3.98)

Republic of Lithuania	3.15 (2.73 to 3.56)
Republic of Latvia	3.05 (2.63 to 3.47)
Kingdom of Tonga	3.04 (2.18 to 3.99)
Tuvalu	3.02 (2.44 to 3.75)
Bosnia and Herzegovina	3.00 (2.15 to 3.99)
Republic of Colombia	2.97 (2.47 to 3.53)
Cook Islands	2.94 (1.70 to 4.97)
Republic of Palau	2.93 (2.24 to 3.79)
Ireland	2.93 (2.47 to 3.25)
Republic of the Marshall Islands	2.92 (2.03 to 3.99)
Republic of Paraguay	2.90 (2.23 to 3.67)
Republic of Angola	2.89 (1.72 to 4.26)
Georgia	2.88 (2.43 to 3.35)
Hungary	2.87 (2.54 to 3.23)
Republic of Ghana	2.87 (1.33 to 4.74)
Commonwealth of the Bahamas	2.85 (2.37 to 3.40)
Czech Republic	2.83 (2.46 to 3.19)
Independent State of Samoa	2.80 (2.12 to 3.57)
American Samoa	2.72 (2.24 to 3.30)
Argentine Republic	2.67 (2.44 to 2.90)
North Macedonia	2.63 (1.80 to 3.81)
Republic of Bulgaria	2.62 (2.12 to 3.23)
Republic of Iceland	2.61 (2.23 to 2.92)
Republic of Madagascar	2.53 (1.19 to 4.29)
United Republic of Tanzania	2.50 (1.21 to 4.29)
Republic of Namibia	2.48 (1.85 to 3.39)
Ukraine	2.47 (1.82 to 3.25)
Republic of Cabo Verde	2.46 (1.19 to 4.30)
Republic of the Gambia	2.44 (1.12 to 4.26)
Republic of Côte d'Ivoire	2.44 (1.10 to 4.31)
Saint Kitts and Nevis	2.44 (2.04 to 2.90)
Republic of Costa Rica	2.41 (2.10 to 2.70)
Republic of Mozambique	2.40 (1.03 to 4.55)
Taiwan (Province of China)	2.37 (2.14 to 2.56)
Islamic Republic of Mauritania	2.37 (0.85 to 4.15)
Australia	2.35 (2.06 to 2.55)
Republic of Cameroon	2.33 (1.42 to 3.97)
Federal Republic of Nigeria	2.30 (1.09 to 4.12)
Democratic Republic of Sao Tome and Principe	2.29 (1.14 to 4.00)
Republic of Haiti	2.29 (1.42 to 3.57)
Togolese Republic	2.29 (0.96 to 4.18)
Slovak Republic	2.29 (1.79 to 2.90)
Republic of South Africa	2.29 (2.02 to 2.54)
Swiss Confederation	2.28 (1.96 to 2.51)

Republic of Turkey	2.27 (1.80 to 2.83)
Canada	2.26 (1.99 to 2.45)
Kingdom of Belgium	2.25 (1.96 to 2.43)
Federal Republic of Germany	2.25 (2.02 to 2.41)
Republic of Guinea-Bissau	2.24 (1.18 to 3.99)
Republic of Vanuatu	2.23 (1.65 to 2.95)
Republic of Kazakhstan	2.23 (1.80 to 2.71)
Grand Duchy of Luxembourg	2.23 (1.97 to 2.46)
Republic of Botswana	2.22 (1.36 to 3.04)
Kingdom of Eswatini	2.21 (1.47 to 3.03)
Lebanese Republic	2.21 (1.80 to 2.76)
Barbados	2.18 (1.77 to 2.68)
Republic of Italy	2.18 (1.95 to 2.34)
Republic of Chile	2.17 (1.98 to 2.32)
Republic of Slovenia	2.16 (1.82 to 2.58)
Republic of Singapore	2.15 (1.91 to 2.32)
Central African Republic	2.14 (1.08 to 3.73)
Democratic Republic of the Congo	2.14 (1.08 to 3.82)
Republic of Senegal	2.12 (0.90 to 3.88)
Saint Vincent and the Grenadines	2.09 (1.85 to 2.35)
Kingdom of Spain	2.08 (1.87 to 2.23)
United States of America	2.06 (1.87 to 2.18)
United States Virgin Islands	2.02 (1.56 to 2.57)
Burkina Faso	2.02 (0.98 to 3.85)
Republic of Suriname	2.02 (1.41 to 2.79)
Kingdom of Thailand	2.01 (1.55 to 2.58)
Union of the Comoros	1.97 (0.74 to 3.60)
Solomon Islands	1.97 (1.35 to 2.84)
Kingdom of Lesotho	1.96 (1.14 to 2.97)
Republic of Liberia	1.95 (0.86 to 3.70)
Republic of Djibouti	1.94 (0.86 to 3.09)
Antigua and Barbuda	1.92 (1.75 to 2.15)
Republic of Korea	1.92 (1.56 to 2.27)
Bolivarian Republic of Venezuela	1.91 (1.50 to 2.37)
State of Eritrea	1.90 (0.84 to 3.58)
Romania	1.90 (1.65 to 2.18)
French Republic	1.89 (1.67 to 2.04)
Republic of Sierra Leone	1.89 (0.82 to 3.62)
Republic of Guinea	1.89 (0.70 to 3.66)
Turkmenistan	1.88 (1.42 to 2.67)
Republic of Malawi	1.85 (0.93 to 3.18)
Republic of Rwanda	1.84 (1.12 to 3.04)
Greenland	1.84 (1.49 to 2.30)
Republic of Austria	1.80 (1.61 to 1.94)

Republic of Kenya	1.78 (1.05 to 2.49)
Guam	1.78 (1.51 to 2.07)
Republic of Panama	1.77 (1.35 to 2.15)
Jamaica	1.75 (1.36 to 2.21)
Republic of San Marino	1.67 (1.06 to 2.59)
Dominican Republic	1.63 (1.19 to 2.15)
Republic of Uganda	1.60 (0.80 to 2.64)
Republic of Benin	1.60 (0.65 to 2.86)
Independent State of Papua New Guinea	1.58 (1.07 to 2.25)
Portuguese Republic	1.51 (1.37 to 1.62)
Republic of Chad	1.50 (0.66 to 2.99)
Republic of Moldova	1.49 (1.32 to 1.67)
Kingdom of Bhutan	1.49 (0.93 to 2.16)
Islamic Republic of Pakistan	1.48 (1.06 to 2.04)
United Arab Emirates	1.45 (1.17 to 1.84)
Republic of Burundi	1.44 (0.62 to 2.67)
Republic of South Sudan	1.39 (0.62 to 2.68)
Republic of Azerbaijan	1.37 (0.93 to 2.08)
Plurinational State of Bolivia	1.36 (1.03 to 1.84)
Republic of the Philippines	1.35 (1.12 to 1.58)
Republic of Ecuador	1.32 (1.06 to 1.66)
Republic of Malta	1.32 (1.17 to 1.47)
State of Israel	1.28 (1.13 to 1.40)
People's Republic of Bangladesh	1.21 (0.80 to 1.99)
Republic of India	1.20 (0.86 to 1.74)
Republic of Honduras	1.19 (0.87 to 1.60)
Federal Democratic Republic of Nepal	1.19 (0.82 to 1.85)
Republic of Mali	1.18 (0.51 to 2.27)
Republic of the Niger	1.17 (0.45 to 2.69)
Federal Democratic Republic of Ethiopia	1.10 (0.57 to 1.87)
Republic of Albania	1.09 (0.76 to 1.50)
Socialist Republic of Viet Nam	1.07 (0.78 to 1.42)
Republic of Seychelles	1.07 (0.74 to 1.51)
Republic of Uzbekistan	1.04 (0.85 to 1.28)
Republic of Indonesia	1.03 (0.71 to 1.36)
Hashemite Kingdom of Jordan	0.97 (0.76 to 1.24)
Republic of the Union of Myanmar	0.96 (0.73 to 1.26)
State of Qatar	0.94 (0.59 to 1.46)
Lao People's Democratic Republic	0.93 (0.68 to 1.28)
Puerto Rico	0.93 (0.77 to 1.08)
Republic of Mauritius	0.88 (0.81 to 0.94)
Belize	0.88 (0.76 to 0.99)
Federal Republic of Somalia	0.86 (0.41 to 2.05)
State of Kuwait	0.86 (0.71 to 1.06)

Kyrgyz Republic	0.79 (0.63 to 0.96)
Palestine	0.77 (0.58 to 0.98)
Kingdom of Cambodia	0.75 (0.47 to 1.21)
Democratic Republic of Timor-Leste	0.74 (0.49 to 1.15)
Kingdom of Bahrain	0.71 (0.56 to 0.90)
Mongolia	0.70 (0.53 to 0.89)
Republic of Peru	0.66 (0.49 to 0.87)
United Mexican States	0.63 (0.56 to 0.71)
Republic of Maldives	0.60 (0.31 to 0.97)
Sultanate of Oman	0.56 (0.35 to 0.88)
Democratic People's Republic of Korea	0.55 (0.41 to 0.72)
Islamic Republic of Iran	0.53 (0.47 to 0.60)
Republic of El Salvador	0.53 (0.42 to 0.68)
People's Republic of China	0.46 (0.36 to 0.59)
Republic of Kiribati	0.45 (0.34 to 0.58)
Republic of Tunisia	0.45 (0.29 to 0.66)
Arab Republic of Egypt	0.45 (0.36 to 0.56)
Kingdom of Morocco	0.44 (0.31 to 0.57)
Republic of Guatemala	0.43 (0.37 to 0.50)
Syrian Arab Republic	0.42 (0.31 to 0.56)
People's Democratic Republic of Algeria	0.42 (0.32 to 0.55)
Republic of Iraq	0.38 (0.28 to 0.48)
Democratic Socialist Republic of Sri Lanka	0.37 (0.25 to 0.50)
State of Libya	0.35 (0.21 to 0.54)
Republic of Nicaragua	0.34 (0.28 to 0.42)
Republic of Sudan	0.34 (0.22 to 0.49)
Republic of Yemen	0.33 (0.20 to 0.51)
Islamic Republic of Afghanistan	0.29 (0.19 to 0.42)
Republic of Tajikistan	0.25 (0.18 to 0.34)
Kingdom of Saudi Arabia	0.21 (0.16 to 0.28)

Data for Figure 2

Fig 2A. ASDR across Four World Regions from 1990 to 2021

Year	Location	ASDR per 100,000
1990	Africa	1.82 (1.14 to 2.82)
1991	Africa	1.82 (1.13 to 2.84)
1992	Africa	1.84 (1.16 to 2.88)
1993	Africa	1.85 (1.16 to 2.91)
1994	Africa	1.86 (1.18 to 2.88)
1995	Africa	1.87 (1.18 to 2.90)
1996	Africa	1.89 (1.20 to 2.89)
1997	Africa	1.90 (1.22 to 2.90)
1998	Africa	1.89 (1.22 to 2.94)
1999	Africa	1.87 (1.21 to 2.88)
2000	Africa	1.86 (1.20 to 2.91)
2001	Africa	1.83 (1.19 to 2.83)
2002	Africa	1.79 (1.16 to 2.79)
2003	Africa	1.74 (1.14 to 2.70)
2004	Africa	1.70 (1.11 to 2.64)
2005	Africa	1.66 (1.08 to 2.59)
2006	Africa	1.64 (1.07 to 2.53)
2007	Africa	1.61 (1.04 to 2.48)
2008	Africa	1.60 (1.03 to 2.46)
2009	Africa	1.59 (1.02 to 2.44)
2010	Africa	1.58 (1.02 to 2.43)
2011	Africa	1.58 (1.02 to 2.44)
2012	Africa	1.58 (1.01 to 2.44)
2013	Africa	1.59 (1.01 to 2.41)
2014	Africa	1.59 (1.01 to 2.42)
2015	Africa	1.60 (1.01 to 2.45)
2016	Africa	1.61 (1.02 to 2.44)
2017	Africa	1.62 (1.02 to 2.45)
2018	Africa	1.63 (1.03 to 2.46)
2019	Africa	1.64 (1.03 to 2.46)
2020	Africa	1.65 (1.05 to 2.47)
2021	Africa	1.65 (1.04 to 2.47)
1990	America	4.43 (4.14 to 4.59)
1991	America	4.42 (4.13 to 4.58)
1992	America	4.35 (4.07 to 4.50)
1993	America	4.33 (4.05 to 4.48)
1994	America	4.28 (4.00 to 4.42)
1995	America	4.26 (3.98 to 4.40)
1996	America	4.20 (3.92 to 4.35)

1997	America	4.14 (3.87 to 4.29)
1998	America	4.06 (3.79 to 4.21)
1999	America	4.01 (3.73 to 4.14)
2000	America	3.92 (3.65 to 4.05)
2001	America	3.81 (3.54 to 3.94)
2002	America	3.71 (3.45 to 3.84)
2003	America	3.63 (3.37 to 3.75)
2004	America	3.48 (3.22 to 3.60)
2005	America	3.38 (3.13 to 3.51)
2006	America	3.26 (3.01 to 3.38)
2007	America	3.13 (2.89 to 3.26)
2008	America	2.94 (2.71 to 3.05)
2009	America	2.81 (2.59 to 2.92)
2010	America	2.70 (2.49 to 2.81)
2011	America	2.62 (2.41 to 2.72)
2012	America	2.54 (2.33 to 2.64)
2013	America	2.49 (2.28 to 2.58)
2014	America	2.45 (2.25 to 2.55)
2015	America	2.43 (2.23 to 2.52)
2016	America	2.41 (2.22 to 2.51)
2017	America	2.36 (2.16 to 2.45)
2018	America	2.33 (2.13 to 2.42)
2019	America	2.33 (2.13 to 2.42)
2020	America	2.32 (2.12 to 2.42)
2021	America	2.33 (2.12 to 2.45)
1990	Asia	1.01 (0.87 to 1.18)
1991	Asia	1.03 (0.89 to 1.21)
1992	Asia	1.05 (0.90 to 1.23)
1993	Asia	1.07 (0.93 to 1.26)
1994	Asia	1.09 (0.95 to 1.27)
1995	Asia	1.12 (0.98 to 1.31)
1996	Asia	1.14 (0.99 to 1.32)
1997	Asia	1.14 (1.00 to 1.32)
1998	Asia	1.16 (1.01 to 1.34)
1999	Asia	1.17 (1.03 to 1.35)
2000	Asia	1.18 (1.03 to 1.36)
2001	Asia	1.21 (1.06 to 1.39)
2002	Asia	1.22 (1.06 to 1.40)
2003	Asia	1.24 (1.08 to 1.42)
2004	Asia	1.27 (1.10 to 1.45)
2005	Asia	1.28 (1.12 to 1.46)
2006	Asia	1.29 (1.12 to 1.47)

2007	Asia	1.32 (1.15 to 1.50)
2008	Asia	1.34 (1.16 to 1.53)
2009	Asia	1.36 (1.19 to 1.55)
2010	Asia	1.40 (1.21 to 1.58)
2011	Asia	1.41 (1.23 to 1.59)
2012	Asia	1.39 (1.20 to 1.58)
2013	Asia	1.39 (1.20 to 1.59)
2014	Asia	1.40 (1.21 to 1.61)
2015	Asia	1.41 (1.22 to 1.61)
2016	Asia	1.44 (1.22 to 1.62)
2017	Asia	1.45 (1.23 to 1.64)
2018	Asia	1.44 (1.23 to 1.63)
2019	Asia	1.43 (1.23 to 1.63)
2020	Asia	1.40 (1.19 to 1.58)
2021	Asia	1.40 (1.20 to 1.59)
1990	Europe	3.95 (3.74 to 4.06)
1991	Europe	4.04 (3.82 to 4.15)
1992	Europe	4.12 (3.90 to 4.23)
1993	Europe	4.28 (4.04 to 4.38)
1994	Europe	4.36 (4.13 to 4.47)
1995	Europe	4.37 (4.13 to 4.48)
1996	Europe	4.32 (4.08 to 4.43)
1997	Europe	4.22 (3.98 to 4.34)
1998	Europe	4.18 (3.95 to 4.30)
1999	Europe	4.31 (4.07 to 4.43)
2000	Europe	4.23 (3.99 to 4.35)
2001	Europe	4.01 (3.76 to 4.13)
2002	Europe	3.97 (3.73 to 4.09)
2003	Europe	3.93 (3.68 to 4.05)
2004	Europe	3.84 (3.61 to 3.97)
2005	Europe	3.79 (3.55 to 3.92)
2006	Europe	3.68 (3.44 to 3.81)
2007	Europe	3.62 (3.39 to 3.75)
2008	Europe	3.57 (3.34 to 3.69)
2009	Europe	3.46 (3.24 to 3.58)
2010	Europe	3.38 (3.17 to 3.50)
2011	Europe	3.27 (3.06 to 3.39)
2012	Europe	3.23 (3.01 to 3.34)
2013	Europe	3.18 (2.96 to 3.29)
2014	Europe	3.16 (2.95 to 3.27)
2015	Europe	3.15 (2.92 to 3.25)
2016	Europe	3.14 (2.91 to 3.25)

2017	Europe	3.07 (2.86 to 3.18)
2018	Europe	3.07 (2.86 to 3.19)
2019	Europe	3.04 (2.81 to 3.16)
2020	Europe	3.00 (2.78 to 3.15)
2021	Europe	2.97 (2.76 to 3.15)

Fig 2B. ASDR across Health System Groupings from 1990 to 2021

Year	Location	ASDR per 100,000
1990	Advanced Health System	4.15 (3.90 to 4.28)
1991	Advanced Health System	4.21 (3.94 to 4.34)
1992	Advanced Health System	4.23 (3.97 to 4.35)
1993	Advanced Health System	4.32 (4.05 to 4.44)
1994	Advanced Health System	4.36 (4.09 to 4.48)
1995	Advanced Health System	4.36 (4.09 to 4.49)
1996	Advanced Health System	4.31 (4.03 to 4.44)
1997	Advanced Health System	4.23 (3.95 to 4.36)
1998	Advanced Health System	4.19 (3.91 to 4.32)
1999	Advanced Health System	4.25 (3.96 to 4.38)
2000	Advanced Health System	4.17 (3.88 to 4.31)
2001	Advanced Health System	4.01 (3.72 to 4.15)
2002	Advanced Health System	3.96 (3.67 to 4.10)
2003	Advanced Health System	3.90 (3.61 to 4.04)
2004	Advanced Health System	3.81 (3.52 to 3.96)
2005	Advanced Health System	3.76 (3.47 to 3.91)
2006	Advanced Health System	3.65 (3.36 to 3.80)
2007	Advanced Health System	3.58 (3.29 to 3.73)
2008	Advanced Health System	3.49 (3.20 to 3.64)
2009	Advanced Health System	3.40 (3.11 to 3.55)
2010	Advanced Health System	3.34 (3.05 to 3.49)
2011	Advanced Health System	3.26 (2.97 to 3.40)
2012	Advanced Health System	3.20 (2.91 to 3.34)
2013	Advanced Health System	3.15 (2.86 to 3.29)
2014	Advanced Health System	3.13 (2.84 to 3.28)
2015	Advanced Health System	3.11 (2.81 to 3.26)
2016	Advanced Health System	3.12 (2.82 to 3.27)
2017	Advanced Health System	3.09 (2.78 to 3.24)
2018	Advanced Health System	3.07 (2.76 to 3.22)
2019	Advanced Health System	3.04 (2.73 to 3.19)
2020	Advanced Health System	2.98 (2.68 to 3.16)
2021	Advanced Health System	2.99 (2.69 to 3.17)
1990	Basic Health System	1.02 (0.94 to 1.11)
1991	Basic Health System	1.03 (0.95 to 1.11)
1992	Basic Health System	1.06 (0.98 to 1.14)
1993	Basic Health System	1.08 (1.00 to 1.16)
1994	Basic Health System	1.09 (1.01 to 1.17)
1995	Basic Health System	1.10 (1.02 to 1.18)
1996	Basic Health System	1.11 (1.04 to 1.18)
1997	Basic Health System	1.11 (1.04 to 1.18)

1998	Basic Health System	1.11 (1.04 to 1.18)
1999	Basic Health System	1.12 (1.04 to 1.18)
2000	Basic Health System	1.13 (1.05 to 1.19)
2001	Basic Health System	1.12 (1.05 to 1.19)
2002	Basic Health System	1.13 (1.05 to 1.19)
2003	Basic Health System	1.14 (1.06 to 1.20)
2004	Basic Health System	1.13 (1.05 to 1.19)
2005	Basic Health System	1.12 (1.05 to 1.18)
2006	Basic Health System	1.12 (1.04 to 1.17)
2007	Basic Health System	1.12 (1.05 to 1.17)
2008	Basic Health System	1.12 (1.04 to 1.17)
2009	Basic Health System	1.11 (1.04 to 1.16)
2010	Basic Health System	1.11 (1.04 to 1.16)
2011	Basic Health System	1.10 (1.03 to 1.14)
2012	Basic Health System	1.08 (1.00 to 1.13)
2013	Basic Health System	1.06 (0.99 to 1.12)
2014	Basic Health System	1.06 (0.99 to 1.12)
2015	Basic Health System	1.06 (0.99 to 1.12)
2016	Basic Health System	1.07 (0.98 to 1.13)
2017	Basic Health System	1.05 (0.97 to 1.12)
2018	Basic Health System	1.05 (0.97 to 1.13)
2019	Basic Health System	1.06 (0.97 to 1.14)
2020	Basic Health System	1.05 (0.96 to 1.15)
2021	Basic Health System	1.05 (0.95 to 1.15)
1990	Limited Health System	0.99 (0.73 to 1.45)
1991	Limited Health System	0.99 (0.74 to 1.46)
1992	Limited Health System	1.01 (0.75 to 1.48)
1993	Limited Health System	1.02 (0.77 to 1.49)
1994	Limited Health System	1.04 (0.79 to 1.52)
1995	Limited Health System	1.07 (0.81 to 1.58)
1996	Limited Health System	1.08 (0.81 to 1.56)
1997	Limited Health System	1.07 (0.81 to 1.55)
1998	Limited Health System	1.06 (0.81 to 1.52)
1999	Limited Health System	1.06 (0.81 to 1.53)
2000	Limited Health System	1.05 (0.81 to 1.50)
2001	Limited Health System	1.05 (0.81 to 1.51)
2002	Limited Health System	1.05 (0.81 to 1.50)
2003	Limited Health System	1.06 (0.81 to 1.52)
2004	Limited Health System	1.08 (0.82 to 1.55)
2005	Limited Health System	1.07 (0.82 to 1.51)
2006	Limited Health System	1.07 (0.83 to 1.52)
2007	Limited Health System	1.09 (0.85 to 1.55)

2008	Limited Health System	1.09 (0.87 to 1.54)
2009	Limited Health System	1.10 (0.87 to 1.55)
2010	Limited Health System	1.12 (0.89 to 1.57)
2011	Limited Health System	1.13 (0.89 to 1.61)
2012	Limited Health System	1.15 (0.90 to 1.64)
2013	Limited Health System	1.19 (0.94 to 1.68)
2014	Limited Health System	1.23 (0.98 to 1.76)
2015	Limited Health System	1.26 (0.99 to 1.79)
2016	Limited Health System	1.27 (1.00 to 1.81)
2017	Limited Health System	1.30 (1.03 to 1.83)
2018	Limited Health System	1.31 (1.05 to 1.84)
2019	Limited Health System	1.33 (1.07 to 1.88)
2020	Limited Health System	1.33 (1.07 to 1.89)
2021	Limited Health System	1.32 (1.06 to 1.83)
1990	Minimal Health System	1.71 (0.93 to 3.14)
1991	Minimal Health System	1.71 (0.93 to 3.15)
1992	Minimal Health System	1.71 (0.93 to 3.13)
1993	Minimal Health System	1.71 (0.93 to 3.12)
1994	Minimal Health System	1.71 (0.93 to 3.13)
1995	Minimal Health System	1.71 (0.93 to 3.13)
1996	Minimal Health System	1.71 (0.93 to 3.14)
1997	Minimal Health System	1.69 (0.91 to 3.11)
1998	Minimal Health System	1.68 (0.90 to 3.09)
1999	Minimal Health System	1.67 (0.88 to 3.08)
2000	Minimal Health System	1.66 (0.87 to 3.08)
2001	Minimal Health System	1.64 (0.86 to 3.08)
2002	Minimal Health System	1.60 (0.83 to 3.04)
2003	Minimal Health System	1.57 (0.80 to 2.97)
2004	Minimal Health System	1.54 (0.79 to 2.92)
2005	Minimal Health System	1.52 (0.78 to 2.89)
2006	Minimal Health System	1.52 (0.78 to 2.92)
2007	Minimal Health System	1.51 (0.78 to 2.92)
2008	Minimal Health System	1.52 (0.79 to 2.94)
2009	Minimal Health System	1.53 (0.79 to 2.96)
2010	Minimal Health System	1.55 (0.79 to 2.97)
2011	Minimal Health System	1.57 (0.80 to 2.99)
2012	Minimal Health System	1.58 (0.80 to 2.99)
2013	Minimal Health System	1.60 (0.81 to 3.01)
2014	Minimal Health System	1.61 (0.81 to 3.01)
2015	Minimal Health System	1.62 (0.82 to 3.02)
2016	Minimal Health System	1.63 (0.82 to 3.05)
2017	Minimal Health System	1.64 (0.82 to 3.06)

2018	Minimal Health System	1.65 (0.82 to 3.07)
2019	Minimal Health System	1.67 (0.82 to 3.08)
2020	Minimal Health System	1.68 (0.83 to 3.09)
2021	Minimal Health System	1.70 (0.84 to 3.09)

Fig 2C. ASDR across different Commonwealth Income regions from 1990 to 2021

Year	Location	ASDR per 100,000
1990	Commonwealth High Income	8.66 (8.18 to 8.94)
1991	Commonwealth High Income	8.67 (8.18 to 8.95)
1992	Commonwealth High Income	8.78 (8.26 to 9.06)
1993	Commonwealth High Income	8.79 (8.28 to 9.06)
1994	Commonwealth High Income	8.71 (8.19 to 8.99)
1995	Commonwealth High Income	8.74 (8.23 to 9.02)
1996	Commonwealth High Income	8.69 (8.19 to 8.96)
1997	Commonwealth High Income	8.49 (7.98 to 8.77)
1998	Commonwealth High Income	8.38 (7.86 to 8.66)
1999	Commonwealth High Income	8.21 (7.71 to 8.49)
2000	Commonwealth High Income	7.96 (7.46 to 8.25)
2001	Commonwealth High Income	7.65 (7.13 to 7.94)
2002	Commonwealth High Income	7.40 (6.89 to 7.67)
2003	Commonwealth High Income	7.10 (6.61 to 7.37)
2004	Commonwealth High Income	6.79 (6.29 to 7.06)
2005	Commonwealth High Income	6.44 (5.93 to 6.69)
2006	Commonwealth High Income	6.14 (5.63 to 6.40)
2007	Commonwealth High Income	5.85 (5.35 to 6.10)
2008	Commonwealth High Income	5.51 (5.02 to 5.74)
2009	Commonwealth High Income	5.15 (4.68 to 5.38)
2010	Commonwealth High Income	4.87 (4.43 to 5.09)
2011	Commonwealth High Income	4.55 (4.13 to 4.75)
2012	Commonwealth High Income	4.36 (3.94 to 4.55)
2013	Commonwealth High Income	4.15 (3.76 to 4.34)
2014	Commonwealth High Income	4.00 (3.62 to 4.18)
2015	Commonwealth High Income	3.84 (3.47 to 4.02)
2016	Commonwealth High Income	3.71 (3.35 to 3.88)
2017	Commonwealth High Income	3.57 (3.22 to 3.73)
2018	Commonwealth High Income	3.48 (3.13 to 3.64)
2019	Commonwealth High Income	3.37 (3.03 to 3.53)
2020	Commonwealth High Income	3.22 (2.87 to 3.38)
2021	Commonwealth High Income	3.22 (2.86 to 3.40)
1990	Commonwealth Low Income	1.27 (0.80 to 2.11)
1991	Commonwealth Low Income	1.28 (0.80 to 2.14)
1992	Commonwealth Low Income	1.31 (0.81 to 2.18)
1993	Commonwealth Low Income	1.33 (0.82 to 2.21)
1994	Commonwealth Low Income	1.34 (0.82 to 2.24)
1995	Commonwealth Low Income	1.36 (0.83 to 2.28)
1996	Commonwealth Low Income	1.36 (0.83 to 2.29)
1997	Commonwealth Low Income	1.37 (0.84 to 2.30)

1998	Commonwealth Low Income	1.38 (0.84 to 2.27)
1999	Commonwealth Low Income	1.40 (0.86 to 2.31)
2000	Commonwealth Low Income	1.41 (0.86 to 2.31)
2001	Commonwealth Low Income	1.40 (0.86 to 2.28)
2002	Commonwealth Low Income	1.40 (0.87 to 2.27)
2003	Commonwealth Low Income	1.39 (0.87 to 2.26)
2004	Commonwealth Low Income	1.38 (0.86 to 2.27)
2005	Commonwealth Low Income	1.37 (0.85 to 2.25)
2006	Commonwealth Low Income	1.38 (0.86 to 2.27)
2007	Commonwealth Low Income	1.40 (0.89 to 2.26)
2008	Commonwealth Low Income	1.41 (0.89 to 2.26)
2009	Commonwealth Low Income	1.41 (0.90 to 2.27)
2010	Commonwealth Low Income	1.42 (0.91 to 2.26)
2011	Commonwealth Low Income	1.39 (0.87 to 2.25)
2012	Commonwealth Low Income	1.35 (0.84 to 2.20)
2013	Commonwealth Low Income	1.35 (0.84 to 2.19)
2014	Commonwealth Low Income	1.38 (0.86 to 2.23)
2015	Commonwealth Low Income	1.39 (0.88 to 2.25)
2016	Commonwealth Low Income	1.41 (0.88 to 2.31)
2017	Commonwealth Low Income	1.47 (0.92 to 2.33)
2018	Commonwealth Low Income	1.49 (0.94 to 2.34)
2019	Commonwealth Low Income	1.51 (0.97 to 2.36)
2020	Commonwealth Low Income	1.52 (0.98 to 2.38)
2021	Commonwealth Low Income	1.53 (0.98 to 2.38)
1990	Commonwealth Middle Income	1.06 (0.83 to 1.45)
1991	Commonwealth Middle Income	1.07 (0.84 to 1.47)
1992	Commonwealth Middle Income	1.09 (0.87 to 1.48)
1993	Commonwealth Middle Income	1.11 (0.88 to 1.51)
1994	Commonwealth Middle Income	1.13 (0.90 to 1.54)
1995	Commonwealth Middle Income	1.18 (0.93 to 1.60)
1996	Commonwealth Middle Income	1.19 (0.94 to 1.59)
1997	Commonwealth Middle Income	1.19 (0.95 to 1.60)
1998	Commonwealth Middle Income	1.18 (0.95 to 1.58)
1999	Commonwealth Middle Income	1.17 (0.94 to 1.56)
2000	Commonwealth Middle Income	1.16 (0.94 to 1.55)
2001	Commonwealth Middle Income	1.15 (0.94 to 1.54)
2002	Commonwealth Middle Income	1.14 (0.93 to 1.54)
2003	Commonwealth Middle Income	1.16 (0.94 to 1.56)
2004	Commonwealth Middle Income	1.18 (0.95 to 1.59)
2005	Commonwealth Middle Income	1.16 (0.94 to 1.58)
2006	Commonwealth Middle Income	1.15 (0.94 to 1.57)
2007	Commonwealth Middle Income	1.17 (0.96 to 1.59)

2008	Commonwealth Middle Income	1.17 (0.96 to 1.58)
2009	Commonwealth Middle Income	1.18 (0.96 to 1.61)
2010	Commonwealth Middle Income	1.19 (0.97 to 1.62)
2011	Commonwealth Middle Income	1.21 (0.98 to 1.65)
2012	Commonwealth Middle Income	1.23 (0.99 to 1.67)
2013	Commonwealth Middle Income	1.26 (1.03 to 1.73)
2014	Commonwealth Middle Income	1.31 (1.07 to 1.81)
2015	Commonwealth Middle Income	1.35 (1.08 to 1.85)
2016	Commonwealth Middle Income	1.36 (1.10 to 1.85)
2017	Commonwealth Middle Income	1.38 (1.13 to 1.85)
2018	Commonwealth Middle Income	1.39 (1.14 to 1.87)
2019	Commonwealth Middle Income	1.41 (1.15 to 1.90)
2020	Commonwealth Middle Income	1.39 (1.13 to 1.89)
2021	Commonwealth Middle Income	1.39 (1.13 to 1.85)

Fig 2D. ASDR across SDI regions from 1990 to 2021

Year	Location	ASDR per 100,000
1990	High SDI	4.76 (4.46 to 4.91)
1991	High SDI	4.81 (4.49 to 4.96)
1992	High SDI	4.77 (4.45 to 4.92)
1993	High SDI	4.76 (4.45 to 4.91)
1994	High SDI	4.73 (4.42 to 4.89)
1995	High SDI	4.74 (4.43 to 4.90)
1996	High SDI	4.71 (4.39 to 4.87)
1997	High SDI	4.64 (4.32 to 4.79)
1998	High SDI	4.59 (4.26 to 4.74)
1999	High SDI	4.53 (4.19 to 4.69)
2000	High SDI	4.45 (4.11 to 4.62)
2001	High SDI	4.36 (4.01 to 4.53)
2002	High SDI	4.27 (3.93 to 4.44)
2003	High SDI	4.17 (3.82 to 4.34)
2004	High SDI	4.05 (3.70 to 4.22)
2005	High SDI	3.95 (3.60 to 4.12)
2006	High SDI	3.83 (3.49 to 4.00)
2007	High SDI	3.72 (3.38 to 3.89)
2008	High SDI	3.58 (3.24 to 3.75)
2009	High SDI	3.47 (3.13 to 3.64)
2010	High SDI	3.39 (3.04 to 3.56)
2011	High SDI	3.30 (2.96 to 3.47)
2012	High SDI	3.21 (2.87 to 3.38)
2013	High SDI	3.14 (2.80 to 3.30)
2014	High SDI	3.08 (2.74 to 3.25)
2015	High SDI	3.04 (2.70 to 3.20)
2016	High SDI	3.04 (2.70 to 3.22)
2017	High SDI	3.00 (2.66 to 3.18)
2018	High SDI	2.96 (2.61 to 3.14)
2019	High SDI	2.92 (2.57 to 3.10)
2020	High SDI	2.85 (2.50 to 3.05)
2021	High SDI	2.87 (2.51 to 3.06)
1990	High-middle SDI	1.99 (1.88 to 2.08)
1991	High-middle SDI	2.03 (1.93 to 2.12)
1992	High-middle SDI	2.10 (1.98 to 2.19)
1993	High-middle SDI	2.23 (2.12 to 2.32)
1994	High-middle SDI	2.32 (2.21 to 2.40)
1995	High-middle SDI	2.30 (2.19 to 2.38)
1996	High-middle SDI	2.23 (2.12 to 2.32)
1997	High-middle SDI	2.17 (2.06 to 2.25)

1998	High-middle SDI	2.15 (2.04 to 2.23)
1999	High-middle SDI	2.32 (2.21 to 2.39)
2000	High-middle SDI	2.25 (2.13 to 2.32)
2001	High-middle SDI	2.06 (1.95 to 2.13)
2002	High-middle SDI	2.06 (1.95 to 2.12)
2003	High-middle SDI	2.07 (1.97 to 2.14)
2004	High-middle SDI	2.03 (1.92 to 2.10)
2005	High-middle SDI	2.05 (1.94 to 2.12)
2006	High-middle SDI	2.00 (1.89 to 2.07)
2007	High-middle SDI	2.00 (1.89 to 2.07)
2008	High-middle SDI	1.99 (1.89 to 2.06)
2009	High-middle SDI	1.96 (1.86 to 2.03)
2010	High-middle SDI	1.95 (1.84 to 2.01)
2011	High-middle SDI	1.91 (1.81 to 1.97)
2012	High-middle SDI	1.89 (1.79 to 1.96)
2013	High-middle SDI	1.87 (1.77 to 1.94)
2014	High-middle SDI	1.89 (1.79 to 1.97)
2015	High-middle SDI	1.90 (1.79 to 1.98)
2016	High-middle SDI	1.90 (1.78 to 1.99)
2017	High-middle SDI	1.87 (1.75 to 1.96)
2018	High-middle SDI	1.87 (1.75 to 1.96)
2019	High-middle SDI	1.85 (1.73 to 1.95)
2020	High-middle SDI	1.82 (1.70 to 1.93)
2021	High-middle SDI	1.79 (1.66 to 1.92)
1990	Low SDI	1.37 (0.83 to 2.37)
1991	Low SDI	1.37 (0.83 to 2.36)
1992	Low SDI	1.38 (0.84 to 2.37)
1993	Low SDI	1.39 (0.84 to 2.40)
1994	Low SDI	1.40 (0.85 to 2.43)
1995	Low SDI	1.41 (0.85 to 2.45)
1996	Low SDI	1.40 (0.84 to 2.41)
1997	Low SDI	1.38 (0.83 to 2.41)
1998	Low SDI	1.37 (0.82 to 2.40)
1999	Low SDI	1.35 (0.81 to 2.38)
2000	Low SDI	1.34 (0.80 to 2.33)
2001	Low SDI	1.32 (0.80 to 2.30)
2002	Low SDI	1.30 (0.79 to 2.27)
2003	Low SDI	1.29 (0.78 to 2.24)
2004	Low SDI	1.27 (0.76 to 2.21)
2005	Low SDI	1.25 (0.75 to 2.18)
2006	Low SDI	1.25 (0.74 to 2.17)
2007	Low SDI	1.25 (0.74 to 2.17)

2008	Low SDI	1.25 (0.75 to 2.16)
2009	Low SDI	1.26 (0.75 to 2.20)
2010	Low SDI	1.28 (0.76 to 2.21)
2011	Low SDI	1.30 (0.78 to 2.24)
2012	Low SDI	1.33 (0.80 to 2.28)
2013	Low SDI	1.39 (0.84 to 2.34)
2014	Low SDI	1.42 (0.86 to 2.41)
2015	Low SDI	1.44 (0.87 to 2.47)
2016	Low SDI	1.46 (0.88 to 2.46)
2017	Low SDI	1.49 (0.90 to 2.49)
2018	Low SDI	1.48 (0.89 to 2.46)
2019	Low SDI	1.49 (0.91 to 2.46)
2020	Low SDI	1.49 (0.92 to 2.47)
2021	Low SDI	1.48 (0.91 to 2.44)
1990	Low-middle SDI	0.89 (0.71 to 1.20)
1991	Low-middle SDI	0.90 (0.71 to 1.21)
1992	Low-middle SDI	0.92 (0.73 to 1.23)
1993	Low-middle SDI	0.93 (0.74 to 1.25)
1994	Low-middle SDI	0.95 (0.77 to 1.27)
1995	Low-middle SDI	0.98 (0.79 to 1.31)
1996	Low-middle SDI	1.00 (0.80 to 1.33)
1997	Low-middle SDI	1.00 (0.80 to 1.34)
1998	Low-middle SDI	1.01 (0.82 to 1.34)
1999	Low-middle SDI	1.02 (0.83 to 1.34)
2000	Low-middle SDI	1.03 (0.84 to 1.36)
2001	Low-middle SDI	1.03 (0.85 to 1.37)
2002	Low-middle SDI	1.04 (0.86 to 1.38)
2003	Low-middle SDI	1.06 (0.86 to 1.40)
2004	Low-middle SDI	1.07 (0.88 to 1.42)
2005	Low-middle SDI	1.07 (0.88 to 1.42)
2006	Low-middle SDI	1.07 (0.88 to 1.42)
2007	Low-middle SDI	1.10 (0.91 to 1.47)
2008	Low-middle SDI	1.12 (0.92 to 1.48)
2009	Low-middle SDI	1.13 (0.93 to 1.50)
2010	Low-middle SDI	1.14 (0.95 to 1.52)
2011	Low-middle SDI	1.15 (0.96 to 1.54)
2012	Low-middle SDI	1.16 (0.97 to 1.55)
2013	Low-middle SDI	1.19 (0.99 to 1.58)
2014	Low-middle SDI	1.22 (1.01 to 1.65)
2015	Low-middle SDI	1.24 (1.03 to 1.66)
2016	Low-middle SDI	1.26 (1.06 to 1.68)
2017	Low-middle SDI	1.29 (1.08 to 1.72)

2018	Low-middle SDI	1.31 (1.09 to 1.73)
2019	Low-middle SDI	1.33 (1.11 to 1.79)
2020	Low-middle SDI	1.33 (1.10 to 1.79)
2021	Low-middle SDI	1.31 (1.09 to 1.76)
1990	Middle SDI	1.03 (0.94 to 1.14)
1991	Middle SDI	1.04 (0.96 to 1.16)
1992	Middle SDI	1.07 (0.98 to 1.19)
1993	Middle SDI	1.10 (1.01 to 1.21)
1994	Middle SDI	1.12 (1.04 to 1.24)
1995	Middle SDI	1.14 (1.05 to 1.25)
1996	Middle SDI	1.16 (1.07 to 1.27)
1997	Middle SDI	1.15 (1.07 to 1.26)
1998	Middle SDI	1.16 (1.07 to 1.27)
1999	Middle SDI	1.16 (1.08 to 1.27)
2000	Middle SDI	1.17 (1.09 to 1.28)
2001	Middle SDI	1.18 (1.09 to 1.29)
2002	Middle SDI	1.17 (1.08 to 1.28)
2003	Middle SDI	1.18 (1.09 to 1.28)
2004	Middle SDI	1.20 (1.11 to 1.30)
2005	Middle SDI	1.18 (1.10 to 1.29)
2006	Middle SDI	1.18 (1.09 to 1.27)
2007	Middle SDI	1.17 (1.09 to 1.27)
2008	Middle SDI	1.17 (1.09 to 1.26)
2009	Middle SDI	1.17 (1.07 to 1.25)
2010	Middle SDI	1.17 (1.07 to 1.25)
2011	Middle SDI	1.15 (1.06 to 1.24)
2012	Middle SDI	1.14 (1.05 to 1.22)
2013	Middle SDI	1.13 (1.04 to 1.22)
2014	Middle SDI	1.14 (1.04 to 1.23)
2015	Middle SDI	1.15 (1.06 to 1.23)
2016	Middle SDI	1.15 (1.06 to 1.24)
2017	Middle SDI	1.14 (1.04 to 1.22)
2018	Middle SDI	1.14 (1.04 to 1.22)
2019	Middle SDI	1.16 (1.05 to 1.24)
2020	Middle SDI	1.16 (1.05 to 1.26)
2021	Middle SDI	1.15 (1.04 to 1.25)

SDI, Socio-Demographic Index.

Fig 2E. ASDR in men and women across the Four World Regions in 2021

Sex	Location	ASDR per 100,000
Male	Africa	2.16 (1.21 to 3.36)
Female	Africa	1.20 (0.76 to 1.75)
Male	America	3.17 (2.97 to 3.32)
Female	America	1.63 (1.42 to 1.76)
Male	Asia	1.81 (1.60 to 2.11)
Female	Asia	1.05 (0.85 to 1.21)
Male	Europe	4.69 (4.38 to 4.92)
Female	Europe	1.70 (1.50 to 1.92)

ASDR, age-standardized death rates.

Fig 2F. ASDR in men and women across Health System Groupings in 2021

Sex	Location	ASDR per 100,000
Male	Basic Health System	1.49 (1.33 to 1.65)
Female	Basic Health System	0.68 (0.59 to 0.75)
Male	Limited Health System	1.72 (1.35 to 2.67)
Female	Limited Health System	0.96 (0.70 to 1.25)
Male	Minimal Health System	2.06 (0.68 to 4.01)
Female	Minimal Health System	1.38 (0.71 to 2.50)
Male	Advanced Health System	4.31 (3.99 to 4.49)
Female	Advanced Health System	1.94 (1.63 to 2.18)

ASDR, age-standardized death rates.

Fig 2G. ASDR in men and women across different Commonwealth Income regions in 2021

Sex	Location	ASDR per 100,000
Male	Commonwealth High Income	4.42 (4.07 to 4.62)
Female	Commonwealth High Income	2.25 (1.90 to 2.44)
Male	Commonwealth Middle Income	1.85 (1.46 to 2.74)
Female	Commonwealth Middle Income	0.99 (0.73 to 1.27)
Male	Commonwealth Low Income	1.96 (1.17 to 3.42)
Female	Commonwealth Low Income	1.12 (0.74 to 1.70)

ASDR, age-standardized death rates.

Fig 2H. ASDR in men and women across SDI regions in 2021

Sex	Location	ASDR per 100,000
Female	High SDI	2.00 (1.63 to 2.24)
Male	High SDI	3.92 (3.60 to 4.09)
Female	High-middle SDI	1.01 (0.90 to 1.12)
Male	High-middle SDI	2.79 (2.59 to 3.00)
Female	Low SDI	1.17 (0.74 to 1.81)

Male	Low SDI	1.81 (0.88 to 3.18)
Female	Low-middle SDI	0.96 (0.74 to 1.21)
Male	Low-middle SDI	1.71 (1.38 to 2.54)
Female	Middle SDI	0.78 (0.69 to 0.87)
Male	Middle SDI	1.60 (1.44 to 1.80)

ASDR, age-standardized death rates.

Data for Figure 3

Fig 3A. ASDR in Europe from 1990 to 2021

Year	Location	ASDR per 100,000
1990	Central Europe	3.07 (2.95 to 3.18)
1991	Central Europe	3.30 (3.16 to 3.41)
1992	Central Europe	3.38 (3.23 to 3.49)
1993	Central Europe	3.39 (3.23 to 3.50)
1994	Central Europe	3.43 (3.27 to 3.54)
1995	Central Europe	3.44 (3.29 to 3.54)
1996	Central Europe	3.42 (3.28 to 3.52)
1997	Central Europe	3.39 (3.25 to 3.49)
1998	Central Europe	3.24 (3.11 to 3.34)
1999	Central Europe	3.27 (3.14 to 3.37)
2000	Central Europe	3.34 (3.19 to 3.43)
2001	Central Europe	3.40 (3.25 to 3.50)
2002	Central Europe	3.45 (3.30 to 3.55)
2003	Central Europe	3.51 (3.37 to 3.62)
2004	Central Europe	3.52 (3.39 to 3.62)
2005	Central Europe	3.49 (3.35 to 3.60)
2006	Central Europe	3.46 (3.31 to 3.56)
2007	Central Europe	3.45 (3.30 to 3.55)
2008	Central Europe	3.44 (3.30 to 3.54)
2009	Central Europe	3.39 (3.24 to 3.49)
2010	Central Europe	3.29 (3.15 to 3.40)
2011	Central Europe	3.20 (3.05 to 3.31)
2012	Central Europe	3.23 (3.07 to 3.35)
2013	Central Europe	3.17 (3.01 to 3.28)
2014	Central Europe	3.09 (2.93 to 3.20)
2015	Central Europe	3.06 (2.90 to 3.17)
2016	Central Europe	3.00 (2.85 to 3.11)
2017	Central Europe	2.97 (2.81 to 3.08)
2018	Central Europe	2.97 (2.81 to 3.09)
2019	Central Europe	2.91 (2.75 to 3.03)
2020	Central Europe	2.93 (2.75 to 3.14)
2021	Central Europe	2.93 (2.69 to 3.21)
1990	Eastern Europe	2.52 (2.43 to 2.62)
1991	Eastern Europe	2.60 (2.51 to 2.70)
1992	Eastern Europe	2.78 (2.69 to 2.88)
1993	Eastern Europe	3.24 (3.14 to 3.34)
1994	Eastern Europe	3.52 (3.41 to 3.63)
1995	Eastern Europe	3.33 (3.23 to 3.45)
1996	Eastern Europe	3.10 (3.01 to 3.20)

1997	Eastern Europe	2.93 (2.84 to 3.02)
1998	Eastern Europe	2.91 (2.82 to 3.00)
1999	Eastern Europe	3.68 (3.56 to 3.81)
2000	Eastern Europe	3.51 (3.40 to 3.64)
2001	Eastern Europe	2.84 (2.76 to 2.94)
2002	Eastern Europe	2.83 (2.74 to 2.93)
2003	Eastern Europe	2.85 (2.75 to 2.94)
2004	Eastern Europe	2.86 (2.77 to 2.95)
2005	Eastern Europe	3.09 (2.99 to 3.20)
2006	Eastern Europe	3.01 (2.91 to 3.10)
2007	Eastern Europe	3.08 (2.98 to 3.18)
2008	Eastern Europe	3.24 (3.14 to 3.34)
2009	Eastern Europe	3.24 (3.13 to 3.33)
2010	Eastern Europe	3.34 (3.23 to 3.42)
2011	Eastern Europe	3.32 (3.21 to 3.40)
2012	Eastern Europe	3.36 (3.24 to 3.44)
2013	Eastern Europe	3.49 (3.36 to 3.56)
2014	Eastern Europe	3.71 (3.57 to 3.80)
2015	Eastern Europe	3.78 (3.62 to 3.86)
2016	Eastern Europe	3.87 (3.71 to 3.96)
2017	Eastern Europe	3.80 (3.63 to 3.90)
2018	Eastern Europe	3.87 (3.69 to 4.00)
2019	Eastern Europe	3.88 (3.70 to 4.04)
2020	Eastern Europe	3.84 (3.64 to 4.04)
2021	Eastern Europe	3.82 (3.52 to 4.12)
1990	Western Europe	4.78 (4.50 to 4.92)
1991	Western Europe	4.84 (4.54 to 4.98)
1992	Western Europe	4.87 (4.58 to 5.02)
1993	Western Europe	4.92 (4.61 to 5.07)
1994	Western Europe	4.93 (4.62 to 5.08)
1995	Western Europe	5.01 (4.70 to 5.16)
1996	Western Europe	5.02 (4.72 to 5.18)
1997	Western Europe	4.94 (4.64 to 5.09)
1998	Western Europe	4.92 (4.62 to 5.08)
1999	Western Europe	4.84 (4.54 to 4.99)
2000	Western Europe	4.75 (4.44 to 4.91)
2001	Western Europe	4.61 (4.29 to 4.77)
2002	Western Europe	4.53 (4.21 to 4.69)
2003	Western Europe	4.43 (4.11 to 4.59)
2004	Western Europe	4.27 (3.95 to 4.44)
2005	Western Europe	4.10 (3.78 to 4.25)
2006	Western Europe	3.95 (3.64 to 4.10)

2007	Western Europe	3.82 (3.52 to 3.97)
2008	Western Europe	3.67 (3.36 to 3.81)
2009	Western Europe	3.51 (3.21 to 3.65)
2010	Western Europe	3.36 (3.07 to 3.50)
2011	Western Europe	3.20 (2.92 to 3.34)
2012	Western Europe	3.11 (2.84 to 3.25)
2013	Western Europe	2.99 (2.72 to 3.12)
2014	Western Europe	2.89 (2.63 to 3.03)
2015	Western Europe	2.85 (2.58 to 2.98)
2016	Western Europe	2.81 (2.54 to 2.94)
2017	Western Europe	2.74 (2.48 to 2.87)
2018	Western Europe	2.72 (2.44 to 2.84)
2019	Western Europe	2.66 (2.40 to 2.79)
2020	Western Europe	2.60 (2.33 to 2.74)
2021	Western Europe	2.57 (2.30 to 2.71)

ASDR, age-standardized death rates.

Fig 3B. ASDR in Western Europe from 1990 to 2021

Year	Location	ASDR per 100,000
1990	Andorra	6.87 (4.36 to 10.33)
1991	Andorra	6.89 (4.36 to 10.07)
1992	Andorra	6.90 (4.39 to 10.01)
1993	Andorra	6.99 (4.39 to 10.20)
1994	Andorra	6.95 (4.41 to 10.04)
1995	Andorra	6.88 (4.42 to 9.87)
1996	Andorra	6.82 (4.43 to 9.73)
1997	Andorra	6.71 (4.42 to 9.50)
1998	Andorra	6.62 (4.44 to 9.35)
1999	Andorra	6.54 (4.42 to 9.26)
2000	Andorra	6.47 (4.44 to 9.09)
2001	Andorra	5.96 (3.98 to 8.45)
2002	Andorra	5.57 (3.62 to 8.07)
2003	Andorra	5.43 (3.56 to 7.75)
2004	Andorra	5.28 (3.50 to 7.49)
2005	Andorra	5.04 (3.36 to 7.21)
2006	Andorra	4.93 (3.28 to 7.26)
2007	Andorra	4.79 (3.21 to 7.17)
2008	Andorra	5.00 (3.40 to 7.26)
2009	Andorra	5.17 (3.61 to 7.42)
2010	Andorra	5.29 (3.75 to 7.45)
2011	Andorra	5.08 (3.69 to 7.01)
2012	Andorra	4.90 (3.58 to 6.66)
2013	Andorra	4.88 (3.54 to 6.60)
2014	Andorra	4.88 (3.50 to 6.52)
2015	Andorra	4.83 (3.45 to 6.45)
2016	Andorra	4.82 (3.39 to 6.40)
2017	Andorra	4.79 (3.34 to 6.50)
2018	Andorra	4.76 (3.32 to 6.54)
2019	Andorra	4.70 (3.22 to 6.62)
2020	Andorra	3.96 (2.52 to 5.92)
2021	Andorra	3.96 (2.50 to 5.97)
1990	Austria	3.20 (3.03 to 3.37)
1991	Austria	3.23 (3.03 to 3.41)
1992	Austria	3.27 (3.06 to 3.46)
1993	Austria	3.37 (3.14 to 3.56)
1994	Austria	3.42 (3.18 to 3.63)
1995	Austria	3.42 (3.17 to 3.62)
1996	Austria	3.37 (3.11 to 3.56)
1997	Austria	3.29 (3.02 to 3.48)

1998	Austria	3.13 (2.86 to 3.32)
1999	Austria	2.95 (2.69 to 3.14)
2000	Austria	2.92 (2.67 to 3.11)
2001	Austria	2.93 (2.67 to 3.11)
2002	Austria	2.88 (2.63 to 3.05)
2003	Austria	2.60 (2.37 to 2.75)
2004	Austria	2.46 (2.24 to 2.60)
2005	Austria	2.41 (2.20 to 2.56)
2006	Austria	2.43 (2.20 to 2.58)
2007	Austria	2.36 (2.14 to 2.51)
2008	Austria	2.27 (2.07 to 2.42)
2009	Austria	2.30 (2.09 to 2.46)
2010	Austria	2.24 (2.04 to 2.39)
2011	Austria	2.17 (1.97 to 2.31)
2012	Austria	2.18 (1.98 to 2.32)
2013	Austria	2.18 (2.00 to 2.32)
2014	Austria	2.14 (1.95 to 2.28)
2015	Austria	2.12 (1.94 to 2.26)
2016	Austria	2.08 (1.89 to 2.22)
2017	Austria	1.98 (1.80 to 2.12)
2018	Austria	2.03 (1.82 to 2.16)
2019	Austria	1.94 (1.74 to 2.07)
2020	Austria	1.92 (1.72 to 2.06)
2021	Austria	1.80 (1.61 to 1.94)
1990	Belgium	5.11 (4.73 to 5.45)
1991	Belgium	5.13 (4.74 to 5.48)
1992	Belgium	5.24 (4.84 to 5.59)
1993	Belgium	5.01 (4.62 to 5.34)
1994	Belgium	4.98 (4.58 to 5.29)
1995	Belgium	4.85 (4.47 to 5.15)
1996	Belgium	4.97 (4.57 to 5.29)
1997	Belgium	4.77 (4.37 to 5.06)
1998	Belgium	4.59 (4.22 to 4.87)
1999	Belgium	4.58 (4.22 to 4.86)
2000	Belgium	4.57 (4.19 to 4.87)
2001	Belgium	4.37 (4.00 to 4.66)
2002	Belgium	4.28 (3.94 to 4.57)
2003	Belgium	4.31 (3.93 to 4.59)
2004	Belgium	4.19 (3.82 to 4.48)
2005	Belgium	3.95 (3.59 to 4.24)
2006	Belgium	3.69 (3.34 to 3.96)
2007	Belgium	3.57 (3.22 to 3.83)

2008	Belgium	3.56 (3.22 to 3.83)
2009	Belgium	3.40 (3.07 to 3.65)
2010	Belgium	3.23 (2.93 to 3.48)
2011	Belgium	3.11 (2.81 to 3.34)
2012	Belgium	2.97 (2.68 to 3.18)
2013	Belgium	2.88 (2.59 to 3.08)
2014	Belgium	2.67 (2.39 to 2.87)
2015	Belgium	2.68 (2.41 to 2.89)
2016	Belgium	2.59 (2.32 to 2.78)
2017	Belgium	2.56 (2.28 to 2.75)
2018	Belgium	2.43 (2.16 to 2.62)
2019	Belgium	2.37 (2.10 to 2.56)
2020	Belgium	2.26 (1.98 to 2.43)
2021	Belgium	2.25 (1.96 to 2.43)
1990	Cyprus	7.82 (6.01 to 10.06)
1991	Cyprus	8.07 (6.33 to 10.25)
1992	Cyprus	8.26 (6.56 to 10.34)
1993	Cyprus	8.15 (6.61 to 10.19)
1994	Cyprus	8.11 (6.51 to 10.29)
1995	Cyprus	7.83 (6.40 to 9.65)
1996	Cyprus	7.50 (6.02 to 9.13)
1997	Cyprus	7.44 (5.92 to 9.06)
1998	Cyprus	7.52 (6.10 to 9.09)
1999	Cyprus	7.43 (6.17 to 8.88)
2000	Cyprus	7.50 (6.31 to 8.87)
2001	Cyprus	7.54 (6.25 to 8.92)
2002	Cyprus	7.45 (6.19 to 8.88)
2003	Cyprus	7.13 (6.00 to 8.37)
2004	Cyprus	6.43 (5.48 to 7.43)
2005	Cyprus	6.05 (5.23 to 6.94)
2006	Cyprus	5.86 (5.05 to 6.69)
2007	Cyprus	5.61 (4.83 to 6.42)
2008	Cyprus	5.39 (4.68 to 6.12)
2009	Cyprus	5.23 (4.56 to 5.93)
2010	Cyprus	5.13 (4.50 to 5.83)
2011	Cyprus	4.92 (4.29 to 5.56)
2012	Cyprus	4.75 (4.11 to 5.39)
2013	Cyprus	4.61 (3.96 to 5.24)
2014	Cyprus	4.49 (3.83 to 5.12)
2015	Cyprus	4.43 (3.78 to 5.09)
2016	Cyprus	4.30 (3.64 to 4.96)
2017	Cyprus	4.29 (3.62 to 4.96)

2018	Cyprus	4.24 (3.52 to 4.97)
2019	Cyprus	4.26 (3.46 to 5.10)
2020	Cyprus	4.00 (3.11 to 4.95)
2021	Cyprus	3.96 (3.04 to 4.88)
1990	Denmark	6.71 (6.21 to 7.17)
1991	Denmark	6.71 (6.22 to 7.14)
1992	Denmark	6.87 (6.40 to 7.28)
1993	Denmark	7.13 (6.65 to 7.55)
1994	Denmark	7.62 (7.11 to 8.07)
1995	Denmark	7.61 (7.12 to 8.06)
1996	Denmark	7.65 (7.12 to 8.07)
1997	Denmark	7.73 (7.21 to 8.17)
1998	Denmark	7.77 (7.23 to 8.19)
1999	Denmark	7.57 (7.07 to 8.01)
2000	Denmark	7.48 (6.96 to 7.93)
2001	Denmark	7.53 (6.97 to 7.99)
2002	Denmark	7.43 (6.91 to 7.89)
2003	Denmark	7.27 (6.69 to 7.75)
2004	Denmark	7.36 (6.78 to 7.83)
2005	Denmark	7.19 (6.60 to 7.69)
2006	Denmark	7.02 (6.44 to 7.47)
2007	Denmark	6.78 (6.20 to 7.20)
2008	Denmark	6.38 (5.83 to 6.79)
2009	Denmark	5.98 (5.45 to 6.38)
2010	Denmark	5.70 (5.19 to 6.08)
2011	Denmark	5.63 (5.12 to 6.02)
2012	Denmark	5.55 (5.02 to 5.93)
2013	Denmark	5.50 (4.96 to 5.88)
2014	Denmark	5.12 (4.62 to 5.49)
2015	Denmark	4.98 (4.46 to 5.34)
2016	Denmark	4.90 (4.40 to 5.27)
2017	Denmark	4.77 (4.28 to 5.15)
2018	Denmark	4.84 (4.33 to 5.25)
2019	Denmark	4.72 (4.21 to 5.14)
2020	Denmark	4.63 (4.14 to 5.03)
2021	Denmark	4.61 (4.10 to 5.02)
1990	Finland	7.88 (7.35 to 8.36)
1991	Finland	7.63 (7.08 to 8.08)
1992	Finland	7.48 (6.98 to 7.93)
1993	Finland	7.32 (6.76 to 7.77)
1994	Finland	7.04 (6.48 to 7.42)
1995	Finland	7.02 (6.45 to 7.39)

1996	Finland	6.98 (6.38 to 7.41)
1997	Finland	6.99 (6.40 to 7.44)
1998	Finland	6.95 (6.38 to 7.41)
1999	Finland	6.74 (6.17 to 7.17)
2000	Finland	6.67 (6.09 to 7.07)
2001	Finland	6.57 (6.02 to 6.97)
2002	Finland	6.73 (6.17 to 7.15)
2003	Finland	6.57 (6.01 to 6.99)
2004	Finland	6.40 (5.84 to 6.80)
2005	Finland	6.11 (5.59 to 6.50)
2006	Finland	6.01 (5.45 to 6.38)
2007	Finland	6.08 (5.49 to 6.45)
2008	Finland	5.87 (5.25 to 6.23)
2009	Finland	5.67 (5.07 to 6.03)
2010	Finland	5.42 (4.85 to 5.79)
2011	Finland	5.14 (4.58 to 5.51)
2012	Finland	5.03 (4.45 to 5.39)
2013	Finland	4.86 (4.29 to 5.24)
2014	Finland	4.66 (4.10 to 5.03)
2015	Finland	4.35 (3.84 to 4.68)
2016	Finland	4.18 (3.71 to 4.51)
2017	Finland	4.03 (3.59 to 4.35)
2018	Finland	3.78 (3.37 to 4.07)
2019	Finland	3.72 (3.31 to 4.01)
2020	Finland	3.66 (3.26 to 3.95)
2021	Finland	3.63 (3.20 to 3.93)
1990	France	3.65 (3.41 to 3.85)
1991	France	3.70 (3.44 to 3.91)
1992	France	3.75 (3.49 to 3.97)
1993	France	3.78 (3.51 to 4.00)
1994	France	3.73 (3.44 to 3.96)
1995	France	3.84 (3.53 to 4.07)
1996	France	3.93 (3.61 to 4.18)
1997	France	3.82 (3.50 to 4.06)
1998	France	3.73 (3.43 to 3.95)
1999	France	3.71 (3.42 to 3.93)
2000	France	3.70 (3.42 to 3.93)
2001	France	3.59 (3.30 to 3.81)
2002	France	3.51 (3.23 to 3.74)
2003	France	3.39 (3.11 to 3.60)
2004	France	3.23 (2.96 to 3.42)
2005	France	3.17 (2.90 to 3.35)

2006	France	3.07 (2.79 to 3.25)
2007	France	2.95 (2.68 to 3.12)
2008	France	2.85 (2.60 to 3.03)
2009	France	2.73 (2.49 to 2.90)
2010	France	2.58 (2.35 to 2.75)
2011	France	2.38 (2.17 to 2.55)
2012	France	2.28 (2.08 to 2.44)
2013	France	2.18 (1.98 to 2.33)
2014	France	2.10 (1.89 to 2.24)
2015	France	2.08 (1.88 to 2.22)
2016	France	2.09 (1.88 to 2.23)
2017	France	2.07 (1.87 to 2.21)
2018	France	2.01 (1.81 to 2.16)
2019	France	1.98 (1.76 to 2.13)
2020	France	1.96 (1.73 to 2.11)
2021	France	1.89 (1.67 to 2.04)
1990	Germany	3.32 (3.07 to 3.56)
1991	Germany	3.32 (3.06 to 3.55)
1992	Germany	3.25 (3.00 to 3.47)
1993	Germany	3.23 (2.98 to 3.44)
1994	Germany	3.16 (2.93 to 3.37)
1995	Germany	3.12 (2.89 to 3.32)
1996	Germany	3.12 (2.88 to 3.32)
1997	Germany	3.11 (2.87 to 3.32)
1998	Germany	3.17 (2.91 to 3.39)
1999	Germany	3.07 (2.82 to 3.29)
2000	Germany	2.94 (2.70 to 3.15)
2001	Germany	2.81 (2.57 to 3.00)
2002	Germany	2.78 (2.55 to 2.96)
2003	Germany	2.74 (2.50 to 2.92)
2004	Germany	2.66 (2.41 to 2.84)
2005	Germany	2.58 (2.33 to 2.77)
2006	Germany	2.57 (2.32 to 2.75)
2007	Germany	2.56 (2.31 to 2.74)
2008	Germany	2.55 (2.32 to 2.73)
2009	Germany	2.46 (2.23 to 2.62)
2010	Germany	2.38 (2.15 to 2.54)
2011	Germany	2.35 (2.13 to 2.50)
2012	Germany	2.31 (2.10 to 2.46)
2013	Germany	2.30 (2.09 to 2.46)
2014	Germany	2.28 (2.07 to 2.44)
2015	Germany	2.24 (2.04 to 2.38)

2016	Germany	2.25 (2.04 to 2.40)
2017	Germany	2.24 (2.03 to 2.39)
2018	Germany	2.32 (2.09 to 2.47)
2019	Germany	2.33 (2.10 to 2.49)
2020	Germany	2.30 (2.06 to 2.45)
2021	Germany	2.25 (2.02 to 2.41)
1990	Greece	3.81 (3.55 to 4.03)
1991	Greece	3.92 (3.66 to 4.14)
1992	Greece	4.00 (3.75 to 4.23)
1993	Greece	4.02 (3.78 to 4.25)
1994	Greece	4.24 (3.96 to 4.47)
1995	Greece	4.47 (4.20 to 4.72)
1996	Greece	4.53 (4.25 to 4.76)
1997	Greece	4.53 (4.27 to 4.76)
1998	Greece	4.73 (4.46 to 4.97)
1999	Greece	4.69 (4.37 to 4.92)
2000	Greece	4.67 (4.35 to 4.91)
2001	Greece	4.69 (4.36 to 4.94)
2002	Greece	4.65 (4.34 to 4.88)
2003	Greece	4.64 (4.34 to 4.88)
2004	Greece	4.68 (4.35 to 4.92)
2005	Greece	4.58 (4.26 to 4.82)
2006	Greece	4.51 (4.19 to 4.75)
2007	Greece	4.46 (4.13 to 4.71)
2008	Greece	4.15 (3.85 to 4.40)
2009	Greece	4.06 (3.76 to 4.31)
2010	Greece	3.94 (3.64 to 4.19)
2011	Greece	3.79 (3.50 to 4.04)
2012	Greece	3.70 (3.41 to 3.96)
2013	Greece	3.60 (3.32 to 3.86)
2014	Greece	3.60 (3.32 to 3.83)
2015	Greece	3.86 (3.56 to 4.11)
2016	Greece	4.04 (3.72 to 4.31)
2017	Greece	3.97 (3.65 to 4.21)
2018	Greece	3.91 (3.59 to 4.15)
2019	Greece	3.93 (3.62 to 4.17)
2020	Greece	3.99 (3.67 to 4.25)
2021	Greece	3.99 (3.65 to 4.26)
1990	Iceland	4.34 (3.91 to 4.65)
1991	Iceland	4.47 (4.01 to 4.80)
1992	Iceland	4.60 (4.14 to 4.95)
1993	Iceland	4.54 (4.08 to 4.88)

1994	Iceland	4.91 (4.44 to 5.28)
1995	Iceland	5.05 (4.57 to 5.45)
1996	Iceland	5.12 (4.64 to 5.52)
1997	Iceland	5.09 (4.60 to 5.48)
1998	Iceland	4.98 (4.54 to 5.34)
1999	Iceland	4.76 (4.34 to 5.12)
2000	Iceland	4.61 (4.21 to 4.97)
2001	Iceland	4.77 (4.32 to 5.12)
2002	Iceland	4.69 (4.25 to 5.04)
2003	Iceland	4.46 (4.05 to 4.81)
2004	Iceland	4.43 (4.01 to 4.78)
2005	Iceland	4.29 (3.89 to 4.63)
2006	Iceland	4.21 (3.82 to 4.54)
2007	Iceland	4.03 (3.66 to 4.34)
2008	Iceland	3.90 (3.53 to 4.20)
2009	Iceland	3.81 (3.43 to 4.11)
2010	Iceland	3.76 (3.38 to 4.08)
2011	Iceland	3.71 (3.31 to 4.03)
2012	Iceland	3.51 (3.12 to 3.83)
2013	Iceland	3.41 (3.03 to 3.73)
2014	Iceland	3.33 (2.97 to 3.62)
2015	Iceland	3.18 (2.83 to 3.45)
2016	Iceland	3.11 (2.76 to 3.39)
2017	Iceland	2.95 (2.60 to 3.23)
2018	Iceland	2.86 (2.49 to 3.16)
2019	Iceland	2.77 (2.37 to 3.08)
2020	Iceland	2.61 (2.24 to 2.90)
2021	Iceland	2.61 (2.23 to 2.92)
1990	Ireland	6.36 (5.94 to 6.78)
1991	Ireland	6.40 (5.96 to 6.79)
1992	Ireland	6.86 (6.40 to 7.29)
1993	Ireland	6.97 (6.50 to 7.39)
1994	Ireland	6.74 (6.32 to 7.16)
1995	Ireland	6.95 (6.47 to 7.37)
1996	Ireland	6.90 (6.46 to 7.30)
1997	Ireland	6.99 (6.54 to 7.40)
1998	Ireland	6.93 (6.49 to 7.33)
1999	Ireland	6.95 (6.46 to 7.35)
2000	Ireland	6.90 (6.41 to 7.30)
2001	Ireland	6.71 (6.20 to 7.12)
2002	Ireland	6.57 (6.04 to 6.98)
2003	Ireland	6.15 (5.64 to 6.52)

2004	Ireland	5.81 (5.33 to 6.17)
2005	Ireland	5.57 (5.11 to 5.91)
2006	Ireland	5.44 (4.97 to 5.81)
2007	Ireland	5.38 (4.93 to 5.74)
2008	Ireland	5.00 (4.55 to 5.33)
2009	Ireland	4.66 (4.23 to 4.98)
2010	Ireland	4.42 (3.99 to 4.73)
2011	Ireland	4.42 (3.99 to 4.74)
2012	Ireland	4.27 (3.86 to 4.58)
2013	Ireland	4.13 (3.73 to 4.44)
2014	Ireland	3.85 (3.44 to 4.13)
2015	Ireland	3.67 (3.30 to 3.97)
2016	Ireland	3.69 (3.29 to 3.99)
2017	Ireland	3.54 (3.14 to 3.84)
2018	Ireland	3.50 (3.07 to 3.81)
2019	Ireland	3.38 (2.93 to 3.69)
2020	Ireland	3.16 (2.72 to 3.49)
2021	Ireland	2.93 (2.47 to 3.25)
1990	Israel	2.34 (2.15 to 2.50)
1991	Israel	2.39 (2.20 to 2.55)
1992	Israel	2.44 (2.26 to 2.60)
1993	Israel	2.40 (2.22 to 2.54)
1994	Israel	2.41 (2.23 to 2.55)
1995	Israel	2.40 (2.22 to 2.54)
1996	Israel	2.34 (2.15 to 2.48)
1997	Israel	2.42 (2.22 to 2.56)
1998	Israel	2.42 (2.22 to 2.56)
1999	Israel	2.43 (2.23 to 2.58)
2000	Israel	2.24 (2.05 to 2.39)
2001	Israel	2.13 (1.95 to 2.26)
2002	Israel	2.22 (2.02 to 2.36)
2003	Israel	2.10 (1.91 to 2.23)
2004	Israel	2.03 (1.85 to 2.15)
2005	Israel	1.93 (1.76 to 2.05)
2006	Israel	1.84 (1.67 to 1.96)
2007	Israel	1.81 (1.64 to 1.93)
2008	Israel	1.71 (1.54 to 1.83)
2009	Israel	1.70 (1.53 to 1.83)
2010	Israel	1.65 (1.48 to 1.78)
2011	Israel	1.53 (1.38 to 1.66)
2012	Israel	1.51 (1.35 to 1.62)
2013	Israel	1.53 (1.37 to 1.64)

2014	Israel	1.47 (1.31 to 1.58)
2015	Israel	1.50 (1.34 to 1.61)
2016	Israel	1.49 (1.34 to 1.60)
2017	Israel	1.44 (1.28 to 1.55)
2018	Israel	1.37 (1.22 to 1.47)
2019	Israel	1.36 (1.21 to 1.47)
2020	Israel	1.31 (1.17 to 1.42)
2021	Israel	1.28 (1.13 to 1.40)
1990	Italy	3.10 (2.91 to 3.21)
1991	Italy	3.18 (2.98 to 3.30)
1992	Italy	3.27 (3.06 to 3.39)
1993	Italy	3.38 (3.15 to 3.52)
1994	Italy	3.56 (3.32 to 3.70)
1995	Italy	3.75 (3.49 to 3.90)
1996	Italy	3.74 (3.48 to 3.90)
1997	Italy	3.68 (3.43 to 3.84)
1998	Italy	3.68 (3.43 to 3.83)
1999	Italy	3.59 (3.34 to 3.74)
2000	Italy	3.47 (3.21 to 3.62)
2001	Italy	3.41 (3.14 to 3.56)
2002	Italy	3.40 (3.13 to 3.55)
2003	Italy	3.52 (3.23 to 3.68)
2004	Italy	3.41 (3.11 to 3.59)
2005	Italy	3.32 (3.02 to 3.49)
2006	Italy	3.15 (2.84 to 3.31)
2007	Italy	3.12 (2.81 to 3.27)
2008	Italy	2.94 (2.66 to 3.09)
2009	Italy	2.82 (2.55 to 2.96)
2010	Italy	2.72 (2.46 to 2.86)
2011	Italy	2.64 (2.38 to 2.77)
2012	Italy	2.58 (2.33 to 2.71)
2013	Italy	2.44 (2.20 to 2.56)
2014	Italy	2.41 (2.16 to 2.54)
2015	Italy	2.40 (2.16 to 2.53)
2016	Italy	2.34 (2.11 to 2.48)
2017	Italy	2.28 (2.06 to 2.41)
2018	Italy	2.26 (2.02 to 2.39)
2019	Italy	2.20 (1.96 to 2.34)
2020	Italy	2.21 (1.98 to 2.36)
2021	Italy	2.18 (1.95 to 2.34)
1990	Luxembourg	4.33 (4.06 to 4.64)
1991	Luxembourg	4.66 (4.37 to 4.98)

1992	Luxembourg	4.74 (4.42 to 5.06)
1993	Luxembourg	4.68 (4.39 to 5.01)
1994	Luxembourg	4.77 (4.46 to 5.08)
1995	Luxembourg	4.76 (4.44 to 5.07)
1996	Luxembourg	4.77 (4.46 to 5.07)
1997	Luxembourg	4.67 (4.37 to 4.98)
1998	Luxembourg	4.57 (4.27 to 4.87)
1999	Luxembourg	4.39 (4.07 to 4.70)
2000	Luxembourg	4.24 (3.92 to 4.54)
2001	Luxembourg	4.27 (3.96 to 4.56)
2002	Luxembourg	4.15 (3.86 to 4.43)
2003	Luxembourg	4.14 (3.84 to 4.41)
2004	Luxembourg	3.91 (3.59 to 4.18)
2005	Luxembourg	3.74 (3.43 to 4.01)
2006	Luxembourg	3.72 (3.44 to 3.97)
2007	Luxembourg	3.55 (3.26 to 3.77)
2008	Luxembourg	3.35 (3.08 to 3.57)
2009	Luxembourg	3.18 (2.92 to 3.40)
2010	Luxembourg	2.96 (2.72 to 3.18)
2011	Luxembourg	2.99 (2.74 to 3.22)
2012	Luxembourg	2.90 (2.66 to 3.14)
2013	Luxembourg	2.65 (2.42 to 2.86)
2014	Luxembourg	2.65 (2.42 to 2.87)
2015	Luxembourg	2.74 (2.50 to 2.97)
2016	Luxembourg	2.60 (2.37 to 2.79)
2017	Luxembourg	2.49 (2.26 to 2.68)
2018	Luxembourg	2.49 (2.25 to 2.68)
2019	Luxembourg	2.40 (2.16 to 2.61)
2020	Luxembourg	2.27 (2.02 to 2.48)
2021	Luxembourg	2.23 (1.97 to 2.46)
1990	Malta	2.60 (2.41 to 2.80)
1991	Malta	2.69 (2.50 to 2.89)
1992	Malta	2.74 (2.55 to 2.94)
1993	Malta	2.73 (2.52 to 2.92)
1994	Malta	2.73 (2.52 to 2.94)
1995	Malta	2.74 (2.53 to 2.95)
1996	Malta	2.71 (2.51 to 2.92)
1997	Malta	2.88 (2.66 to 3.10)
1998	Malta	2.78 (2.58 to 3.00)
1999	Malta	2.75 (2.55 to 2.98)
2000	Malta	2.81 (2.60 to 3.05)
2001	Malta	2.71 (2.50 to 2.94)

2002	Malta	2.52 (2.31 to 2.72)
2003	Malta	2.46 (2.26 to 2.67)
2004	Malta	2.47 (2.27 to 2.67)
2005	Malta	2.47 (2.26 to 2.68)
2006	Malta	2.40 (2.19 to 2.59)
2007	Malta	2.30 (2.10 to 2.48)
2008	Malta	2.16 (1.98 to 2.34)
2009	Malta	1.95 (1.77 to 2.11)
2010	Malta	1.86 (1.69 to 2.01)
2011	Malta	1.80 (1.64 to 1.95)
2012	Malta	1.71 (1.57 to 1.86)
2013	Malta	1.65 (1.51 to 1.79)
2014	Malta	1.60 (1.45 to 1.73)
2015	Malta	1.51 (1.37 to 1.63)
2016	Malta	1.46 (1.32 to 1.59)
2017	Malta	1.39 (1.25 to 1.53)
2018	Malta	1.40 (1.26 to 1.54)
2019	Malta	1.39 (1.23 to 1.54)
2020	Malta	1.26 (1.11 to 1.40)
2021	Malta	1.32 (1.17 to 1.47)
1990	Monaco	5.66 (4.29 to 7.02)
1991	Monaco	5.64 (4.26 to 7.03)
1992	Monaco	5.62 (4.23 to 6.98)
1993	Monaco	5.63 (4.20 to 7.03)
1994	Monaco	5.67 (4.18 to 7.01)
1995	Monaco	5.71 (4.22 to 7.06)
1996	Monaco	5.76 (4.34 to 7.13)
1997	Monaco	5.84 (4.40 to 7.15)
1998	Monaco	5.92 (4.43 to 7.25)
1999	Monaco	6.00 (4.51 to 7.32)
2000	Monaco	6.10 (4.66 to 7.35)
2001	Monaco	6.16 (4.79 to 7.47)
2002	Monaco	6.15 (4.83 to 7.37)
2003	Monaco	6.14 (4.90 to 7.35)
2004	Monaco	6.10 (4.91 to 7.27)
2005	Monaco	6.08 (4.93 to 7.19)
2006	Monaco	6.04 (4.98 to 7.14)
2007	Monaco	5.95 (4.94 to 7.02)
2008	Monaco	5.87 (4.84 to 6.94)
2009	Monaco	5.85 (4.89 to 6.96)
2010	Monaco	5.85 (4.95 to 6.95)
2011	Monaco	5.78 (4.84 to 6.86)

2012	Monaco	5.74 (4.81 to 6.79)
2013	Monaco	5.69 (4.73 to 6.73)
2014	Monaco	5.62 (4.64 to 6.71)
2015	Monaco	5.54 (4.53 to 6.73)
2016	Monaco	5.46 (4.38 to 6.79)
2017	Monaco	5.38 (4.31 to 6.80)
2018	Monaco	5.31 (4.18 to 6.82)
2019	Monaco	5.23 (4.04 to 6.84)
2020	Monaco	5.17 (3.90 to 6.90)
2021	Monaco	5.15 (3.88 to 6.95)
1990	Netherlands	7.44 (6.89 to 7.96)
1991	Netherlands	7.60 (7.04 to 8.10)
1992	Netherlands	7.58 (7.00 to 8.07)
1993	Netherlands	7.69 (7.08 to 8.19)
1994	Netherlands	7.73 (7.09 to 8.20)
1995	Netherlands	7.98 (7.33 to 8.45)
1996	Netherlands	7.87 (7.26 to 8.36)
1997	Netherlands	7.82 (7.20 to 8.32)
1998	Netherlands	7.74 (7.12 to 8.22)
1999	Netherlands	7.65 (7.02 to 8.13)
2000	Netherlands	7.68 (7.05 to 8.19)
2001	Netherlands	7.68 (7.04 to 8.18)
2002	Netherlands	7.69 (7.02 to 8.18)
2003	Netherlands	7.32 (6.65 to 7.80)
2004	Netherlands	7.07 (6.42 to 7.54)
2005	Netherlands	6.83 (6.19 to 7.30)
2006	Netherlands	6.49 (5.90 to 6.95)
2007	Netherlands	6.10 (5.54 to 6.53)
2008	Netherlands	5.82 (5.27 to 6.24)
2009	Netherlands	5.55 (5.00 to 5.96)
2010	Netherlands	5.42 (4.86 to 5.80)
2011	Netherlands	5.20 (4.66 to 5.57)
2012	Netherlands	4.90 (4.38 to 5.27)
2013	Netherlands	4.43 (3.94 to 4.79)
2014	Netherlands	4.20 (3.71 to 4.54)
2015	Netherlands	4.12 (3.66 to 4.47)
2016	Netherlands	4.07 (3.60 to 4.41)
2017	Netherlands	3.88 (3.42 to 4.21)
2018	Netherlands	3.84 (3.37 to 4.17)
2019	Netherlands	3.77 (3.30 to 4.10)
2020	Netherlands	3.75 (3.26 to 4.08)
2021	Netherlands	3.74 (3.24 to 4.08)

1990	Norway	7.58 (7.16 to 7.90)
1991	Norway	7.44 (6.99 to 7.72)
1992	Norway	7.68 (7.20 to 7.99)
1993	Norway	7.99 (7.49 to 8.31)
1994	Norway	7.92 (7.40 to 8.25)
1995	Norway	8.05 (7.49 to 8.39)
1996	Norway	8.20 (7.59 to 8.55)
1997	Norway	8.07 (7.46 to 8.44)
1998	Norway	8.27 (7.71 to 8.65)
1999	Norway	8.25 (7.65 to 8.65)
2000	Norway	8.09 (7.47 to 8.51)
2001	Norway	7.95 (7.31 to 8.36)
2002	Norway	7.79 (7.19 to 8.19)
2003	Norway	7.68 (7.05 to 8.06)
2004	Norway	7.62 (6.97 to 8.03)
2005	Norway	7.24 (6.62 to 7.61)
2006	Norway	6.89 (6.27 to 7.24)
2007	Norway	6.82 (6.18 to 7.15)
2008	Norway	6.25 (5.66 to 6.58)
2009	Norway	6.24 (5.63 to 6.55)
2010	Norway	6.09 (5.51 to 6.43)
2011	Norway	5.87 (5.33 to 6.20)
2012	Norway	5.66 (5.13 to 5.97)
2013	Norway	5.43 (4.89 to 5.75)
2014	Norway	4.96 (4.43 to 5.25)
2015	Norway	4.98 (4.45 to 5.27)
2016	Norway	4.71 (4.22 to 4.98)
2017	Norway	4.70 (4.19 to 4.98)
2018	Norway	4.74 (4.24 to 5.03)
2019	Norway	4.84 (4.29 to 5.13)
2020	Norway	4.69 (4.07 to 5.00)
2021	Norway	4.75 (4.15 to 5.08)
1990	Portugal	1.75 (1.64 to 1.86)
1991	Portugal	1.83 (1.72 to 1.94)
1992	Portugal	1.80 (1.69 to 1.91)
1993	Portugal	1.86 (1.75 to 1.96)
1994	Portugal	1.81 (1.70 to 1.92)
1995	Portugal	1.95 (1.82 to 2.05)
1996	Portugal	1.95 (1.83 to 2.06)
1997	Portugal	1.95 (1.83 to 2.06)
1998	Portugal	1.94 (1.82 to 2.05)
1999	Portugal	1.85 (1.74 to 1.96)

2000	Portugal	1.89 (1.76 to 2.00)
2001	Portugal	1.89 (1.75 to 2.00)
2002	Portugal	1.82 (1.68 to 1.93)
2003	Portugal	1.81 (1.68 to 1.92)
2004	Portugal	1.71 (1.58 to 1.82)
2005	Portugal	1.50 (1.39 to 1.61)
2006	Portugal	1.52 (1.42 to 1.62)
2007	Portugal	1.61 (1.50 to 1.72)
2008	Portugal	1.66 (1.54 to 1.77)
2009	Portugal	1.66 (1.54 to 1.77)
2010	Portugal	1.62 (1.49 to 1.73)
2011	Portugal	1.57 (1.45 to 1.67)
2012	Portugal	1.58 (1.46 to 1.68)
2013	Portugal	1.50 (1.37 to 1.59)
2014	Portugal	1.49 (1.37 to 1.59)
2015	Portugal	1.53 (1.41 to 1.63)
2016	Portugal	1.52 (1.40 to 1.62)
2017	Portugal	1.54 (1.41 to 1.64)
2018	Portugal	1.58 (1.43 to 1.68)
2019	Portugal	1.56 (1.42 to 1.67)
2020	Portugal	1.50 (1.37 to 1.62)
2021	Portugal	1.51 (1.37 to 1.62)
1990	San Marino	3.09 (2.49 to 3.76)
1991	San Marino	3.09 (2.50 to 3.71)
1992	San Marino	3.09 (2.53 to 3.72)
1993	San Marino	3.09 (2.55 to 3.74)
1994	San Marino	3.10 (2.55 to 3.73)
1995	San Marino	3.07 (2.52 to 3.62)
1996	San Marino	3.05 (2.50 to 3.56)
1997	San Marino	3.00 (2.47 to 3.53)
1998	San Marino	2.98 (2.49 to 3.49)
1999	San Marino	2.94 (2.45 to 3.45)
2000	San Marino	2.91 (2.41 to 3.51)
2001	San Marino	2.88 (2.37 to 3.50)
2002	San Marino	2.84 (2.33 to 3.47)
2003	San Marino	2.80 (2.26 to 3.47)
2004	San Marino	2.73 (2.16 to 3.43)
2005	San Marino	2.72 (2.12 to 3.40)
2006	San Marino	2.73 (2.14 to 3.43)
2007	San Marino	2.72 (2.08 to 3.48)
2008	San Marino	2.73 (2.07 to 3.52)
2009	San Marino	2.73 (2.03 to 3.60)

2010	San Marino	2.75 (2.05 to 3.62)
2011	San Marino	2.77 (2.07 to 3.68)
2012	San Marino	2.78 (2.05 to 3.78)
2013	San Marino	2.80 (2.09 to 3.77)
2014	San Marino	2.81 (2.06 to 3.82)
2015	San Marino	2.83 (2.07 to 3.80)
2016	San Marino	2.84 (2.06 to 3.82)
2017	San Marino	2.86 (2.06 to 3.77)
2018	San Marino	2.85 (2.08 to 3.81)
2019	San Marino	2.84 (2.06 to 3.85)
2020	San Marino	1.77 (1.19 to 2.59)
2021	San Marino	1.67 (1.06 to 2.59)
1990	Spain	2.54 (2.37 to 2.68)
1991	Spain	2.76 (2.57 to 2.92)
1992	Spain	2.78 (2.59 to 2.95)
1993	Spain	2.86 (2.67 to 3.02)
1994	Spain	2.97 (2.76 to 3.14)
1995	Spain	3.08 (2.88 to 3.26)
1996	Spain	3.10 (2.91 to 3.26)
1997	Spain	3.08 (2.88 to 3.23)
1998	Spain	3.11 (2.91 to 3.27)
1999	Spain	3.15 (2.95 to 3.32)
2000	Spain	3.14 (2.95 to 3.32)
2001	Spain	3.07 (2.87 to 3.24)
2002	Spain	3.08 (2.88 to 3.26)
2003	Spain	3.04 (2.85 to 3.21)
2004	Spain	2.89 (2.69 to 3.05)
2005	Spain	2.78 (2.59 to 2.95)
2006	Spain	2.73 (2.54 to 2.89)
2007	Spain	2.65 (2.46 to 2.81)
2008	Spain	2.59 (2.39 to 2.75)
2009	Spain	2.51 (2.33 to 2.67)
2010	Spain	2.40 (2.20 to 2.55)
2011	Spain	2.30 (2.09 to 2.45)
2012	Spain	2.26 (2.04 to 2.40)
2013	Spain	2.18 (1.98 to 2.32)
2014	Spain	2.17 (1.96 to 2.31)
2015	Spain	2.19 (1.99 to 2.33)
2016	Spain	2.19 (2.00 to 2.34)
2017	Spain	2.22 (2.04 to 2.37)
2018	Spain	2.22 (2.02 to 2.37)
2019	Spain	2.17 (1.97 to 2.32)

2020	Spain	2.09 (1.89 to 2.25)
2021	Spain	2.08 (1.87 to 2.23)
1990	Sweden	7.41 (6.91 to 7.79)
1991	Sweden	7.56 (7.07 to 7.94)
1992	Sweden	7.58 (7.07 to 7.97)
1993	Sweden	7.77 (7.27 to 8.17)
1994	Sweden	7.71 (7.19 to 8.13)
1995	Sweden	7.66 (7.15 to 8.08)
1996	Sweden	7.85 (7.34 to 8.26)
1997	Sweden	7.90 (7.39 to 8.33)
1998	Sweden	7.97 (7.44 to 8.41)
1999	Sweden	7.87 (7.31 to 8.31)
2000	Sweden	7.71 (7.17 to 8.14)
2001	Sweden	7.64 (7.05 to 8.06)
2002	Sweden	7.38 (6.82 to 7.82)
2003	Sweden	7.25 (6.68 to 7.66)
2004	Sweden	7.24 (6.68 to 7.65)
2005	Sweden	6.77 (6.22 to 7.15)
2006	Sweden	6.55 (5.98 to 6.92)
2007	Sweden	5.90 (5.38 to 6.24)
2008	Sweden	5.63 (5.12 to 5.96)
2009	Sweden	5.54 (5.03 to 5.87)
2010	Sweden	5.30 (4.85 to 5.64)
2011	Sweden	5.10 (4.68 to 5.42)
2012	Sweden	4.87 (4.46 to 5.18)
2013	Sweden	4.77 (4.36 to 5.08)
2014	Sweden	4.48 (4.05 to 4.78)
2015	Sweden	4.38 (3.94 to 4.69)
2016	Sweden	4.40 (3.96 to 4.71)
2017	Sweden	4.42 (3.99 to 4.72)
2018	Sweden	4.32 (3.88 to 4.63)
2019	Sweden	4.16 (3.72 to 4.47)
2020	Sweden	4.02 (3.58 to 4.34)
2021	Sweden	3.88 (3.38 to 4.33)
1990	Switzerland	4.87 (4.43 to 5.26)
1991	Switzerland	4.82 (4.40 to 5.17)
1992	Switzerland	4.69 (4.26 to 5.04)
1993	Switzerland	4.54 (4.14 to 4.87)
1994	Switzerland	4.50 (4.12 to 4.81)
1995	Switzerland	4.67 (4.25 to 5.00)
1996	Switzerland	4.64 (4.22 to 4.96)
1997	Switzerland	4.70 (4.30 to 5.00)

1998	Switzerland	4.55 (4.17 to 4.84)
1999	Switzerland	4.30 (3.92 to 4.57)
2000	Switzerland	4.37 (3.98 to 4.66)
2001	Switzerland	4.29 (3.90 to 4.56)
2002	Switzerland	4.19 (3.80 to 4.47)
2003	Switzerland	3.94 (3.58 to 4.22)
2004	Switzerland	3.71 (3.35 to 3.99)
2005	Switzerland	3.78 (3.40 to 4.09)
2006	Switzerland	3.69 (3.29 to 3.99)
2007	Switzerland	3.60 (3.22 to 3.87)
2008	Switzerland	3.48 (3.12 to 3.75)
2009	Switzerland	3.37 (3.00 to 3.63)
2010	Switzerland	3.26 (2.90 to 3.50)
2011	Switzerland	3.08 (2.73 to 3.32)
2012	Switzerland	3.04 (2.69 to 3.28)
2013	Switzerland	3.01 (2.66 to 3.25)
2014	Switzerland	2.92 (2.58 to 3.16)
2015	Switzerland	2.75 (2.43 to 2.97)
2016	Switzerland	2.72 (2.39 to 2.94)
2017	Switzerland	2.57 (2.26 to 2.77)
2018	Switzerland	2.53 (2.21 to 2.74)
2019	Switzerland	2.40 (2.09 to 2.61)
2020	Switzerland	2.36 (2.04 to 2.59)
2021	Switzerland	2.28 (1.96 to 2.51)
1990	United Kingdom of Great Britain and Northern Ireland	9.72 (9.16 to 10.00)
1991	United Kingdom of Great Britain and Northern Ireland	9.84 (9.31 to 10.12)
1992	United Kingdom of Great Britain and Northern Ireland	10.04 (9.47 to 10.34)
1993	United Kingdom of Great Britain and Northern Ireland	10.19 (9.65 to 10.47)
1994	United Kingdom of Great Britain and Northern Ireland	10.17 (9.57 to 10.48)
1995	United Kingdom of Great Britain and Northern Ireland	10.36 (9.78 to 10.66)
1996	United Kingdom of Great Britain and Northern Ireland	10.42 (9.86 to 10.73)
1997	United Kingdom of Great Britain and Northern Ireland	10.18 (9.61 to 10.49)
1998	United Kingdom of Great Britain and Northern Ireland	10.17 (9.57 to 10.49)
1999	United Kingdom of Great Britain and Northern Ireland	10.04 (9.45 to 10.35)
2000	United Kingdom of Great Britain and Northern Ireland	9.89 (9.27 to 10.21)
2001	United Kingdom of Great Britain and Northern Ireland	9.56 (8.90 to 9.89)
2002	United Kingdom of Great Britain and Northern Ireland	9.33 (8.71 to 9.65)
2003	United Kingdom of Great Britain and Northern Ireland	9.06 (8.42 to 9.37)
2004	United Kingdom of Great Britain and Northern Ireland	8.67 (8.02 to 9.01)
2005	United Kingdom of Great Britain and Northern Ireland	8.19 (7.56 to 8.49)
2006	United Kingdom of Great Britain and Northern Ireland	7.83 (7.21 to 8.14)
2007	United Kingdom of Great Britain and Northern Ireland	7.48 (6.88 to 7.78)

2008	United Kingdom of Great Britain and Northern Ireland	7.06 (6.48 to 7.34)
2009	United Kingdom of Great Britain and Northern Ireland	6.62 (6.03 to 6.91)
2010	United Kingdom of Great Britain and Northern Ireland	6.26 (5.72 to 6.53)
2011	United Kingdom of Great Britain and Northern Ireland	5.83 (5.29 to 6.10)
2012	United Kingdom of Great Britain and Northern Ireland	5.64 (5.11 to 5.90)
2013	United Kingdom of Great Britain and Northern Ireland	5.38 (4.88 to 5.62)
2014	United Kingdom of Great Britain and Northern Ireland	5.15 (4.65 to 5.39)
2015	United Kingdom of Great Britain and Northern Ireland	4.94 (4.45 to 5.17)
2016	United Kingdom of Great Britain and Northern Ireland	4.76 (4.29 to 4.98)
2017	United Kingdom of Great Britain and Northern Ireland	4.55 (4.11 to 4.76)
2018	United Kingdom of Great Britain and Northern Ireland	4.38 (3.94 to 4.58)
2019	United Kingdom of Great Britain and Northern Ireland	4.19 (3.76 to 4.38)
2020	United Kingdom of Great Britain and Northern Ireland	4.02 (3.60 to 4.23)
2021	United Kingdom of Great Britain and Northern Ireland	4.03 (3.61 to 4.25)

ASDR, age-standardized death rates.

Fig 3C. ASDR in Central Europe from 1990 to 2021

Year	Location	ASDR per 100,000
1990	Albania	0.91 (0.76 to 1.08)
1991	Albania	0.94 (0.78 to 1.11)
1992	Albania	0.91 (0.75 to 1.08)
1993	Albania	0.88 (0.73 to 1.06)
1994	Albania	0.84 (0.68 to 1.01)
1995	Albania	0.87 (0.71 to 1.03)
1996	Albania	0.90 (0.76 to 1.05)
1997	Albania	0.92 (0.78 to 1.06)
1998	Albania	0.92 (0.78 to 1.08)
1999	Albania	0.94 (0.79 to 1.10)
2000	Albania	0.96 (0.82 to 1.13)
2001	Albania	0.97 (0.80 to 1.16)
2002	Albania	1.00 (0.83 to 1.20)
2003	Albania	1.03 (0.87 to 1.24)
2004	Albania	1.05 (0.87 to 1.25)
2005	Albania	1.03 (0.86 to 1.23)
2006	Albania	1.00 (0.82 to 1.22)
2007	Albania	0.98 (0.77 to 1.22)
2008	Albania	0.97 (0.76 to 1.21)
2009	Albania	0.96 (0.75 to 1.21)
2010	Albania	0.98 (0.75 to 1.28)
2011	Albania	1.00 (0.75 to 1.31)
2012	Albania	1.02 (0.77 to 1.33)
2013	Albania	1.04 (0.78 to 1.36)
2014	Albania	1.05 (0.79 to 1.42)
2015	Albania	1.06 (0.78 to 1.42)
2016	Albania	1.07 (0.79 to 1.45)
2017	Albania	1.09 (0.79 to 1.46)
2018	Albania	1.10 (0.80 to 1.49)
2019	Albania	1.14 (0.80 to 1.58)
2020	Albania	1.06 (0.76 to 1.43)
2021	Albania	1.09 (0.76 to 1.50)
1990	Bosnia and Herzegovina	2.12 (1.55 to 2.89)
1991	Bosnia and Herzegovina	2.22 (1.59 to 3.00)
1992	Bosnia and Herzegovina	2.21 (1.58 to 3.11)
1993	Bosnia and Herzegovina	2.24 (1.59 to 3.15)
1994	Bosnia and Herzegovina	2.24 (1.54 to 3.16)
1995	Bosnia and Herzegovina	2.23 (1.49 to 3.21)
1996	Bosnia and Herzegovina	2.24 (1.56 to 3.08)
1997	Bosnia and Herzegovina	2.29 (1.63 to 3.14)

1998	Bosnia and Herzegovina	2.28 (1.70 to 3.09)
1999	Bosnia and Herzegovina	2.29 (1.73 to 3.08)
2000	Bosnia and Herzegovina	2.34 (1.74 to 3.15)
2001	Bosnia and Herzegovina	2.37 (1.74 to 3.16)
2002	Bosnia and Herzegovina	2.44 (1.80 to 3.23)
2003	Bosnia and Herzegovina	2.51 (1.93 to 3.23)
2004	Bosnia and Herzegovina	2.55 (1.98 to 3.23)
2005	Bosnia and Herzegovina	2.68 (2.08 to 3.47)
2006	Bosnia and Herzegovina	2.73 (2.14 to 3.50)
2007	Bosnia and Herzegovina	2.91 (2.29 to 3.69)
2008	Bosnia and Herzegovina	2.86 (2.26 to 3.60)
2009	Bosnia and Herzegovina	2.87 (2.29 to 3.58)
2010	Bosnia and Herzegovina	2.84 (2.27 to 3.52)
2011	Bosnia and Herzegovina	2.87 (2.34 to 3.49)
2012	Bosnia and Herzegovina	2.83 (2.32 to 3.45)
2013	Bosnia and Herzegovina	2.88 (2.37 to 3.53)
2014	Bosnia and Herzegovina	2.90 (2.37 to 3.56)
2015	Bosnia and Herzegovina	2.94 (2.38 to 3.65)
2016	Bosnia and Herzegovina	2.99 (2.39 to 3.76)
2017	Bosnia and Herzegovina	2.95 (2.34 to 3.71)
2018	Bosnia and Herzegovina	2.98 (2.33 to 3.76)
2019	Bosnia and Herzegovina	3.06 (2.38 to 3.94)
2020	Bosnia and Herzegovina	3.16 (2.42 to 4.09)
2021	Bosnia and Herzegovina	3.00 (2.15 to 3.99)
1990	Bulgaria	2.03 (1.82 to 2.23)
1991	Bulgaria	1.94 (1.76 to 2.13)
1992	Bulgaria	2.00 (1.80 to 2.19)
1993	Bulgaria	2.18 (1.95 to 2.40)
1994	Bulgaria	2.31 (2.08 to 2.57)
1995	Bulgaria	2.37 (2.14 to 2.62)
1996	Bulgaria	2.39 (2.14 to 2.65)
1997	Bulgaria	2.34 (2.08 to 2.63)
1998	Bulgaria	2.22 (1.99 to 2.50)
1999	Bulgaria	2.24 (1.99 to 2.52)
2000	Bulgaria	2.48 (2.22 to 2.78)
2001	Bulgaria	2.58 (2.29 to 2.89)
2002	Bulgaria	2.54 (2.27 to 2.85)
2003	Bulgaria	2.67 (2.39 to 3.02)
2004	Bulgaria	2.66 (2.38 to 2.98)
2005	Bulgaria	2.88 (2.56 to 3.22)
2006	Bulgaria	2.88 (2.59 to 3.24)
2007	Bulgaria	2.82 (2.53 to 3.17)

2008	Bulgaria	2.76 (2.46 to 3.07)
2009	Bulgaria	2.69 (2.41 to 2.98)
2010	Bulgaria	2.57 (2.31 to 2.86)
2011	Bulgaria	2.60 (2.33 to 2.88)
2012	Bulgaria	2.61 (2.32 to 2.92)
2013	Bulgaria	2.58 (2.30 to 2.89)
2014	Bulgaria	2.56 (2.30 to 2.90)
2015	Bulgaria	2.57 (2.27 to 2.94)
2016	Bulgaria	2.58 (2.26 to 2.97)
2017	Bulgaria	2.61 (2.27 to 3.01)
2018	Bulgaria	2.57 (2.23 to 2.99)
2019	Bulgaria	2.58 (2.21 to 3.05)
2020	Bulgaria	2.59 (2.15 to 3.13)
2021	Bulgaria	2.62 (2.12 to 3.23)
1990	Croatia	2.79 (2.45 to 3.19)
1991	Croatia	3.02 (2.67 to 3.48)
1992	Croatia	3.06 (2.71 to 3.51)
1993	Croatia	3.11 (2.74 to 3.54)
1994	Croatia	3.15 (2.74 to 3.59)
1995	Croatia	3.24 (2.80 to 3.73)
1996	Croatia	3.24 (2.74 to 3.74)
1997	Croatia	3.21 (2.73 to 3.73)
1998	Croatia	3.21 (2.73 to 3.72)
1999	Croatia	3.34 (2.86 to 3.87)
2000	Croatia	3.47 (2.99 to 3.97)
2001	Croatia	3.61 (3.14 to 4.12)
2002	Croatia	3.67 (3.23 to 4.22)
2003	Croatia	3.68 (3.24 to 4.20)
2004	Croatia	3.50 (3.06 to 3.98)
2005	Croatia	3.57 (3.11 to 4.09)
2006	Croatia	3.42 (2.95 to 3.92)
2007	Croatia	3.54 (3.03 to 4.08)
2008	Croatia	3.79 (3.26 to 4.38)
2009	Croatia	3.82 (3.29 to 4.42)
2010	Croatia	3.92 (3.37 to 4.56)
2011	Croatia	3.62 (3.13 to 4.19)
2012	Croatia	3.78 (3.26 to 4.34)
2013	Croatia	3.76 (3.26 to 4.33)
2014	Croatia	3.61 (3.12 to 4.14)
2015	Croatia	3.67 (3.19 to 4.19)
2016	Croatia	3.44 (2.99 to 3.92)
2017	Croatia	3.42 (2.99 to 3.85)

2018	Croatia	3.42 (2.97 to 3.86)
2019	Croatia	3.34 (2.91 to 3.78)
2020	Croatia	3.31 (2.87 to 3.76)
2021	Croatia	3.34 (2.87 to 3.86)
1990	Czech Republic	2.96 (2.76 to 3.18)
1991	Czech Republic	3.06 (2.86 to 3.28)
1992	Czech Republic	3.08 (2.87 to 3.30)
1993	Czech Republic	3.14 (2.90 to 3.38)
1994	Czech Republic	3.24 (2.97 to 3.47)
1995	Czech Republic	3.19 (2.91 to 3.44)
1996	Czech Republic	3.18 (2.89 to 3.43)
1997	Czech Republic	3.22 (2.92 to 3.48)
1998	Czech Republic	3.16 (2.86 to 3.44)
1999	Czech Republic	3.34 (3.04 to 3.62)
2000	Czech Republic	3.78 (3.46 to 4.11)
2001	Czech Republic	4.03 (3.69 to 4.35)
2002	Czech Republic	4.00 (3.68 to 4.30)
2003	Czech Republic	4.08 (3.74 to 4.38)
2004	Czech Republic	4.03 (3.70 to 4.30)
2005	Czech Republic	3.85 (3.55 to 4.12)
2006	Czech Republic	3.73 (3.44 to 3.99)
2007	Czech Republic	3.61 (3.34 to 3.87)
2008	Czech Republic	3.55 (3.28 to 3.82)
2009	Czech Republic	3.57 (3.29 to 3.84)
2010	Czech Republic	3.67 (3.38 to 3.96)
2011	Czech Republic	3.64 (3.35 to 3.93)
2012	Czech Republic	3.47 (3.19 to 3.74)
2013	Czech Republic	3.31 (3.04 to 3.59)
2014	Czech Republic	3.24 (2.99 to 3.49)
2015	Czech Republic	3.16 (2.91 to 3.42)
2016	Czech Republic	3.00 (2.77 to 3.26)
2017	Czech Republic	2.86 (2.62 to 3.09)
2018	Czech Republic	2.90 (2.64 to 3.12)
2019	Czech Republic	2.87 (2.62 to 3.08)
2020	Czech Republic	2.86 (2.57 to 3.11)
2021	Czech Republic	2.83 (2.46 to 3.19)
1990	Hungary	3.23 (3.00 to 3.48)
1991	Hungary	3.42 (3.16 to 3.67)
1992	Hungary	3.68 (3.40 to 3.94)
1993	Hungary	3.70 (3.42 to 3.97)
1994	Hungary	3.76 (3.47 to 4.04)
1995	Hungary	3.73 (3.45 to 3.99)

1996	Hungary	3.64 (3.35 to 3.91)
1997	Hungary	3.49 (3.21 to 3.78)
1998	Hungary	3.48 (3.17 to 3.77)
1999	Hungary	3.55 (3.22 to 3.86)
2000	Hungary	3.38 (3.06 to 3.69)
2001	Hungary	3.34 (3.00 to 3.65)
2002	Hungary	3.38 (3.04 to 3.70)
2003	Hungary	3.43 (3.11 to 3.74)
2004	Hungary	3.44 (3.13 to 3.72)
2005	Hungary	3.55 (3.26 to 3.82)
2006	Hungary	3.41 (3.15 to 3.65)
2007	Hungary	3.52 (3.26 to 3.75)
2008	Hungary	3.31 (3.07 to 3.54)
2009	Hungary	3.31 (3.08 to 3.54)
2010	Hungary	3.29 (3.05 to 3.52)
2011	Hungary	3.19 (2.97 to 3.42)
2012	Hungary	3.20 (2.99 to 3.43)
2013	Hungary	3.13 (2.91 to 3.36)
2014	Hungary	3.05 (2.85 to 3.27)
2015	Hungary	3.03 (2.82 to 3.25)
2016	Hungary	2.90 (2.69 to 3.09)
2017	Hungary	2.97 (2.75 to 3.19)
2018	Hungary	3.11 (2.88 to 3.31)
2019	Hungary	2.96 (2.74 to 3.17)
2020	Hungary	2.94 (2.67 to 3.21)
2021	Hungary	2.87 (2.54 to 3.23)
1990	Montenegro	6.70 (5.35 to 8.64)
1991	Montenegro	6.77 (5.29 to 8.87)
1992	Montenegro	6.72 (5.26 to 8.72)
1993	Montenegro	6.63 (5.26 to 8.46)
1994	Montenegro	6.61 (5.27 to 8.35)
1995	Montenegro	6.50 (5.27 to 8.08)
1996	Montenegro	6.41 (5.25 to 7.86)
1997	Montenegro	6.50 (5.37 to 7.82)
1998	Montenegro	6.66 (5.59 to 7.97)
1999	Montenegro	6.72 (5.75 to 8.06)
2000	Montenegro	6.66 (5.71 to 8.01)
2001	Montenegro	6.77 (5.73 to 8.13)
2002	Montenegro	6.87 (5.84 to 8.20)
2003	Montenegro	7.04 (6.02 to 8.35)
2004	Montenegro	6.99 (5.96 to 8.31)
2005	Montenegro	6.91 (5.92 to 8.17)

2006	Montenegro	6.99 (6.05 to 8.20)
2007	Montenegro	6.80 (5.83 to 8.01)
2008	Montenegro	6.84 (5.75 to 8.10)
2009	Montenegro	7.27 (5.98 to 8.64)
2010	Montenegro	7.62 (6.17 to 9.18)
2011	Montenegro	7.71 (6.30 to 9.40)
2012	Montenegro	7.79 (6.49 to 9.48)
2013	Montenegro	7.97 (6.59 to 9.80)
2014	Montenegro	8.09 (6.64 to 9.90)
2015	Montenegro	8.35 (6.81 to 10.30)
2016	Montenegro	8.61 (7.03 to 10.78)
2017	Montenegro	8.77 (7.08 to 11.05)
2018	Montenegro	8.76 (6.95 to 10.97)
2019	Montenegro	8.85 (6.95 to 11.21)
2020	Montenegro	9.31 (7.28 to 11.88)
2021	Montenegro	8.65 (6.59 to 11.28)
1990	North Macedonia	1.94 (1.51 to 2.42)
1991	North Macedonia	1.96 (1.51 to 2.43)
1992	North Macedonia	2.06 (1.63 to 2.50)
1993	North Macedonia	2.12 (1.68 to 2.55)
1994	North Macedonia	2.16 (1.73 to 2.57)
1995	North Macedonia	2.23 (1.84 to 2.64)
1996	North Macedonia	2.31 (1.94 to 2.74)
1997	North Macedonia	2.37 (1.98 to 2.81)
1998	North Macedonia	2.49 (2.11 to 2.95)
1999	North Macedonia	2.59 (2.20 to 3.04)
2000	North Macedonia	2.56 (2.16 to 3.05)
2001	North Macedonia	2.62 (2.21 to 3.12)
2002	North Macedonia	2.64 (2.21 to 3.16)
2003	North Macedonia	2.58 (2.18 to 3.10)
2004	North Macedonia	2.51 (2.11 to 2.96)
2005	North Macedonia	2.55 (2.14 to 3.02)
2006	North Macedonia	2.69 (2.26 to 3.20)
2007	North Macedonia	2.73 (2.34 to 3.22)
2008	North Macedonia	2.67 (2.22 to 3.13)
2009	North Macedonia	2.68 (2.22 to 3.18)
2010	North Macedonia	2.64 (2.17 to 3.12)
2011	North Macedonia	2.55 (2.11 to 3.00)
2012	North Macedonia	2.56 (2.12 to 3.04)
2013	North Macedonia	2.53 (2.08 to 2.99)
2014	North Macedonia	2.54 (2.10 to 3.05)
2015	North Macedonia	2.49 (2.03 to 2.97)

2016	North Macedonia	2.55 (2.07 to 3.07)
2017	North Macedonia	2.46 (2.02 to 2.98)
2018	North Macedonia	2.54 (2.06 to 3.13)
2019	North Macedonia	2.64 (2.12 to 3.27)
2020	North Macedonia	2.68 (1.86 to 3.67)
2021	North Macedonia	2.63 (1.80 to 3.81)
1990	Poland	4.54 (4.36 to 4.66)
1991	Poland	5.14 (4.95 to 5.29)
1992	Poland	5.25 (5.05 to 5.40)
1993	Poland	5.19 (4.98 to 5.34)
1994	Poland	5.21 (5.00 to 5.35)
1995	Poland	5.21 (5.02 to 5.36)
1996	Poland	5.16 (4.95 to 5.31)
1997	Poland	5.11 (4.92 to 5.26)
1998	Poland	4.74 (4.54 to 4.88)
1999	Poland	4.71 (4.52 to 4.86)
2000	Poland	4.71 (4.49 to 4.84)
2001	Poland	4.73 (4.50 to 4.87)
2002	Poland	4.81 (4.58 to 4.96)
2003	Poland	4.85 (4.61 to 4.99)
2004	Poland	4.89 (4.66 to 5.03)
2005	Poland	4.75 (4.52 to 4.89)
2006	Poland	4.76 (4.55 to 4.90)
2007	Poland	4.72 (4.50 to 4.86)
2008	Poland	4.74 (4.52 to 4.88)
2009	Poland	4.55 (4.33 to 4.68)
2010	Poland	4.26 (4.05 to 4.40)
2011	Poland	4.11 (3.89 to 4.25)
2012	Poland	4.17 (3.95 to 4.32)
2013	Poland	4.11 (3.88 to 4.25)
2014	Poland	3.90 (3.68 to 4.05)
2015	Poland	3.83 (3.62 to 3.97)
2016	Poland	3.72 (3.52 to 3.86)
2017	Poland	3.62 (3.42 to 3.75)
2018	Poland	3.55 (3.36 to 3.69)
2019	Poland	3.41 (3.22 to 3.53)
2020	Poland	3.42 (3.17 to 3.66)
2021	Poland	3.44 (3.09 to 3.79)
1990	Romania	1.40 (1.30 to 1.52)
1991	Romania	1.42 (1.32 to 1.53)
1992	Romania	1.49 (1.39 to 1.60)
1993	Romania	1.51 (1.41 to 1.62)

1994	Romania	1.54 (1.44 to 1.64)
1995	Romania	1.59 (1.49 to 1.69)
1996	Romania	1.64 (1.55 to 1.75)
1997	Romania	1.62 (1.53 to 1.72)
1998	Romania	1.56 (1.48 to 1.65)
1999	Romania	1.55 (1.46 to 1.63)
2000	Romania	1.55 (1.47 to 1.64)
2001	Romania	1.61 (1.52 to 1.70)
2002	Romania	1.68 (1.59 to 1.77)
2003	Romania	1.75 (1.66 to 1.85)
2004	Romania	1.78 (1.67 to 1.88)
2005	Romania	1.77 (1.67 to 1.86)
2006	Romania	1.70 (1.60 to 1.80)
2007	Romania	1.66 (1.56 to 1.76)
2008	Romania	1.71 (1.61 to 1.82)
2009	Romania	1.78 (1.67 to 1.89)
2010	Romania	1.77 (1.67 to 1.89)
2011	Romania	1.64 (1.54 to 1.74)
2012	Romania	1.72 (1.62 to 1.83)
2013	Romania	1.63 (1.53 to 1.73)
2014	Romania	1.69 (1.59 to 1.80)
2015	Romania	1.63 (1.52 to 1.74)
2016	Romania	1.73 (1.61 to 1.84)
2017	Romania	1.78 (1.65 to 1.91)
2018	Romania	1.87 (1.72 to 2.01)
2019	Romania	1.86 (1.69 to 2.02)
2020	Romania	1.85 (1.66 to 2.06)
2021	Romania	1.90 (1.65 to 2.18)
1990	Serbia	4.22 (3.50 to 5.14)
1991	Serbia	4.21 (3.41 to 5.21)
1992	Serbia	4.15 (3.34 to 5.24)
1993	Serbia	4.15 (3.35 to 5.12)
1994	Serbia	4.18 (3.51 to 4.97)
1995	Serbia	4.23 (3.69 to 4.88)
1996	Serbia	4.22 (3.70 to 4.81)
1997	Serbia	4.17 (3.60 to 4.83)
1998	Serbia	3.94 (3.48 to 4.45)
1999	Serbia	3.96 (3.51 to 4.44)
2000	Serbia	4.07 (3.64 to 4.58)
2001	Serbia	4.03 (3.60 to 4.51)
2002	Serbia	4.03 (3.59 to 4.46)
2003	Serbia	4.11 (3.67 to 4.62)

2004	Serbia	4.17 (3.69 to 4.70)
2005	Serbia	4.19 (3.71 to 4.77)
2006	Serbia	4.20 (3.66 to 4.75)
2007	Serbia	4.25 (3.68 to 4.80)
2008	Serbia	4.26 (3.71 to 4.82)
2009	Serbia	4.20 (3.67 to 4.81)
2010	Serbia	4.14 (3.62 to 4.77)
2011	Serbia	4.14 (3.57 to 4.74)
2012	Serbia	4.14 (3.58 to 4.67)
2013	Serbia	4.07 (3.54 to 4.59)
2014	Serbia	4.07 (3.55 to 4.60)
2015	Serbia	4.12 (3.62 to 4.71)
2016	Serbia	4.06 (3.56 to 4.67)
2017	Serbia	4.08 (3.53 to 4.72)
2018	Serbia	3.98 (3.36 to 4.64)
2019	Serbia	4.01 (3.37 to 4.68)
2020	Serbia	4.18 (3.39 to 5.09)
2021	Serbia	4.05 (3.21 to 5.10)
1990	Slovakia	2.29 (1.97 to 2.68)
1991	Slovakia	2.24 (1.94 to 2.60)
1992	Slovakia	2.23 (1.97 to 2.56)
1993	Slovakia	2.21 (1.98 to 2.52)
1994	Slovakia	2.21 (1.99 to 2.51)
1995	Slovakia	2.22 (2.00 to 2.51)
1996	Slovakia	2.21 (2.00 to 2.46)
1997	Slovakia	2.28 (2.06 to 2.53)
1998	Slovakia	2.32 (2.12 to 2.58)
1999	Slovakia	2.34 (2.14 to 2.58)
2000	Slovakia	2.34 (2.14 to 2.58)
2001	Slovakia	2.37 (2.13 to 2.61)
2002	Slovakia	2.38 (2.14 to 2.62)
2003	Slovakia	2.38 (2.13 to 2.62)
2004	Slovakia	2.36 (2.10 to 2.61)
2005	Slovakia	2.42 (2.17 to 2.69)
2006	Slovakia	2.41 (2.16 to 2.69)
2007	Slovakia	2.43 (2.18 to 2.71)
2008	Slovakia	2.39 (2.12 to 2.65)
2009	Slovakia	2.35 (2.11 to 2.61)
2010	Slovakia	2.30 (2.06 to 2.54)
2011	Slovakia	2.31 (2.08 to 2.55)
2012	Slovakia	2.30 (2.04 to 2.53)
2013	Slovakia	2.26 (2.03 to 2.52)

2014	Slovakia	2.26 (2.01 to 2.54)
2015	Slovakia	2.28 (2.04 to 2.57)
2016	Slovakia	2.22 (1.95 to 2.54)
2017	Slovakia	2.24 (1.94 to 2.55)
2018	Slovakia	2.26 (1.96 to 2.60)
2019	Slovakia	2.21 (1.87 to 2.61)
2020	Slovakia	2.27 (1.84 to 2.78)
2021	Slovakia	2.29 (1.79 to 2.90)
1990	Slovenia	2.66 (2.47 to 2.84)
1991	Slovenia	2.94 (2.72 to 3.13)
1992	Slovenia	3.02 (2.81 to 3.22)
1993	Slovenia	3.15 (2.91 to 3.36)
1994	Slovenia	3.07 (2.86 to 3.28)
1995	Slovenia	2.96 (2.76 to 3.17)
1996	Slovenia	2.99 (2.77 to 3.21)
1997	Slovenia	3.05 (2.81 to 3.29)
1998	Slovenia	3.13 (2.87 to 3.39)
1999	Slovenia	3.21 (2.92 to 3.49)
2000	Slovenia	3.23 (2.95 to 3.51)
2001	Slovenia	3.38 (3.10 to 3.67)
2002	Slovenia	3.40 (3.12 to 3.68)
2003	Slovenia	3.46 (3.17 to 3.76)
2004	Slovenia	3.27 (2.98 to 3.54)
2005	Slovenia	3.14 (2.85 to 3.40)
2006	Slovenia	3.11 (2.86 to 3.38)
2007	Slovenia	2.97 (2.71 to 3.23)
2008	Slovenia	2.93 (2.68 to 3.15)
2009	Slovenia	2.82 (2.59 to 3.04)
2010	Slovenia	2.84 (2.60 to 3.07)
2011	Slovenia	2.73 (2.48 to 2.95)
2012	Slovenia	2.84 (2.58 to 3.06)
2013	Slovenia	2.71 (2.44 to 2.92)
2014	Slovenia	2.56 (2.29 to 2.78)
2015	Slovenia	2.64 (2.40 to 2.86)
2016	Slovenia	2.58 (2.33 to 2.82)
2017	Slovenia	2.50 (2.25 to 2.71)
2018	Slovenia	2.43 (2.17 to 2.66)
2019	Slovenia	2.25 (2.01 to 2.47)
2020	Slovenia	2.22 (1.94 to 2.58)
2021	Slovenia	2.16 (1.82 to 2.58)

ASDR, age-standardized death rates.

Fig 3D. ASDR in Eastern Europe from 1990 to 2021

Year	Location	ASDR per 100,000
1990	Belarus	2.67 (2.31 to 3.23)
1991	Belarus	2.75 (2.40 to 3.33)
1992	Belarus	2.88 (2.52 to 3.43)
1993	Belarus	3.19 (2.80 to 3.78)
1994	Belarus	3.25 (2.85 to 3.84)
1995	Belarus	3.42 (3.00 to 4.04)
1996	Belarus	3.40 (2.97 to 3.99)
1997	Belarus	3.43 (3.01 to 4.00)
1998	Belarus	3.49 (3.04 to 4.09)
1999	Belarus	3.58 (3.12 to 4.19)
2000	Belarus	3.42 (3.00 to 3.96)
2001	Belarus	3.64 (3.22 to 4.24)
2002	Belarus	4.04 (3.55 to 4.69)
2003	Belarus	4.00 (3.53 to 4.65)
2004	Belarus	3.99 (3.50 to 4.59)
2005	Belarus	4.17 (3.67 to 4.80)
2006	Belarus	4.08 (3.63 to 4.68)
2007	Belarus	3.91 (3.49 to 4.44)
2008	Belarus	3.98 (3.55 to 4.48)
2009	Belarus	4.04 (3.61 to 4.53)
2010	Belarus	4.12 (3.71 to 4.64)
2011	Belarus	4.23 (3.82 to 4.69)
2012	Belarus	3.73 (3.38 to 4.11)
2013	Belarus	3.70 (3.39 to 4.02)
2014	Belarus	3.65 (3.36 to 3.96)
2015	Belarus	3.54 (3.27 to 3.83)
2016	Belarus	3.55 (3.28 to 3.83)
2017	Belarus	3.54 (3.28 to 3.82)
2018	Belarus	3.66 (3.38 to 3.98)
2019	Belarus	3.74 (3.45 to 4.07)
2020	Belarus	3.76 (3.27 to 4.32)
2021	Belarus	3.75 (3.05 to 4.49)
1990	Estonia	2.86 (2.60 to 3.13)
1991	Estonia	3.01 (2.78 to 3.29)
1992	Estonia	3.08 (2.84 to 3.31)
1993	Estonia	3.39 (3.13 to 3.66)
1994	Estonia	3.81 (3.54 to 4.09)
1995	Estonia	3.65 (3.40 to 3.93)
1996	Estonia	3.28 (3.06 to 3.53)
1997	Estonia	3.43 (3.19 to 3.68)

1998	Estonia	3.53 (3.28 to 3.78)
1999	Estonia	4.04 (3.73 to 4.32)
2000	Estonia	3.73 (3.47 to 3.99)
2001	Estonia	3.22 (2.99 to 3.43)
2002	Estonia	3.06 (2.86 to 3.27)
2003	Estonia	2.87 (2.66 to 3.07)
2004	Estonia	2.84 (2.63 to 3.06)
2005	Estonia	2.79 (2.58 to 3.00)
2006	Estonia	2.90 (2.68 to 3.10)
2007	Estonia	3.17 (2.92 to 3.43)
2008	Estonia	2.93 (2.72 to 3.17)
2009	Estonia	3.05 (2.79 to 3.31)
2010	Estonia	3.06 (2.79 to 3.33)
2011	Estonia	3.13 (2.89 to 3.41)
2012	Estonia	3.22 (2.93 to 3.48)
2013	Estonia	3.23 (2.95 to 3.51)
2014	Estonia	3.47 (3.18 to 3.77)
2015	Estonia	3.31 (3.02 to 3.61)
2016	Estonia	3.43 (3.13 to 3.72)
2017	Estonia	3.37 (3.07 to 3.67)
2018	Estonia	3.51 (3.19 to 3.86)
2019	Estonia	3.40 (3.07 to 3.71)
2020	Estonia	3.36 (3.03 to 3.71)
2021	Estonia	3.34 (2.90 to 3.78)
1990	Latvia	2.21 (2.03 to 2.40)
1991	Latvia	2.27 (2.10 to 2.47)
1992	Latvia	2.38 (2.20 to 2.56)
1993	Latvia	2.76 (2.57 to 2.96)
1994	Latvia	3.08 (2.86 to 3.31)
1995	Latvia	2.93 (2.74 to 3.12)
1996	Latvia	2.61 (2.44 to 2.78)
1997	Latvia	2.57 (2.42 to 2.74)
1998	Latvia	2.72 (2.55 to 2.90)
1999	Latvia	3.01 (2.81 to 3.19)
2000	Latvia	2.75 (2.57 to 2.91)
2001	Latvia	2.44 (2.30 to 2.59)
2002	Latvia	2.39 (2.23 to 2.54)
2003	Latvia	2.39 (2.23 to 2.55)
2004	Latvia	2.39 (2.24 to 2.55)
2005	Latvia	2.55 (2.40 to 2.72)
2006	Latvia	2.65 (2.50 to 2.81)
2007	Latvia	2.73 (2.57 to 2.89)

2008	Latvia	2.45 (2.31 to 2.59)
2009	Latvia	2.47 (2.32 to 2.61)
2010	Latvia	2.51 (2.35 to 2.66)
2011	Latvia	2.50 (2.35 to 2.66)
2012	Latvia	2.60 (2.43 to 2.75)
2013	Latvia	2.73 (2.55 to 2.90)
2014	Latvia	2.74 (2.56 to 2.92)
2015	Latvia	2.77 (2.59 to 2.97)
2016	Latvia	2.91 (2.70 to 3.10)
2017	Latvia	3.15 (2.91 to 3.40)
2018	Latvia	3.17 (2.92 to 3.42)
2019	Latvia	3.02 (2.75 to 3.28)
2020	Latvia	3.07 (2.76 to 3.44)
2021	Latvia	3.05 (2.63 to 3.47)
1990	Lithuania	2.16 (1.97 to 2.36)
1991	Lithuania	2.27 (2.10 to 2.47)
1992	Lithuania	2.30 (2.13 to 2.48)
1993	Lithuania	2.63 (2.45 to 2.82)
1994	Lithuania	2.78 (2.59 to 2.97)
1995	Lithuania	2.63 (2.45 to 2.80)
1996	Lithuania	2.53 (2.36 to 2.71)
1997	Lithuania	2.42 (2.27 to 2.58)
1998	Lithuania	2.51 (2.35 to 2.67)
1999	Lithuania	3.01 (2.83 to 3.18)
2000	Lithuania	2.73 (2.57 to 2.88)
2001	Lithuania	2.56 (2.40 to 2.69)
2002	Lithuania	2.49 (2.35 to 2.62)
2003	Lithuania	2.43 (2.30 to 2.56)
2004	Lithuania	2.43 (2.30 to 2.57)
2005	Lithuania	2.72 (2.56 to 2.87)
2006	Lithuania	2.71 (2.55 to 2.88)
2007	Lithuania	2.92 (2.73 to 3.11)
2008	Lithuania	2.89 (2.71 to 3.08)
2009	Lithuania	2.74 (2.56 to 2.93)
2010	Lithuania	2.83 (2.64 to 3.04)
2011	Lithuania	2.95 (2.76 to 3.18)
2012	Lithuania	2.86 (2.67 to 3.08)
2013	Lithuania	3.00 (2.79 to 3.23)
2014	Lithuania	3.06 (2.83 to 3.30)
2015	Lithuania	3.24 (3.01 to 3.47)
2016	Lithuania	3.30 (3.07 to 3.54)
2017	Lithuania	3.25 (3.02 to 3.50)

2018	Lithuania	3.12 (2.88 to 3.36)
2019	Lithuania	3.14 (2.90 to 3.37)
2020	Lithuania	3.18 (2.84 to 3.50)
2021	Lithuania	3.15 (2.73 to 3.56)
1990	Republic of Moldova	0.96 (0.89 to 1.06)
1991	Republic of Moldova	1.05 (0.96 to 1.14)
1992	Republic of Moldova	1.02 (0.94 to 1.10)
1993	Republic of Moldova	1.05 (0.98 to 1.13)
1994	Republic of Moldova	1.18 (1.10 to 1.27)
1995	Republic of Moldova	1.21 (1.13 to 1.30)
1996	Republic of Moldova	1.15 (1.07 to 1.23)
1997	Republic of Moldova	1.08 (1.00 to 1.15)
1998	Republic of Moldova	1.07 (0.99 to 1.14)
1999	Republic of Moldova	1.26 (1.17 to 1.34)
2000	Republic of Moldova	1.14 (1.07 to 1.21)
2001	Republic of Moldova	0.92 (0.85 to 0.97)
2002	Republic of Moldova	0.93 (0.87 to 0.99)
2003	Republic of Moldova	0.96 (0.89 to 1.02)
2004	Republic of Moldova	0.93 (0.87 to 1.00)
2005	Republic of Moldova	1.04 (0.97 to 1.11)
2006	Republic of Moldova	1.11 (1.04 to 1.19)
2007	Republic of Moldova	1.11 (1.03 to 1.19)
2008	Republic of Moldova	1.15 (1.06 to 1.24)
2009	Republic of Moldova	1.19 (1.10 to 1.29)
2010	Republic of Moldova	1.25 (1.15 to 1.36)
2011	Republic of Moldova	1.18 (1.08 to 1.28)
2012	Republic of Moldova	1.24 (1.15 to 1.35)
2013	Republic of Moldova	1.32 (1.23 to 1.43)
2014	Republic of Moldova	1.41 (1.31 to 1.52)
2015	Republic of Moldova	1.49 (1.39 to 1.61)
2016	Republic of Moldova	1.46 (1.36 to 1.60)
2017	Republic of Moldova	1.39 (1.29 to 1.51)
2018	Republic of Moldova	1.46 (1.35 to 1.60)
2019	Republic of Moldova	1.47 (1.32 to 1.62)
2020	Republic of Moldova	1.47 (1.34 to 1.61)
2021	Republic of Moldova	1.49 (1.32 to 1.67)
1990	Russian Federation	2.72 (2.62 to 2.78)
1991	Russian Federation	2.78 (2.69 to 2.84)
1992	Russian Federation	2.99 (2.90 to 3.06)
1993	Russian Federation	3.60 (3.50 to 3.68)
1994	Russian Federation	3.95 (3.85 to 4.04)
1995	Russian Federation	3.59 (3.49 to 3.66)

1996	Russian Federation	3.27 (3.19 to 3.34)
1997	Russian Federation	3.05 (2.97 to 3.11)
1998	Russian Federation	3.07 (2.98 to 3.14)
1999	Russian Federation	4.18 (4.03 to 4.33)
2000	Russian Federation	3.91 (3.77 to 4.06)
2001	Russian Federation	2.91 (2.83 to 3.00)
2002	Russian Federation	2.85 (2.78 to 2.90)
2003	Russian Federation	2.87 (2.80 to 2.93)
2004	Russian Federation	2.85 (2.78 to 2.91)
2005	Russian Federation	3.10 (3.02 to 3.16)
2006	Russian Federation	3.02 (2.94 to 3.08)
2007	Russian Federation	3.09 (3.00 to 3.16)
2008	Russian Federation	3.31 (3.22 to 3.39)
2009	Russian Federation	3.42 (3.32 to 3.50)
2010	Russian Federation	3.60 (3.49 to 3.67)
2011	Russian Federation	3.60 (3.48 to 3.68)
2012	Russian Federation	3.68 (3.56 to 3.77)
2013	Russian Federation	3.85 (3.71 to 3.94)
2014	Russian Federation	4.21 (4.05 to 4.31)
2015	Russian Federation	4.33 (4.15 to 4.43)
2016	Russian Federation	4.46 (4.27 to 4.57)
2017	Russian Federation	4.33 (4.13 to 4.44)
2018	Russian Federation	4.40 (4.21 to 4.51)
2019	Russian Federation	4.42 (4.21 to 4.52)
2020	Russian Federation	4.39 (4.19 to 4.61)
2021	Russian Federation	4.38 (4.01 to 4.74)
1990	Ukraine	2.13 (1.92 to 2.37)
1991	Ukraine	2.27 (2.04 to 2.51)
1992	Ukraine	2.42 (2.19 to 2.66)
1993	Ukraine	2.54 (2.31 to 2.76)
1994	Ukraine	2.66 (2.42 to 2.89)
1995	Ukraine	2.87 (2.60 to 3.13)
1996	Ukraine	2.80 (2.53 to 3.06)
1997	Ukraine	2.69 (2.44 to 2.95)
1998	Ukraine	2.53 (2.30 to 2.76)
1999	Ukraine	2.61 (2.38 to 2.86)
2000	Ukraine	2.71 (2.47 to 2.98)
2001	Ukraine	2.66 (2.43 to 2.93)
2002	Ukraine	2.72 (2.49 to 3.00)
2003	Ukraine	2.74 (2.49 to 3.01)
2004	Ukraine	2.86 (2.62 to 3.14)
2005	Ukraine	3.06 (2.82 to 3.35)

2006	Ukraine	2.95 (2.71 to 3.22)
2007	Ukraine	3.08 (2.83 to 3.35)
2008	Ukraine	3.12 (2.89 to 3.38)
2009	Ukraine	2.78 (2.57 to 2.99)
2010	Ukraine	2.67 (2.49 to 2.86)
2011	Ukraine	2.58 (2.42 to 2.74)
2012	Ukraine	2.59 (2.44 to 2.76)
2013	Ukraine	2.61 (2.45 to 2.76)
2014	Ukraine	2.55 (2.39 to 2.70)
2015	Ukraine	2.49 (2.34 to 2.65)
2016	Ukraine	2.50 (2.34 to 2.66)
2017	Ukraine	2.53 (2.26 to 2.81)
2018	Ukraine	2.59 (2.21 to 3.03)
2019	Ukraine	2.62 (2.16 to 3.14)
2020	Ukraine	2.47 (2.07 to 2.97)
2021	Ukraine	2.47 (1.82 to 3.25)

ASDR, age-standardized death rates.

Fig 3E. ASDR in men and women from Europe in 2021

Sex	Location	ASDR per 100,000
Male	Central Europe	4.74 (4.38 to 5.15)
Female	Central Europe	1.64 (1.42 to 1.95)
Male	Eastern Europe	6.52 (5.85 to 7.16)
Female	Eastern Europe	2.17 (1.92 to 2.45)
Male	Western Europe	3.95 (3.63 to 4.13)
Female	Western Europe	1.50 (1.27 to 1.68)

ASDR, age-standardized death rates.

Fig 3F. ASDR in men and women from Western Europe in 2021

Sex	Location	ASDR per 100,000
Male	Andorra	5.80 (3.35 to 9.23)
Female	Andorra	2.24 (1.45 to 3.29)
Male	Austria	2.52 (2.25 to 2.72)
Female	Austria	1.24 (1.04 to 1.38)
Male	Belgium	3.70 (3.28 to 4.03)
Female	Belgium	1.16 (0.96 to 1.32)
Male	Cyprus	6.54 (4.90 to 8.30)
Female	Cyprus	1.87 (1.42 to 2.38)
Male	Denmark	6.77 (6.01 to 7.44)
Female	Denmark	2.96 (2.55 to 3.34)
Male	Finland	5.58 (4.96 to 6.06)
Female	Finland	2.10 (1.76 to 2.41)
Male	France	3.11 (2.74 to 3.35)
Female	France	1.00 (0.82 to 1.13)

Male	Germany	3.30 (2.96 to 3.57)
Female	Germany	1.39 (1.19 to 1.57)
Male	Greece	6.71 (6.13 to 7.18)
Female	Greece	1.71 (1.46 to 1.89)
Male	Iceland	3.28 (2.86 to 3.67)
Female	Iceland	2.01 (1.65 to 2.31)
Male	Ireland	3.74 (3.21 to 4.19)
Female	Ireland	2.24 (1.81 to 2.65)
Male	Israel	1.88 (1.66 to 2.07)
Female	Israel	0.79 (0.65 to 0.90)
Male	Italy	3.59 (3.28 to 3.79)
Female	Italy	1.12 (0.90 to 1.36)
Male	Luxembourg	3.69 (3.29 to 4.12)
Female	Luxembourg	1.10 (0.95 to 1.27)
Male	Malta	2.30 (2.05 to 2.60)
Female	Malta	0.52 (0.43 to 0.59)
Male	Monaco	6.35 (4.63 to 8.60)
Female	Monaco	4.11 (2.83 to 5.96)
Male	Netherlands	5.64 (4.95 to 6.22)
Female	Netherlands	2.37 (1.98 to 2.70)
Male	Norway	6.32 (5.66 to 6.71)
Female	Norway	3.46 (2.92 to 3.79)
Male	Portugal	2.61 (2.39 to 2.83)
Female	Portugal	0.67 (0.57 to 0.77)
Male	San Marino	3.13 (1.98 to 4.92)
Female	San Marino	0.37 (0.22 to 0.57)
Male	Spain	3.81 (3.43 to 4.08)
Female	Spain	0.76 (0.62 to 0.89)
Male	Sweden	4.83 (4.11 to 5.51)
Female	Sweden	3.09 (2.55 to 3.55)
Male	Switzerland	3.39 (2.99 to 3.70)
Female	Switzerland	1.42 (1.11 to 1.67)
Male	United Kingdom of Great Britain and Northern Ireland	5.51 (5.06 to 5.74)
Female	United Kingdom of Great Britain and Northern Ireland	2.84 (2.41 to 3.13)

ASDR, age-standardized death rates.

Fig 3G. ASDR in men and women from Central Europe

Sex	Location	ASDR per 100,000
Male	Albania	1.58 (0.95 to 2.31)
Female	Albania	0.68 (0.46 to 0.96)
Male	Bosnia and Herzegovina	4.94 (3.26 to 6.82)
Female	Bosnia and Herzegovina	1.54 (1.05 to 2.23)
Male	Bulgaria	4.49 (3.68 to 5.52)

Female	Bulgaria	1.23 (0.94 to 1.61)
Male	Croatia	5.47 (4.69 to 6.32)
Female	Croatia	1.84 (1.49 to 2.34)
Male	Czech Republic	4.15 (3.60 to 4.68)
Female	Czech Republic	1.83 (1.51 to 2.26)
Male	Hungary	4.15 (3.64 to 4.69)
Female	Hungary	1.98 (1.73 to 2.35)
Male	Montenegro	14.76 (10.31 to 21.14)
Female	Montenegro	4.29 (3.31 to 5.25)
Male	North Macedonia	4.06 (2.69 to 5.99)
Female	North Macedonia	1.45 (0.77 to 2.28)
Male	Poland	5.96 (5.38 to 6.55)
Female	Poland	1.79 (1.50 to 2.20)
Male	Romania	2.78 (2.38 to 3.25)
Female	Romania	1.22 (1.00 to 1.55)
Male	Serbia	6.70 (5.06 to 8.79)
Female	Serbia	2.01 (1.54 to 2.61)
Male	Slovakia	3.72 (2.81 to 4.86)
Female	Slovakia	1.28 (0.93 to 1.69)
Male	Slovenia	3.49 (2.91 to 4.11)
Female	Slovenia	1.19 (0.91 to 1.72)

ASDR, age-standardized death rates.

Fig 3H. ASDR in men and women from Eastern Europe in 2021

Sex	Location	ASDR per 100,000
Male	Belarus	7.18 (5.87 to 8.77)
Female	Belarus	1.73 (1.39 to 2.11)
Male	Estonia	6.16 (5.28 to 7.17)
Female	Estonia	1.69 (1.39 to 1.97)
Male	Latvia	5.81 (5.00 to 6.76)
Female	Latvia	1.46 (1.18 to 1.87)
Male	Lithuania	5.67 (4.97 to 6.42)
Female	Lithuania	1.62 (1.32 to 2.05)
Male	Republic of Moldova	2.45 (2.11 to 2.81)
Female	Republic of Moldova	0.84 (0.72 to 0.98)
Male	Russian Federation	7.34 (6.55 to 8.04)
Female	Russian Federation	2.60 (2.30 to 2.98)
Male	Ukraine	4.53 (3.00 to 6.31)
Female	Ukraine	1.16 (0.79 to 1.67)

Data for Figure 4

Fig 4A. ASDR in Asia from 1990 to 2021

Year	Location	ASDR per 100,000
1990	Central Asia	0.94 (0.81 to 1.13)
1991	Central Asia	1.00 (0.87 to 1.18)
1992	Central Asia	1.07 (0.94 to 1.24)
1993	Central Asia	1.14 (1.01 to 1.31)
1994	Central Asia	1.16 (1.02 to 1.32)
1995	Central Asia	1.20 (1.06 to 1.37)
1996	Central Asia	1.21 (1.07 to 1.37)
1997	Central Asia	1.18 (1.03 to 1.34)
1998	Central Asia	1.16 (1.02 to 1.34)
1999	Central Asia	1.11 (0.98 to 1.27)
2000	Central Asia	1.15 (1.02 to 1.30)
2001	Central Asia	1.15 (1.02 to 1.29)
2002	Central Asia	1.19 (1.06 to 1.34)
2003	Central Asia	1.27 (1.14 to 1.41)
2004	Central Asia	1.27 (1.15 to 1.40)
2005	Central Asia	1.32 (1.20 to 1.45)
2006	Central Asia	1.36 (1.24 to 1.48)
2007	Central Asia	1.43 (1.31 to 1.55)
2008	Central Asia	1.45 (1.33 to 1.56)
2009	Central Asia	1.45 (1.33 to 1.56)
2010	Central Asia	1.54 (1.41 to 1.66)
2011	Central Asia	1.60 (1.47 to 1.72)
2012	Central Asia	1.69 (1.56 to 1.81)
2013	Central Asia	1.74 (1.60 to 1.86)
2014	Central Asia	1.81 (1.68 to 1.95)
2015	Central Asia	1.95 (1.80 to 2.10)
2016	Central Asia	2.00 (1.84 to 2.16)
2017	Central Asia	1.97 (1.81 to 2.13)
2018	Central Asia	2.05 (1.88 to 2.23)
2019	Central Asia	2.01 (1.85 to 2.20)
2020	Central Asia	2.00 (1.83 to 2.20)
2021	Central Asia	1.98 (1.77 to 2.21)
1990	East Asia	0.36 (0.29 to 0.44)
1991	East Asia	0.37 (0.30 to 0.45)
1992	East Asia	0.37 (0.31 to 0.46)
1993	East Asia	0.38 (0.32 to 0.46)
1994	East Asia	0.39 (0.34 to 0.46)
1995	East Asia	0.40 (0.35 to 0.47)
1996	East Asia	0.40 (0.36 to 0.47)

1997	East Asia	0.41 (0.36 to 0.46)
1998	East Asia	0.41 (0.37 to 0.46)
1999	East Asia	0.41 (0.37 to 0.46)
2000	East Asia	0.42 (0.38 to 0.46)
2001	East Asia	0.42 (0.38 to 0.46)
2002	East Asia	0.43 (0.39 to 0.47)
2003	East Asia	0.43 (0.40 to 0.47)
2004	East Asia	0.42 (0.39 to 0.46)
2005	East Asia	0.43 (0.39 to 0.46)
2006	East Asia	0.44 (0.40 to 0.47)
2007	East Asia	0.45 (0.42 to 0.49)
2008	East Asia	0.47 (0.43 to 0.51)
2009	East Asia	0.49 (0.44 to 0.53)
2010	East Asia	0.50 (0.45 to 0.55)
2011	East Asia	0.50 (0.45 to 0.55)
2012	East Asia	0.50 (0.44 to 0.55)
2013	East Asia	0.49 (0.43 to 0.55)
2014	East Asia	0.49 (0.43 to 0.55)
2015	East Asia	0.49 (0.43 to 0.56)
2016	East Asia	0.50 (0.43 to 0.58)
2017	East Asia	0.50 (0.42 to 0.60)
2018	East Asia	0.50 (0.42 to 0.59)
2019	East Asia	0.50 (0.41 to 0.60)
2020	East Asia	0.50 (0.41 to 0.62)
2021	East Asia	0.50 (0.41 to 0.63)
1990	Japan	2.92 (2.68 to 3.04)
1991	Japan	3.05 (2.81 to 3.19)
1992	Japan	3.10 (2.84 to 3.23)
1993	Japan	3.21 (2.93 to 3.35)
1994	Japan	3.27 (2.98 to 3.42)
1995	Japan	3.39 (3.09 to 3.56)
1996	Japan	3.44 (3.12 to 3.61)
1997	Japan	3.44 (3.12 to 3.62)
1998	Japan	3.61 (3.26 to 3.80)
1999	Japan	3.69 (3.31 to 3.87)
2000	Japan	3.77 (3.38 to 3.98)
2001	Japan	3.91 (3.49 to 4.14)
2002	Japan	3.96 (3.52 to 4.20)
2003	Japan	4.01 (3.56 to 4.26)
2004	Japan	4.16 (3.68 to 4.43)
2005	Japan	4.25 (3.75 to 4.52)
2006	Japan	4.26 (3.75 to 4.54)

2007	Japan	4.36 (3.82 to 4.65)
2008	Japan	4.50 (3.93 to 4.80)
2009	Japan	4.63 (4.04 to 4.94)
2010	Japan	4.81 (4.19 to 5.15)
2011	Japan	4.90 (4.26 to 5.24)
2012	Japan	4.86 (4.22 to 5.21)
2013	Japan	4.86 (4.20 to 5.23)
2014	Japan	4.88 (4.21 to 5.25)
2015	Japan	4.93 (4.25 to 5.32)
2016	Japan	5.12 (4.42 to 5.53)
2017	Japan	5.19 (4.46 to 5.60)
2018	Japan	5.16 (4.41 to 5.55)
2019	Japan	5.14 (4.39 to 5.53)
2020	Japan	5.01 (4.28 to 5.43)
2021	Japan	5.07 (4.33 to 5.47)
1990	Republic of Korea	1.84 (1.25 to 2.57)
1991	Republic of Korea	1.85 (1.30 to 2.51)
1992	Republic of Korea	1.84 (1.37 to 2.43)
1993	Republic of Korea	1.85 (1.43 to 2.37)
1994	Republic of Korea	1.87 (1.52 to 2.33)
1995	Republic of Korea	1.87 (1.58 to 2.28)
1996	Republic of Korea	1.89 (1.63 to 2.27)
1997	Republic of Korea	1.91 (1.66 to 2.25)
1998	Republic of Korea	1.98 (1.74 to 2.31)
1999	Republic of Korea	2.03 (1.79 to 2.38)
2000	Republic of Korea	2.10 (1.86 to 2.43)
2001	Republic of Korea	2.12 (1.89 to 2.42)
2002	Republic of Korea	2.13 (1.89 to 2.44)
2003	Republic of Korea	2.16 (1.91 to 2.46)
2004	Republic of Korea	2.16 (1.91 to 2.47)
2005	Republic of Korea	2.17 (1.92 to 2.47)
2006	Republic of Korea	2.15 (1.89 to 2.42)
2007	Republic of Korea	2.13 (1.86 to 2.39)
2008	Republic of Korea	2.09 (1.81 to 2.35)
2009	Republic of Korea	2.08 (1.78 to 2.35)
2010	Republic of Korea	2.05 (1.77 to 2.32)
2011	Republic of Korea	2.03 (1.74 to 2.30)
2012	Republic of Korea	1.99 (1.69 to 2.24)
2013	Republic of Korea	1.96 (1.67 to 2.20)
2014	Republic of Korea	1.94 (1.64 to 2.18)
2015	Republic of Korea	1.92 (1.62 to 2.18)
2016	Republic of Korea	1.93 (1.61 to 2.19)

2017	Republic of Korea	1.90 (1.58 to 2.18)
2018	Republic of Korea	1.91 (1.58 to 2.20)
2019	Republic of Korea	1.91 (1.54 to 2.22)
2020	Republic of Korea	1.87 (1.51 to 2.20)
2021	Republic of Korea	1.92 (1.56 to 2.27)
1990	Singapore	2.65 (2.49 to 2.79)
1991	Singapore	2.76 (2.60 to 2.91)
1992	Singapore	2.76 (2.59 to 2.92)
1993	Singapore	2.71 (2.54 to 2.86)
1994	Singapore	2.63 (2.47 to 2.78)
1995	Singapore	2.67 (2.51 to 2.82)
1996	Singapore	2.61 (2.45 to 2.76)
1997	Singapore	2.54 (2.38 to 2.69)
1998	Singapore	2.80 (2.62 to 2.96)
1999	Singapore	2.75 (2.56 to 2.92)
2000	Singapore	2.84 (2.65 to 3.02)
2001	Singapore	2.83 (2.63 to 3.02)
2002	Singapore	2.91 (2.69 to 3.09)
2003	Singapore	2.72 (2.51 to 2.89)
2004	Singapore	2.79 (2.57 to 2.96)
2005	Singapore	2.71 (2.50 to 2.88)
2006	Singapore	2.81 (2.59 to 2.99)
2007	Singapore	2.79 (2.57 to 2.96)
2008	Singapore	2.76 (2.54 to 2.93)
2009	Singapore	2.70 (2.47 to 2.88)
2010	Singapore	2.71 (2.48 to 2.89)
2011	Singapore	2.58 (2.36 to 2.74)
2012	Singapore	2.58 (2.35 to 2.74)
2013	Singapore	2.56 (2.32 to 2.74)
2014	Singapore	2.54 (2.30 to 2.72)
2015	Singapore	2.40 (2.16 to 2.57)
2016	Singapore	2.40 (2.15 to 2.56)
2017	Singapore	2.44 (2.20 to 2.60)
2018	Singapore	2.41 (2.16 to 2.57)
2019	Singapore	2.30 (2.04 to 2.46)
2020	Singapore	2.25 (2.02 to 2.42)
2021	Singapore	2.15 (1.91 to 2.32)
1990	South Asia	0.71 (0.45 to 1.13)
1991	South Asia	0.72 (0.45 to 1.14)
1992	South Asia	0.74 (0.47 to 1.16)
1993	South Asia	0.75 (0.48 to 1.18)
1994	South Asia	0.78 (0.51 to 1.21)

1995	South Asia	0.82 (0.54 to 1.27)
1996	South Asia	0.82 (0.54 to 1.27)
1997	South Asia	0.82 (0.54 to 1.28)
1998	South Asia	0.81 (0.52 to 1.28)
1999	South Asia	0.81 (0.54 to 1.25)
2000	South Asia	0.80 (0.53 to 1.26)
2001	South Asia	0.81 (0.54 to 1.27)
2002	South Asia	0.83 (0.55 to 1.27)
2003	South Asia	0.85 (0.57 to 1.31)
2004	South Asia	0.90 (0.61 to 1.36)
2005	South Asia	0.89 (0.61 to 1.34)
2006	South Asia	0.90 (0.61 to 1.37)
2007	South Asia	0.94 (0.64 to 1.41)
2008	South Asia	0.95 (0.64 to 1.44)
2009	South Asia	0.96 (0.66 to 1.47)
2010	South Asia	0.99 (0.68 to 1.48)
2011	South Asia	1.01 (0.69 to 1.52)
2012	South Asia	1.03 (0.72 to 1.52)
2013	South Asia	1.07 (0.73 to 1.61)
2014	South Asia	1.12 (0.76 to 1.67)
2015	South Asia	1.16 (0.82 to 1.71)
2016	South Asia	1.17 (0.84 to 1.72)
2017	South Asia	1.21 (0.87 to 1.76)
2018	South Asia	1.22 (0.87 to 1.76)
2019	South Asia	1.24 (0.87 to 1.79)
2020	South Asia	1.24 (0.88 to 1.78)
2021	South Asia	1.22 (0.88 to 1.78)
1990	Southeast Asia	1.03 (0.83 to 1.29)
1991	Southeast Asia	1.04 (0.85 to 1.30)
1992	Southeast Asia	1.06 (0.86 to 1.31)
1993	Southeast Asia	1.09 (0.89 to 1.33)
1994	Southeast Asia	1.10 (0.91 to 1.34)
1995	Southeast Asia	1.12 (0.93 to 1.35)
1996	Southeast Asia	1.14 (0.96 to 1.37)
1997	Southeast Asia	1.14 (0.96 to 1.35)
1998	Southeast Asia	1.17 (0.98 to 1.38)
1999	Southeast Asia	1.18 (0.99 to 1.39)
2000	Southeast Asia	1.19 (1.01 to 1.40)
2001	Southeast Asia	1.20 (1.02 to 1.41)
2002	Southeast Asia	1.20 (1.03 to 1.40)
2003	Southeast Asia	1.22 (1.05 to 1.40)
2004	Southeast Asia	1.23 (1.05 to 1.41)

2005	Southeast Asia	1.23 (1.07 to 1.42)
2006	Southeast Asia	1.24 (1.07 to 1.42)
2007	Southeast Asia	1.24 (1.07 to 1.43)
2008	Southeast Asia	1.25 (1.09 to 1.43)
2009	Southeast Asia	1.26 (1.10 to 1.42)
2010	Southeast Asia	1.27 (1.11 to 1.44)
2011	Southeast Asia	1.27 (1.12 to 1.45)
2012	Southeast Asia	1.26 (1.11 to 1.43)
2013	Southeast Asia	1.26 (1.10 to 1.43)
2014	Southeast Asia	1.27 (1.11 to 1.43)
2015	Southeast Asia	1.28 (1.13 to 1.45)
2016	Southeast Asia	1.29 (1.14 to 1.46)
2017	Southeast Asia	1.31 (1.15 to 1.48)
2018	Southeast Asia	1.34 (1.18 to 1.52)
2019	Southeast Asia	1.36 (1.20 to 1.54)
2020	Southeast Asia	1.35 (1.16 to 1.53)
2021	Southeast Asia	1.39 (1.21 to 1.60)

ASDR, age-standardized death rates.

Fig 4B. ASDR in South Asia from 1990 to 2021

Year	Location	ASDR
1990	Nepal	0.64 (0.37 to 1.13)
1991	Nepal	0.65 (0.38 to 1.14)
1992	Nepal	0.65 (0.38 to 1.11)
1993	Nepal	0.66 (0.39 to 1.12)
1994	Nepal	0.67 (0.40 to 1.13)
1995	Nepal	0.68 (0.40 to 1.14)
1996	Nepal	0.68 (0.42 to 1.13)
1997	Nepal	0.68 (0.43 to 1.13)
1998	Nepal	0.69 (0.43 to 1.14)
1999	Nepal	0.70 (0.44 to 1.15)
2000	Nepal	0.70 (0.45 to 1.15)
2001	Nepal	0.71 (0.46 to 1.14)
2002	Nepal	0.72 (0.48 to 1.16)
2003	Nepal	0.73 (0.48 to 1.17)
2004	Nepal	0.74 (0.50 to 1.18)
2005	Nepal	0.76 (0.51 to 1.22)
2006	Nepal	0.78 (0.53 to 1.26)
2007	Nepal	0.81 (0.54 to 1.28)
2008	Nepal	0.83 (0.55 to 1.32)
2009	Nepal	0.86 (0.58 to 1.37)
2010	Nepal	0.89 (0.60 to 1.41)
2011	Nepal	0.93 (0.63 to 1.49)
2012	Nepal	0.95 (0.64 to 1.54)
2013	Nepal	0.99 (0.67 to 1.57)
2014	Nepal	1.01 (0.68 to 1.62)
2015	Nepal	1.06 (0.72 to 1.67)
2016	Nepal	1.08 (0.73 to 1.70)
2017	Nepal	1.12 (0.76 to 1.72)
2018	Nepal	1.14 (0.77 to 1.79)
2019	Nepal	1.16 (0.79 to 1.82)
2020	Nepal	1.17 (0.81 to 1.84)
2021	Nepal	1.19 (0.82 to 1.85)
1990	Pakistan	0.84 (0.57 to 1.29)
1991	Pakistan	0.87 (0.59 to 1.33)
1992	Pakistan	0.91 (0.61 to 1.37)
1993	Pakistan	0.95 (0.64 to 1.42)
1994	Pakistan	1.00 (0.68 to 1.50)
1995	Pakistan	1.05 (0.70 to 1.59)
1996	Pakistan	1.09 (0.75 to 1.61)
1997	Pakistan	1.13 (0.77 to 1.67)

1998	Pakistan	1.16 (0.80 to 1.71)
1999	Pakistan	1.19 (0.82 to 1.74)
2000	Pakistan	1.22 (0.86 to 1.78)
2001	Pakistan	1.26 (0.87 to 1.84)
2002	Pakistan	1.29 (0.90 to 1.87)
2003	Pakistan	1.32 (0.94 to 1.91)
2004	Pakistan	1.35 (0.97 to 1.95)
2005	Pakistan	1.37 (0.97 to 1.95)
2006	Pakistan	1.38 (0.98 to 1.93)
2007	Pakistan	1.39 (0.99 to 1.97)
2008	Pakistan	1.40 (1.01 to 1.98)
2009	Pakistan	1.41 (1.02 to 1.98)
2010	Pakistan	1.42 (1.03 to 1.98)
2011	Pakistan	1.41 (1.02 to 1.95)
2012	Pakistan	1.40 (1.02 to 2.00)
2013	Pakistan	1.40 (1.05 to 1.94)
2014	Pakistan	1.40 (1.01 to 1.97)
2015	Pakistan	1.41 (1.03 to 1.99)
2016	Pakistan	1.42 (1.02 to 1.93)
2017	Pakistan	1.44 (1.04 to 1.97)
2018	Pakistan	1.44 (1.06 to 1.96)
2019	Pakistan	1.45 (1.08 to 2.01)
2020	Pakistan	1.47 (1.06 to 2.01)
2021	Pakistan	1.48 (1.06 to 2.04)
1990	Bhutan	0.78 (0.51 to 1.16)
1991	Bhutan	0.79 (0.53 to 1.15)
1992	Bhutan	0.80 (0.53 to 1.17)
1993	Bhutan	0.82 (0.54 to 1.19)
1994	Bhutan	0.84 (0.56 to 1.21)
1995	Bhutan	0.86 (0.58 to 1.24)
1996	Bhutan	0.86 (0.58 to 1.26)
1997	Bhutan	0.89 (0.61 to 1.28)
1998	Bhutan	0.90 (0.62 to 1.27)
1999	Bhutan	0.90 (0.63 to 1.27)
2000	Bhutan	0.93 (0.66 to 1.31)
2001	Bhutan	0.95 (0.67 to 1.31)
2002	Bhutan	0.97 (0.70 to 1.32)
2003	Bhutan	1.00 (0.73 to 1.37)
2004	Bhutan	1.03 (0.75 to 1.42)
2005	Bhutan	1.06 (0.76 to 1.45)
2006	Bhutan	1.08 (0.76 to 1.50)
2007	Bhutan	1.11 (0.78 to 1.53)

2008	Bhutan	1.15 (0.79 to 1.59)
2009	Bhutan	1.19 (0.82 to 1.66)
2010	Bhutan	1.22 (0.82 to 1.70)
2011	Bhutan	1.26 (0.83 to 1.74)
2012	Bhutan	1.28 (0.84 to 1.77)
2013	Bhutan	1.31 (0.86 to 1.82)
2014	Bhutan	1.33 (0.86 to 1.85)
2015	Bhutan	1.36 (0.87 to 1.93)
2016	Bhutan	1.39 (0.89 to 1.97)
2017	Bhutan	1.42 (0.90 to 2.01)
2018	Bhutan	1.44 (0.90 to 2.05)
2019	Bhutan	1.46 (0.92 to 2.07)
2020	Bhutan	1.48 (0.94 to 2.12)
2021	Bhutan	1.49 (0.93 to 2.16)
1990	Bangladesh	0.82 (0.49 to 1.42)
1991	Bangladesh	0.80 (0.49 to 1.41)
1992	Bangladesh	0.81 (0.50 to 1.42)
1993	Bangladesh	0.82 (0.51 to 1.44)
1994	Bangladesh	0.83 (0.52 to 1.46)
1995	Bangladesh	0.85 (0.53 to 1.49)
1996	Bangladesh	0.86 (0.53 to 1.50)
1997	Bangladesh	0.86 (0.54 to 1.53)
1998	Bangladesh	0.87 (0.55 to 1.54)
1999	Bangladesh	0.92 (0.57 to 1.60)
2000	Bangladesh	0.94 (0.58 to 1.66)
2001	Bangladesh	0.95 (0.58 to 1.70)
2002	Bangladesh	0.98 (0.60 to 1.75)
2003	Bangladesh	1.00 (0.61 to 1.78)
2004	Bangladesh	0.99 (0.61 to 1.78)
2005	Bangladesh	1.00 (0.62 to 1.77)
2006	Bangladesh	1.03 (0.64 to 1.84)
2007	Bangladesh	1.07 (0.67 to 1.89)
2008	Bangladesh	1.09 (0.71 to 1.90)
2009	Bangladesh	1.10 (0.74 to 1.86)
2010	Bangladesh	1.12 (0.74 to 1.88)
2011	Bangladesh	1.05 (0.72 to 1.74)
2012	Bangladesh	0.99 (0.68 to 1.66)
2013	Bangladesh	1.00 (0.68 to 1.67)
2014	Bangladesh	1.04 (0.70 to 1.79)
2015	Bangladesh	1.05 (0.71 to 1.82)
2016	Bangladesh	1.08 (0.72 to 1.89)
2017	Bangladesh	1.15 (0.77 to 2.03)

2018	Bangladesh	1.17 (0.78 to 2.05)
2019	Bangladesh	1.19 (0.79 to 2.04)
2020	Bangladesh	1.20 (0.80 to 2.06)
2021	Bangladesh	1.21 (0.80 to 1.99)
1990	India	0.68 (0.40 to 1.08)
1991	India	0.68 (0.41 to 1.09)
1992	India	0.70 (0.41 to 1.12)
1993	India	0.71 (0.43 to 1.13)
1994	India	0.74 (0.46 to 1.17)
1995	India	0.78 (0.49 to 1.23)
1996	India	0.78 (0.49 to 1.23)
1997	India	0.77 (0.48 to 1.23)
1998	India	0.76 (0.46 to 1.21)
1999	India	0.75 (0.48 to 1.17)
2000	India	0.73 (0.46 to 1.17)
2001	India	0.75 (0.47 to 1.18)
2002	India	0.76 (0.48 to 1.20)
2003	India	0.79 (0.51 to 1.23)
2004	India	0.84 (0.56 to 1.29)
2005	India	0.84 (0.56 to 1.27)
2006	India	0.84 (0.55 to 1.29)
2007	India	0.89 (0.58 to 1.34)
2008	India	0.90 (0.58 to 1.38)
2009	India	0.91 (0.60 to 1.39)
2010	India	0.94 (0.63 to 1.42)
2011	India	0.97 (0.65 to 1.48)
2012	India	1.00 (0.68 to 1.48)
2013	India	1.05 (0.69 to 1.59)
2014	India	1.10 (0.72 to 1.67)
2015	India	1.15 (0.78 to 1.69)
2016	India	1.16 (0.81 to 1.71)
2017	India	1.20 (0.84 to 1.74)
2018	India	1.21 (0.84 to 1.75)
2019	India	1.23 (0.86 to 1.77)
2020	India	1.22 (0.83 to 1.76)
2021	India	1.20 (0.86 to 1.74)

ASDR, age-standardized death rates.

Fig 4C. ASDR in Central Asia from 1990 to 2021

Year	Location	ASDR per 100,000
1990	Armenia	4.55 (3.71 to 5.57)
1991	Armenia	4.75 (3.85 to 5.85)
1992	Armenia	5.18 (4.25 to 6.32)
1993	Armenia	5.42 (4.47 to 6.61)
1994	Armenia	5.24 (4.30 to 6.40)
1995	Armenia	5.11 (4.22 to 6.25)
1996	Armenia	5.13 (4.24 to 6.39)
1997	Armenia	4.89 (4.03 to 6.09)
1998	Armenia	4.80 (3.94 to 5.84)
1999	Armenia	4.86 (4.06 to 5.87)
2000	Armenia	4.80 (4.03 to 5.74)
2001	Armenia	4.88 (4.10 to 5.85)
2002	Armenia	4.97 (4.18 to 5.98)
2003	Armenia	5.14 (4.27 to 6.14)
2004	Armenia	5.31 (4.43 to 6.26)
2005	Armenia	5.70 (4.76 to 6.68)
2006	Armenia	5.93 (4.91 to 6.95)
2007	Armenia	6.08 (5.08 to 7.07)
2008	Armenia	6.25 (5.26 to 7.20)
2009	Armenia	6.67 (5.60 to 7.70)
2010	Armenia	7.58 (6.39 to 8.67)
2011	Armenia	7.79 (6.56 to 8.92)
2012	Armenia	7.88 (6.62 to 9.00)
2013	Armenia	7.95 (6.73 to 9.04)
2014	Armenia	8.35 (7.11 to 9.48)
2015	Armenia	9.02 (7.74 to 10.25)
2016	Armenia	9.02 (7.81 to 10.26)
2017	Armenia	8.55 (7.36 to 9.69)
2018	Armenia	9.26 (8.08 to 10.51)
2019	Armenia	9.06 (7.89 to 10.25)
2020	Armenia	9.10 (7.74 to 10.51)
2021	Armenia	9.16 (7.61 to 10.81)
1990	Azerbaijan	0.73 (0.55 to 0.96)
1991	Azerbaijan	0.74 (0.57 to 0.96)
1992	Azerbaijan	0.77 (0.61 to 0.96)
1993	Azerbaijan	0.77 (0.62 to 0.95)
1994	Azerbaijan	0.80 (0.64 to 1.00)
1995	Azerbaijan	0.77 (0.62 to 0.99)
1996	Azerbaijan	0.77 (0.60 to 0.99)
1997	Azerbaijan	0.75 (0.57 to 0.99)

1998	Azerbaijan	0.75 (0.55 to 1.00)
1999	Azerbaijan	0.75 (0.54 to 1.01)
2000	Azerbaijan	0.75 (0.55 to 1.02)
2001	Azerbaijan	0.77 (0.56 to 1.05)
2002	Azerbaijan	0.83 (0.61 to 1.09)
2003	Azerbaijan	0.90 (0.69 to 1.18)
2004	Azerbaijan	0.97 (0.75 to 1.22)
2005	Azerbaijan	1.04 (0.83 to 1.27)
2006	Azerbaijan	1.08 (0.88 to 1.34)
2007	Azerbaijan	1.14 (0.92 to 1.40)
2008	Azerbaijan	1.19 (0.95 to 1.46)
2009	Azerbaijan	1.22 (0.98 to 1.55)
2010	Azerbaijan	1.26 (0.99 to 1.61)
2011	Azerbaijan	1.27 (0.99 to 1.64)
2012	Azerbaijan	1.29 (1.00 to 1.68)
2013	Azerbaijan	1.31 (1.02 to 1.71)
2014	Azerbaijan	1.30 (0.99 to 1.70)
2015	Azerbaijan	1.33 (1.00 to 1.75)
2016	Azerbaijan	1.32 (0.95 to 1.75)
2017	Azerbaijan	1.35 (0.98 to 1.80)
2018	Azerbaijan	1.31 (0.95 to 1.77)
2019	Azerbaijan	1.37 (0.98 to 1.97)
2020	Azerbaijan	1.34 (0.96 to 1.98)
2021	Azerbaijan	1.37 (0.93 to 2.08)
1990	Georgia	0.61 (0.51 to 0.72)
1991	Georgia	0.61 (0.52 to 0.73)
1992	Georgia	0.61 (0.52 to 0.73)
1993	Georgia	0.58 (0.50 to 0.69)
1994	Georgia	0.56 (0.49 to 0.66)
1995	Georgia	0.56 (0.49 to 0.66)
1996	Georgia	0.59 (0.51 to 0.68)
1997	Georgia	0.59 (0.50 to 0.70)
1998	Georgia	0.61 (0.52 to 0.71)
1999	Georgia	0.37 (0.32 to 0.43)
2000	Georgia	0.44 (0.40 to 0.49)
2001	Georgia	0.40 (0.36 to 0.44)
2002	Georgia	0.45 (0.41 to 0.48)
2003	Georgia	0.62 (0.57 to 0.68)
2004	Georgia	0.71 (0.64 to 0.78)
2005	Georgia	0.72 (0.63 to 0.79)
2006	Georgia	0.81 (0.71 to 0.91)
2007	Georgia	1.09 (0.96 to 1.23)

2008	Georgia	1.36 (1.18 to 1.54)
2009	Georgia	1.41 (1.22 to 1.60)
2010	Georgia	1.59 (1.36 to 1.78)
2011	Georgia	2.05 (1.78 to 2.31)
2012	Georgia	2.26 (1.96 to 2.54)
2013	Georgia	2.43 (2.11 to 2.73)
2014	Georgia	2.52 (2.19 to 2.85)
2015	Georgia	2.75 (2.40 to 3.13)
2016	Georgia	2.93 (2.58 to 3.31)
2017	Georgia	2.73 (2.40 to 3.10)
2018	Georgia	2.96 (2.58 to 3.35)
2019	Georgia	2.89 (2.50 to 3.27)
2020	Georgia	2.99 (2.58 to 3.40)
2021	Georgia	2.88 (2.43 to 3.35)
1990	Kazakhstan	1.46 (1.19 to 1.87)
1991	Kazakhstan	1.61 (1.32 to 2.04)
1992	Kazakhstan	1.76 (1.44 to 2.18)
1993	Kazakhstan	1.97 (1.62 to 2.43)
1994	Kazakhstan	2.10 (1.71 to 2.58)
1995	Kazakhstan	2.31 (1.88 to 2.82)
1996	Kazakhstan	2.33 (1.91 to 2.84)
1997	Kazakhstan	2.29 (1.85 to 2.81)
1998	Kazakhstan	2.24 (1.81 to 2.74)
1999	Kazakhstan	2.15 (1.73 to 2.62)
2000	Kazakhstan	2.25 (1.82 to 2.76)
2001	Kazakhstan	2.26 (1.82 to 2.77)
2002	Kazakhstan	2.28 (1.84 to 2.80)
2003	Kazakhstan	2.36 (1.93 to 2.86)
2004	Kazakhstan	2.28 (1.89 to 2.73)
2005	Kazakhstan	2.28 (1.89 to 2.72)
2006	Kazakhstan	2.29 (1.91 to 2.70)
2007	Kazakhstan	2.30 (1.94 to 2.69)
2008	Kazakhstan	2.16 (1.82 to 2.50)
2009	Kazakhstan	2.06 (1.75 to 2.38)
2010	Kazakhstan	2.12 (1.83 to 2.41)
2011	Kazakhstan	2.10 (1.81 to 2.37)
2012	Kazakhstan	2.12 (1.85 to 2.40)
2013	Kazakhstan	2.09 (1.82 to 2.41)
2014	Kazakhstan	2.11 (1.82 to 2.42)
2015	Kazakhstan	2.18 (1.88 to 2.51)
2016	Kazakhstan	2.25 (1.92 to 2.60)
2017	Kazakhstan	2.29 (1.94 to 2.65)

2018	Kazakhstan	2.31 (1.94 to 2.73)
2019	Kazakhstan	2.32 (1.94 to 2.76)
2020	Kazakhstan	2.28 (1.87 to 2.72)
2021	Kazakhstan	2.23 (1.80 to 2.71)
1990	Kyrgyzstan	0.28 (0.24 to 0.33)
1991	Kyrgyzstan	0.29 (0.25 to 0.34)
1992	Kyrgyzstan	0.30 (0.26 to 0.34)
1993	Kyrgyzstan	0.32 (0.28 to 0.36)
1994	Kyrgyzstan	0.33 (0.30 to 0.37)
1995	Kyrgyzstan	0.33 (0.30 to 0.37)
1996	Kyrgyzstan	0.32 (0.29 to 0.36)
1997	Kyrgyzstan	0.32 (0.28 to 0.36)
1998	Kyrgyzstan	0.31 (0.28 to 0.33)
1999	Kyrgyzstan	0.30 (0.28 to 0.32)
2000	Kyrgyzstan	0.31 (0.29 to 0.33)
2001	Kyrgyzstan	0.28 (0.26 to 0.31)
2002	Kyrgyzstan	0.47 (0.44 to 0.51)
2003	Kyrgyzstan	0.61 (0.56 to 0.66)
2004	Kyrgyzstan	0.42 (0.38 to 0.46)
2005	Kyrgyzstan	0.41 (0.38 to 0.45)
2006	Kyrgyzstan	0.46 (0.42 to 0.51)
2007	Kyrgyzstan	0.57 (0.51 to 0.63)
2008	Kyrgyzstan	0.63 (0.56 to 0.70)
2009	Kyrgyzstan	0.50 (0.44 to 0.56)
2010	Kyrgyzstan	0.43 (0.37 to 0.48)
2011	Kyrgyzstan	0.44 (0.39 to 0.49)
2012	Kyrgyzstan	0.62 (0.55 to 0.69)
2013	Kyrgyzstan	0.69 (0.61 to 0.77)
2014	Kyrgyzstan	0.73 (0.65 to 0.82)
2015	Kyrgyzstan	0.79 (0.70 to 0.89)
2016	Kyrgyzstan	0.81 (0.72 to 0.92)
2017	Kyrgyzstan	0.81 (0.71 to 0.91)
2018	Kyrgyzstan	0.84 (0.73 to 0.96)
2019	Kyrgyzstan	0.81 (0.70 to 0.94)
2020	Kyrgyzstan	0.81 (0.67 to 0.95)
2021	Kyrgyzstan	0.79 (0.63 to 0.96)
1990	Mongolia	0.31 (0.22 to 0.42)
1991	Mongolia	0.32 (0.23 to 0.43)
1992	Mongolia	0.33 (0.23 to 0.44)
1993	Mongolia	0.33 (0.23 to 0.46)
1994	Mongolia	0.33 (0.23 to 0.45)
1995	Mongolia	0.34 (0.24 to 0.47)

1996	Mongolia	0.35 (0.25 to 0.47)
1997	Mongolia	0.35 (0.25 to 0.46)
1998	Mongolia	0.35 (0.25 to 0.46)
1999	Mongolia	0.34 (0.26 to 0.46)
2000	Mongolia	0.34 (0.26 to 0.46)
2001	Mongolia	0.34 (0.25 to 0.45)
2002	Mongolia	0.36 (0.26 to 0.48)
2003	Mongolia	0.37 (0.28 to 0.50)
2004	Mongolia	0.39 (0.29 to 0.53)
2005	Mongolia	0.40 (0.30 to 0.55)
2006	Mongolia	0.41 (0.30 to 0.55)
2007	Mongolia	0.41 (0.30 to 0.56)
2008	Mongolia	0.41 (0.30 to 0.55)
2009	Mongolia	0.42 (0.31 to 0.57)
2010	Mongolia	0.41 (0.30 to 0.56)
2011	Mongolia	0.46 (0.33 to 0.62)
2012	Mongolia	0.50 (0.37 to 0.65)
2013	Mongolia	0.54 (0.40 to 0.70)
2014	Mongolia	0.58 (0.44 to 0.78)
2015	Mongolia	0.61 (0.47 to 0.81)
2016	Mongolia	0.64 (0.49 to 0.83)
2017	Mongolia	0.67 (0.52 to 0.86)
2018	Mongolia	0.70 (0.54 to 0.90)
2019	Mongolia	0.72 (0.55 to 0.92)
2020	Mongolia	0.70 (0.54 to 0.90)
2021	Mongolia	0.70 (0.53 to 0.89)
1990	Tajikistan	0.28 (0.19 to 0.36)
1991	Tajikistan	0.29 (0.20 to 0.38)
1992	Tajikistan	0.30 (0.22 to 0.39)
1993	Tajikistan	0.29 (0.22 to 0.36)
1994	Tajikistan	0.29 (0.23 to 0.36)
1995	Tajikistan	0.30 (0.23 to 0.37)
1996	Tajikistan	0.29 (0.22 to 0.36)
1997	Tajikistan	0.27 (0.21 to 0.33)
1998	Tajikistan	0.27 (0.20 to 0.34)
1999	Tajikistan	0.27 (0.20 to 0.35)
2000	Tajikistan	0.27 (0.19 to 0.35)
2001	Tajikistan	0.26 (0.20 to 0.35)
2002	Tajikistan	0.27 (0.20 to 0.36)
2003	Tajikistan	0.28 (0.21 to 0.37)
2004	Tajikistan	0.29 (0.22 to 0.37)
2005	Tajikistan	0.30 (0.22 to 0.38)

2006	Tajikistan	0.30 (0.23 to 0.38)
2007	Tajikistan	0.31 (0.25 to 0.39)
2008	Tajikistan	0.31 (0.25 to 0.40)
2009	Tajikistan	0.32 (0.25 to 0.41)
2010	Tajikistan	0.30 (0.23 to 0.38)
2011	Tajikistan	0.29 (0.23 to 0.37)
2012	Tajikistan	0.28 (0.22 to 0.35)
2013	Tajikistan	0.26 (0.20 to 0.34)
2014	Tajikistan	0.26 (0.20 to 0.33)
2015	Tajikistan	0.25 (0.20 to 0.32)
2016	Tajikistan	0.25 (0.19 to 0.31)
2017	Tajikistan	0.25 (0.19 to 0.31)
2018	Tajikistan	0.24 (0.19 to 0.32)
2019	Tajikistan	0.24 (0.19 to 0.31)
2020	Tajikistan	0.24 (0.18 to 0.32)
2021	Tajikistan	0.25 (0.18 to 0.34)
1990	Turkmenistan	1.03 (0.84 to 1.26)
1991	Turkmenistan	1.08 (0.89 to 1.33)
1992	Turkmenistan	1.08 (0.90 to 1.32)
1993	Turkmenistan	1.21 (1.02 to 1.49)
1994	Turkmenistan	1.22 (1.03 to 1.48)
1995	Turkmenistan	1.19 (1.00 to 1.43)
1996	Turkmenistan	1.22 (1.04 to 1.47)
1997	Turkmenistan	1.21 (1.04 to 1.47)
1998	Turkmenistan	1.22 (1.03 to 1.47)
1999	Turkmenistan	1.26 (1.07 to 1.50)
2000	Turkmenistan	1.29 (1.08 to 1.52)
2001	Turkmenistan	1.27 (1.07 to 1.51)
2002	Turkmenistan	1.33 (1.12 to 1.57)
2003	Turkmenistan	1.37 (1.16 to 1.60)
2004	Turkmenistan	1.46 (1.25 to 1.69)
2005	Turkmenistan	1.58 (1.34 to 1.83)
2006	Turkmenistan	1.60 (1.37 to 1.83)
2007	Turkmenistan	1.61 (1.37 to 1.85)
2008	Turkmenistan	1.57 (1.34 to 1.84)
2009	Turkmenistan	1.48 (1.25 to 1.74)
2010	Turkmenistan	1.47 (1.24 to 1.74)
2011	Turkmenistan	1.50 (1.25 to 1.77)
2012	Turkmenistan	1.52 (1.26 to 1.81)
2013	Turkmenistan	1.62 (1.35 to 1.94)
2014	Turkmenistan	1.65 (1.37 to 1.99)
2015	Turkmenistan	1.69 (1.39 to 2.08)

2016	Turkmenistan	1.72 (1.39 to 2.17)
2017	Turkmenistan	1.78 (1.42 to 2.31)
2018	Turkmenistan	1.82 (1.40 to 2.44)
2019	Turkmenistan	1.87 (1.41 to 2.58)
2020	Turkmenistan	1.88 (1.43 to 2.63)
2021	Turkmenistan	1.88 (1.42 to 2.67)
1990	Uzbekistan	0.21 (0.16 to 0.30)
1991	Uzbekistan	0.22 (0.17 to 0.31)
1992	Uzbekistan	0.23 (0.17 to 0.31)
1993	Uzbekistan	0.24 (0.18 to 0.32)
1994	Uzbekistan	0.25 (0.19 to 0.34)
1995	Uzbekistan	0.26 (0.19 to 0.34)
1996	Uzbekistan	0.26 (0.20 to 0.35)
1997	Uzbekistan	0.26 (0.20 to 0.34)
1998	Uzbekistan	0.26 (0.20 to 0.34)
1999	Uzbekistan	0.26 (0.21 to 0.34)
2000	Uzbekistan	0.27 (0.22 to 0.34)
2001	Uzbekistan	0.27 (0.22 to 0.34)
2002	Uzbekistan	0.28 (0.23 to 0.35)
2003	Uzbekistan	0.29 (0.24 to 0.36)
2004	Uzbekistan	0.30 (0.25 to 0.37)
2005	Uzbekistan	0.33 (0.27 to 0.40)
2006	Uzbekistan	0.36 (0.29 to 0.43)
2007	Uzbekistan	0.37 (0.30 to 0.45)
2008	Uzbekistan	0.38 (0.31 to 0.45)
2009	Uzbekistan	0.37 (0.31 to 0.45)
2010	Uzbekistan	0.38 (0.31 to 0.45)
2011	Uzbekistan	0.38 (0.32 to 0.45)
2012	Uzbekistan	0.52 (0.44 to 0.61)
2013	Uzbekistan	0.62 (0.53 to 0.72)
2014	Uzbekistan	0.75 (0.65 to 0.88)
2015	Uzbekistan	0.90 (0.77 to 1.05)
2016	Uzbekistan	0.99 (0.84 to 1.15)
2017	Uzbekistan	1.03 (0.87 to 1.19)
2018	Uzbekistan	1.08 (0.91 to 1.25)
2019	Uzbekistan	1.04 (0.89 to 1.22)
2020	Uzbekistan	1.04 (0.86 to 1.25)
2021	Uzbekistan	1.04 (0.85 to 1.28)

ASDR, age-standardized death rates.

Fig 4D. ASDR in Southeast Asia from 1990 to 2021

Year	Location	ASDR per 100,000
1990	Cambodia	0.55 (0.32 to 0.87)
1991	Cambodia	0.55 (0.33 to 0.85)
1992	Cambodia	0.56 (0.33 to 0.85)
1993	Cambodia	0.56 (0.34 to 0.86)
1994	Cambodia	0.57 (0.34 to 0.86)
1995	Cambodia	0.57 (0.35 to 0.86)
1996	Cambodia	0.57 (0.35 to 0.85)
1997	Cambodia	0.57 (0.36 to 0.85)
1998	Cambodia	0.57 (0.36 to 0.84)
1999	Cambodia	0.57 (0.37 to 0.84)
2000	Cambodia	0.57 (0.37 to 0.84)
2001	Cambodia	0.57 (0.38 to 0.84)
2002	Cambodia	0.57 (0.38 to 0.84)
2003	Cambodia	0.57 (0.39 to 0.83)
2004	Cambodia	0.58 (0.39 to 0.82)
2005	Cambodia	0.58 (0.40 to 0.82)
2006	Cambodia	0.59 (0.40 to 0.83)
2007	Cambodia	0.60 (0.41 to 0.85)
2008	Cambodia	0.61 (0.42 to 0.86)
2009	Cambodia	0.63 (0.43 to 0.86)
2010	Cambodia	0.64 (0.43 to 0.88)
2011	Cambodia	0.65 (0.44 to 0.91)
2012	Cambodia	0.66 (0.44 to 0.92)
2013	Cambodia	0.67 (0.45 to 0.93)
2014	Cambodia	0.68 (0.45 to 0.96)
2015	Cambodia	0.70 (0.46 to 0.99)
2016	Cambodia	0.71 (0.47 to 1.04)
2017	Cambodia	0.72 (0.47 to 1.07)
2018	Cambodia	0.73 (0.48 to 1.11)
2019	Cambodia	0.74 (0.48 to 1.15)
2020	Cambodia	0.74 (0.48 to 1.17)
2021	Cambodia	0.75 (0.47 to 1.21)
1990	Indonesia	0.63 (0.41 to 0.87)
1991	Indonesia	0.65 (0.42 to 0.89)
1992	Indonesia	0.67 (0.44 to 0.90)
1993	Indonesia	0.69 (0.46 to 0.92)
1994	Indonesia	0.71 (0.49 to 0.95)
1995	Indonesia	0.72 (0.49 to 0.95)
1996	Indonesia	0.74 (0.51 to 0.97)
1997	Indonesia	0.75 (0.52 to 0.99)

1998	Indonesia	0.77 (0.53 to 1.00)
1999	Indonesia	0.78 (0.54 to 1.04)
2000	Indonesia	0.80 (0.56 to 1.04)
2001	Indonesia	0.81 (0.56 to 1.07)
2002	Indonesia	0.82 (0.57 to 1.07)
2003	Indonesia	0.83 (0.58 to 1.09)
2004	Indonesia	0.85 (0.59 to 1.12)
2005	Indonesia	0.86 (0.60 to 1.12)
2006	Indonesia	0.87 (0.61 to 1.14)
2007	Indonesia	0.88 (0.61 to 1.17)
2008	Indonesia	0.89 (0.61 to 1.18)
2009	Indonesia	0.89 (0.62 to 1.19)
2010	Indonesia	0.90 (0.62 to 1.20)
2011	Indonesia	0.92 (0.62 to 1.23)
2012	Indonesia	0.92 (0.64 to 1.24)
2013	Indonesia	0.93 (0.64 to 1.24)
2014	Indonesia	0.94 (0.65 to 1.27)
2015	Indonesia	0.95 (0.65 to 1.28)
2016	Indonesia	0.97 (0.65 to 1.28)
2017	Indonesia	0.98 (0.67 to 1.32)
2018	Indonesia	0.99 (0.68 to 1.34)
2019	Indonesia	1.01 (0.68 to 1.34)
2020	Indonesia	1.02 (0.68 to 1.38)
2021	Indonesia	1.03 (0.71 to 1.36)
1990	Lao People's Democratic Republic	0.78 (0.51 to 1.22)
1991	Lao People's Democratic Republic	0.79 (0.51 to 1.21)
1992	Lao People's Democratic Republic	0.79 (0.52 to 1.21)
1993	Lao People's Democratic Republic	0.80 (0.52 to 1.20)
1994	Lao People's Democratic Republic	0.80 (0.53 to 1.19)
1995	Lao People's Democratic Republic	0.80 (0.54 to 1.18)
1996	Lao People's Democratic Republic	0.81 (0.55 to 1.17)
1997	Lao People's Democratic Republic	0.81 (0.56 to 1.17)
1998	Lao People's Democratic Republic	0.82 (0.57 to 1.16)
1999	Lao People's Democratic Republic	0.82 (0.58 to 1.16)
2000	Lao People's Democratic Republic	0.82 (0.58 to 1.13)
2001	Lao People's Democratic Republic	0.83 (0.60 to 1.13)
2002	Lao People's Democratic Republic	0.82 (0.60 to 1.11)
2003	Lao People's Democratic Republic	0.83 (0.60 to 1.09)
2004	Lao People's Democratic Republic	0.83 (0.61 to 1.09)
2005	Lao People's Democratic Republic	0.83 (0.61 to 1.09)
2006	Lao People's Democratic Republic	0.84 (0.61 to 1.11)
2007	Lao People's Democratic Republic	0.84 (0.62 to 1.11)

2008	Lao People's Democratic Republic	0.85 (0.64 to 1.13)
2009	Lao People's Democratic Republic	0.86 (0.65 to 1.12)
2010	Lao People's Democratic Republic	0.86 (0.66 to 1.12)
2011	Lao People's Democratic Republic	0.87 (0.67 to 1.11)
2012	Lao People's Democratic Republic	0.87 (0.66 to 1.14)
2013	Lao People's Democratic Republic	0.88 (0.67 to 1.16)
2014	Lao People's Democratic Republic	0.89 (0.67 to 1.20)
2015	Lao People's Democratic Republic	0.90 (0.68 to 1.21)
2016	Lao People's Democratic Republic	0.90 (0.68 to 1.23)
2017	Lao People's Democratic Republic	0.91 (0.69 to 1.26)
2018	Lao People's Democratic Republic	0.91 (0.69 to 1.25)
2019	Lao People's Democratic Republic	0.92 (0.70 to 1.24)
2020	Lao People's Democratic Republic	0.92 (0.69 to 1.27)
2021	Lao People's Democratic Republic	0.93 (0.68 to 1.28)
1990	Malaysia	3.30 (2.66 to 4.00)
1991	Malaysia	3.26 (2.66 to 3.96)
1992	Malaysia	3.35 (2.74 to 4.01)
1993	Malaysia	3.51 (2.87 to 4.14)
1994	Malaysia	3.49 (2.92 to 4.21)
1995	Malaysia	3.67 (3.09 to 4.41)
1996	Malaysia	3.81 (3.24 to 4.45)
1997	Malaysia	3.94 (3.35 to 4.51)
1998	Malaysia	4.15 (3.54 to 4.74)
1999	Malaysia	4.45 (3.90 to 5.01)
2000	Malaysia	4.35 (3.79 to 4.87)
2001	Malaysia	4.21 (3.75 to 4.71)
2002	Malaysia	4.08 (3.66 to 4.48)
2003	Malaysia	4.38 (3.94 to 4.83)
2004	Malaysia	4.40 (3.96 to 4.85)
2005	Malaysia	4.32 (3.88 to 4.74)
2006	Malaysia	4.18 (3.73 to 4.60)
2007	Malaysia	4.11 (3.67 to 4.54)
2008	Malaysia	4.17 (3.73 to 4.59)
2009	Malaysia	4.26 (3.81 to 4.72)
2010	Malaysia	4.27 (3.69 to 4.83)
2011	Malaysia	4.13 (3.62 to 4.71)
2012	Malaysia	3.99 (3.56 to 4.48)
2013	Malaysia	3.92 (3.41 to 4.50)
2014	Malaysia	3.95 (3.46 to 4.49)
2015	Malaysia	4.02 (3.52 to 4.57)
2016	Malaysia	3.97 (3.43 to 4.59)
2017	Malaysia	3.97 (3.42 to 4.59)

2018	Malaysia	3.97 (3.41 to 4.65)
2019	Malaysia	4.01 (3.40 to 4.74)
2020	Malaysia	3.43 (2.87 to 4.08)
2021	Malaysia	4.04 (3.38 to 4.88)
1990	Maldives	0.66 (0.47 to 0.91)
1991	Maldives	0.68 (0.49 to 0.92)
1992	Maldives	0.70 (0.51 to 0.94)
1993	Maldives	0.72 (0.51 to 0.96)
1994	Maldives	0.73 (0.51 to 0.98)
1995	Maldives	0.74 (0.53 to 1.00)
1996	Maldives	0.74 (0.56 to 0.98)
1997	Maldives	0.76 (0.57 to 0.99)
1998	Maldives	0.76 (0.56 to 0.99)
1999	Maldives	0.73 (0.56 to 0.93)
2000	Maldives	0.74 (0.57 to 0.94)
2001	Maldives	0.73 (0.55 to 0.93)
2002	Maldives	0.71 (0.53 to 0.93)
2003	Maldives	0.70 (0.51 to 0.92)
2004	Maldives	0.69 (0.50 to 0.92)
2005	Maldives	0.68 (0.48 to 0.90)
2006	Maldives	0.67 (0.46 to 0.89)
2007	Maldives	0.66 (0.45 to 0.87)
2008	Maldives	0.65 (0.44 to 0.85)
2009	Maldives	0.64 (0.42 to 0.85)
2010	Maldives	0.63 (0.41 to 0.84)
2011	Maldives	0.62 (0.40 to 0.83)
2012	Maldives	0.62 (0.39 to 0.83)
2013	Maldives	0.60 (0.38 to 0.81)
2014	Maldives	0.60 (0.37 to 0.82)
2015	Maldives	0.61 (0.36 to 0.85)
2016	Maldives	0.60 (0.35 to 0.85)
2017	Maldives	0.57 (0.34 to 0.79)
2018	Maldives	0.60 (0.33 to 0.88)
2019	Maldives	0.61 (0.32 to 0.95)
2020	Maldives	0.59 (0.30 to 0.94)
2021	Maldives	0.60 (0.31 to 0.97)
1990	Mauritius	1.50 (1.40 to 1.60)
1991	Mauritius	1.42 (1.31 to 1.52)
1992	Mauritius	1.25 (1.17 to 1.34)
1993	Mauritius	1.03 (0.97 to 1.11)
1994	Mauritius	1.11 (1.03 to 1.18)
1995	Mauritius	1.07 (0.99 to 1.15)

1996	Mauritius	0.85 (0.79 to 0.91)
1997	Mauritius	0.99 (0.92 to 1.05)
1998	Mauritius	0.91 (0.85 to 0.97)
1999	Mauritius	0.86 (0.79 to 0.91)
2000	Mauritius	1.02 (0.95 to 1.09)
2001	Mauritius	1.11 (1.03 to 1.17)
2002	Mauritius	1.05 (0.98 to 1.11)
2003	Mauritius	1.06 (0.99 to 1.12)
2004	Mauritius	1.05 (0.99 to 1.11)
2005	Mauritius	0.95 (0.88 to 1.00)
2006	Mauritius	0.87 (0.81 to 0.92)
2007	Mauritius	0.80 (0.75 to 0.85)
2008	Mauritius	0.81 (0.76 to 0.86)
2009	Mauritius	0.84 (0.78 to 0.88)
2010	Mauritius	0.84 (0.78 to 0.89)
2011	Mauritius	0.80 (0.74 to 0.85)
2012	Mauritius	0.76 (0.70 to 0.80)
2013	Mauritius	0.76 (0.70 to 0.81)
2014	Mauritius	0.78 (0.72 to 0.83)
2015	Mauritius	0.75 (0.70 to 0.80)
2016	Mauritius	0.82 (0.76 to 0.88)
2017	Mauritius	0.83 (0.77 to 0.89)
2018	Mauritius	0.86 (0.80 to 0.92)
2019	Mauritius	0.86 (0.79 to 0.92)
2020	Mauritius	0.83 (0.76 to 0.89)
2021	Mauritius	0.88 (0.81 to 0.94)
1990	Myanmar	0.80 (0.47 to 1.16)
1991	Myanmar	0.80 (0.47 to 1.19)
1992	Myanmar	0.80 (0.48 to 1.20)
1993	Myanmar	0.81 (0.49 to 1.21)
1994	Myanmar	0.81 (0.50 to 1.21)
1995	Myanmar	0.82 (0.52 to 1.21)
1996	Myanmar	0.83 (0.52 to 1.20)
1997	Myanmar	0.83 (0.52 to 1.21)
1998	Myanmar	0.84 (0.53 to 1.21)
1999	Myanmar	0.84 (0.55 to 1.21)
2000	Myanmar	0.85 (0.56 to 1.21)
2001	Myanmar	0.86 (0.57 to 1.23)
2002	Myanmar	0.86 (0.57 to 1.21)
2003	Myanmar	0.86 (0.57 to 1.20)
2004	Myanmar	0.87 (0.58 to 1.21)
2005	Myanmar	0.87 (0.60 to 1.19)

2006	Myanmar	0.87 (0.61 to 1.19)
2007	Myanmar	0.87 (0.63 to 1.19)
2008	Myanmar	0.87 (0.63 to 1.19)
2009	Myanmar	0.87 (0.64 to 1.19)
2010	Myanmar	0.87 (0.65 to 1.19)
2011	Myanmar	0.89 (0.66 to 1.20)
2012	Myanmar	0.88 (0.66 to 1.18)
2013	Myanmar	0.89 (0.67 to 1.18)
2014	Myanmar	0.90 (0.69 to 1.18)
2015	Myanmar	0.91 (0.70 to 1.17)
2016	Myanmar	0.92 (0.71 to 1.17)
2017	Myanmar	0.93 (0.72 to 1.20)
2018	Myanmar	0.94 (0.71 to 1.23)
2019	Myanmar	0.95 (0.72 to 1.23)
2020	Myanmar	0.95 (0.72 to 1.25)
2021	Myanmar	0.96 (0.73 to 1.26)
1990	Philippines	1.22 (1.07 to 1.39)
1991	Philippines	1.18 (1.02 to 1.35)
1992	Philippines	1.16 (1.02 to 1.31)
1993	Philippines	1.16 (1.03 to 1.31)
1994	Philippines	1.18 (1.05 to 1.32)
1995	Philippines	1.19 (1.06 to 1.33)
1996	Philippines	1.22 (1.09 to 1.35)
1997	Philippines	1.22 (1.08 to 1.36)
1998	Philippines	1.24 (1.10 to 1.36)
1999	Philippines	1.24 (1.11 to 1.38)
2000	Philippines	1.26 (1.14 to 1.38)
2001	Philippines	1.28 (1.15 to 1.40)
2002	Philippines	1.27 (1.17 to 1.37)
2003	Philippines	1.26 (1.15 to 1.36)
2004	Philippines	1.25 (1.13 to 1.37)
2005	Philippines	1.25 (1.14 to 1.35)
2006	Philippines	1.25 (1.14 to 1.34)
2007	Philippines	1.25 (1.14 to 1.36)
2008	Philippines	1.26 (1.15 to 1.36)
2009	Philippines	1.27 (1.16 to 1.37)
2010	Philippines	1.27 (1.16 to 1.38)
2011	Philippines	1.28 (1.18 to 1.39)
2012	Philippines	1.28 (1.16 to 1.38)
2013	Philippines	1.28 (1.16 to 1.40)
2014	Philippines	1.26 (1.17 to 1.37)
2015	Philippines	1.32 (1.21 to 1.45)

2016	Philippines	1.32 (1.20 to 1.42)
2017	Philippines	1.32 (1.21 to 1.42)
2018	Philippines	1.33 (1.20 to 1.46)
2019	Philippines	1.34 (1.21 to 1.49)
2020	Philippines	1.34 (1.20 to 1.47)
2021	Philippines	1.35 (1.12 to 1.58)
1990	Seychelles	1.08 (0.88 to 1.29)
1991	Seychelles	1.09 (0.90 to 1.30)
1992	Seychelles	1.10 (0.92 to 1.31)
1993	Seychelles	1.12 (0.93 to 1.33)
1994	Seychelles	1.14 (0.95 to 1.35)
1995	Seychelles	1.15 (0.96 to 1.37)
1996	Seychelles	1.16 (0.96 to 1.39)
1997	Seychelles	1.16 (0.96 to 1.43)
1998	Seychelles	1.16 (0.94 to 1.45)
1999	Seychelles	1.16 (0.92 to 1.46)
2000	Seychelles	1.14 (0.89 to 1.46)
2001	Seychelles	1.13 (0.88 to 1.45)
2002	Seychelles	1.17 (0.90 to 1.49)
2003	Seychelles	1.21 (0.94 to 1.54)
2004	Seychelles	1.22 (0.95 to 1.57)
2005	Seychelles	1.25 (0.97 to 1.58)
2006	Seychelles	1.24 (0.95 to 1.57)
2007	Seychelles	1.23 (0.93 to 1.56)
2008	Seychelles	1.21 (0.92 to 1.54)
2009	Seychelles	1.22 (0.93 to 1.55)
2010	Seychelles	1.18 (0.89 to 1.54)
2011	Seychelles	1.16 (0.85 to 1.55)
2012	Seychelles	1.16 (0.87 to 1.51)
2013	Seychelles	1.16 (0.86 to 1.54)
2014	Seychelles	1.15 (0.85 to 1.54)
2015	Seychelles	1.16 (0.84 to 1.57)
2016	Seychelles	1.18 (0.84 to 1.59)
2017	Seychelles	1.20 (0.85 to 1.62)
2018	Seychelles	1.21 (0.85 to 1.65)
2019	Seychelles	1.21 (0.85 to 1.68)
2020	Seychelles	1.01 (0.70 to 1.42)
2021	Seychelles	1.07 (0.74 to 1.51)
1990	Sri Lanka	0.35 (0.29 to 0.42)
1991	Sri Lanka	0.33 (0.28 to 0.38)
1992	Sri Lanka	0.33 (0.28 to 0.38)
1993	Sri Lanka	0.32 (0.27 to 0.38)

1994	Sri Lanka	0.33 (0.28 to 0.38)
1995	Sri Lanka	0.34 (0.29 to 0.39)
1996	Sri Lanka	0.33 (0.28 to 0.39)
1997	Sri Lanka	0.35 (0.30 to 0.40)
1998	Sri Lanka	0.34 (0.29 to 0.39)
1999	Sri Lanka	0.34 (0.30 to 0.40)
2000	Sri Lanka	0.35 (0.30 to 0.40)
2001	Sri Lanka	0.34 (0.30 to 0.39)
2002	Sri Lanka	0.34 (0.30 to 0.39)
2003	Sri Lanka	0.36 (0.32 to 0.41)
2004	Sri Lanka	0.37 (0.32 to 0.42)
2005	Sri Lanka	0.39 (0.34 to 0.44)
2006	Sri Lanka	0.40 (0.35 to 0.45)
2007	Sri Lanka	0.40 (0.36 to 0.46)
2008	Sri Lanka	0.41 (0.36 to 0.45)
2009	Sri Lanka	0.40 (0.35 to 0.45)
2010	Sri Lanka	0.39 (0.35 to 0.44)
2011	Sri Lanka	0.40 (0.36 to 0.45)
2012	Sri Lanka	0.40 (0.35 to 0.44)
2013	Sri Lanka	0.40 (0.36 to 0.45)
2014	Sri Lanka	0.40 (0.36 to 0.45)
2015	Sri Lanka	0.41 (0.34 to 0.47)
2016	Sri Lanka	0.39 (0.31 to 0.48)
2017	Sri Lanka	0.39 (0.30 to 0.49)
2018	Sri Lanka	0.39 (0.29 to 0.51)
2019	Sri Lanka	0.38 (0.27 to 0.50)
2020	Sri Lanka	0.34 (0.20 to 0.49)
2021	Sri Lanka	0.37 (0.25 to 0.50)
1990	Thailand	2.02 (1.53 to 2.71)
1991	Thailand	2.07 (1.58 to 2.72)
1992	Thailand	2.11 (1.58 to 2.75)
1993	Thailand	2.16 (1.64 to 2.84)
1994	Thailand	2.18 (1.66 to 2.83)
1995	Thailand	2.19 (1.67 to 2.82)
1996	Thailand	2.23 (1.71 to 2.82)
1997	Thailand	2.14 (1.65 to 2.74)
1998	Thailand	2.19 (1.65 to 2.82)
1999	Thailand	2.15 (1.62 to 2.73)
2000	Thailand	2.15 (1.68 to 2.69)
2001	Thailand	2.14 (1.71 to 2.67)
2002	Thailand	2.16 (1.74 to 2.66)
2003	Thailand	2.15 (1.72 to 2.65)

2004	Thailand	2.16 (1.74 to 2.58)
2005	Thailand	2.13 (1.74 to 2.59)
2006	Thailand	2.11 (1.71 to 2.61)
2007	Thailand	2.08 (1.68 to 2.52)
2008	Thailand	2.06 (1.71 to 2.45)
2009	Thailand	2.02 (1.69 to 2.40)
2010	Thailand	2.01 (1.69 to 2.39)
2011	Thailand	1.98 (1.66 to 2.35)
2012	Thailand	1.92 (1.60 to 2.31)
2013	Thailand	1.86 (1.55 to 2.22)
2014	Thailand	1.86 (1.53 to 2.22)
2015	Thailand	1.83 (1.54 to 2.15)
2016	Thailand	1.85 (1.56 to 2.15)
2017	Thailand	1.86 (1.55 to 2.18)
2018	Thailand	1.93 (1.60 to 2.29)
2019	Thailand	1.98 (1.64 to 2.37)
2020	Thailand	1.99 (1.61 to 2.46)
2021	Thailand	2.01 (1.55 to 2.58)
1990	Timor-Leste	0.54 (0.38 to 0.82)
1991	Timor-Leste	0.55 (0.39 to 0.82)
1992	Timor-Leste	0.56 (0.40 to 0.83)
1993	Timor-Leste	0.56 (0.41 to 0.82)
1994	Timor-Leste	0.57 (0.42 to 0.84)
1995	Timor-Leste	0.57 (0.43 to 0.83)
1996	Timor-Leste	0.57 (0.43 to 0.82)
1997	Timor-Leste	0.57 (0.43 to 0.80)
1998	Timor-Leste	0.58 (0.44 to 0.80)
1999	Timor-Leste	0.58 (0.43 to 0.80)
2000	Timor-Leste	0.58 (0.43 to 0.81)
2001	Timor-Leste	0.58 (0.43 to 0.81)
2002	Timor-Leste	0.58 (0.44 to 0.82)
2003	Timor-Leste	0.59 (0.44 to 0.82)
2004	Timor-Leste	0.58 (0.44 to 0.82)
2005	Timor-Leste	0.59 (0.44 to 0.83)
2006	Timor-Leste	0.60 (0.45 to 0.85)
2007	Timor-Leste	0.60 (0.45 to 0.86)
2008	Timor-Leste	0.61 (0.46 to 0.88)
2009	Timor-Leste	0.63 (0.47 to 0.91)
2010	Timor-Leste	0.65 (0.47 to 0.94)
2011	Timor-Leste	0.67 (0.48 to 0.99)
2012	Timor-Leste	0.69 (0.49 to 1.04)
2013	Timor-Leste	0.71 (0.49 to 1.07)

2014	Timor-Leste	0.72 (0.50 to 1.08)
2015	Timor-Leste	0.72 (0.49 to 1.09)
2016	Timor-Leste	0.72 (0.49 to 1.09)
2017	Timor-Leste	0.73 (0.49 to 1.12)
2018	Timor-Leste	0.73 (0.49 to 1.13)
2019	Timor-Leste	0.74 (0.49 to 1.14)
2020	Timor-Leste	0.75 (0.49 to 1.15)
2021	Timor-Leste	0.74 (0.49 to 1.15)
1990	Viet Nam	0.67 (0.48 to 0.90)
1991	Viet Nam	0.68 (0.49 to 0.92)
1992	Viet Nam	0.69 (0.50 to 0.94)
1993	Viet Nam	0.70 (0.51 to 0.94)
1994	Viet Nam	0.72 (0.52 to 0.96)
1995	Viet Nam	0.73 (0.54 to 0.97)
1996	Viet Nam	0.75 (0.55 to 0.99)
1997	Viet Nam	0.76 (0.56 to 1.00)
1998	Viet Nam	0.77 (0.58 to 1.02)
1999	Viet Nam	0.79 (0.59 to 1.03)
2000	Viet Nam	0.80 (0.59 to 1.04)
2001	Viet Nam	0.81 (0.60 to 1.07)
2002	Viet Nam	0.83 (0.61 to 1.09)
2003	Viet Nam	0.85 (0.62 to 1.10)
2004	Viet Nam	0.87 (0.64 to 1.12)
2005	Viet Nam	0.89 (0.66 to 1.13)
2006	Viet Nam	0.90 (0.67 to 1.15)
2007	Viet Nam	0.92 (0.69 to 1.15)
2008	Viet Nam	0.93 (0.70 to 1.16)
2009	Viet Nam	0.94 (0.71 to 1.20)
2010	Viet Nam	0.96 (0.71 to 1.22)
2011	Viet Nam	0.97 (0.72 to 1.24)
2012	Viet Nam	0.99 (0.74 to 1.27)
2013	Viet Nam	1.00 (0.74 to 1.30)
2014	Viet Nam	1.01 (0.75 to 1.32)
2015	Viet Nam	1.03 (0.76 to 1.34)
2016	Viet Nam	1.03 (0.76 to 1.36)
2017	Viet Nam	1.04 (0.76 to 1.39)
2018	Viet Nam	1.05 (0.77 to 1.40)
2019	Viet Nam	1.06 (0.79 to 1.41)
2020	Viet Nam	1.07 (0.79 to 1.42)
2021	Viet Nam	1.07 (0.78 to 1.42)

ASDR, age-standardized death rates.

Fig 4E. ASDR in East Asia from 1990 to 2021

Year	Location	ASDR per 100,000
1990	China	0.33 (0.27 to 0.42)
1991	China	0.34 (0.27 to 0.42)
1992	China	0.34 (0.27 to 0.43)
1993	China	0.35 (0.28 to 0.43)
1994	China	0.35 (0.29 to 0.42)
1995	China	0.35 (0.30 to 0.42)
1996	China	0.35 (0.30 to 0.41)
1997	China	0.35 (0.31 to 0.41)
1998	China	0.35 (0.31 to 0.40)
1999	China	0.35 (0.31 to 0.40)
2000	China	0.35 (0.31 to 0.40)
2001	China	0.35 (0.32 to 0.39)
2002	China	0.36 (0.32 to 0.40)
2003	China	0.36 (0.33 to 0.40)
2004	China	0.36 (0.33 to 0.40)
2005	China	0.37 (0.33 to 0.40)
2006	China	0.38 (0.35 to 0.41)
2007	China	0.39 (0.36 to 0.43)
2008	China	0.41 (0.37 to 0.45)
2009	China	0.43 (0.39 to 0.48)
2010	China	0.45 (0.40 to 0.50)
2011	China	0.45 (0.40 to 0.50)
2012	China	0.44 (0.39 to 0.50)
2013	China	0.44 (0.38 to 0.50)
2014	China	0.44 (0.38 to 0.51)
2015	China	0.44 (0.38 to 0.52)
2016	China	0.45 (0.38 to 0.53)
2017	China	0.45 (0.37 to 0.55)
2018	China	0.46 (0.37 to 0.55)
2019	China	0.46 (0.36 to 0.56)
2020	China	0.46 (0.37 to 0.58)
2021	China	0.46 (0.36 to 0.59)
1990	Democratic People's Republic of Korea	0.55 (0.41 to 0.73)
1991	Democratic People's Republic of Korea	0.55 (0.41 to 0.72)
1992	Democratic People's Republic of Korea	0.55 (0.41 to 0.72)
1993	Democratic People's Republic of Korea	0.55 (0.41 to 0.71)
1994	Democratic People's Republic of Korea	0.54 (0.41 to 0.70)
1995	Democratic People's Republic of Korea	0.54 (0.41 to 0.68)
1996	Democratic People's Republic of Korea	0.54 (0.40 to 0.68)
1997	Democratic People's Republic of Korea	0.53 (0.40 to 0.68)

1998	Democratic People's Republic of Korea	0.53 (0.40 to 0.68)
1999	Democratic People's Republic of Korea	0.52 (0.40 to 0.67)
2000	Democratic People's Republic of Korea	0.52 (0.39 to 0.67)
2001	Democratic People's Republic of Korea	0.52 (0.39 to 0.66)
2002	Democratic People's Republic of Korea	0.52 (0.40 to 0.66)
2003	Democratic People's Republic of Korea	0.52 (0.39 to 0.67)
2004	Democratic People's Republic of Korea	0.53 (0.40 to 0.67)
2005	Democratic People's Republic of Korea	0.53 (0.40 to 0.68)
2006	Democratic People's Republic of Korea	0.54 (0.41 to 0.70)
2007	Democratic People's Republic of Korea	0.55 (0.42 to 0.71)
2008	Democratic People's Republic of Korea	0.55 (0.42 to 0.72)
2009	Democratic People's Republic of Korea	0.56 (0.43 to 0.73)
2010	Democratic People's Republic of Korea	0.57 (0.43 to 0.74)
2011	Democratic People's Republic of Korea	0.56 (0.43 to 0.74)
2012	Democratic People's Republic of Korea	0.56 (0.42 to 0.74)
2013	Democratic People's Republic of Korea	0.56 (0.42 to 0.73)
2014	Democratic People's Republic of Korea	0.56 (0.42 to 0.73)
2015	Democratic People's Republic of Korea	0.56 (0.42 to 0.73)
2016	Democratic People's Republic of Korea	0.56 (0.43 to 0.74)
2017	Democratic People's Republic of Korea	0.55 (0.42 to 0.74)
2018	Democratic People's Republic of Korea	0.55 (0.43 to 0.73)
2019	Democratic People's Republic of Korea	0.55 (0.42 to 0.73)
2020	Democratic People's Republic of Korea	0.55 (0.42 to 0.73)
2021	Democratic People's Republic of Korea	0.55 (0.41 to 0.72)

ASDR, age-standardized death rates.

Fig 4F. ASDR in men and women from Asia in 2021

Sex	Location	ASDR per 100,000
Male	Central Asia	3.08 (2.75 to 3.43)
Female	Central Asia	1.22 (1.07 to 1.39)
Male	East Asia	0.78 (0.58 to 1.00)
Female	East Asia	0.27 (0.20 to 0.34)
Male	Japan	6.61 (6.07 to 6.89)
Female	Japan	3.72 (2.89 to 4.25)
Male	Republic of Korea	2.48 (1.94 to 3.02)
Female	Republic of Korea	1.50 (1.04 to 1.90)
Male	Singapore	2.86 (2.56 to 3.13)
Female	Singapore	1.55 (1.30 to 1.75)
Male	South Asia	1.57 (1.01 to 2.63)
Female	South Asia	0.90 (0.59 to 1.26)
Male	Southeast Asia	2.00 (1.69 to 2.41)
Female	Southeast Asia	0.92 (0.77 to 1.11)

ASDR, age-standardized death rates.

Fig 4G. ASDR in men and women from South Asia in 2021

Sex	Location	ASDR per 100,000
Female	Nepal	1.04 (0.62 to 1.66)
Male	Nepal	1.35 (0.75 to 2.59)
Female	Pakistan	1.24 (0.77 to 1.79)
Male	Pakistan	1.69 (1.06 to 2.77)
Female	Bhutan	1.19 (0.77 to 1.63)
Male	Bhutan	1.78 (0.81 to 3.14)
Female	Bangladesh	0.92 (0.53 to 1.46)
Male	Bangladesh	1.48 (0.82 to 2.94)
Female	India	0.88 (0.55 to 1.23)
Male	India	1.57 (0.95 to 2.61)

ASDR, age-standardized death rates.

Fig 4H. ASDR in men and women from Central Asia in 2021

Sex	Location	ASDR per 100,000
Male	Armenia	13.82 (11.42 to 16.43)
Female	Armenia	5.85 (4.76 to 7.20)
Male	Azerbaijan	2.10 (1.27 to 3.34)
Female	Azerbaijan	0.83 (0.53 to 1.31)
Male	Georgia	5.57 (4.73 to 6.50)
Female	Georgia	1.11 (0.88 to 1.49)
Male	Kazakhstan	3.75 (3.02 to 4.56)
Female	Kazakhstan	1.31 (1.02 to 1.63)
Male	Kyrgyzstan	1.26 (1.00 to 1.54)
Female	Kyrgyzstan	0.47 (0.38 to 0.57)

Male	Mongolia	0.86 (0.55 to 1.28)
Female	Mongolia	0.57 (0.41 to 0.77)
Male	Tajikistan	0.43 (0.29 to 0.61)
Female	Tajikistan	0.08 (0.06 to 0.11)
Male	Turkmenistan	2.87 (2.13 to 4.30)
Female	Turkmenistan	1.14 (0.81 to 1.51)
Male	Uzbekistan	1.38 (1.12 to 1.68)
Female	Uzbekistan	0.78 (0.61 to 0.98)

Fig 4I. ASDR in men and women from Southeast Asia in 2021

Sex	Location	ASDR per 100,000
Male	Cambodia	1.15 (0.67 to 2.07)
Female	Cambodia	0.51 (0.31 to 0.80)
Male	Indonesia	1.33 (0.74 to 1.96)
Female	Indonesia	0.78 (0.49 to 1.17)
Male	Lao People's Democratic Republic	1.25 (0.82 to 1.84)
Female	Lao People's Democratic Republic	0.66 (0.47 to 0.94)
Male	Malaysia	5.94 (4.63 to 7.45)
Female	Malaysia	2.22 (1.74 to 2.73)
Male	Maldives	0.81 (0.32 to 1.42)
Female	Maldives	0.37 (0.24 to 0.54)
Male	Mauritius	1.49 (1.36 to 1.59)
Female	Mauritius	0.44 (0.39 to 0.49)
Male	Myanmar	1.49 (1.02 to 2.10)
Female	Myanmar	0.61 (0.44 to 0.84)
Male	Philippines	1.88 (1.45 to 2.39)
Female	Philippines	0.97 (0.76 to 1.21)
Male	Seychelles	1.65 (0.91 to 2.51)
Female	Seychelles	0.63 (0.42 to 0.88)
Male	Sri Lanka	0.63 (0.41 to 0.90)
Female	Sri Lanka	0.19 (0.12 to 0.27)
Male	Thailand	2.82 (2.16 to 3.73)
Female	Thailand	1.36 (0.98 to 1.82)
Male	Timor-Leste	0.92 (0.55 to 1.64)
Female	Timor-Leste	0.58 (0.38 to 0.91)
Male	Viet Nam	1.80 (1.18 to 2.62)
Female	Viet Nam	0.63 (0.46 to 0.85)

ASDR, age-standardized death rates.

Fig 4J. ASDR in men and women from GBD East Asia in 2021

Sex	Location	ASDR per 100,000
Male	China	0.71 (0.51 to 0.94)
Female	China	0.24 (0.18 to 0.32)
Male	Democratic People's Republic of Korea	0.74 (0.53 to 1.03)
Female	Democratic People's Republic of Korea	0.39 (0.27 to 0.53)

ASDR, age-standardized death rates.

Data for Figure 5

Fig 5A. Global death number, death rate and ASDR in men and women and the difference between men and women in these metrics from 1990 to 2021

Year	Sex	ASDR per 100,000
1990	Female	1.58 (1.41 to 1.76)
1991	Female	1.60 (1.43 to 1.77)
1992	Female	1.60 (1.43 to 1.77)
1993	Female	1.62 (1.45 to 1.79)
1994	Female	1.64 (1.47 to 1.81)
1995	Female	1.64 (1.47 to 1.81)
1996	Female	1.64 (1.45 to 1.80)
1997	Female	1.61 (1.43 to 1.78)
1998	Female	1.61 (1.43 to 1.77)
1999	Female	1.65 (1.46 to 1.81)
2000	Female	1.63 (1.44 to 1.78)
2001	Female	1.57 (1.38 to 1.72)
2002	Female	1.54 (1.36 to 1.69)
2003	Female	1.53 (1.34 to 1.67)
2004	Female	1.51 (1.32 to 1.65)
2005	Female	1.49 (1.30 to 1.63)
2006	Female	1.46 (1.27 to 1.60)
2007	Female	1.44 (1.25 to 1.58)
2008	Female	1.41 (1.23 to 1.55)
2009	Female	1.39 (1.21 to 1.53)
2010	Female	1.37 (1.19 to 1.51)
2011	Female	1.36 (1.17 to 1.49)
2012	Female	1.34 (1.16 to 1.47)
2013	Female	1.33 (1.15 to 1.46)
2014	Female	1.33 (1.15 to 1.46)
2015	Female	1.33 (1.15 to 1.46)
2016	Female	1.34 (1.15 to 1.46)
2017	Female	1.33 (1.14 to 1.45)
2018	Female	1.32 (1.13 to 1.44)
2019	Female	1.31 (1.13 to 1.43)
2020	Female	1.29 (1.09 to 1.41)
2021	Female	1.28 (1.10 to 1.42)
1990	Male	3.87 (3.61 to 4.18)
1991	Male	3.89 (3.63 to 4.20)
1992	Male	3.90 (3.64 to 4.21)
1993	Male	3.95 (3.69 to 4.25)
1994	Male	3.94 (3.68 to 4.24)
1995	Male	3.94 (3.68 to 4.24)

1996	Male	3.89 (3.64 to 4.19)
1997	Male	3.81 (3.57 to 4.11)
1998	Male	3.76 (3.52 to 4.06)
1999	Male	3.74 (3.50 to 4.02)
2000	Male	3.67 (3.42 to 3.95)
2001	Male	3.56 (3.32 to 3.83)
2002	Male	3.52 (3.28 to 3.79)
2003	Male	3.46 (3.23 to 3.73)
2004	Male	3.37 (3.14 to 3.64)
2005	Male	3.32 (3.09 to 3.58)
2006	Male	3.23 (3.00 to 3.48)
2007	Male	3.16 (2.94 to 3.41)
2008	Male	3.09 (2.87 to 3.34)
2009	Male	3.00 (2.79 to 3.25)
2010	Male	2.95 (2.73 to 3.19)
2011	Male	2.87 (2.66 to 3.10)
2012	Male	2.80 (2.59 to 3.03)
2013	Male	2.75 (2.53 to 3.00)
2014	Male	2.72 (2.52 to 2.97)
2015	Male	2.71 (2.49 to 2.93)
2016	Male	2.70 (2.48 to 2.92)
2017	Male	2.66 (2.46 to 2.88)
2018	Male	2.64 (2.44 to 2.85)
2019	Male	2.62 (2.41 to 2.83)
2020	Male	2.58 (2.37 to 2.80)
2021	Male	2.57 (2.36 to 2.79)

Year	Sex	Death number
1990	Female	30796 (27621.84 to 34388.37)
1991	Female	32018 (28760.18 to 35713.27)
1992	Female	32929 (29546.00 to 36521.50)
1993	Female	34150 (30857.21 to 37849.78)
1994	Female	35522 (32095.53 to 39259.72)
1995	Female	36453 (32909.40 to 40202.92)
1996	Female	37152 (33520.06 to 40867.28)
1997	Female	37568 (33679.04 to 41416.85)
1998	Female	38415 (34352.16 to 42231.70)
1999	Female	40348 (36116.73 to 44289.87)
2000	Female	40909 (36529.67 to 44759.55)
2001	Female	40427 (35967.20 to 44404.53)
2002	Female	40829 (36275.75 to 44859.55)
2003	Female	41502 (36737.80 to 45521.07)
2004	Female	42119 (37170.78 to 46118.02)

2005	Female	42724 (37544.64 to 46844.95)
2006	Female	43146 (37831.51 to 47438.28)
2007	Female	44000 (38461.52 to 48273.65)
2008	Female	44439 (38841.74 to 48837.93)
2009	Female	45162 (39268.30 to 49554.07)
2010	Female	46036 (39920.77 to 50431.42)
2011	Female	47002 (40690.93 to 51422.72)
2012	Female	47972 (41370.03 to 52553.08)
2013	Female	49111 (42467.50 to 53803.22)
2014	Female	50588 (43612.81 to 55435.66)
2015	Female	52147 (45040.36 to 57020.52)
2016	Female	54054 (46640.12 to 59129.70)
2017	Female	55256 (47542.19 to 60563.92)
2018	Female	56562 (48677.71 to 61898.47)
2019	Female	58066 (50156.32 to 63606.68)
2020	Female	58673 (49841.95 to 64515.16)
2021	Female	60063 (51302.68 to 66297.91)
1990	Male	57557 (53978.50 to 62640.82)
1991	Male	59505 (55885.62 to 64553.35)
1992	Male	61319 (57685.00 to 66512.24)
1993	Male	63908 (60012.88 to 69154.28)
1994	Male	65529 (61579.58 to 70895.33)
1995	Male	67188 (63127.56 to 72577.24)
1996	Male	67904 (63840.78 to 73405.35)
1997	Male	68196 (64070.49 to 73915.81)
1998	Male	68923 (64730.30 to 74707.99)
1999	Male	70523 (66185.68 to 76033.74)
2000	Male	70982 (66693.60 to 76685.79)
2001	Male	70769 (66367.24 to 76496.40)
2002	Male	71802 (67395.20 to 77614.21)
2003	Male	72666 (68244.54 to 78483.35)
2004	Male	72799 (68276.63 to 78639.03)
2005	Male	73763 (69258.79 to 79660.02)
2006	Male	73871 (69414.50 to 79958.75)
2007	Male	74751 (70054.06 to 80778.75)
2008	Male	75479 (70689.82 to 81904.33)
2009	Male	75794 (70863.68 to 82386.26)
2010	Male	76930 (71966.03 to 83437.86)
2011	Male	77204 (72213.93 to 83588.15)
2012	Male	77926 (72672.01 to 84414.01)
2013	Male	78949 (73221.27 to 86059.97)
2014	Male	80646 (75349.05 to 88032.37)
2015	Male	82576 (76866.22 to 89531.10)
2016	Male	85054 (79039.08 to 92352.61)

2017	Male	86727 (80708.98 to 94031.04)
2018	Male	88951 (82813.76 to 96597.18)
2019	Male	90946 (84449.84 to 98829.59)
2020	Male	92175 (85150.78 to 100312.14)
2021	Male	93864 (86610.38 to 102152.79)

Year	Sex	Death rate per 100,000
1990	Female	1.16 (1.04 to 1.30)
1991	Female	1.19 (1.07 to 1.33)
1992	Female	1.21 (1.08 to 1.34)
1993	Female	1.23 (1.12 to 1.37)
1994	Female	1.27 (1.15 to 1.40)
1995	Female	1.28 (1.16 to 1.42)
1996	Female	1.29 (1.17 to 1.42)
1997	Female	1.29 (1.16 to 1.42)
1998	Female	1.30 (1.16 to 1.43)
1999	Female	1.35 (1.21 to 1.48)
2000	Female	1.35 (1.21 to 1.48)
2001	Female	1.32 (1.17 to 1.45)
2002	Female	1.31 (1.17 to 1.44)
2003	Female	1.32 (1.17 to 1.45)
2004	Female	1.32 (1.17 to 1.45)
2005	Female	1.32 (1.16 to 1.45)
2006	Female	1.32 (1.16 to 1.45)
2007	Female	1.33 (1.16 to 1.45)
2008	Female	1.32 (1.15 to 1.45)
2009	Female	1.32 (1.15 to 1.45)
2010	Female	1.33 (1.15 to 1.46)
2011	Female	1.34 (1.16 to 1.47)
2012	Female	1.35 (1.17 to 1.48)
2013	Female	1.37 (1.18 to 1.50)
2014	Female	1.39 (1.20 to 1.52)
2015	Female	1.42 (1.22 to 1.55)
2016	Female	1.45 (1.25 to 1.59)
2017	Female	1.46 (1.26 to 1.61)
2018	Female	1.48 (1.28 to 1.62)
2019	Female	1.51 (1.30 to 1.65)
2020	Female	1.51 (1.28 to 1.66)
2021	Female	1.53 (1.30 to 1.69)
1990	Male	2.14 (2.01 to 2.33)
1991	Male	2.18 (2.05 to 2.37)
1992	Male	2.21 (2.08 to 2.40)
1993	Male	2.27 (2.14 to 2.46)
1994	Male	2.30 (2.16 to 2.49)

1995	Male	2.33 (2.19 to 2.52)
1996	Male	2.32 (2.18 to 2.51)
1997	Male	2.30 (2.16 to 2.50)
1998	Male	2.30 (2.16 to 2.49)
1999	Male	2.32 (2.18 to 2.51)
2000	Male	2.31 (2.17 to 2.50)
2001	Male	2.28 (2.13 to 2.46)
2002	Male	2.28 (2.14 to 2.46)
2003	Male	2.28 (2.14 to 2.46)
2004	Male	2.25 (2.11 to 2.43)
2005	Male	2.25 (2.12 to 2.44)
2006	Male	2.23 (2.09 to 2.41)
2007	Male	2.23 (2.09 to 2.41)
2008	Male	2.22 (2.08 to 2.41)
2009	Male	2.20 (2.05 to 2.39)
2010	Male	2.20 (2.06 to 2.39)
2011	Male	2.18 (2.04 to 2.36)
2012	Male	2.18 (2.03 to 2.36)
2013	Male	2.18 (2.02 to 2.38)
2014	Male	2.20 (2.05 to 2.40)
2015	Male	2.22 (2.07 to 2.41)
2016	Male	2.26 (2.10 to 2.46)
2017	Male	2.28 (2.12 to 2.47)
2018	Male	2.31 (2.15 to 2.51)
2019	Male	2.34 (2.17 to 2.54)
2020	Male	2.35 (2.17 to 2.56)
2021	Male	2.37 (2.19 to 2.58)

Year	Mean difference in death number
1990	26762 (21978 to 32987)
1991	27487 (22618 to 33744)
1992	28390 (23425 to 34705)
1993	29758 (24658 to 36178)
1994	30007 (24779 to 36547)
1995	30735 (25346 to 37301)
1996	30752 (25302 to 37390)
1997	30627 (24958 to 37521)
1998	30507 (24670 to 37438)
1999	30175 (24115 to 36950)
2000	30073 (23944 to 36955)
2001	30342 (24075 to 37315)
2002	30973 (24636 to 38046)
2003	31164 (24665 to 38235)
2004	30680 (23977 to 37758)

2005	31039 (24175 to 38233)
2006	30725 (23789 to 38174)
2007	30751 (23490 to 38141)
2008	31040 (23673 to 38827)
2009	30632 (22948 to 38554)
2010	30894 (23018 to 38747)
2011	30202 (22157 to 37968)
2012	29954 (21516 to 37896)
2013	29838 (21067 to 38358)
2014	30057 (21299 to 38892)
2015	30430 (21314 to 38922)
2016	30999 (21453 to 39890)
2017	31472 (21688 to 40501)
2018	32390 (22399 to 41714)
2019	32881 (22645 to 42516)
2020	33502 (22218 to 43519)
2021	33801 (22427 to 44173)

Year	Mean difference in death rate per 100,000
1990	0.98 (0.80 to 1.21)
1991	0.99 (0.81 to 1.22)
1992	1.01 (0.83 to 1.24)
1993	1.04 (0.86 to 1.27)
1994	1.03 (0.85 to 1.26)
1995	1.04 (0.86 to 1.27)
1996	1.03 (0.84 to 1.26)
1997	1.01 (0.82 to 1.25)
1998	1.00 (0.80 to 1.23)
1999	0.97 (0.77 to 1.20)
2000	0.96 (0.76 to 1.18)
2001	0.96 (0.75 to 1.18)
2002	0.97 (0.76 to 1.19)
2003	0.96 (0.75 to 1.18)
2004	0.93 (0.72 to 1.15)
2005	0.93 (0.72 to 1.15)
2006	0,91 (0.70 to 1.14)
2007	0.90 (0.68 to 1.12)
2008	0.90 (0.68 to 1.13)
2009	0.87 (0.65 to 1.10)
2010	0.87 (0.64 to 1.10)
2011	0.84 (0.61 to 1.06)
2012	0.83 (0.59 to 1.05)
2013	0.81 (0.57 to 1.05)
2014	0.81 (0.57 to 1.05)

2015	0.81 (0.56 to 1.04)
2016	0.81 (0.56 to 1.05)
2017	0.82 (0.56 to 1.05)
2018	0.83 (0.57 to 1.07)
2019	0.83 (0.57 to 1.08)
2020	0.84 (0.55 to 1.10)
2021	0.84 (0.55 to 1.11)

Year	Mean difference in ASDR
1990	2.29 (0.31 to 0.36)
1991	2.30 (0.31 to 0.35)
1992	2.30 (0.31 to 0.35)
1993	2.33 (0.31 to 0.35)
1994	2.29 (0.30 to 0.34)
1995	2.29 (0.31 to 0.34)
1996	2.25 (0.30 to 0.34)
1997	2.20 (0.31 to 0.34)
1998	2.15 (0.30 to 0.34)
1999	2.10 (0.31 to 0.32)
2000	2.04 (0.31 to 0.32)
2001	2.00 (0.30 to 0.31)
2002	1.97 (0.30 to 0.31)
2003	1.94 (0.29 to 0.31)
2004	1.87 (0.30 to 0.30)
2005	1.83 (0.29 to 0.30)
2006	1.77 (0.30 to 0.30)
2007	1.72 (0.29 to 0.28)
2008	1.67 (0.28 to 0.29)
2009	1.61 (0.28 to 0.28)
2010	1.58 (0.29 to 0.27)
2011	1.51 (0.28 to 0.26)
2012	1.46 (0.29 to 0.26)
2013	1.42 (0.29 to 0.28)
2014	1.39 (0.28 to 0.28)
2015	1.37 (0.28 to 0.26)
2016	1.36 (0.28 to 0.25)
2017	1.34 (0.27 to 0.25)
2018	1.33 (0.28 to 0.24)
2019	1.31 (0.27 to 0.25)
2020	1.29 (0.28 to 0.25)
2021	1.29 (0.28 to 0.26)

Fig 5B. Global percentage changes in death number, death rate and ASDR in men and women from 1990 to 2021

Metric	Sex	% change<br>1990, 2021
Number	Female	95.04 (75.25 to 110.89)
Rate	Female	31.34 (18.02 to 42.02)
ASDR	Female	-18.79 (-26.03 to -12.76)
Number	Male	63.08 (53.44 to 73.74)
Rate	Male	10.62 (4.08 to 17.85)
ASDR	Male	-33.58 (-37.06 to -29.76)

ASDR, age-standardized death rates.

Fig 5C. Global death number and death rate per 100,000 in men and women and the difference between men and women in these metrics per age groups in 2021

Age	Sex	Death number
15-19 years	Female	73 (60 to 88)
20-24 years	Female	133 (110 to 163)
25-29 years	Female	184 (153 to 218)
30-34 years	Female	256 (227 to 292)
35-39 years	Female	404 (364 to 444)
40-44 years	Female	577 (515 to 638)
45-49 years	Female	925 (828 to 1026)
50-54 years	Female	1384 (1250 to 1553)
55-59 years	Female	2288 (2040 to 2587)
60-64 years	Female	3323 (3007 to 3779)
65-69 years	Female	5058 (4610 to 5562)
70-74 years	Female	7212 (6484 to 8060)
75-79 years	Female	8211 (7266 to 8967)
80-84 years	Female	9830 (8218 to 10872)
85-89 years	Female	9705 (7419 to 11081)
90-94 years	Female	6984 (5046 to 8073)
95+ years	Female	3515 (2367 to 4171)
15-19 years	Male	131 (108 to 158)
20-24 years	Male	240 (203 to 286)
25-29 years	Male	405 (356 to 470)
30-34 years	Male	759 (665 to 882)
35-39 years	Male	1260 (1101 to 1470)
40-44 years	Male	1923 (1713 to 2189)
45-49 years	Male	2802 (2553 to 3128)
50-54 years	Male	4021 (3679 to 4456)
55-59 years	Male	6002 (5485 to 6617)
60-64 years	Male	8346 (7788 to 9181)
65-69 years	Male	11426 (10604 to 12616)
70-74 years	Male	13805 (12950 to 15214)
75-79 years	Male	13297 (12314 to 14558)
80-84 years	Male	12727 (11357 to 13895)
85-89 years	Male	9997 (8696 to 10773)
90-94 years	Male	5204 (4387 to 5674)
95+ years	Male	1520 (1157 to 1697)

Age	Sex	Death rate per 100,000
15-19 years	Female	0.02 (0.02 to 0.03)
20-24 years	Female	0.05 (0.04 to 0.06)
25-29 years	Female	0.06 (0.05 to 0.07)
30-34 years	Female	0.09 (0.08 to 0.10)
35-39 years	Female	0.15 (0.13 to 0.16)

40-44 years	Female	0.23 (0.21 to 0.26)
45-49 years	Female	0.39 (0.35 to 0.44)
50-54 years	Female	0.62 (0.56 to 0.70)
55-59 years	Female	1.14 (1.01 to 1.29)
60-64 years	Female	2.02 (1.83 to 2.30)
65-69 years	Female	3.51 (3.20 to 3.86)
70-74 years	Female	6.59 (5.92 to 7.36)
75-79 years	Female	11.39 (10.08 to 12.44)
80-84 years	Female	19.30 (16.14 to 21.35)
85-89 years	Female	34.09 (26.06 to 38.92)
90-94 years	Female	57.91 (41.84 to 66.94)
95+ years	Female	89.24 (60.11 to 105.90)
15-19 years	Male	0.04 (0.03 to 0.05)
20-24 years	Male	0.08 (0.07 to 0.09)
25-29 years	Male	0.14 (0.12 to 0.16)
30-34 years	Male	0.25 (0.22 to 0.29)
35-39 years	Male	0.45 (0.39 to 0.52)
40-44 years	Male	0.76 (0.68 to 0.87)
45-49 years	Male	1.18 (1.07 to 1.32)
50-54 years	Male	1.81 (1.66 to 2.01)
55-59 years	Male	3.08 (2.82 to 3.40)
60-64 years	Male	5.37 (5.01 to 5.90)
65-69 years	Male	8.67 (8.04 to 9.57)
70-74 years	Male	14.32 (13.43 to 15.78)
75-79 years	Male	22.24 (20.60 to 24.35)
80-84 years	Male	34.72 (30.99 to 37.91)
85-89 years	Male	57.94 (50.41 to 62.44)
90-94 years	Male	89.29 (75.27 to 97.35)
95+ years	Male	100.50 (76.52 to 112.24)

Age	Mean difference in death number
15-19 years	58 (31 to 89)
20-24 years	107 (63 to 162)
25-29 years	222 (164 to 295)
30-34 years	503 (404 to 631)
35-39 years	856 (692 to 1069)
40-44 years	1346 (1128 to 1619)
45-49 years	1877 (1609 to 2218)
50-54 years	2638 (2270 to 3105)
55-59 years	3714 (3140 to 4397)
60-64 years	5022 (4381 to 5973)
65-69 years	6368 (5431 to 7660)

70-74 years	6593 (5469 to 8237)
75-79 years	5086 (3722 to 6556)
80-84 years	2896 (781 to 4462)
85-89 years	292 (-2338 to 1872)
90-94 years	-1780 (-3884 to -595)
95+ years	-1995 (-3198 to -1315)

Age	Mean difference in death rate per 100,000
15-19 years	0.02 (0.01 to 0.03)
20-24 years	0.03 (0.02 to 0.05)
25-29 years	0.07 (0.05 to 0.10)
30-34 years	0.16 (0.13 to 0.20)
35-39 years	0.30 (0.24 to 0.38)
40-44 years	0.53 (0.44 to 0.64)
45-49 years	0.79 (0.67 to 0.93)
50-54 years	1.19 (1.03 to 1.40)
55-59 years	1.94 (1.65 to 2.29)
60-64 years	3.35 (2.94 to 3.95)
65-69 years	5.15 (4.46 to 6.12)
70-74 years	7.73 (6.62 to 9.39)
75-79 years	10.85 (8.75 to 13.21)
80-84 years	15.42 (10.52 to 19.21)
85-89 years	23.85 (12.84 to 30.46)
90-94 years	31.38 (10.06 to 43.48)
95+ years	11.26 (-26.48 to 31.64)

Fig 5D. Percentage changes in Four World Regions in aortic aneurysm-caused ASDR of men and women in 2021 compared to 1990

Location	Men	Women
**Europe**	-34.5 (-40.3 to -28.2)	-17.9 (-30.9 to 0.7)
**America**	-54.6 (-58.8 to -50.0)	-36.2 (-47.0 to -23.1)
**Africa**	-12.0 (-52.6 to 140.7)	-33.3 (-65.7 to 19.0)
**Asia**	65.3 (-30.5 to 203.1)	58.9 (-22.9 to 188.5)

ASDR, age-standardized death rates.

Fig 5E. Percentage changes in USA and some European countries in aortic aneurysm-caused ASDR of men and women in 2021 compared to 1990

Location	Men	Women
**USA**	-68.0 (-69.2 to -66.8)	-49.5 (-51.9 to -47.4)
**UK**	-65.6 (-67.1 to -64.5)	-50.4 (-53.6 to -47.8)
**Sweden**	-59.7 (-65.4 to -53.7)	-24.2 (-34.3 to -14.0)
**Norway**	-50.6 (-53.5 to -47.6)	-10.8 (-18.4 to -4.7)
**Denmark**	-40.9 (-48.1 to -33.7)	-9.0 (-20.7 to 2.3)
**Poland**	-29.9 (-36.7 to -22.9)	-15.9 (-28.0 to 1.3)
**Montenegro**	32.0 (-17.1 to 114.9)	23.8 (-6.7 to 65.5)
**Russia**	44.5 (28.7 to 58.1)	58.5 (41.4 to 79.2)

ASDR, age-standardized death rates.

Fig 5F. Percentage changes in selected Asian countries in AA-caused ASDR of men and women in 2021 compared to 1990

Location	Men	Women
**Georgia**	478.1 (322.5 to 672.4)	181.5 (83.9 to 380.0)
**Armenia**	128.6 (51.1 to 234.0)	69.9 (5.7 to 169.6)
**India**	103.6 (-45.9 to 511.5)	51.9 (-53.1 to 343.0)
**China**	71.6 (-10.5 to 241.5)	-8.8 (-52.6 to 67.5)
**Japan**	44.7 (28.3 to 59.4)	106.1 (49.6 to 167.9)
**Malaysia**	24.8 (-26.4 to 113.0)	11.5 (-27.8 to 68.4)
**South Korea**	8.6 (-43.4 to 116.6)	-4.3 (-52.3 to 83.5)
**North Korea**	2.0 (-48.6 to 106.8)	-10.8 (-53.9 to 74.6)
**Singapore**	-31.0 (-42.0 to -19.2)	4.7 (-18.8 to 29.9)

ASDR, age-standardized death rates.

Fig 5G. Percentage changes in top 10 countries with highest ASDR in aortic aneurysm-caused ASDR of men and women in 2021 compared to 1990

Location	Men	Women
Armenia	128.6 (67.7 to 202.0)	69.9 (18.3 to 132.0)
Montenegro	32.0 (-17.1 to 114.9)	23.8 (-6.7 to 66.0)
Japan	44.7 (38.7 to 50.2)	106.1 (80.1 to 122.0)
Saint Lucia	-45.9 (-57.4 to -33.8)	-57.2 (-66.1 to -48.0)
Brunei Darussalam	-3.1 (-32.5 to 46.8)	-16.6 (-41.1 to 26.0)
Norway	-50.6 (-53.5 to -47.6)	-10.8 (-18.4 to -5.0)
Denmark	-40.9 (-48.1 to -33.7)	-9.0 (-20.7 to 2.0)
Grenada	-4.2 (-27.4 to 16.7)	5.3 (-28.5 to 35.0)
Russian Federation	44.5 (28.7 to 58.1)	58.5 (41.4 to 79.0)
Uruguay	-27.2 (-37.0 to -18.5)	-18.4 (-30.8 to -7.0)

Data for Figure 6

Fig 6A. Percentage contribution of risk factors to global AA-ASDR grouped by sex in 2021

Risk factor	Sex	Percent contribution to ASDR (%)
Diet high in sodium	Male	1.01 (0.10 to 2.99)
Diet high in sodium	Female	0.64 (0.03 to 2.26)
Diet high in sodium	Both	0.87 (0.08 to 2.73)
Diet low in fruits	Male	3.50 (2.41 to 4.65)
Diet low in fruits	Female	3.76 (2.57 to 4.95)
Diet low in fruits	Both	3.63 (2.48 to 4.78)
Diet low in vegetables	Male	2.80 (1.88 to 3.97)
Diet low in vegetables	Female	2.92 (1.94 to 4.16)
Diet low in vegetables	Both	2.85 (1.90 to 4.02)
High body-mass index	Male	6.99 (3.79 to 11.85)
High body-mass index	Female	7.89 (4.26 to 13.30)
High body-mass index	Both	7.39 (4.01 to 12.72)
High systolic blood pressure	Male	16.80 (12.64 to 21.29)
High systolic blood pressure	Female	17.94 (13.40 to 22.59)
High systolic blood pressure	Both	17.32 (13.04 to 21.92)
Lead exposure	Male	0.73 (-0.10 to 1.89)
Lead exposure	Female	0.55 (-0.08 to 1.43)
Lead exposure	Both	0.65 (-0.09 to 1.70)
Smoking	Male	39.31 (33.55 to 45.51)
Smoking	Female	14.16 (11.23 to 17.36)
Smoking	Both	29.98 (25.47 to 34.85)

AA, aortic aneurysm; ASDR, age-standardized death rates.

Fig 6B. Percentage contribution of risk factors to AA-ASDR in world regions
Four world regions

Risk factor	Location	Percent contribution to ASDR (%)
Diet high in sodium	Africa	0.45 (0.01 to 1.82)
Diet low in fruits	Africa	4.45 (3.07 to 5.78)
Diet low in vegetables	Africa	4.84 (3.31 to 6.65)
High body-mass index	Africa	6.78 (3.81 to 11.57)
High systolic blood pressure	Africa	19.23 (14.57 to 24.09)
Lead exposure	Africa	0.87 (-0.12 to 2.19)
Smoking	Africa	16.85 (13.72 to 20.35)
Diet high in sodium	America	0.79 (0.03 to 2.70)
Diet low in fruits	America	2.77 (1.90 to 3.60)
Diet low in vegetables	America	3.60 (2.43 to 4.96)
High body-mass index	America	9.91 (5.25 to 16.90)
High systolic blood pressure	America	16.17 (11.81 to 20.63)
Lead exposure	America	0.64 (-0.08 to 1.67)
Smoking	America	33.05 (27.52 to 39.04)
Diet high in sodium	Asia	1.12 (0.12 to 3.26)
Diet low in fruits	Asia	4.03 (2.72 to 5.37)
Diet low in vegetables	Asia	2.32 (1.49 to 3.31)
High body-mass index	Asia	4.40 (2.40 to 7.04)
High systolic blood pressure	Asia	16.54 (12.33 to 21.47)
Lead exposure	Asia	0.77 (-0.11 to 2.00)
Smoking	Asia	26.82 (22.73 to 31.41)
Diet high in sodium	Europe	0.70 (0.06 to 2.31)
Diet low in fruits	Europe	3.53 (2.46 to 4.67)
Diet low in vegetables	Europe	2.65 (1.72 to 3.81)
High body-mass index	Europe	9.95 (5.27 to 17.18)
High systolic blood pressure	Europe	18.69 (14.18 to 23.62)
Lead exposure	Europe	0.46 (-0.06 to 1.16)
Smoking	Europe	37.24 (31.83 to 42.89)

Health System Grouping

Risk factor	Location	Percent contribution to ASDR (%)
Lead exposure	Advanced Health System	0.44 (-0.06 to 1.15)
Smoking	Advanced Health System	33.93 (28.57 to 39.27)
Diet low in fruits	Advanced Health System	3.53 (2.42 to 4.65)
Diet low in vegetables	Advanced Health System	2.33 (1.50 to 3.38)
Diet high in sodium	Advanced Health System	0.80 (0.05 to 2.63)
High systolic blood pressure	Advanced Health System	17.35 (13.00 to 22.07)
High body-mass index	Advanced Health System	8.56 (4.57 to 14.71)
Lead exposure	Basic Health System	0.80 (-0.11 to 2.08)
Smoking	Basic Health System	33.68 (28.72 to 38.74)

Diet low in fruits	Basic Health System	2.98 (2.05 to 3.98)
Diet low in vegetables	Basic Health System	3.14 (2.12 to 4.36)
Diet high in sodium	Basic Health System	1.21 (0.17 to 3.27)
High systolic blood pressure	Basic Health System	17.65 (13.06 to 22.15)
High body-mass index	Basic Health System	7.52 (4.07 to 12.76)
Lead exposure	Limited Health System	1.26 (-0.17 to 3.20)
Smoking	Limited Health System	23.13 (18.32 to 29.19)
Diet low in fruits	Limited Health System	5.17 (3.57 to 6.82)
Diet low in vegetables	Limited Health System	4.41 (3.01 to 6.07)
Diet high in sodium	Limited Health System	0.69 (0.02 to 2.48)
High systolic blood pressure	Limited Health System	17.03 (12.80 to 21.57)
High body-mass index	Limited Health System	4.02 (2.26 to 6.55)
Smoking	Minimal Health System	12.47 (7.19 to 17.54)
Diet low in fruits	Minimal Health System	5.03 (3.50 to 6.63)
Diet low in vegetables	Minimal Health System	5.53 (3.80 to 7.58)
Diet high in sodium	Minimal Health System	0.43 (0.00 to 1.87)
High systolic blood pressure	Minimal Health System	17.93 (13.31 to 22.74)
High body-mass index	Minimal Health System	4.25 (2.37 to 7.09)

Income

Risk factor	Location	Percent contribution to ASDR (%)
Diet high in sodium	Commonwealth High Income	0.52 (0.01 to 2.20)
Diet low in fruits	Commonwealth High Income	3.44 (2.39 to 4.52)
Diet low in vegetables	Commonwealth High Income	2.99 (1.97 to 4.22)
High body-mass index	Commonwealth High Income	8.89 (4.67 to 15.19)
High systolic blood pressure	Commonwealth High Income	15.02 (11.15 to 18.97)
Lead exposure	Commonwealth High Income	0.50 (-0.06 to 1.28)
Smoking	Commonwealth High Income	30.56 (24.71 to 37.65)
Diet high in sodium	Commonwealth Low Income	0.75 (0.02 to 2.52)
Diet low in fruits	Commonwealth Low Income	4.59 (3.16 to 6.06)
Diet low in vegetables	Commonwealth Low Income	5.24 (3.58 to 7.17)
High body-mass index	Commonwealth Low Income	3.50 (1.90 to 5.64)
High systolic blood pressure	Commonwealth Low Income	17.43 (13.13 to 22.23)
Lead exposure	Commonwealth Low Income	1.25 (-0.17 to 3.23)
Smoking	Commonwealth Low Income	27.63 (21.79 to 34.58)
Diet high in sodium	Commonwealth Middle Income	0.66 (0.03 to 2.39)
Diet low in fruits	Commonwealth Middle Income	5.28 (3.67 to 6.96)
Diet low in vegetables	Commonwealth Middle Income	4.29 (2.91 to 5.94)
High body-mass index	Commonwealth Middle Income	4.53 (2.54 to 7.53)
High systolic blood pressure	Commonwealth Middle Income	17.57 (13.21 to 22.12)
Lead exposure	Commonwealth Middle Income	1.18 (-0.16 to 3.04)
Smoking	Commonwealth Middle Income	22.67 (17.78 to 28.48)

SDI

Risk factor	Location	Percent contribution to ASDR (%)
Diet high in sodium	High SDI	0.86 (0.06 to 2.81)
Diet low in fruits	High SDI	3.56 (2.46 to 4.69)
Diet low in vegetables	High SDI	2.32 (1.50 to 3.37)
High body-mass index	High SDI	7.60 (4.09 to 12.96)
High systolic blood pressure	High SDI	16.52 (12.20 to 21.03)
Lead exposure	High SDI	0.44 (-0.06 to 1.15)
Smoking	High SDI	31.74 (26.59 to 37.29)
Diet high in sodium	High-middle SDI	0.92 (0.12 to 2.60)
Diet low in fruits	High-middle SDI	3.32 (2.31 to 4.39)
Diet low in vegetables	High-middle SDI	2.16 (1.39 to 3.14)
High body-mass index	High-middle SDI	9.71 (5.17 to 16.71)
High systolic blood pressure	High-middle SDI	18.75 (14.11 to 23.67)
Lead exposure	High-middle SDI	0.55 (-0.07 to 1.41)
Smoking	High-middle SDI	37.21 (32.14 to 41.99)
Diet high in sodium	Low SDI	0.62 (0.01 to 2.32)
Diet low in fruits	Low SDI	4.67 (3.24 to 6.17)
Diet low in vegetables	Low SDI	4.95 (3.38 to 6.77)
High body-mass index	Low SDI	3.74 (2.05 to 6.15)
High systolic blood pressure	Low SDI	16.60 (12.38 to 20.83)
Lead exposure	Low SDI	1.32 (-0.18 to 3.29)
Smoking	Low SDI	18.02 (13.92 to 23.13)
Diet high in sodium	Low-middle SDI	0.74 (0.04 to 2.53)
Diet low in fruits	Low-middle SDI	4.70 (3.23 to 6.27)
Diet low in vegetables	Low-middle SDI	4.41 (3.02 to 6.10)
High body-mass index	Low-middle SDI	5.01 (2.74 to 8.30)
High systolic blood pressure	Low-middle SDI	17.43 (13.19 to 22.10)
Lead exposure	Low-middle SDI	1.28 (-0.18 to 3.27)
Smoking	Low-middle SDI	26.69 (21.51 to 33.19)
Diet high in sodium	Middle SDI	1.04 (0.12 to 2.97)
Diet low in fruits	Middle SDI	3.38 (2.28 to 4.44)
Diet low in vegetables	Middle SDI	3.61 (2.45 to 4.98)
High body-mass index	Middle SDI	6.96 (3.76 to 11.86)
High systolic blood pressure	Middle SDI	17.51 (13.14 to 22.19)
Lead exposure	Middle SDI	0.81 (-0.11 to 2.09)
Smoking	Middle SDI	29.94 (25.48 to 34.57)

AA, aortic aneurysm; ASDR, age-standardized death rates; SDI, socio-demographic index.

Fig 6C. Percentage contribution of risk factors to AA-ASDR in world regions grouped by sex in 2021

Four World Regions

Risk factor	Sex	Location	Percent contribution to ASDR (%)
Diet high in sodium	Male	Africa	0.44 (0.01 to 1.85)
Diet high in sodium	Female	Africa	0.46 (0.01 to 1.78)
Diet low in fruits	Male	Africa	4.24 (2.93 to 5.50)
Diet low in fruits	Female	Africa	4.74 (3.31 to 6.24)
Diet low in vegetables	Male	Africa	4.62 (3.17 to 6.38)
Diet low in vegetables	Female	Africa	5.14 (3.49 to 7.06)
High body-mass index	Male	Africa	6.16 (3.39 to 10.68)
High body-mass index	Female	Africa	7.76 (4.24 to 12.95)
High systolic blood pressure	Male	Africa	18.26 (13.64 to 22.87)
High systolic blood pressure	Female	Africa	20.66 (15.65 to 25.75)
Lead exposure	Male	Africa	0.99 (-0.14 to 2.52)
Lead exposure	Female	Africa	0.68 (-0.10 to 1.81)
Smoking	Male	Africa	24.33 (19.73 to 29.23)
Smoking	Female	Africa	5.18 (3.93 to 6.62)
Diet high in sodium	Male	America	0.96 (0.05 to 3.10)
Diet high in sodium	Female	America	0.51 (0.00 to 2.07)
Diet low in fruits	Male	America	2.68 (1.86 to 3.51)
Diet low in fruits	Female	America	2.87 (1.91 to 3.80)
Diet low in vegetables	Male	America	3.60 (2.43 to 5.06)
Diet low in vegetables	Female	America	3.58 (2.40 to 4.98)
High body-mass index	Male	America	9.55 (5.07 to 16.39)
High body-mass index	Female	America	10.34 (5.44 to 17.92)
High systolic blood pressure	Male	America	15.59 (11.50 to 19.62)
High systolic blood pressure	Female	America	16.94 (12.39 to 21.81)
Lead exposure	Male	America	0.71 (-0.09 to 1.82)
Lead exposure	Female	America	0.55 (-0.07 to 1.44)
Smoking	Male	America	37.80 (31.46 to 44.56)
Smoking	Female	America	24.91 (20.22 to 30.08)
Diet high in sodium	Male	Asia	1.29 (0.15 to 3.63)
Diet high in sodium	Female	Asia	0.88 (0.06 to 2.76)
Diet low in fruits	Male	Asia	3.97 (2.71 to 5.29)
Diet low in fruits	Female	Asia	4.08 (2.76 to 5.46)
Diet low in vegetables	Male	Asia	2.26 (1.49 to 3.34)
Diet low in vegetables	Female	Asia	2.39 (1.48 to 3.47)

High body-mass index	Male	Asia	4.10 (2.27 to 6.66)
High body-mass index	Female	Asia	4.73 (2.57 to 7.72)
High systolic blood pressure	Male	Asia	16.20 (12.18 to 20.76)
High systolic blood pressure	Female	Asia	16.96 (12.35 to 22.07)
Lead exposure	Male	Asia	0.87 (-0.11 to 2.21)
Lead exposure	Female	Asia	0.63 (-0.09 to 1.67)
Smoking	Male	Asia	39.54 (33.31 to 45.56)
Smoking	Female	Asia	8.83 (6.81 to 11.46)
Diet high in sodium	Male	Europe	0.83 (0.07 to 2.58)
Diet high in sodium	Female	Europe	0.43 (0.02 to 1.76)
Diet low in fruits	Male	Europe	3.38 (2.38 to 4.49)
Diet low in fruits	Female	Europe	3.72 (2.60 to 4.88)
Diet low in vegetables	Male	Europe	2.61 (1.71 to 3.77)
Diet low in vegetables	Female	Europe	2.71 (1.76 to 3.88)
High body-mass index	Male	Europe	9.29 (4.95 to 16.17)
High body-mass index	Female	Europe	10.98 (5.77 to 18.87)
High systolic blood pressure	Male	Europe	18.13 (13.72 to 23.03)
High systolic blood pressure	Female	Europe	19.34 (14.58 to 24.22)
Lead exposure	Male	Europe	0.51 (-0.07 to 1.30)
Lead exposure	Female	Europe	0.37 (-0.05 to 0.94)
Smoking	Male	Europe	45.15 (38.60 to 51.90)
Smoking	Female	Europe	18.72 (15.34 to 22.36)

Health System Grouping

Risk factor	Sex	Location	Percent contribution to ASDR (%)
Diet high in sodium	Male	Advanced Health System	0.93 (0.07 to 2.90)
Diet high in sodium	Female	Advanced Health System	0.58 (0.02 to 2.17)
Diet low in fruits	Male	Advanced Health System	3.42 (2.36 to 4.56)
Diet low in fruits	Female	Advanced Health System	3.62 (2.49 to 4.73)
Diet low in vegetables	Male	Advanced Health System	2.32 (1.49 to 3.39)
Diet low in vegetables	Female	Advanced Health System	2.32 (1.49 to 3.38)
High body-mass index	Male	Advanced Health System	8.26 (4.45 to 14.08)
High body-mass index	Female	Advanced Health System	8.83 (4.72 to 15.09)
High systolic blood pressure	Male	Advanced Health System	16.95 (12.71 to 21.75)
High systolic blood pressure	Female	Advanced Health System	17.77 (13.08 to 22.52)
Lead exposure	Male	Advanced Health System	0.49 (-0.06 to 1.28)
Lead exposure	Female	Advanced Health System	0.37 (-0.05 to 0.97)

Smoking	Male	Advanced Health System	42.58 (36.35 to 49.25)
Smoking	Female	Advanced Health System	17.83 (14.41 to 21.44)
Diet high in sodium	Male	Basic Health System	1.39 (0.22 to 3.63)
Diet high in sodium	Female	Basic Health System	0.84 (0.07 to 2.56)
Diet low in fruits	Male	Basic Health System	2.92 (2.01 to 3.88)
Diet low in fruits	Female	Basic Health System	3.05 (2.07 to 4.09)
Diet low in vegetables	Male	Basic Health System	3.02 (2.06 to 4.20)
Diet low in vegetables	Female	Basic Health System	3.35 (2.24 to 4.70)
High body-mass index	Male	Basic Health System	6.89 (3.71 to 11.59)
High body-mass index	Female	Basic Health System	8.53 (4.57 to 14.81)
High systolic blood pressure	Male	Basic Health System	17.18 (12.67 to 21.74)
High systolic blood pressure	Female	Basic Health System	18.34 (13.79 to 23.24)
Lead exposure	Male	Basic Health System	0.89 (-0.12 to 2.29)
Lead exposure	Female	Basic Health System	0.66 (-0.09 to 1.72)
Smoking	Male	Basic Health System	43.05 (37.13 to 48.85)
Smoking	Female	Basic Health System	15.26 (12.27 to 18.33)
Diet high in sodium	Male	Limited Health System	0.78 (0.03 to 2.65)
Diet high in sodium	Female	Limited Health System	0.56 (0.01 to 2.16)
Diet low in fruits	Male	Limited Health System	5.04 (3.48 to 6.69)
Diet low in fruits	Female	Limited Health System	5.33 (3.69 to 7.01)
Diet low in vegetables	Male	Limited Health System	4.28 (2.91 to 5.86)
Diet low in vegetables	Female	Limited Health System	4.59 (3.12 to 6.32)
High body-mass index	Male	Limited Health System	3.57 (2.00 to 5.89)
High body-mass index	Female	Limited Health System	4.73 (2.59 to 8.06)
High systolic blood pressure	Male	Limited Health System	16.31 (12.14 to 20.83)
High systolic blood pressure	Female	Limited Health System	18.13 (13.62 to 22.65)
Lead exposure	Male	Limited Health System	1.37 (-0.18 to 3.48)
Lead exposure	Female	Limited Health System	1.08 (-0.15 to 2.74)
Smoking	Male	Limited Health System	32.61 (26.92 to 39.26)
Smoking	Female	Limited Health System	7.85 (6.04 to 10.19)
Diet high in sodium	Male	Minimal Health System	0.45 (0.00 to 1.97)
Diet high in sodium	Female	Minimal Health System	0.41 (0.00 to 1.74)
Diet low in fruits	Male	Minimal Health System	4.87 (3.38 to 6.42)
Diet low in fruits	Female	Minimal Health System	5.18 (3.61 to 6.85)
Diet low in vegetables	Male	Minimal Health System	5.41 (3.72 to 7.36)
Diet low in vegetables	Female	Minimal Health System	5.65 (3.84 to 7.73)
High body-mass index	Male	Minimal Health System	3.80 (2.09 to 6.22)
High body-mass index	Female	Minimal Health System	4.75 (2.63 to 8.04)
High systolic blood pressure	Male	Minimal Health System	16.98 (12.40 to 21.13)
High systolic blood	Female	Minimal Health System	18.92 (14.09 to 24.00)

pressure			
Lead exposure	Male	Minimal Health System	1.37 (-0.20 to 3.37)
Lead exposure	Female	Minimal Health System	0.95 (-0.13 to 2.42)
Smoking	Male	Minimal Health System	20.58 (16.80 to 24.76)
Smoking	Female	Minimal Health System	2.75 (2.11 to 3.50)

Income

Risk factor	Sex	Location	Percent contribution to ASDR (%)
Diet high in sodium	Male	Commonwealth High Income	0.63 (0.01 to 2.45)
Diet high in sodium	Female	Commonwealth High Income	0.34 (0.00 to 1.73)
Diet low in fruits	Male	Commonwealth High Income	3.36 (2.33 to 4.50)
Diet low in fruits	Female	Commonwealth High Income	3.53 (2.46 to 4.69)
Diet low in vegetables	Male	Commonwealth High Income	2.99 (1.99 to 4.24)
Diet low in vegetables	Female	Commonwealth High Income	2.97 (1.94 to 4.24)
High body-mass index	Male	Commonwealth High Income	8.67 (4.51 to 15.06)
High body-mass index	Female	Commonwealth High Income	9.10 (4.75 to 15.51)
High systolic blood pressure	Male	Commonwealth High Income	14.42 (10.59 to 18.48)
High systolic blood pressure	Female	Commonwealth High Income	15.82 (11.45 to 20.20)
Lead exposure	Male	Commonwealth High Income	0.56 (-0.07 to 1.46)
Lead exposure	Female	Commonwealth High Income	0.41 (-0.05 to 1.06)
Smoking	Male	Commonwealth High Income	33.92 (27.47 to 41.57)
Smoking	Female	Commonwealth High Income	24.36 (19.03 to 30.47)
Diet high in sodium	Male	Commonwealth Low Income	0.74 (0.01 to 2.50)
Diet high in sodium	Female	Commonwealth Low Income	0.77 (0.02 to 2.60)
Diet low in fruits	Male	Commonwealth Low Income	4.42 (3.05 to 5.91)
Diet low in fruits	Female	Commonwealth Low Income	4.84 (3.30 to 6.54)
Diet low in vegetables	Male	Commonwealth Low Income	5.04 (3.42 to 6.84)
Diet low in vegetables	Female	Commonwealth Low Income	5.55 (3.73 to 7.68)
High body-mass index	Male	Commonwealth Low Income	2.85 (1.55 to 4.68)
High body-mass index	Female	Commonwealth Low Income	4.55 (2.43 to 7.70)
High systolic blood pressure	Male	Commonwealth Low Income	15.96 (11.67 to 20.38)
High systolic blood pressure	Female	Commonwealth Low Income	19.74 (14.91 to 25.24)
Lead exposure	Male	Commonwealth Low Income	1.48 (-0.20 to 3.86)
Lead exposure	Female	Commonwealth Low Income	0.89 (-0.13 to 2.32)
Smoking	Male	Commonwealth Low Income	39.50 (33.38 to 46.24)
Smoking	Female	Commonwealth Low Income	8.37 (6.28 to 10.40)
Diet high in sodium	Male	Commonwealth Middle Income	0.77 (0.04 to 2.63)
Diet high in sodium	Female	Commonwealth Middle Income	0.48 (0.00 to 1.96)
Diet low in fruits	Male	Commonwealth Middle Income	5.14 (3.58 to 6.82)
Diet low in fruits	Female	Commonwealth Middle Income	5.47 (3.79 to 7.21)
Diet low in vegetables	Male	Commonwealth Middle Income	4.18 (2.85 to 5.81)
Diet low in vegetables	Female	Commonwealth Middle Income	4.47 (3.02 to 6.16)
High body-mass index	Male	Commonwealth Middle Income	4.04 (2.22 to 6.75)

High body-mass index	Female	Commonwealth Middle Income	5.34 (2.90 to 9.18)
High systolic blood pressure	Male	Commonwealth Middle Income	17.01 (12.70 to 21.73)
High systolic blood pressure	Female	Commonwealth Middle Income	18.48 (13.74 to 23.30)
Lead exposure	Male	Commonwealth Middle Income	1.26 (-0.17 to 3.22)
Lead exposure	Female	Commonwealth Middle Income	1.06 (-0.15 to 2.72)
Smoking	Male	Commonwealth Middle Income	31.64 (25.94 to 38.37)
Smoking	Female	Commonwealth Middle Income	7.67 (5.84 to 10.02)

SDI

Risk factor	Sex	Location	Percent contribution to ADR (%)
Diet high in sodium	Male	High SDI	0.99 (0.07 to 3.11)
Diet high in sodium	Female	High SDI	0.65 (0.03 to 2.35)
Diet low in fruits	Male	High SDI	3.51 (2.44 to 4.63)
Diet low in fruits	Female	High SDI	3.58 (2.46 to 4.70)
Diet low in vegetables	Male	High SDI	2.33 (1.53 to 3.39)
Diet low in vegetables	Female	High SDI	2.30 (1.46 to 3.34)
High body-mass index	Male	High SDI	7.48 (4.04 to 12.70)
High body-mass index	Female	High SDI	7.60 (4.07 to 12.83)
High systolic blood pressure	Male	High SDI	16.10 (12.03 to 20.76)
High systolic blood pressure	Female	High SDI	17.01 (12.40 to 21.91)
Lead exposure	Male	High SDI	0.48 (-0.06 to 1.28)
Lead exposure	Female	High SDI	0.38 (-0.05 to 0.98)
Smoking	Male	High SDI	39.44 (33.05 to 46.18)
Smoking	Female	High SDI	18.93 (15.01 to 23.10)
Diet high in sodium	Male	High-middle SDI	1.05 (0.15 to 2.85)
Diet high in sodium	Female	High-middle SDI	0.59 (0.05 to 1.94)
Diet low in fruits	Male	High-middle SDI	3.12 (2.19 to 4.14)
Diet low in fruits	Female	High-middle SDI	3.57 (2.45 to 4.73)
Diet low in vegetables	Male	High-middle SDI	2.13 (1.37 to 3.08)
Diet low in vegetables	Female	High-middle SDI	2.24 (1.42 to 3.28)
High body-mass index	Male	High-middle SDI	8.81 (4.68 to 15.31)
High body-mass index	Female	High-middle SDI	11.26 (5.98 to 19.23)
High systolic blood pressure	Male	High-middle SDI	18.04 (13.55 to 22.66)
High systolic blood pressure	Female	High-middle SDI	19.75 (14.97 to 24.57)
Lead exposure	Male	High-middle SDI	0.62 (-0.08 to 1.60)
Lead exposure	Female	High-middle SDI	0.43 (-0.06 to 1.10)
Smoking	Male	High-middle SDI	47.19 (40.93 to 53.06)
Smoking	Female	High-middle SDI	13.35 (10.85 to 15.87)
Diet high in sodium	Male	Low SDI	0.65 (0.02 to 2.35)
Diet high in sodium	Female	Low SDI	0.60 (0.01 to 2.29)
Diet low in fruits	Male	Low SDI	4.55 (3.15 to 6.03)
Diet low in fruits	Female	Low SDI	4.82 (3.36 to 6.38)
Diet low in vegetables	Male	Low SDI	4.85 (3.30 to 6.69)

Diet low in vegetables	Female	Low SDI	5.07 (3.48 to 6.89)
High body-mass index	Male	Low SDI	3.36 (1.87 to 5.48)
High body-mass index	Female	Low SDI	4.23 (2.31 to 7.15)
High systolic blood pressure	Male	Low SDI	15.87 (11.79 to 20.03)
High systolic blood pressure	Female	Low SDI	17.49 (13.12 to 22.07)
Lead exposure	Male	Low SDI	1.45 (-0.20 to 3.65)
Lead exposure	Female	Low SDI	1.14 (-0.16 to 2.91)
Smoking	Male	Low SDI	25.29 (20.88 to 30.31)
Smoking	Female	Low SDI	8.03 (5.97 to 10.57)
Diet high in sodium	Male	Low-middle SDI	0.85 (0.05 to 2.76)
Diet high in sodium	Female	Low-middle SDI	0.56 (0.01 to 2.17)
Diet low in fruits	Male	Low-middle SDI	4.57 (3.14 to 6.08)
Diet low in fruits	Female	Low-middle SDI	4.86 (3.34 to 6.49)
Diet low in vegetables	Male	Low-middle SDI	4.27 (2.92 to 5.89)
Diet low in vegetables	Female	Low-middle SDI	4.61 (3.12 to 6.38)
High body-mass index	Male	Low-middle SDI	4.52 (2.50 to 7.63)
High body-mass index	Female	Low-middle SDI	5.77 (3.13 to 10.05)
High systolic blood pressure	Male	Low-middle SDI	16.63 (12.40 to 21.23)
High systolic blood pressure	Female	Low-middle SDI	18.60 (14.12 to 23.26)
Lead exposure	Male	Low-middle SDI	1.41 (-0.19 to 3.60)
Lead exposure	Female	Low-middle SDI	1.08 (-0.16 to 2.74)
Smoking	Male	Low-middle SDI	37.32 (31.30 to 44.46)
Smoking	Female	Low-middle SDI	10.10 (7.89 to 12.78)
Diet high in sodium	Male	Middle SDI	1.22 (0.16 to 3.33)
Diet high in sodium	Female	Middle SDI	0.73 (0.05 to 2.39)
Diet low in fruits	Male	Middle SDI	3.31 (2.26 to 4.38)
Diet low in fruits	Female	Middle SDI	3.46 (2.32 to 4.67)
Diet low in vegetables	Male	Middle SDI	3.51 (2.39 to 4.83)
Diet low in vegetables	Female	Middle SDI	3.77 (2.56 to 5.25)
High body-mass index	Male	Middle SDI	6.37 (3.44 to 10.68)
High body-mass index	Female	Middle SDI	7.87 (4.29 to 13.43)
High systolic blood pressure	Male	Middle SDI	17.10 (12.74 to 21.52)
High systolic blood pressure	Female	Middle SDI	18.08 (13.47 to 22.96)
Lead exposure	Male	Middle SDI	0.90 (-0.12 to 2.31)
Lead exposure	Female	Middle SDI	0.67 (-0.09 to 1.76)
Smoking	Male	Middle SDI	39.15 (33.23 to 44.76)
Smoking	Female	Middle SDI	13.31 (10.65 to 16.09)

AA, aortic aneurysm; ASDR, age-standardized death rates.

Fig 6D. Percentage contribution of risk factors to AA-ASDR in high-burden countries in 2021

Risk factor	Sex	Location	Percent contribution to ASDR (%)
Diet high in sodium	Male	Armenia	0.98 (0.05 to 3.12)
Diet high in sodium	Female	Armenia	0.58 (0 to 2.35)
Diet low in fruits	Male	Armenia	2.29 (1.46 to 3.27)
Diet low in fruits	Female	Armenia	2.50 (1.54 to 3.63)
Diet low in vegetables	Male	Armenia	0.09 (0.01 to 0.28)
Diet low in vegetables	Female	Armenia	0.11 (0 to 0.42)
High body-mass index	Male	Armenia	8.01 (4.35 to 13.88)
High body-mass index	Female	Armenia	12.19 (6.30 to 20.94)
High systolic blood pressure	Male	Armenia	19.62 (14.30 to 25.63)
High systolic blood pressure	Female	Armenia	20.10 (13.79 to 28.11)
Lead exposure	Male	Armenia	0.60 (-0.08 to 1.61)
Lead exposure	Female	Armenia	0.47 (-0.06 to 1.32)
Smoking	Male	Armenia	60.34 (53.32 to 66.51)
Smoking	Female	Armenia	4.27 (2.95 to 6.17)
Diet high in sodium	Male	Brunei Darussalam	1.59 (0.16 to 4.29)
Diet high in sodium	Female	Brunei Darussalam	1.21 (0.05 to 3.67)
Diet low in fruits	Male	Brunei Darussalam	4.35 (3.01 to 5.85)
Diet low in fruits	Female	Brunei Darussalam	4.44 (2.99 to 6.11)
Diet low in vegetables	Male	Brunei Darussalam	4.13 (2.76 to 5.80)
Diet low in vegetables	Female	Brunei Darussalam	4.10 (2.65 to 5.89)
High body-mass index	Male	Brunei Darussalam	4.46 (2.43 to 7.53)
High body-mass index	Female	Brunei Darussalam	4.86 (2.65 to 8.18)
High systolic blood pressure	Male	Brunei Darussalam	15.41 (10.76 to 20.77)
High systolic blood pressure	Female	Brunei Darussalam	15.91 (9.94 to 22.52)
Lead exposure	Male	Brunei Darussalam	0.72 (-0.09 to 1.94)
Lead exposure	Female	Brunei Darussalam	0.52 (-0.07 to 1.44)
Smoking	Male	Brunei Darussalam	36.38 (28.77 to 44.41)
Smoking	Female	Brunei Darussalam	13.31 (9.37 to 18.27)
Diet high in sodium	Male	Denmark	0.57 (0 to 2.21)
Diet high in sodium	Female	Denmark	0.28 (0 to 1.49)
Diet low in fruits	Male	Denmark	2.60 (1.60 to 3.74)
Diet low in fruits	Female	Denmark	2.92 (1.84 to 4.40)
Diet low in vegetables	Male	Denmark	2.94 (1.84 to 4.41)
Diet low in vegetables	Female	Denmark	3.05 (1.81 to 4.77)
High body-mass index	Male	Denmark	7.53 (3.89 to 13.06)
High body-mass index	Female	Denmark	8.50 (4.50 to 14.93)
High systolic blood pressure	Male	Denmark	19.13 (13.47 to 25.36)
High systolic blood pressure	Female	Denmark	21.07 (13.89 to 29.87)
Lead exposure	Male	Denmark	0.41 (-0.06 to 1.12)
Lead exposure	Female	Denmark	0.36 (-0.05 to 0.93)
Smoking	Male	Denmark	44.89 (36.72 to 52.97)

Smoking	Female	Denmark	33.35 (26.92 to 40.74)
Diet high in sodium	Male	Grenada	0.71 (0 to 2.72)
Diet high in sodium	Female	Grenada	0.40 (0 to 1.91)
Diet low in fruits	Male	Grenada	2.17 (1.41 to 3.12)
Diet low in fruits	Female	Grenada	2.30 (1.48 to 3.28)
Diet low in vegetables	Male	Grenada	4.97 (3.39 to 7.00)
Diet low in vegetables	Female	Grenada	5.01 (3.27 to 7.15)
High body-mass index	Male	Grenada	6.10 (3.21 to 10.09)
High body-mass index	Female	Grenada	8.69 (4.56 to 14.76)
High systolic blood pressure	Male	Grenada	16.46 (11.63 to 22.13)
High systolic blood pressure	Female	Grenada	16.92 (10.63 to 23.64)
Lead exposure	Male	Grenada	1.19 (-0.16 to 3.21)
Lead exposure	Female	Grenada	0.91 (-0.13 to 2.38)
Smoking	Male	Grenada	22.95 (17.76 to 29.05)
Smoking	Female	Grenada	8.39 (6.09 to 11.30)
Diet high in sodium	Male	Japan	1.19 (0.08 to 3.32)
Diet high in sodium	Female	Japan	0.86 (0.02 to 2.81)
Diet low in fruits	Male	Japan	4.22 (2.92 to 5.69)
Diet low in fruits	Female	Japan	3.86 (2.61 to 5.22)
Diet low in vegetables	Male	Japan	1.24 (0.63 to 2.03)
Diet low in vegetables	Female	Japan	1.34 (0.69 to 2.25)
High body-mass index	Male	Japan	4.55 (2.46 to 7.37)
High body-mass index	Female	Japan	4.45 (2.30 to 7.45)
High systolic blood pressure	Male	Japan	17.86 (13.28 to 23.53)
High systolic blood pressure	Female	Japan	17.19 (11.77 to 23.51)
Lead exposure	Male	Japan	0.35 (-0.04 to 0.95)
Lead exposure	Female	Japan	0.33 (-0.05 to 0.86)
Smoking	Male	Japan	42.02 (35.73 to 48.55)
Smoking	Female	Japan	11.64 (8.77 to 15.14)
Diet high in sodium	Male	Montenegro	1.78 (0.35 to 3.90)
Diet high in sodium	Female	Montenegro	1.12 (0.10 to 3.06)
Diet low in fruits	Male	Montenegro	1.48 (0.93 to 2.24)
Diet low in fruits	Female	Montenegro	1.70 (1.04 to 2.53)
Diet low in vegetables	Male	Montenegro	0.52 (0.16 to 1.16)
Diet low in vegetables	Female	Montenegro	0.47 (0.10 to 1.07)
High body-mass index	Male	Montenegro	12.91 (6.71 to 21.80)
High body-mass index	Female	Montenegro	12.95 (6.70 to 22.01)
High systolic blood pressure	Male	Montenegro	21.03 (15.24 to 27.56)
High systolic blood pressure	Female	Montenegro	21.76 (15.00 to 29.54)
Lead exposure	Male	Montenegro	0.38 (-0.05 to 1.05)
Lead exposure	Female	Montenegro	0.29 (-0.04 to 0.77)
Smoking	Male	Montenegro	49.16 (41.22 to 56.70)
Smoking	Female	Montenegro	24.78 (18.80 to 30.62)
Diet high in sodium	Male	Norway	0.55 (0 to 2.08)

Diet high in sodium	Female	Norway	0.18 (0 to 0.98)
Diet low in fruits	Male	Norway	2.80 (1.90 to 3.84)
Diet low in fruits	Female	Norway	3.16 (2.06 to 4.51)
Diet low in vegetables	Male	Norway	4.01 (2.65 to 5.74)
Diet low in vegetables	Female	Norway	4.25 (2.74 to 6.37)
High body-mass index	Male	Norway	7.11 (3.72 to 12.47)
High body-mass index	Female	Norway	8.26 (4.29 to 14.30)
High systolic blood pressure	Male	Norway	18.32 (13.37 to 24.10)
High systolic blood pressure	Female	Norway	20.60 (14.47 to 27.60)
Lead exposure	Male	Norway	0.48 (-0.06 to 1.24)
Lead exposure	Female	Norway	0.30 (-0.05 to 0.85)
Smoking	Male	Norway	27.81 (21.97 to 35.31)
Smoking	Female	Norway	16.10 (11.62 to 21.98)
Diet high in sodium	Male	Russian Federation	0.97 (0.06 to 2.88)
Diet high in sodium	Female	Russian Federation	0.42 (0 to 1.65)
Diet low in fruits	Male	Russian Federation	4.33 (3.04 to 5.78)
Diet low in fruits	Female	Russian Federation	4.56 (3.18 to 6.03)
Diet low in vegetables	Male	Russian Federation	3.22 (2.09 to 4.65)
Diet low in vegetables	Female	Russian Federation	3.11 (2.02 to 4.48)
High body-mass index	Male	Russian Federation	9.91 (5.18 to 17.21)
High body-mass index	Female	Russian Federation	13.15 (6.84 to 22.13)
High systolic blood pressure	Male	Russian Federation	19.64 (14.84 to 24.65)
High systolic blood pressure	Female	Russian Federation	20.86 (15.66 to 26.07)
Lead exposure	Male	Russian Federation	0.36 (-0.05 to 0.93)
Lead exposure	Female	Russian Federation	0.28 (-0.04 to 0.72)
Smoking	Male	Russian Federation	53.52 (46.94 to 60.34)
Smoking	Female	Russian Federation	10.66 (8.65 to 13.15)
Diet high in sodium	Male	Saint Lucia	0.68 (0 to 2.58)
Diet high in sodium	Female	Saint Lucia	0.37 (0 to 1.76)
Diet low in fruits	Male	Saint Lucia	2.77 (1.83 to 3.88)
Diet low in fruits	Female	Saint Lucia	2.92 (1.85 to 4.22)
Diet low in vegetables	Male	Saint Lucia	4.86 (3.24 to 6.83)
Diet low in vegetables	Female	Saint Lucia	4.95 (3.35 to 7.15)
High body-mass index	Male	Saint Lucia	6.14 (3.27 to 10.47)
High body-mass index	Female	Saint Lucia	7.95 (4.14 to 13.69)
High systolic blood pressure	Male	Saint Lucia	16.20 (11.08 to 21.75)
High systolic blood pressure	Female	Saint Lucia	17.07 (11.04 to 23.87)
Lead exposure	Male	Saint Lucia	1.17 (-0.16 to 3.06)
Lead exposure	Female	Saint Lucia	0.85 (-0.13 to 2.21)
Smoking	Male	Saint Lucia	24.46 (18.95 to 31.39)
Smoking	Female	Saint Lucia	8.48 (6.16 to 11.51)
Diet high in sodium	Male	Uruguay	1.11 (0.03 to 3.57)
Diet high in sodium	Female	Uruguay	0.68 (0 to 2.48)
Diet low in fruits	Male	Uruguay	3.12 (2.07 to 4.27)

Diet low in fruits	Female	Uruguay	3.36 (2.29 to 4.88)
Diet low in vegetables	Male	Uruguay	4.35 (3.01 to 6.16)
Diet low in vegetables	Female	Uruguay	4.47 (2.99 to 6.41)
High body-mass index	Male	Uruguay	9.02 (4.81 to 15.09)
High body-mass index	Female	Uruguay	10.21 (5.18 to 17.73)
High systolic blood pressure	Male	Uruguay	16.46 (11.64 to 21.88)
High systolic blood pressure	Female	Uruguay	18.05 (12.22 to 24.56)
Lead exposure	Male	Uruguay	0.84 (-0.11 to 2.18)
Lead exposure	Female	Uruguay	0.66 (-0.08 to 1.77)
Smoking	Male	Uruguay	41.04 (34.35 to 47.81)
Smoking	Female	Uruguay	23.23 (18.91 to 27.87)

AA, aortic aneurysm; ASDR, age-standardized death rates.

Fig 6E. World maps of percentage contribution of risk factors to AA-caused ASDR in 2021

Diet high in sodium

Risk factor	Location	Percent contribution to ASDR (%)
Diet high in sodium	People's Republic of China	2.16 (0.42 to 4.96)
Diet high in sodium	Republic of Korea	1.99 (0.25 to 5.12)
Diet high in sodium	Czech Republic	1.91 (0.33 to 4.48)
Diet high in sodium	Democratic People's Republic of Korea	1.81 (0.28 to 4.65)
Diet high in sodium	Bosnia and Herzegovina	1.77 (0.33 to 4.13)
Diet high in sodium	Republic of Bulgaria	1.77 (0.32 to 3.97)
Diet high in sodium	North Macedonia	1.64 (0.28 to 3.83)
Diet high in sodium	Republic of Singapore	1.63 (0.12 to 4.41)
Diet high in sodium	Slovak Republic	1.61 (0.30 to 3.75)
Diet high in sodium	Montenegro	1.60 (0.29 to 3.61)
Diet high in sodium	Republic of Slovenia	1.60 (0.30 to 3.55)
Diet high in sodium	Kingdom of Cambodia	1.59 (0.14 to 4.40)
Diet high in sodium	Republic of Albania	1.59 (0.28 to 3.62)
Diet high in sodium	Republic of Croatia	1.58 (0.29 to 3.66)
Diet high in sodium	Republic of Serbia	1.52 (0.29 to 3.36)
Diet high in sodium	Kingdom of Thailand	1.50 (0.14 to 4.14)
Diet high in sodium	Hungary	1.47 (0.29 to 3.28)
Diet high in sodium	Republic of the Philippines	1.45 (0.15 to 4.02)
Diet high in sodium	Brunei Darussalam	1.44 (0.13 to 4.05)
Diet high in sodium	Lao People's Democratic Republic	1.43 (0.13 to 4.01)
Diet high in sodium	Romania	1.42 (0.26 to 3.30)
Diet high in sodium	Independent State of Papua New Guinea	1.41 (0.09 to 4.18)
Diet high in sodium	Republic of Colombia	1.41 (0.22 to 3.44)
Diet high in sodium	Republic of Poland	1.40 (0.16 to 3.74)

Diet high in sodium	Solomon Islands	1.40 (0.10 to 4.14)
Diet high in sodium	Republic of Mauritius	1.39 (0.16 to 3.74)
Diet high in sodium	Republic of the Marshall Islands	1.34 (0.08 to 4.13)
Diet high in sodium	Republic of Kiribati	1.33 (0.11 to 3.94)
Diet high in sodium	Republic of the Union of Myanmar	1.32 (0.14 to 3.50)
Diet high in sodium	Federated States of Micronesia	1.31 (0.08 to 3.94)
Diet high in sodium	Democratic Socialist Republic of Sri Lanka	1.31 (0.12 to 3.63)
Diet high in sodium	Kingdom of Tonga	1.30 (0.10 to 3.77)
Diet high in sodium	Republic of Maldives	1.28 (0.11 to 3.48)
Diet high in sodium	Cook Islands	1.27 (0.08 to 3.73)
Diet high in sodium	Democratic Republic of Timor-Leste	1.26 (0.12 to 3.47)
Diet high in sodium	Guam	1.24 (0.10 to 3.52)
Diet high in sodium	Tuvalu	1.24 (0.08 to 3.65)
Diet high in sodium	Tokelau	1.24 (0.08 to 3.88)
Diet high in sodium	Northern Mariana Islands	1.22 (0.08 to 3.59)
Diet high in sodium	Republic of Palau	1.22 (0.07 to 3.62)
Diet high in sodium	Socialist Republic of Viet Nam	1.20 (0.12 to 3.35)
Diet high in sodium	Republic of Seychelles	1.18 (0.11 to 3.25)
Diet high in sodium	Republic of Fiji	1.15 (0.08 to 3.39)
Diet high in sodium	Malaysia	1.15 (0.12 to 3.10)
Diet high in sodium	Republic of Guatemala	1.12 (0.05 to 3.50)
Diet high in sodium	Taiwan (Province of China)	1.11 (0.06 to 3.39)
Diet high in sodium	Republic of El Salvador	1.09 (0.06 to 3.25)
Diet high in sodium	Republic of Nauru	1.09 (0.07 to 3.26)
Diet high in sodium	Republic of Nicaragua	1.08 (0.06 to 3.35)
Diet high in sodium	Republic of Honduras	1.05 (0.04 to 3.21)
Diet high in sodium	Japan	1.05 (0.06 to 3.16)
Diet high in sodium	Republic of Panama	1.03 (0.05 to 3.16)
Diet high in sodium	Republic of Niue	1.03 (0.08 to 2.88)
Diet high in sodium	Federal Democratic Republic of Ethiopia	1.02 (0.03 to 3.43)
Diet high in sodium	Republic of Ecuador	1.00 (0.02 to 3.47)
Diet high in sodium	Plurinational State of Bolivia	0.97 (0.02 to 3.36)
Diet high in sodium	Eastern Republic of Uruguay	0.96 (0.02 to 3.19)
Diet high in sodium	Republic of Costa Rica	0.96 (0.06 to 2.85)
Diet high in sodium	Argentine Republic	0.95 (0.02 to 3.29)
Diet high in sodium	Republic of Zambia	0.95 (0.02 to 3.30)
Diet high in sodium	State of Eritrea	0.94 (0.02 to 3.27)
Diet high in sodium	Republic of Rwanda	0.94 (0.02 to 3.26)
Diet high in sodium	Republic of Tajikistan	0.93 (0.04 to 3.04)
Diet high in sodium	Republic of Peru	0.92 (0.02 to 3.22)
Diet high in sodium	Federal Republic of Somalia	0.92 (0.01 to 3.01)
Diet high in sodium	Kyrgyz Republic	0.92 (0.03 to 3.11)
Diet high in sodium	Republic of Paraguay	0.91 (0.02 to 2.99)
Diet high in sodium	Bolivarian Republic of Venezuela	0.91 (0.05 to 2.61)

Diet high in sodium	Republic of Vanuatu	0.89 (0.07 to 2.55)
Diet high in sodium	Republic of Uzbekistan	0.89 (0.03 to 2.94)
Diet high in sodium	United Republic of Tanzania	0.87 (0.05 to 2.60)
Diet high in sodium	Republic of South Sudan	0.87 (0.01 to 3.05)
Diet high in sodium	Federal Democratic Republic of Nepal	0.86 (0.01 to 3.20)
Diet high in sodium	Union of the Comoros	0.85 (0.02 to 2.80)
Diet high in sodium	Republic of Austria	0.85 (0.02 to 2.66)
Diet high in sodium	Republic of Burundi	0.84 (0.02 to 2.85)
Diet high in sodium	Republic of Azerbaijan	0.83 (0.03 to 2.88)
Diet high in sodium	Republic of Indonesia	0.83 (0.09 to 2.15)
Diet high in sodium	Republic of Madagascar	0.83 (0.01 to 2.85)
Diet high in sodium	Federative Republic of Brazil	0.82 (0.02 to 2.72)
Diet high in sodium	Republic of Armenia	0.82 (0.03 to 2.77)
Diet high in sodium	Republic of Djibouti	0.79 (0.01 to 2.66)
Diet high in sodium	Republic of Uganda	0.79 (0.01 to 2.78)
Diet high in sodium	People's Republic of Bangladesh	0.79 (0.01 to 2.84)
Diet high in sodium	Kingdom of Bhutan	0.79 (0.00 to 2.85)
Diet high in sodium	Russian Federation	0.78 (0.04 to 2.42)
Diet high in sodium	Republic of Italy	0.77 (0.03 to 2.63)
Diet high in sodium	Republic of Malawi	0.76 (0.01 to 2.47)
Diet high in sodium	Kingdom of Belgium	0.76 (0.01 to 2.64)
Diet high in sodium	Turkmenistan	0.76 (0.03 to 2.61)
Diet high in sodium	Republic of Mozambique	0.75 (0.02 to 2.50)
Diet high in sodium	Republic of Chile	0.74 (0.02 to 2.41)
Diet high in sodium	Canada	0.72 (0.01 to 2.74)
Diet high in sodium	Islamic Republic of Pakistan	0.71 (0.01 to 2.59)
Diet high in sodium	Republic of Malta	0.71 (0.02 to 2.56)
Diet high in sodium	Mongolia	0.71 (0.02 to 2.33)
Diet high in sodium	Georgia	0.70 (0.03 to 2.17)
Diet high in sodium	Republic of India	0.70 (0.02 to 2.61)
Diet high in sodium	United Mexican States	0.69 (0.01 to 2.45)
Diet high in sodium	United States of America	0.69 (0.01 to 2.59)
Diet high in sodium	Republic of Suriname	0.68 (0.00 to 2.82)
Diet high in sodium	Greenland	0.67 (0.00 to 2.48)
Diet high in sodium	Republic of Cuba	0.65 (0.00 to 2.65)
Diet high in sodium	American Samoa	0.64 (0.01 to 2.47)
Diet high in sodium	Saint Vincent and the Grenadines	0.64 (0.00 to 2.55)
Diet high in sodium	Republic of Iceland	0.64 (0.01 to 2.46)
Diet high in sodium	Hellenic Republic	0.63 (0.00 to 2.57)
Diet high in sodium	Bermuda	0.62 (0.00 to 2.49)
Diet high in sodium	Portuguese Republic	0.62 (0.01 to 2.46)
Diet high in sodium	Republic of Kazakhstan	0.62 (0.02 to 2.10)
Diet high in sodium	Antigua and Barbuda	0.61 (0.00 to 2.52)
Diet high in sodium	Jamaica	0.61 (0.00 to 2.47)

Diet high in sodium	Dominican Republic	0.61 (0.00 to 2.45)
Diet high in sodium	Republic of Ghana	0.60 (0.00 to 2.26)
Diet high in sodium	United States Virgin Islands	0.59 (0.00 to 2.28)
Diet high in sodium	Puerto Rico	0.58 (0.00 to 2.47)
Diet high in sodium	Saint Kitts and Nevis	0.58 (0.00 to 2.35)
Diet high in sodium	Federal Republic of Germany	0.58 (0.01 to 2.19)
Diet high in sodium	Republic of San Marino	0.57 (0.00 to 2.26)
Diet high in sodium	Swiss Confederation	0.57 (0.00 to 2.38)
Diet high in sodium	Grenada	0.57 (0.00 to 2.29)
Diet high in sodium	Republic of Latvia	0.56 (0.01 to 2.02)
Diet high in sodium	Commonwealth of Dominica	0.56 (0.00 to 2.35)
Diet high in sodium	Independent State of Samoa	0.56 (0.00 to 2.33)
Diet high in sodium	Republic of Guyana	0.56 (0.00 to 2.32)
Diet high in sodium	Belize	0.56 (0.00 to 2.35)
Diet high in sodium	Republic of Haiti	0.55 (0.00 to 2.29)
Diet high in sodium	Commonwealth of the Bahamas	0.55 (0.00 to 2.36)
Diet high in sodium	Saint Lucia	0.52 (0.00 to 2.15)
Diet high in sodium	Republic of Cyprus	0.52 (0.00 to 2.14)
Diet high in sodium	Kingdom of Sweden	0.52 (0.00 to 2.03)
Diet high in sodium	State of Kuwait	0.51 (0.00 to 2.26)
Diet high in sodium	Republic of Benin	0.51 (0.00 to 2.19)
Diet high in sodium	State of Israel	0.50 (0.00 to 2.02)
Diet high in sodium	Grand Duchy of Luxembourg	0.49 (0.00 to 2.05)
Diet high in sodium	New Zealand	0.49 (0.00 to 2.19)
Diet high in sodium	Republic of Finland	0.49 (0.00 to 1.94)
Diet high in sodium	Principality of Monaco	0.48 (0.00 to 2.00)
Diet high in sodium	Kingdom of Denmark	0.47 (0.00 to 1.91)
Diet high in sodium	French Republic	0.47 (0.00 to 1.99)
Diet high in sodium	United Kingdom of Great Britain and Northern Ireland	0.47 (0.00 to 2.06)
Diet high in sodium	Republic of Chad	0.47 (0.00 to 2.09)
Diet high in sodium	Burkina Faso	0.46 (0.00 to 2.09)
Diet high in sodium	Republic of Guinea	0.46 (0.00 to 2.05)
Diet high in sodium	Republic of Trinidad and Tobago	0.45 (0.00 to 1.86)
Diet high in sodium	Republic of Côte d'Ivoire	0.44 (0.00 to 1.93)
Diet high in sodium	Kingdom of the Netherlands	0.44 (0.00 to 1.88)
Diet high in sodium	Republic of Cameroon	0.42 (0.00 to 1.96)
Diet high in sodium	Islamic Republic of Mauritania	0.42 (0.00 to 1.90)
Diet high in sodium	Republic of Mali	0.42 (0.00 to 1.84)
Diet high in sodium	Ukraine	0.42 (0.00 to 1.80)
Diet high in sodium	Republic of Guinea-Bissau	0.42 (0.00 to 1.90)
Diet high in sodium	Republic of the Niger	0.41 (0.00 to 1.92)
Diet high in sodium	Republic of Lithuania	0.41 (0.00 to 1.72)
Diet high in sodium	Republic of Liberia	0.40 (0.00 to 1.78)
Diet high in sodium	Kingdom of Norway	0.40 (0.00 to 1.65)

Diet high in sodium	Republic of Kenya	0.39 (0.01 to 1.45)
Diet high in sodium	Republic of the Gambia	0.39 (0.00 to 1.71)
Diet high in sodium	Gabonese Republic	0.39 (0.00 to 1.87)
Diet high in sodium	Republic of Cabo Verde	0.39 (0.00 to 1.60)
Diet high in sodium	Republic of Zimbabwe	0.38 (0.00 to 1.75)
Diet high in sodium	Principality of Andorra	0.38 (0.00 to 1.70)
Diet high in sodium	Togolese Republic	0.37 (0.00 to 1.68)
Diet high in sodium	Democratic Republic of Sao Tome and Principe	0.37 (0.00 to 1.69)
Diet high in sodium	Republic of Belarus	0.37 (0.00 to 1.59)
Diet high in sodium	Barbados	0.36 (0.00 to 1.78)
Diet high in sodium	Republic of Senegal	0.36 (0.00 to 1.56)
Diet high in sodium	Republic of Angola	0.36 (0.00 to 1.76)
Diet high in sodium	Central African Republic	0.36 (0.00 to 1.75)
Diet high in sodium	Republic of Moldova	0.35 (0.00 to 1.62)
Diet high in sodium	Republic of the Congo	0.35 (0.00 to 1.63)
Diet high in sodium	Republic of Equatorial Guinea	0.34 (0.00 to 1.62)
Diet high in sodium	Ireland	0.33 (0.00 to 1.64)
Diet high in sodium	Kingdom of Spain	0.32 (0.00 to 1.47)
Diet high in sodium	Republic of Sierra Leone	0.30 (0.00 to 1.33)
Diet high in sodium	Federal Republic of Nigeria	0.30 (0.00 to 1.36)
Diet high in sodium	Kingdom of Lesotho	0.29 (0.00 to 1.64)
Diet high in sodium	Australia	0.29 (0.00 to 1.59)
Diet high in sodium	Republic of Namibia	0.27 (0.00 to 1.46)
Diet high in sodium	Kingdom of Eswatini	0.27 (0.00 to 1.44)
Diet high in sodium	Republic of Estonia	0.26 (0.00 to 1.35)
Diet high in sodium	Republic of Botswana	0.25 (0.00 to 1.39)
Diet high in sodium	Arab Republic of Egypt	0.24 (0.00 to 1.40)
Diet high in sodium	Sultanate of Oman	0.24 (0.00 to 1.34)
Diet high in sodium	Palestine	0.24 (0.00 to 1.35)
Diet high in sodium	Hashemite Kingdom of Jordan	0.23 (0.00 to 1.35)
Diet high in sodium	Republic of Yemen	0.23 (0.00 to 1.35)
Diet high in sodium	Islamic Republic of Iran	0.23 (0.00 to 1.29)
Diet high in sodium	Republic of Tunisia	0.23 (0.00 to 1.33)
Diet high in sodium	Islamic Republic of Afghanistan	0.23 (0.00 to 1.35)
Diet high in sodium	Syrian Arab Republic	0.23 (0.00 to 1.32)
Diet high in sodium	Lebanese Republic	0.22 (0.00 to 1.27)
Diet high in sodium	State of Libya	0.21 (0.00 to 1.15)
Diet high in sodium	Kingdom of Bahrain	0.21 (0.00 to 1.14)
Diet high in sodium	State of Qatar	0.21 (0.00 to 1.15)
Diet high in sodium	Republic of South Africa	0.21 (0.00 to 1.08)
Diet high in sodium	People's Democratic Republic of Algeria	0.21 (0.00 to 1.22)
Diet high in sodium	Republic of Sudan	0.20 (0.00 to 1.14)
Diet high in sodium	Kingdom of Morocco	0.20 (0.00 to 1.12)
Diet high in sodium	United Arab Emirates	0.20 (0.00 to 1.27)

Diet high in sodium	Republic of Iraq	0.19 (0.00 to 1.10)
Diet high in sodium	Kingdom of Saudi Arabia	0.19 (0.00 to 1.16)
Diet high in sodium	Democratic Republic of the Congo	0.19 (0.00 to 1.23)
Diet high in sodium	Republic of Turkey	0.16 (0.00 to 1.06)

Diet low in fruits

Risk factor	Location	Percent contribution to ASDR (%)
Diet low in fruits	Togolese Republic	6.68 (4.67 to 8.89)
Diet low in fruits	Republic of Zimbabwe	6.63 (4.57 to 8.86)
Diet low in fruits	Republic of the Gambia	6.62 (4.56 to 8.85)
Diet low in fruits	Mongolia	6.47 (4.42 to 8.59)
Diet low in fruits	Islamic Republic of Mauritania	6.26 (4.28 to 8.42)
Diet low in fruits	Burkina Faso	6.06 (4.13 to 8.19)
Diet low in fruits	Republic of Sierra Leone	6.02 (4.21 to 8.03)
Diet low in fruits	Republic of Senegal	5.99 (4.14 to 8.02)
Diet low in fruits	Republic of Djibouti	5.98 (4.17 to 7.95)
Diet low in fruits	Republic of Botswana	5.86 (4.11 to 7.99)
Diet low in fruits	Republic of the Niger	5.82 (3.94 to 8.04)
Diet low in fruits	Republic of Mozambique	5.77 (3.99 to 7.73)
Diet low in fruits	Republic of South Africa	5.69 (3.96 to 7.55)
Diet low in fruits	Democratic Republic of Timor-Leste	5.66 (3.89 to 7.51)
Diet low in fruits	Republic of India	5.65 (3.94 to 7.38)
Diet low in fruits	Republic of Chad	5.64 (3.95 to 7.73)
Diet low in fruits	Kingdom of Lesotho	5.63 (3.77 to 7.62)
Diet low in fruits	Republic of Fiji	5.51 (3.75 to 7.49)
Diet low in fruits	Federal Republic of Somalia	5.49 (3.83 to 7.41)
Diet low in fruits	Ukraine	5.49 (3.87 to 7.38)
Diet low in fruits	Republic of Nicaragua	5.48 (3.80 to 7.35)
Diet low in fruits	Republic of Latvia	5.44 (3.75 to 7.24)
Diet low in fruits	Republic of Namibia	5.44 (3.78 to 7.38)
Diet low in fruits	Republic of Zambia	5.42 (3.81 to 7.18)
Diet low in fruits	People's Republic of Bangladesh	5.33 (3.70 to 7.16)
Diet low in fruits	Republic of Indonesia	5.29 (3.62 to 7.17)
Diet low in fruits	Republic of Benin	5.26 (3.66 to 6.97)
Diet low in fruits	Republic of Liberia	5.24 (3.67 to 7.11)
Diet low in fruits	Republic of Moldova	5.21 (3.55 to 6.97)
Diet low in fruits	Republic of Mauritius	5.20 (3.60 to 6.89)
Diet low in fruits	Republic of Cabo Verde	5.15 (3.60 to 6.91)
Diet low in fruits	Republic of Mali	5.10 (3.50 to 6.84)
Diet low in fruits	Federal Democratic Republic of Ethiopia	5.08 (3.52 to 6.87)
Diet low in fruits	Hungary	5.04 (3.53 to 6.77)
Diet low in fruits	Georgia	5.01 (3.40 to 6.77)
Diet low in fruits	Republic of Kazakhstan	5.00 (3.50 to 6.61)

Diet low in fruits	Central African Republic	4.99 (3.47 to 6.80)
Diet low in fruits	Republic of the Union of Myanmar	4.97 (3.55 to 6.67)
Diet low in fruits	Republic of Tajikistan	4.96 (3.49 to 6.64)
Diet low in fruits	Kingdom of Eswatini	4.95 (3.41 to 6.61)
Diet low in fruits	Islamic Republic of Pakistan	4.94 (3.45 to 6.52)
Diet low in fruits	Republic of Guinea-Bissau	4.90 (3.39 to 6.66)
Diet low in fruits	Democratic Socialist Republic of Sri Lanka	4.89 (3.37 to 6.60)
Diet low in fruits	Republic of Lithuania	4.87 (3.31 to 6.40)
Diet low in fruits	Saint Kitts and Nevis	4.83 (3.30 to 6.50)
Diet low in fruits	Republic of Trinidad and Tobago	4.82 (3.25 to 6.42)
Diet low in fruits	Kyrgyz Republic	4.80 (3.35 to 6.33)
Diet low in fruits	Democratic Republic of the Congo	4.79 (3.36 to 6.40)
Diet low in fruits	Republic of Madagascar	4.78 (3.33 to 6.52)
Diet low in fruits	Federal Republic of Nigeria	4.76 (3.31 to 6.30)
Diet low in fruits	Kingdom of Cambodia	4.76 (3.28 to 6.45)
Diet low in fruits	Slovak Republic	4.74 (3.27 to 6.38)
Diet low in fruits	Republic of Belarus	4.71 (3.28 to 6.19)
Diet low in fruits	Republic of Malawi	4.69 (3.23 to 6.20)
Diet low in fruits	Republic of the Congo	4.66 (3.22 to 6.21)
Diet low in fruits	State of Eritrea	4.66 (3.15 to 6.45)
Diet low in fruits	Republic of Estonia	4.66 (3.30 to 6.26)
Diet low in fruits	Barbados	4.63 (3.20 to 6.22)
Diet low in fruits	Republic of Yemen	4.62 (3.20 to 6.13)
Diet low in fruits	Republic of Bulgaria	4.60 (3.13 to 6.04)
Diet low in fruits	Islamic Republic of Afghanistan	4.57 (3.19 to 6.11)
Diet low in fruits	Republic of Kenya	4.56 (3.20 to 6.10)
Diet low in fruits	Republic of South Sudan	4.49 (3.07 to 6.04)
Diet low in fruits	Brunei Darussalam	4.47 (3.12 to 5.93)
Diet low in fruits	Russian Federation	4.47 (3.13 to 5.92)
Diet low in fruits	Tuvalu	4.39 (2.99 to 5.94)
Diet low in fruits	Kingdom of Bhutan	4.38 (3.04 to 5.87)
Diet low in fruits	Socialist Republic of Viet Nam	4.34 (2.98 to 5.77)
Diet low in fruits	Republic of Angola	4.32 (2.98 to 5.78)
Diet low in fruits	Republic of Vanuatu	4.32 (2.83 to 6.00)
Diet low in fruits	Kingdom of Tonga	4.29 (2.99 to 5.78)
Diet low in fruits	Czech Republic	4.28 (3.01 to 5.77)
Diet low in fruits	Republic of Iraq	4.22 (2.93 to 5.50)
Diet low in fruits	Republic of Finland	4.21 (2.91 to 5.75)
Diet low in fruits	Republic of Maldives	4.20 (2.92 to 5.69)
Diet low in fruits	Tokelau	4.17 (2.76 to 5.74)
Diet low in fruits	Republic of Guyana	4.15 (2.85 to 5.58)
Diet low in fruits	Republic of the Marshall Islands	4.12 (2.84 to 5.62)
Diet low in fruits	Independent State of Samoa	4.11 (2.85 to 5.59)
Diet low in fruits	Japan	4.11 (2.78 to 5.46)

Diet low in fruits	Solomon Islands	4.04 (2.83 to 5.45)
Diet low in fruits	Republic of Sudan	4.04 (2.76 to 5.51)
Diet low in fruits	Republic of Côte d'Ivoire	4.03 (2.78 to 5.37)
Diet low in fruits	Republic of Chile	4.03 (2.73 to 5.42)
Diet low in fruits	Republic of Poland	4.01 (2.80 to 5.31)
Diet low in fruits	French Republic	3.98 (2.77 to 5.37)
Diet low in fruits	Republic of Nauru	3.96 (2.68 to 5.48)
Diet low in fruits	Republic of Haiti	3.94 (2.69 to 5.25)
Diet low in fruits	Federated States of Micronesia	3.93 (2.69 to 5.32)
Diet low in fruits	Democratic People's Republic of Korea	3.92 (2.71 to 5.30)
Diet low in fruits	Republic of Niue	3.90 (2.70 to 5.33)
Diet low in fruits	Republic of Panama	3.90 (2.71 to 5.23)
Diet low in fruits	Malaysia	3.89 (2.64 to 5.28)
Diet low in fruits	Republic of Seychelles	3.88 (2.68 to 5.24)
Diet low in fruits	Republic of El Salvador	3.88 (2.75 to 5.12)
Diet low in fruits	Republic of Kiribati	3.83 (2.65 to 5.12)
Diet low in fruits	American Samoa	3.80 (2.57 to 5.18)
Diet low in fruits	Romania	3.78 (2.63 to 5.07)
Diet low in fruits	Federal Republic of Germany	3.78 (2.61 to 5.02)
Diet low in fruits	Republic of Guatemala	3.70 (2.52 to 4.86)
Diet low in fruits	Federal Democratic Republic of Nepal	3.70 (2.58 to 5.02)
Diet low in fruits	United Kingdom of Great Britain and Northern Ireland	3.66 (2.55 to 4.86)
Diet low in fruits	United Republic of Tanzania	3.64 (2.56 to 4.90)
Diet low in fruits	Republic of Palau	3.63 (2.42 to 4.93)
Diet low in fruits	Republic of Iceland	3.62 (2.54 to 4.85)
Diet low in fruits	Lao People's Democratic Republic	3.59 (2.46 to 4.83)
Diet low in fruits	Independent State of Papua New Guinea	3.58 (2.44 to 4.86)
Diet low in fruits	Hashemite Kingdom of Jordan	3.55 (2.44 to 4.70)
Diet low in fruits	Republic of Honduras	3.55 (2.41 to 4.72)
Diet low in fruits	Republic of Uzbekistan	3.51 (2.47 to 4.72)
Diet low in fruits	Australia	3.50 (2.42 to 4.73)
Diet low in fruits	Turkmenistan	3.47 (2.40 to 4.63)
Diet low in fruits	Ireland	3.44 (2.38 to 4.76)
Diet low in fruits	Bosnia and Herzegovina	3.43 (2.36 to 4.68)
Diet low in fruits	Bolivarian Republic of Venezuela	3.41 (2.35 to 4.65)
Diet low in fruits	Kingdom of Belgium	3.39 (2.29 to 4.58)
Diet low in fruits	Bermuda	3.36 (2.28 to 4.55)
Diet low in fruits	Grand Duchy of Luxembourg	3.36 (2.30 to 4.57)
Diet low in fruits	Union of the Comoros	3.33 (2.31 to 4.46)
Diet low in fruits	People's Republic of China	3.32 (2.27 to 4.50)
Diet low in fruits	Republic of Cameroon	3.31 (2.22 to 4.44)
Diet low in fruits	Palestine	3.31 (2.26 to 4.48)
Diet low in fruits	Republic of Malta	3.31 (2.25 to 4.43)
Diet low in fruits	Kingdom of Sweden	3.30 (2.23 to 4.47)

Diet low in fruits	Republic of Croatia	3.25 (2.22 to 4.43)
Diet low in fruits	Eastern Republic of Uruguay	3.22 (2.16 to 4.39)
Diet low in fruits	Kingdom of Saudi Arabia	3.19 (2.22 to 4.28)
Diet low in fruits	Republic of Peru	3.17 (2.17 to 4.26)
Diet low in fruits	Republic of Suriname	3.14 (2.19 to 4.25)
Diet low in fruits	Commonwealth of the Bahamas	3.13 (2.12 to 4.21)
Diet low in fruits	United States of America	3.13 (2.15 to 4.14)
Diet low in fruits	State of Libya	3.11 (2.15 to 4.25)
Diet low in fruits	Kingdom of the Netherlands	3.10 (2.07 to 4.24)
Diet low in fruits	Republic of Guinea	3.10 (2.09 to 4.27)
Diet low in fruits	Cook Islands	3.09 (2.12 to 4.23)
Diet low in fruits	State of Kuwait	3.06 (2.09 to 4.08)
Diet low in fruits	Northern Mariana Islands	3.04 (2.07 to 4.23)
Diet low in fruits	Republic of Equatorial Guinea	3.01 (1.96 to 4.25)
Diet low in fruits	Republic of Azerbaijan	2.98 (2.02 to 4.08)
Diet low in fruits	Republic of Korea	2.96 (2.07 to 3.93)
Diet low in fruits	Kingdom of Norway	2.96 (2.00 to 4.08)
Diet low in fruits	Swiss Confederation	2.92 (2.02 to 4.01)
Diet low in fruits	Canada	2.92 (1.99 to 3.95)
Diet low in fruits	Plurinational State of Bolivia	2.92 (1.98 to 3.95)
Diet low in fruits	Republic of Colombia	2.88 (1.98 to 3.81)
Diet low in fruits	Jamaica	2.86 (1.98 to 3.78)
Diet low in fruits	Saint Lucia	2.86 (1.89 to 3.88)
Diet low in fruits	New Zealand	2.81 (1.95 to 3.78)
Diet low in fruits	Republic of Cyprus	2.80 (1.91 to 3.75)
Diet low in fruits	Guam	2.74 (1.85 to 3.78)
Diet low in fruits	Republic of Slovenia	2.73 (1.91 to 3.76)
Diet low in fruits	Republic of San Marino	2.72 (1.80 to 3.83)
Diet low in fruits	Kingdom of Denmark	2.71 (1.83 to 3.81)
Diet low in fruits	North Macedonia	2.70 (1.84 to 3.62)
Diet low in fruits	Puerto Rico	2.69 (1.81 to 3.69)
Diet low in fruits	Republic of the Philippines	2.69 (1.80 to 3.70)
Diet low in fruits	Syrian Arab Republic	2.68 (1.80 to 3.61)
Diet low in fruits	People's Democratic Republic of Algeria	2.67 (1.87 to 3.59)
Diet low in fruits	Republic of Paraguay	2.60 (1.73 to 3.52)
Diet low in fruits	Portuguese Republic	2.59 (1.80 to 3.50)
Diet low in fruits	Greenland	2.54 (1.75 to 3.60)
Diet low in fruits	Republic of Costa Rica	2.49 (1.68 to 3.36)
Diet low in fruits	Republic of Serbia	2.48 (1.65 to 3.40)
Diet low in fruits	Principality of Andorra	2.48 (1.54 to 3.52)
Diet low in fruits	United Mexican States	2.47 (1.69 to 3.38)
Diet low in fruits	Antigua and Barbuda	2.44 (1.65 to 3.36)
Diet low in fruits	Republic of Burundi	2.41 (1.63 to 3.35)
Diet low in fruits	Democratic Republic of Sao Tome and Principe	2.40 (1.60 to 3.39)

Diet low in fruits	Republic of Armenia	2.38 (1.62 to 3.34)
Diet low in fruits	Federative Republic of Brazil	2.34 (1.58 to 3.12)
Diet low in fruits	Saint Vincent and the Grenadines	2.30 (1.57 to 3.19)
Diet low in fruits	Republic of Austria	2.29 (1.55 to 3.20)
Diet low in fruits	Grenada	2.27 (1.51 to 3.09)
Diet low in fruits	United Arab Emirates	2.14 (1.43 to 2.98)
Diet low in fruits	Republic of Tunisia	2.13 (1.44 to 2.91)
Diet low in fruits	Argentine Republic	2.10 (1.42 to 2.92)
Diet low in fruits	Kingdom of Thailand	2.07 (1.41 to 2.83)
Diet low in fruits	Republic of Cuba	2.07 (1.40 to 2.77)
Diet low in fruits	Taiwan (Province of China)	2.04 (1.34 to 2.78)
Diet low in fruits	Republic of Ghana	1.90 (1.27 to 2.67)
Diet low in fruits	Republic of Singapore	1.87 (1.21 to 2.56)
Diet low in fruits	Gabonese Republic	1.85 (1.22 to 2.60)
Diet low in fruits	Kingdom of Morocco	1.84 (1.23 to 2.59)
Diet low in fruits	Kingdom of Spain	1.75 (1.15 to 2.47)
Diet low in fruits	Republic of Albania	1.75 (1.08 to 2.47)
Diet low in fruits	United States Virgin Islands	1.75 (1.14 to 2.55)
Diet low in fruits	Republic of Italy	1.67 (1.12 to 2.24)
Diet low in fruits	Arab Republic of Egypt	1.61 (1.05 to 2.21)
Diet low in fruits	Kingdom of Bahrain	1.60 (1.00 to 2.45)
Diet low in fruits	Montenegro	1.54 (1.01 to 2.18)
Diet low in fruits	State of Israel	1.49 (0.99 to 2.13)
Diet low in fruits	Sultanate of Oman	1.47 (0.95 to 2.09)
Diet low in fruits	Lebanese Republic	1.43 (0.92 to 2.05)
Diet low in fruits	Republic of Uganda	1.40 (0.92 to 2.03)
Diet low in fruits	Hellenic Republic	1.22 (0.79 to 1.74)
Diet low in fruits	Republic of Ecuador	1.20 (0.75 to 1.72)
Diet low in fruits	Islamic Republic of Iran	1.16 (0.79 to 1.60)
Diet low in fruits	Principality of Monaco	1.11 (0.63 to 1.74)
Diet low in fruits	Dominican Republic	0.86 (0.51 to 1.28)
Diet low in fruits	Republic of Turkey	0.86 (0.56 to 1.23)
Diet low in fruits	Belize	0.85 (0.49 to 1.27)
Diet low in fruits	State of Qatar	0.81 (0.44 to 1.32)
Diet low in fruits	Commonwealth of Dominica	0.42 (0.24 to 0.70)
Diet low in fruits	Republic of Rwanda	0.12 (0.05 to 0.24)

Diet low in vegetables

Risk factor	Location	Percent contribution to ASDR (%)
Diet low in vegetables	Republic of Zimbabwe	6.57 (4.46 to 9.16)
Diet low in vegetables	Togolese Republic	6.31 (4.37 to 8.75)
Diet low in vegetables	Republic of Sierra Leone	6.12 (4.09 to 8.64)
Diet low in vegetables	Republic of Guinea-Bissau	6.08 (4.11 to 8.47)

Diet low in vegetables	Republic of the Congo	6.08 (4.12 to 8.45)
Diet low in vegetables	Republic of Vanuatu	6.06 (4.12 to 8.54)
Diet low in vegetables	Republic of the Gambia	6.01 (4.04 to 8.38)
Diet low in vegetables	Republic of Liberia	6.00 (4.01 to 8.52)
Diet low in vegetables	Democratic Republic of Sao Tome and Principe	6.00 (4.00 to 8.32)
Diet low in vegetables	Republic of Mozambique	5.98 (4.02 to 8.49)
Diet low in vegetables	Central African Republic	5.96 (4.05 to 8.36)
Diet low in vegetables	Islamic Republic of Mauritania	5.92 (4.00 to 8.39)
Diet low in vegetables	Union of the Comoros	5.92 (4.04 to 8.15)
Diet low in vegetables	Kingdom of Eswatini	5.90 (4.01 to 8.05)
Diet low in vegetables	Republic of Malawi	5.89 (3.98 to 8.07)
Diet low in vegetables	Republic of Indonesia	5.87 (3.94 to 8.21)
Diet low in vegetables	Republic of Nicaragua	5.84 (3.96 to 8.00)
Diet low in vegetables	Republic of Haiti	5.77 (3.87 to 7.89)
Diet low in vegetables	Republic of Equatorial Guinea	5.72 (3.90 to 7.95)
Diet low in vegetables	Federal Republic of Somalia	5.71 (3.84 to 8.10)
Diet low in vegetables	Republic of Madagascar	5.66 (3.91 to 7.86)
Diet low in vegetables	Democratic Republic of the Congo	5.65 (3.79 to 7.73)
Diet low in vegetables	Republic of Burundi	5.64 (3.80 to 7.98)
Diet low in vegetables	Republic of Chad	5.63 (3.87 to 7.94)
Diet low in vegetables	Burkina Faso	5.58 (3.74 to 7.95)
Diet low in vegetables	Democratic Republic of Timor-Leste	5.56 (3.71 to 7.61)
Diet low in vegetables	Republic of Mali	5.55 (3.74 to 7.86)
Diet low in vegetables	Gabonese Republic	5.52 (3.75 to 7.67)
Diet low in vegetables	Republic of Trinidad and Tobago	5.51 (3.72 to 7.71)
Diet low in vegetables	State of Eritrea	5.50 (3.68 to 7.92)
Diet low in vegetables	Republic of Uganda	5.48 (3.75 to 7.50)
Diet low in vegetables	Independent State of Samoa	5.47 (3.71 to 7.60)
Diet low in vegetables	Republic of Botswana	5.44 (3.71 to 7.61)
Diet low in vegetables	Kingdom of Lesotho	5.43 (3.56 to 7.69)
Diet low in vegetables	Republic of Côte d'Ivoire	5.39 (3.66 to 7.51)
Diet low in vegetables	Islamic Republic of Afghanistan	5.39 (3.71 to 7.44)
Diet low in vegetables	Kingdom of Tonga	5.34 (3.59 to 7.40)
Diet low in vegetables	Republic of Senegal	5.32 (3.54 to 7.57)
Diet low in vegetables	People's Republic of Bangladesh	5.25 (3.59 to 7.20)
Diet low in vegetables	Tuvalu	5.24 (3.58 to 7.29)
Diet low in vegetables	Republic of Panama	5.23 (3.57 to 7.36)
Diet low in vegetables	Republic of Namibia	5.23 (3.54 to 7.28)
Diet low in vegetables	Republic of Seychelles	5.23 (3.55 to 7.27)
Diet low in vegetables	Islamic Republic of Pakistan	5.22 (3.56 to 7.24)
Diet low in vegetables	Republic of Niue	5.19 (3.55 to 7.14)
Diet low in vegetables	Republic of Nauru	5.19 (3.47 to 7.32)
Diet low in vegetables	Mongolia	5.18 (3.45 to 7.04)
Diet low in vegetables	Puerto Rico	5.16 (3.48 to 7.14)

Diet low in vegetables	Republic of South Sudan	5.14 (3.53 to 7.17)
Diet low in vegetables	Republic of Rwanda	5.10 (3.50 to 7.06)
Diet low in vegetables	Republic of Costa Rica	5.09 (3.47 to 7.07)
Diet low in vegetables	American Samoa	5.09 (3.42 to 7.27)
Diet low in vegetables	Tokelau	5.09 (3.35 to 7.10)
Diet low in vegetables	Grenada	5.05 (3.42 to 7.05)
Diet low in vegetables	Republic of Fiji	5.05 (3.36 to 7.27)
Diet low in vegetables	Republic of South Africa	5.05 (3.41 to 7.01)
Diet low in vegetables	Republic of Zambia	5.04 (3.46 to 7.08)
Diet low in vegetables	Solomon Islands	5.03 (3.40 to 7.08)
Diet low in vegetables	Republic of Ghana	5.01 (3.40 to 6.98)
Diet low in vegetables	Republic of Palau	5.00 (3.31 to 6.93)
Diet low in vegetables	Malaysia	4.98 (3.34 to 6.92)
Diet low in vegetables	United Republic of Tanzania	4.93 (3.31 to 6.77)
Diet low in vegetables	Saint Lucia	4.92 (3.35 to 6.89)
Diet low in vegetables	Republic of Djibouti	4.92 (3.38 to 6.92)
Diet low in vegetables	Republic of Angola	4.92 (3.25 to 6.88)
Diet low in vegetables	Republic of the Marshall Islands	4.89 (3.27 to 6.87)
Diet low in vegetables	Republic of Guinea	4.88 (3.26 to 6.79)
Diet low in vegetables	Republic of Paraguay	4.87 (3.32 to 6.91)
Diet low in vegetables	Kingdom of Cambodia	4.87 (3.28 to 6.89)
Diet low in vegetables	Republic of Kenya	4.81 (3.29 to 6.71)
Diet low in vegetables	Republic of Yemen	4.80 (3.27 to 6.56)
Diet low in vegetables	Bolivarian Republic of Venezuela	4.76 (3.16 to 6.61)
Diet low in vegetables	Republic of Colombia	4.76 (3.28 to 6.63)
Diet low in vegetables	Northern Mariana Islands	4.72 (3.16 to 6.81)
Diet low in vegetables	Federal Democratic Republic of Ethiopia	4.71 (3.19 to 6.53)
Diet low in vegetables	Democratic Socialist Republic of Sri Lanka	4.70 (3.16 to 6.50)
Diet low in vegetables	Cook Islands	4.69 (3.14 to 6.62)
Diet low in vegetables	Republic of Kiribati	4.68 (3.14 to 6.57)
Diet low in vegetables	Federated States of Micronesia	4.64 (3.18 to 6.56)
Diet low in vegetables	Belize	4.61 (3.10 to 6.45)
Diet low in vegetables	Republic of Benin	4.59 (3.10 to 6.57)
Diet low in vegetables	Republic of Honduras	4.57 (3.14 to 6.44)
Diet low in vegetables	Saint Vincent and the Grenadines	4.57 (3.03 to 6.43)
Diet low in vegetables	Federal Republic of Nigeria	4.56 (3.08 to 6.53)
Diet low in vegetables	Saint Kitts and Nevis	4.53 (2.97 to 6.29)
Diet low in vegetables	Republic of Ecuador	4.51 (3.08 to 6.19)
Diet low in vegetables	Guam	4.45 (2.99 to 6.16)
Diet low in vegetables	Republic of El Salvador	4.44 (3.03 to 6.20)
Diet low in vegetables	Republic of the Niger	4.43 (2.93 to 6.29)
Diet low in vegetables	Eastern Republic of Uruguay	4.41 (3.04 to 6.24)
Diet low in vegetables	Federative Republic of Brazil	4.38 (2.99 to 6.08)
Diet low in vegetables	Antigua and Barbuda	4.35 (2.96 to 6.10)

Diet low in vegetables	Plurinational State of Bolivia	4.33 (2.90 to 6.05)
Diet low in vegetables	Republic of Guyana	4.33 (2.90 to 6.13)
Diet low in vegetables	Barbados	4.32 (2.87 to 6.07)
Diet low in vegetables	Lao People's Democratic Republic	4.28 (2.80 to 6.06)
Diet low in vegetables	Kingdom of Bhutan	4.28 (2.82 to 6.05)
Diet low in vegetables	Republic of Guatemala	4.25 (2.90 to 5.87)
Diet low in vegetables	Republic of Peru	4.22 (2.87 to 5.91)
Diet low in vegetables	Republic of Suriname	4.20 (2.87 to 5.88)
Diet low in vegetables	Brunei Darussalam	4.18 (2.80 to 5.87)
Diet low in vegetables	Republic of Cameroon	4.17 (2.73 to 5.92)
Diet low in vegetables	Republic of the Union of Myanmar	4.16 (2.85 to 5.84)
Diet low in vegetables	Republic of Sudan	4.12 (2.73 to 5.89)
Diet low in vegetables	Kingdom of Norway	4.12 (2.76 to 5.84)
Diet low in vegetables	Kingdom of Thailand	4.11 (2.75 to 5.65)
Diet low in vegetables	Dominican Republic	4.04 (2.71 to 5.68)
Diet low in vegetables	Republic of Mauritius	4.02 (2.67 to 5.68)
Diet low in vegetables	Republic of India	4.00 (2.73 to 5.53)
Diet low in vegetables	Republic of the Philippines	3.90 (2.59 to 5.54)
Diet low in vegetables	Republic of Moldova	3.89 (2.54 to 5.59)
Diet low in vegetables	United Mexican States	3.89 (2.62 to 5.48)
Diet low in vegetables	Independent State of Papua New Guinea	3.88 (2.57 to 5.33)
Diet low in vegetables	Republic of Maldives	3.84 (2.56 to 5.45)
Diet low in vegetables	Jamaica	3.84 (2.56 to 5.33)
Diet low in vegetables	Republic of Iceland	3.84 (2.52 to 5.40)
Diet low in vegetables	Czech Republic	3.82 (2.51 to 5.59)
Diet low in vegetables	Republic of Finland	3.80 (2.47 to 5.46)
Diet low in vegetables	United States Virgin Islands	3.77 (2.51 to 5.28)
Diet low in vegetables	Kingdom of the Netherlands	3.73 (2.56 to 5.31)
Diet low in vegetables	Republic of Slovenia	3.73 (2.46 to 5.49)
Diet low in vegetables	Kingdom of Sweden	3.72 (2.40 to 5.37)
Diet low in vegetables	French Republic	3.68 (2.44 to 5.17)
Diet low in vegetables	Republic of Cyprus	3.65 (2.36 to 5.14)
Diet low in vegetables	Federal Republic of Germany	3.60 (2.40 to 5.19)
Diet low in vegetables	Commonwealth of Dominica	3.60 (2.40 to 5.11)
Diet low in vegetables	Republic of Cabo Verde	3.59 (2.34 to 5.33)
Diet low in vegetables	Federal Democratic Republic of Nepal	3.58 (2.39 to 4.96)
Diet low in vegetables	Argentine Republic	3.54 (2.36 to 5.09)
Diet low in vegetables	Republic of Croatia	3.53 (2.27 to 5.13)
Diet low in vegetables	Georgia	3.48 (2.20 to 5.07)
Diet low in vegetables	Socialist Republic of Viet Nam	3.41 (2.21 to 4.92)
Diet low in vegetables	Syrian Arab Republic	3.35 (2.21 to 4.87)
Diet low in vegetables	Commonwealth of the Bahamas	3.27 (2.15 to 4.65)
Diet low in vegetables	Swiss Confederation	3.23 (2.09 to 4.58)
Diet low in vegetables	Russian Federation	3.20 (2.10 to 4.57)

Diet low in vegetables	Grand Duchy of Luxembourg	3.17 (2.01 to 4.59)
Diet low in vegetables	Republic of Estonia	3.16 (1.99 to 4.70)
Diet low in vegetables	Bermuda	3.11 (2.04 to 4.48)
Diet low in vegetables	United Kingdom of Great Britain and Northern Ireland	3.10 (2.03 to 4.45)
Diet low in vegetables	Republic of Serbia	3.09 (1.97 to 4.51)
Diet low in vegetables	Slovak Republic	3.09 (2.01 to 4.66)
Diet low in vegetables	Republic of Austria	3.08 (2.00 to 4.39)
Diet low in vegetables	Republic of Latvia	3.05 (1.97 to 4.50)
Diet low in vegetables	Australia	3.01 (1.95 to 4.30)
Diet low in vegetables	Republic of Chile	3.00 (1.90 to 4.42)
Diet low in vegetables	Kingdom of Denmark	2.98 (1.85 to 4.58)
Diet low in vegetables	Republic of Lithuania	2.98 (1.88 to 4.47)
Diet low in vegetables	Kingdom of Saudi Arabia	2.95 (1.92 to 4.31)
Diet low in vegetables	New Zealand	2.93 (1.90 to 4.18)
Diet low in vegetables	State of Libya	2.85 (1.84 to 4.22)
Diet low in vegetables	Ireland	2.84 (1.81 to 4.15)
Diet low in vegetables	People's Democratic Republic of Algeria	2.80 (1.80 to 4.04)
Diet low in vegetables	Democratic People's Republic of Korea	2.78 (1.79 to 4.00)
Diet low in vegetables	United States of America	2.78 (1.83 to 3.97)
Diet low in vegetables	Palestine	2.76 (1.80 to 4.01)
Diet low in vegetables	Republic of San Marino	2.71 (1.64 to 4.13)
Diet low in vegetables	Hashemite Kingdom of Jordan	2.69 (1.69 to 3.91)
Diet low in vegetables	Canada	2.65 (1.68 to 3.82)
Diet low in vegetables	United Arab Emirates	2.58 (1.59 to 3.88)
Diet low in vegetables	Principality of Andorra	2.49 (1.49 to 3.77)
Diet low in vegetables	Hungary	2.47 (1.46 to 3.96)
Diet low in vegetables	Sultanate of Oman	2.46 (1.50 to 3.67)
Diet low in vegetables	Republic of Cuba	2.43 (1.56 to 3.52)
Diet low in vegetables	Kingdom of Belgium	2.24 (1.35 to 3.41)
Diet low in vegetables	Kingdom of Spain	2.23 (1.34 to 3.37)
Diet low in vegetables	Republic of Italy	2.15 (1.35 to 3.19)
Diet low in vegetables	Kingdom of Morocco	2.04 (1.17 to 3.19)
Diet low in vegetables	Greenland	1.98 (1.12 to 3.16)
Diet low in vegetables	Taiwan (Province of China)	1.73 (0.99 to 2.70)
Diet low in vegetables	Bosnia and Herzegovina	1.73 (0.92 to 2.81)
Diet low in vegetables	Republic of Iraq	1.71 (0.91 to 2.73)
Diet low in vegetables	Republic of Korea	1.71 (1.03 to 2.66)
Diet low in vegetables	Republic of Singapore	1.64 (0.96 to 2.51)
Diet low in vegetables	Ukraine	1.54 (0.80 to 2.61)
Diet low in vegetables	Republic of Malta	1.51 (0.77 to 2.52)
Diet low in vegetables	Republic of Tajikistan	1.48 (0.76 to 2.48)
Diet low in vegetables	Republic of Belarus	1.46 (0.75 to 2.52)
Diet low in vegetables	Portuguese Republic	1.36 (0.71 to 2.25)
Diet low in vegetables	Kyrgyz Republic	1.30 (0.65 to 2.20)

Diet low in vegetables	Japan	1.29 (0.72 to 2.04)
Diet low in vegetables	Kingdom of Bahrain	1.26 (0.61 to 2.14)
Diet low in vegetables	Republic of Poland	1.25 (0.66 to 2.10)
Diet low in vegetables	Republic of Tunisia	1.15 (0.56 to 2.01)
Diet low in vegetables	Republic of Kazakhstan	0.95 (0.41 to 1.79)
Diet low in vegetables	Islamic Republic of Iran	0.93 (0.49 to 1.56)
Diet low in vegetables	Republic of Bulgaria	0.93 (0.44 to 1.68)
Diet low in vegetables	State of Kuwait	0.84 (0.33 to 1.53)
Diet low in vegetables	North Macedonia	0.68 (0.25 to 1.33)
Diet low in vegetables	Hellenic Republic	0.59 (0.22 to 1.20)
Diet low in vegetables	Principality of Monaco	0.56 (0.16 to 1.17)
Diet low in vegetables	Republic of Azerbaijan	0.53 (0.17 to 1.15)
Diet low in vegetables	Republic of Albania	0.53 (0.15 to 1.11)
Diet low in vegetables	Montenegro	0.49 (0.18 to 1.03)
Diet low in vegetables	Turkmenistan	0.41 (0.12 to 0.84)
Diet low in vegetables	State of Israel	0.40 (0.12 to 0.88)
Diet low in vegetables	People's Republic of China	0.33 (0.14 to 0.63)
Diet low in vegetables	Lebanese Republic	0.29 (0.07 to 0.65)
Diet low in vegetables	State of Qatar	0.23 (0.04 to 0.59)
Diet low in vegetables	Republic of Uzbekistan	0.15 (0.03 to 0.39)
Diet low in vegetables	Romania	0.15 (0.03 to 0.38)
Diet low in vegetables	Arab Republic of Egypt	0.11 (0.01 to 0,29)
Diet low in vegetables	Republic of Armenia	0.10 (0.01 to 0.28)
Diet low in vegetables	Republic of Turkey	0.04 (0.00 to 0.15)

High body-mass index

Risk factor	Location	Percent contribution to ASDR (%)
High body-mass index	State of Qatar	14.28 (7.71 to 24.22)
High body-mass index	State of Kuwait	14.20 (7.53 to 23.42)
High body-mass index	Hungary	14.15 (7.62 to 23.35)
High body-mass index	Kingdom of Saudi Arabia	13.72 (7.21 to 23.44)
High body-mass index	Republic of Moldova	13.72 (7.39 to 22.85)
High body-mass index	United Arab Emirates	13.33 (7.08 to 22.36)
High body-mass index	State of Libya	13.25 (6.98 to 22.52)
High body-mass index	Syrian Arab Republic	13.15 (6.90 to 22.56)
High body-mass index	Montenegro	13.07 (6.80 to 22.00)
High body-mass index	Hashemite Kingdom of Jordan	12.95 (7.01 to 22.36)
High body-mass index	American Samoa	12.94 (7.04 to 21.96)
High body-mass index	Kingdom of Tonga	12.88 (7.00 to 21.35)
High body-mass index	Arab Republic of Egypt	12.86 (6.91 to 21.67)
High body-mass index	Republic of Iraq	12.73 (6.71 to 21.88)
High body-mass index	Republic of Lithuania	12.45 (6.49 to 21.18)
High body-mass index	Sultanate of Oman	12.43 (6.72 to 20.96)

High body-mass index	Republic of Chile	12.35 (6.40 to 21.43)
High body-mass index	Slovak Republic	12.25 (6.41 to 20.92)
High body-mass index	Republic of Fiji	12.20 (6.59 to 20.53)
High body-mass index	Kingdom of Eswatini	12.17 (6.58 to 21.19)
High body-mass index	Republic of Panama	12.06 (6.33 to 20.76)
High body-mass index	Republic of Slovenia	12.05 (6.21 to 20.81)
High body-mass index	Cook Islands	12.00 (6.26 to 19.84)
High body-mass index	Republic of Latvia	12.00 (6.31 to 20.48)
High body-mass index	Republic of Belarus	11.94 (6.28 to 20.70)
High body-mass index	Kingdom of Bahrain	11.85 (6.22 to 20.19)
High body-mass index	Republic of Croatia	11.84 (6.23 to 20.14)
High body-mass index	Republic of Nauru	11.76 (5.72 to 21.32)
High body-mass index	Northern Mariana Islands	11.74 (6.39 to 19.70)
High body-mass index	Romania	11.74 (6.20 to 20.07)
High body-mass index	Republic of Serbia	11.63 (6.23 to 19.83)
High body-mass index	Republic of Estonia	11.60 (6.04 to 19.86)
High body-mass index	Ukraine	11.59 (6.23 to 19.70)
High body-mass index	Czech Republic	11.54 (5.91 to 19.78)
High body-mass index	Lebanese Republic	11.42 (5.93 to 19.79)
High body-mass index	Republic of Palau	11.39 (6.08 to 19.59)
High body-mass index	Russian Federation	11.24 (5.88 to 19.46)
High body-mass index	Independent State of Samoa	11.23 (5.98 to 19.78)
High body-mass index	Republic of Turkey	11.22 (6.02 to 18.97)
High body-mass index	Republic of Bulgaria	11.19 (5.91 to 18.98)
High body-mass index	Republic of Niue	11.15 (5.82 to 19.33)
High body-mass index	Bolivarian Republic of Venezuela	11.13 (5.70 to 18.95)
High body-mass index	Republic of South Africa	11.12 (5.89 to 19.05)
High body-mass index	Commonwealth of Dominica	11.04 (5.78 to 18.89)
High body-mass index	Palestine	10.98 (5.72 to 19.00)
High body-mass index	Republic of Kazakhstan	10.95 (5.96 to 18.23)
High body-mass index	Puerto Rico	10.79 (5.74 to 17.93)
High body-mass index	United States of America	10.76 (5.80 to 18.29)
High body-mass index	Guam	10.63 (5.71 to 17.86)
High body-mass index	North Macedonia	10.62 (5.52 to 18.11)
High body-mass index	Republic of Albania	10.58 (5.46 to 17.97)
High body-mass index	Republic of El Salvador	10.58 (5.56 to 18.38)
High body-mass index	Republic of Azerbaijan	10.43 (5.69 to 17.80)
High body-mass index	Bermuda	10.41 (5.40 to 17.66)
High body-mass index	Republic of Nicaragua	10.34 (5.43 to 17.78)
High body-mass index	Argentine Republic	10.33 (5.36 to 17.35)
High body-mass index	Tokelau	10.32 (5.09 to 18.14)
High body-mass index	United Mexican States	10.18 (5.37 to 17.34)
High body-mass index	Republic of Sudan	10.14 (5.55 to 16.88)
High body-mass index	Georgia	10.10 (5.46 to 17.41)

High body-mass index	Bosnia and Herzegovina	10.10 (5.33 to 17.16)
High body-mass index	Belize	10.01 (5.20 to 17.23)
High body-mass index	Republic of Costa Rica	9.99 (5.28 to 17.64)
High body-mass index	United States Virgin Islands	9.96 (5.20 to 17.31)
High body-mass index	Tuvalu	9.96 (5.25 to 17.15)
High body-mass index	Republic of Cameroon	9.95 (5.34 to 16.87)
High body-mass index	Republic of Trinidad and Tobago	9.90 (5.13 to 16.80)
High body-mass index	Kingdom of Spain	9.85 (5.20 to 17.40)
High body-mass index	Barbados	9.81 (5.23 to 17.26)
High body-mass index	Federal Republic of Germany	9.80 (5.14 to 16.83)
High body-mass index	Commonwealth of the Bahamas	9.72 (5.17 to 16.70)
High body-mass index	Republic of the Marshall Islands	9.67 (5.02 to 16.48)
High body-mass index	Republic of Armenia	9.67 (5.25 to 16.37)
High body-mass index	Republic of Colombia	9.64 (5.16 to 16.30)
High body-mass index	Kyrgyz Republic	9.64 (5.10 to 16.77)
High body-mass index	Eastern Republic of Uruguay	9.59 (5.08 to 16.46)
High body-mass index	Republic of Paraguay	9.55 (4.99 to 17.17)
High body-mass index	Republic of Poland	9.53 (5.00 to 16.69)
High body-mass index	Principality of Monaco	9.51 (4.95 to 16.55)
High body-mass index	Islamic Republic of Mauritania	9.51 (4.92 to 16.44)
High body-mass index	Australia	9.46 (5.00 to 16.27)
High body-mass index	Turkmenistan	9.43 (4.93 to 16.15)
High body-mass index	Federative Republic of Brazil	9.41 (4.95 to 16.40)
High body-mass index	Republic of Finland	9.35 (4.91 to 16.57)
High body-mass index	Grand Duchy of Luxembourg	9.30 (4.83 to 15.99)
High body-mass index	Republic of San Marino	9.22 (4.75 to 16.41)
High body-mass index	New Zealand	9.20 (4.73 to 15.82)
High body-mass index	Islamic Republic of Iran	9.19 (4.95 to 15.72)
High body-mass index	Republic of Uzbekistan	9.14 (4.92 to 16.03)
High body-mass index	Republic of Kiribati	9.04 (4.79 to 15.54)
High body-mass index	Federated States of Micronesia	9.02 (4.77 to 16.01)
High body-mass index	Republic of Iceland	9.01 (4.60 to 15.72)
High body-mass index	United Kingdom of Great Britain and Northern Ireland	8.97 (4.68 to 15.61)
High body-mass index	Ireland	8.95 (4.58 to 16.00)
High body-mass index	People's Democratic Republic of Algeria	8.89 (4.59 to 15.32)
High body-mass index	State of Israel	8.89 (4.67 to 15.60)
High body-mass index	Saint Kitts and Nevis	8.83 (4.53 to 15.88)
High body-mass index	Gabonese Republic	8.77 (4.48 to 15.56)
High body-mass index	Hellenic Republic	8.73 (4.60 to 15.34)
High body-mass index	Kingdom of Morocco	8.73 (4.74 to 14.88)
High body-mass index	Canada	8.71 (4.64 to 15.53)
High body-mass index	Republic of Austria	8.66 (4.58 to 15.41)
High body-mass index	Republic of Guatemala	8.64 (4.58 to 14.91)
High body-mass index	Greenland	8.53 (4.38 to 15.00)

High body-mass index	Republic of Ecuador	8.53 (4.55 to 14.81)
High body-mass index	Jamaica	8.52 (4.48 to 14.61)
High body-mass index	Mongolia	8.51 (4.49 to 14.40)
High body-mass index	Republic of Tunisia	8.47 (4.51 to 15.31)
High body-mass index	Republic of Tajikistan	8.34 (4.50 to 14.12)
High body-mass index	French Republic	8.31 (4.31 to 15.08)
High body-mass index	Republic of Honduras	8.26 (4.43 to 14.28)
High body-mass index	Kingdom of Belgium	8.24 (4.42 to 14.35)
High body-mass index	Portuguese Republic	8.19 (4.23 to 14.33)
High body-mass index	Republic of Cyprus	8.16 (4.32 to 14.18)
High body-mass index	Federal Republic of Nigeria	8.10 (4.46 to 13.63)
High body-mass index	Republic of Malta	8.08 (4.22 to 14.67)
High body-mass index	Republic of Equatorial Guinea	8.06 (4.44 to 13.67)
High body-mass index	Antigua and Barbuda	8.06 (4.26 to 13.74)
High body-mass index	Republic of Namibia	8.05 (4.15 to 14.13)
High body-mass index	Kingdom of Sweden	8.04 (4.20 to 13.68)
High body-mass index	Republic of Vanuatu	8.02 (4.22 to 14.04)
High body-mass index	Republic of Liberia	7.98 (4.08 to 13.94)
High body-mass index	Kingdom of Lesotho	7.98 (4.14 to 13.83)
High body-mass index	Republic of Botswana	7.95 (4.08 to 14.16)
High body-mass index	Kingdom of Denmark	7.95 (4.18 to 13.95)
High body-mass index	Democratic Republic of Sao Tome and Principe	7.94 (4.15 to 13.82)
High body-mass index	Principality of Andorra	7.91 (4.22 to 13.69)
High body-mass index	Republic of Seychelles	7.86 (4.25 to 13.13)
High body-mass index	Kingdom of the Netherlands	7.82 (3.95 to 13.19)
High body-mass index	Republic of Italy	7.73 (4.04 to 13.63)
High body-mass index	Republic of Guyana	7.63 (3.96 to 12.89)
High body-mass index	Kingdom of Norway	7.61 (4.09 to 13.56)
High body-mass index	Republic of Peru	7.50 (4.04 to 12.90)
High body-mass index	Grenada	7.26 (3.80 to 12.34)
High body-mass index	Republic of Zimbabwe	7.16 (3.79 to 12.18)
High body-mass index	Saint Lucia	7.07 (3.81 to 12.09)
High body-mass index	Dominican Republic	6.99 (3.83 to 11.89)
High body-mass index	Swiss Confederation	6.82 (3.67 to 12.12)
High body-mass index	Republic of Cabo Verde	6.79 (3.57 to 11.76)
High body-mass index	Plurinational State of Bolivia	6.67 (3.52 to 11.14)
High body-mass index	Republic of Cuba	6.57 (3.50 to 11.48)
High body-mass index	Islamic Republic of Afghanistan	6.52 (3.52 to 11.49)
High body-mass index	Republic of Côte d'Ivoire	6.42 (3.39 to 11.42)
High body-mass index	Republic of Yemen	6.41 (3.52 to 10.90)
High body-mass index	Republic of Mauritius	6.33 (3.40 to 11.36)
High body-mass index	Republic of Ghana	6.29 (3.29 to 10.82)
High body-mass index	Togolese Republic	6.17 (3.29 to 11.15)
High body-mass index	Republic of the Gambia	6.10 (3.13 to 10.80)

High body-mass index	Taiwan (Province of China)	6.07 (3.29 to 10.44)
High body-mass index	Malaysia	5.97 (3.23 to 9.99)
High body-mass index	Saint Vincent and the Grenadines	5.96 (3.20 to 10.44)
High body-mass index	Republic of Senegal	5.88 (3.17 to 9.74)
High body-mass index	Republic of the Congo	5.86 (3.13 to 9.83)
High body-mass index	Republic of Suriname	5.80 (3.12 to 9.86)
High body-mass index	People's Republic of China	5.71 (3.12 to 9.70)
High body-mass index	Republic of Sierra Leone	5.57 (2.92 to 9.66)
High body-mass index	Solomon Islands	5.54 (2.90 to 9.70)
High body-mass index	Union of the Comoros	5.51 (3.02 to 9.56)
High body-mass index	Republic of Benin	5.50 (2.86 to 9.79)
High body-mass index	United Republic of Tanzania	5.28 (2.76 to 9.19)
High body-mass index	Republic of Kenya	5.19 (2.82 to 8.93)
High body-mass index	Islamic Republic of Pakistan	5.14 (2.81 to 8.56)
High body-mass index	Republic of Guinea-Bissau	5.06 (2.73 to 8.50)
High body-mass index	Democratic Republic of the Congo	5.05 (2.74 to 8.39)
High body-mass index	Kingdom of Bhutan	4.99 (2.73 to 8.36)
High body-mass index	Brunei Darussalam	4.85 (2.72 to 7.95)
High body-mass index	Republic of Malawi	4.82 (2.59 to 8.10)
High body-mass index	Republic of Singapore	4.61 (2.43 to 7.86)
High body-mass index	Republic of Zambia	4.58 (2.47 to 7.66)
High body-mass index	Japan	4.57 (2.49 to 7.25)
High body-mass index	Republic of Angola	4.41 (2.45 to 7.45)
High body-mass index	Republic of Guinea	4.23 (2.32 to 7.41)
High body-mass index	Kingdom of Thailand	4.06 (2.16 to 6.60)
High body-mass index	Republic of Maldives	4.06 (2.12 to 6.83)
High body-mass index	Republic of Mozambique	4.05 (2.20 to 6.86)
High body-mass index	Democratic Socialist Republic of Sri Lanka	4.04 (2.23 to 6.84)
High body-mass index	Independent State of Papua New Guinea	4.02 (2.10 to 6.92)
High body-mass index	Republic of Mali	3.95 (1.92 to 7.34)
High body-mass index	Republic of the Philippines	3.89 (2.14 to 6.34)
High body-mass index	Democratic People's Republic of Korea	3.86 (2.00 to 6.67)
High body-mass index	Republic of the Niger	3.83 (1.79 to 7.39)
High body-mass index	Republic of Indonesia	3.78 (2.07 to 5.77)
High body-mass index	Republic of Korea	3.78 (2.01 to 6.14)
High body-mass index	Central African Republic	3.76 (2.09 to 6.39)
High body-mass index	Republic of Haiti	3.70 (2.06 to 6.23)
High body-mass index	Republic of Chad	3.68 (1.90 to 6.60)
High body-mass index	Republic of Uganda	3.51 (1.88 to 5.72)
High body-mass index	Republic of Madagascar	3.40 (1.80 to 5.77)
High body-mass index	Federal Republic of Somalia	3.31 (1.70 to 5.52)
High body-mass index	Republic of Rwanda	3.27 (1.77 to 5.42)
High body-mass index	Republic of India	3.14 (1.79 to 5.09)
High body-mass index	Lao People's Democratic Republic	2.89 (1.55 to 4.78)

High body-mass index	Republic of Djibouti	2.86 (1.48 to 4.61)
High body-mass index	Federal Democratic Republic of Ethiopia	2.75 (1.49 to 4.57)
High body-mass index	Burkina Faso	2.67 (1.43 to 4.40)
High body-mass index	Republic of the Union of Myanmar	2.64 (1.42 to 4.18)
High body-mass index	Republic of Burundi	2.51 (1.34 to 4.40)
High body-mass index	Republic of South Sudan	2.36 (1.28 to 3.97)
High body-mass index	People's Republic of Bangladesh	2.33 (1.26 to 3.81)
High body-mass index	State of Eritrea	2.26 (1.20 to 3.89)
High body-mass index	Socialist Republic of Viet Nam	2.17 (1.17 to 3.49)
High body-mass index	Federal Democratic Republic of Nepal	2.15 (1.17 to 3.43)
High body-mass index	Kingdom of Cambodia	1.97 (1.06 to 3.12)
High body-mass index	Democratic Republic of Timor-Leste	1.76 (0.94 to 2.86)

High systolic blood pressure

Risk factor	Location	Percent contribution to ASDR (%)
High systolic blood pressure	Republic of Indonesia	26.26 (20.01 to 32.45)
High systolic blood pressure	Hungary	25.90 (19.96 to 32.82)
High systolic blood pressure	Republic of Moldova	24.72 (18.33 to 31.43)
High systolic blood pressure	Republic of Sierra Leone	24.41 (18.01 to 31.39)
High systolic blood pressure	Republic of Kazakhstan	23.87 (18.45 to 29.84)
High systolic blood pressure	Republic of Lithuania	23.43 (17.71 to 29.46)
High systolic blood pressure	Malaysia	23.41 (17.36 to 29.76)
High systolic blood pressure	Georgia	22.78 (16.54 to 29.17)
High systolic blood pressure	Republic of Latvia	22.67 (16.90 to 28.72)
High systolic blood pressure	Republic of Belarus	22.62 (16.97 to 28.69)
High systolic blood pressure	Romania	22.48 (16.97 to 28.18)
High systolic blood pressure	Republic of Vanuatu	22.43 (16.58 to 29.13)
High systolic blood pressure	Republic of Serbia	22.36 (16.79 to 28.23)
High systolic blood pressure	Democratic Republic of Sao Tome and Principe	22.32 (16.91 to 28.30)
High systolic blood pressure	Republic of Estonia	22.31 (16.34 to 28.21)
High systolic blood pressure	Federal Republic of Nigeria	22.30 (16.50 to 28.23)
High systolic blood pressure	Republic of Zimbabwe	22.19 (16.49 to 28.41)
High systolic blood pressure	Togolese Republic	22.18 (16.73 to 28.22)
High systolic blood pressure	Republic of Cameroon	22.07 (16.27 to 28.19)
High systolic blood pressure	Republic of Senegal	21.97 (16.28 to 27.95)
High systolic blood pressure	Republic of Croatia	21.70 (15.88 to 27.85)
High systolic blood pressure	Ukraine	21.67 (16.09 to 27.90)
High systolic blood pressure	Republic of Cabo Verde	21.62 (15.78 to 27.44)
High systolic blood pressure	Republic of Iraq	21.56 (16.38 to 27.11)
High systolic blood pressure	Republic of Slovenia	21.50 (15.71 to 27.83)
High systolic blood pressure	Republic of Albania	21.49 (15.99 to 27.03)
High systolic blood pressure	Montenegro	21.40 (15.87 to 27.79)

High systolic blood pressure	Slovak Republic	21.39 (15.92 to 27.40)
High systolic blood pressure	Republic of Chile	21.33 (15.99 to 26.86)
High systolic blood pressure	Bosnia and Herzegovina	21.30 (15.35 to 27.55)
High systolic blood pressure	Republic of Trinidad and Tobago	21.25 (15.60 to 26.90)
High systolic blood pressure	Socialist Republic of Viet Nam	21.18 (15.42 to 26.97)
High systolic blood pressure	Republic of the Gambia	21.10 (15.24 to 27.34)
High systolic blood pressure	Mongolia	20.98 (14.87 to 26.68)
High systolic blood pressure	Republic of Bulgaria	20.96 (15.92 to 27.28)
High systolic blood pressure	North Macedonia	20.94 (15.26 to 27.24)
High systolic blood pressure	Republic of Côte d'Ivoire	20.92 (15.49 to 27.13)
High systolic blood pressure	Republic of South Africa	20.84 (15.49 to 26.64)
High systolic blood pressure	Turkmenistan	20.74 (15.50 to 26.44)
High systolic blood pressure	Republic of Liberia	20.71 (15.27 to 26.56)
High systolic blood pressure	Republic of Sudan	20.61 (15.54 to 26.05)
High systolic blood pressure	Republic of Colombia	20.60 (15.35 to 26.41)
High systolic blood pressure	Islamic Republic of Mauritania	20.47 (14.14 to 26.66)
High systolic blood pressure	Republic of Equatorial Guinea	20.47 (14.73 to 26.86)
High systolic blood pressure	Republic of Ghana	20.27 (14.75 to 25.81)
High systolic blood pressure	Russian Federation	20.24 (15.31 to 25.31)
High systolic blood pressure	Republic of Azerbaijan	20.18 (14.62 to 25.94)
High systolic blood pressure	Republic of Malawi	20.07 (14.47 to 26.24)
High systolic blood pressure	Federal Republic of Germany	20.02 (14.35 to 25.45)
High systolic blood pressure	Republic of Mozambique	20.00 (14.57 to 25.69)
High systolic blood pressure	Kingdom of Morocco	19.96 (15.00 to 25.61)
High systolic blood pressure	Kingdom of Denmark	19.95 (14.44 to 26.56)
High systolic blood pressure	Republic of Botswana	19.92 (14.07 to 26.05)
High systolic blood pressure	Republic of the Congo	19.92 (14.07 to 25.84)
High systolic blood pressure	Republic of Armenia	19.85 (14.77 to 25.85)
High systolic blood pressure	Bolivarian Republic of Venezuela	19.83 (14.20 to 25.93)
High systolic blood pressure	Czech Republic	19.73 (14.72 to 25.99)
High systolic blood pressure	Grand Duchy of Luxembourg	19.72 (13.84 to 25.13)
High systolic blood pressure	Republic of Costa Rica	19.71 (14.87 to 25.12)
High systolic blood pressure	Republic of Finland	19.70 (14.27 to 25.37)
High systolic blood pressure	Kingdom of Eswatini	19.64 (14.12 to 25.82)
High systolic blood pressure	Gabonese Republic	19.59 (14.09 to 25.41)
High systolic blood pressure	Republic of Guinea-Bissau	19.56 (14.15 to 25.18)
High systolic blood pressure	State of Libya	19.41 (14.32 to 24.73)
High systolic blood pressure	State of Israel	19.36 (14.40 to 24.96)
High systolic blood pressure	Kingdom of Norway	19.35 (14.09 to 25.25)
High systolic blood pressure	Republic of Benin	19.34 (13.85 to 25.46)
High systolic blood pressure	Republic of Seychelles	19.30 (13.81 to 25.75)
High systolic blood pressure	Republic of the Niger	19.29 (13.60 to 26.78)
High systolic blood pressure	Republic of Kenya	19.28 (14.60 to 24.54)
High systolic blood pressure	Kingdom of Belgium	19.25 (14.36 to 24.31)

High systolic blood pressure	Republic of Fiji	19.22 (13.87 to 25.08)
High systolic blood pressure	Republic of Panama	19.20 (13.90 to 24.54)
High systolic blood pressure	Republic of Angola	19.19 (14.08 to 25.33)
High systolic blood pressure	Republic of Djibouti	18.98 (13.99 to 24.26)
High systolic blood pressure	Republic of Mali	18.94 (13.36 to 25.17)
High systolic blood pressure	Barbados	18.92 (13.99 to 24.56)
High systolic blood pressure	Republic of Mauritius	18.88 (13.64 to 24.69)
High systolic blood pressure	State of Qatar	18.87 (13.91 to 24.35)
High systolic blood pressure	Republic of Austria	18.78 (13.57 to 24.44)
High systolic blood pressure	Republic of Nicaragua	18.77 (13.55 to 24.28)
High systolic blood pressure	Republic of Niue	18.77 (13.88 to 23.89)
High systolic blood pressure	Democratic Republic of Timor-Leste	18.72 (13.59 to 24.15)
High systolic blood pressure	Republic of Tajikistan	18.68 (13.42 to 24.43)
High systolic blood pressure	Sultanate of Oman	18.68 (13.75 to 24.34)
High systolic blood pressure	Ireland	18.68 (13.59 to 24.65)
High systolic blood pressure	Democratic Socialist Republic of Sri Lanka	18.63 (13.51 to 24.17)
High systolic blood pressure	Kingdom of the Netherlands	18.61 (13.56 to 25.11)
High systolic blood pressure	Republic of Cyprus	18.59 (13.49 to 23.81)
High systolic blood pressure	Principality of Andorra	18.54 (13.02 to 24.44)
High systolic blood pressure	American Samoa	18.48 (12.87 to 23.90)
High systolic blood pressure	Republic of Maldives	18.47 (13.34 to 23.99)
High systolic blood pressure	Republic of Haiti	18.45 (13.26 to 24.04)
High systolic blood pressure	Kingdom of Sweden	18.40 (13.09 to 23.89)
High systolic blood pressure	Union of the Comoros	18.39 (13.58 to 23.78)
High systolic blood pressure	French Republic	18.36 (13.50 to 23.63)
High systolic blood pressure	Republic of San Marino	18.35 (12.94 to 24.16)
High systolic blood pressure	Republic of Nauru	18.27 (12.30 to 24.81)
High systolic blood pressure	Central African Republic	18.22 (12.64 to 24.38)
High systolic blood pressure	Islamic Republic of Pakistan	18.22 (13.20 to 23.52)
High systolic blood pressure	Republic of the Union of Myanmar	18.21 (13.23 to 24.03)
High systolic blood pressure	Republic of Burundi	18.10 (12.36 to 25.21)
High systolic blood pressure	Burkina Faso	18.09 (12.69 to 24.17)
High systolic blood pressure	Principality of Monaco	18.07 (13.29 to 23.07)
High systolic blood pressure	Republic of Namibia	18.00 (13.02 to 23.58)
High systolic blood pressure	Republic of Malta	17.99 (12.82 to 24.18)
High systolic blood pressure	Kingdom of Tonga	17.80 (12.46 to 23.83)
High systolic blood pressure	Japan	17.64 (13.11 to 22.97)
High systolic blood pressure	Northern Mariana Islands	17.63 (12.50 to 23.72)
High systolic blood pressure	Saint Kitts and Nevis	17.63 (12.46 to 23.00)
High systolic blood pressure	Republic of El Salvador	17.63 (12.60 to 23.05)
High systolic blood pressure	Kyrgyz Republic	17.57 (12.33 to 22.95)
High systolic blood pressure	Kingdom of Bahrain	17.51 (11.74 to 23.00)
High systolic blood pressure	Cook Islands	17.51 (12.35 to 23.27)
High systolic blood pressure	Republic of Paraguay	17.48 (12.01 to 22.82)

High systolic blood pressure	Puerto Rico	17.46 (12.50 to 23.34)
High systolic blood pressure	Republic of Uganda	17.44 (12.69 to 22.43)
High systolic blood pressure	Federative Republic of Brazil	17.43 (13.06 to 22.13)
High systolic blood pressure	Republic of Poland	17.39 (12.66 to 22.11)
High systolic blood pressure	Republic of Honduras	17.37 (12.55 to 22.59)
High systolic blood pressure	Republic of Uzbekistan	17.30 (12.41 to 22.22)
High systolic blood pressure	Republic of Iceland	17.27 (12.28 to 22.76)
High systolic blood pressure	Republic of Palau	17.25 (12.07 to 23.73)
High systolic blood pressure	Guam	17.22 (12.13 to 23.27)
High systolic blood pressure	Argentine Republic	17.19 (12.66 to 22.60)
High systolic blood pressure	Kingdom of Spain	17.13 (12.09 to 22.29)
High systolic blood pressure	Republic of Madagascar	17.12 (11.83 to 22.59)
High systolic blood pressure	Lebanese Republic	17.11 (12.49 to 21.80)
High systolic blood pressure	Kingdom of Lesotho	17.10 (11.38 to 23.68)
High systolic blood pressure	United Arab Emirates	17.09 (12.14 to 22.16)
High systolic blood pressure	Lao People's Democratic Republic	17.05 (12.17 to 22.95)
High systolic blood pressure	Eastern Republic of Uruguay	17.05 (12.37 to 22.14)
High systolic blood pressure	Grenada	16.86 (11.92 to 22.35)
High systolic blood pressure	Commonwealth of the Bahamas	16.86 (12.08 to 22.71)
High systolic blood pressure	Syrian Arab Republic	16.83 (11.97 to 22.14)
High systolic blood pressure	Republic of South Sudan	16.83 (11.77 to 22.74)
High systolic blood pressure	Tokelau	16.82 (11.27 to 23.08)
High systolic blood pressure	People's Republic of Bangladesh	16.81 (12.07 to 22.06)
High systolic blood pressure	United Mexican States	16.80 (12.15 to 21.47)
High systolic blood pressure	State of Eritrea	16.74 (11.48 to 22.60)
High systolic blood pressure	Republic of Guinea	16.74 (11.95 to 21.80)
High systolic blood pressure	Saint Lucia	16.71 (11.67 to 22.04)
High systolic blood pressure	Democratic Republic of the Congo	16.70 (11.84 to 21.61)
High systolic blood pressure	Antigua and Barbuda	16.69 (11.81 to 21.55)
High systolic blood pressure	Arab Republic of Egypt	16.67 (11.94 to 21.33)
High systolic blood pressure	Federal Republic of Somalia	16.67 (10.87 to 23.72)
High systolic blood pressure	Republic of Chad	16.64 (11.78 to 22.35)
High systolic blood pressure	Commonwealth of Dominica	16.61 (11.60 to 22.07)
High systolic blood pressure	Republic of Guyana	16.59 (12.06 to 21.89)
High systolic blood pressure	Tuvalu	16.43 (11.40 to 22.44)
High systolic blood pressure	Bermuda	16.42 (11.93 to 21.76)
High systolic blood pressure	State of Kuwait	16.39 (11.85 to 21.03)
High systolic blood pressure	United Republic of Tanzania	16.38 (11.53 to 21.30)
High systolic blood pressure	Hashemite Kingdom of Jordan	16.35 (11.72 to 21.27)
High systolic blood pressure	People's Democratic Republic of Algeria	16.31 (11.52 to 21.31)
High systolic blood pressure	Portuguese Republic	16.30 (11.83 to 21.11)
High systolic blood pressure	Jamaica	16.24 (11.61 to 21.27)
High systolic blood pressure	Kingdom of Saudi Arabia	16.22 (12.00 to 21.32)
High systolic blood pressure	Republic of Rwanda	16.15 (10.98 to 21.38)

High systolic blood pressure	Dominican Republic	16.09 (11.54 to 21.42)
High systolic blood pressure	Republic of India	16.08 (12.02 to 20.48)
High systolic blood pressure	Republic of Turkey	16.05 (11.87 to 20.54)
High systolic blood pressure	Belize	16.05 (11.35 to 20.91)
High systolic blood pressure	United States Virgin Islands	16.04 (11.47 to 21.61)
High systolic blood pressure	Independent State of Samoa	15.99 (11.19 to 21.21)
High systolic blood pressure	Brunei Darussalam	15.94 (11.28 to 20.95)
High systolic blood pressure	People's Republic of China	15.94 (11.35 to 20.62)
High systolic blood pressure	Republic of Guatemala	15.76 (11.34 to 20.42)
High systolic blood pressure	Kingdom of Bhutan	15.52 (10.91 to 21.01)
High systolic blood pressure	Saint Vincent and the Grenadines	15.46 (11.25 to 20.57)
High systolic blood pressure	New Zealand	15.41 (11.02 to 20.01)
High systolic blood pressure	Swiss Confederation	15.35 (10.66 to 20.59)
High systolic blood pressure	United Kingdom of Great Britain and Northern Ireland	15.27 (11.16 to 19.39)
High systolic blood pressure	Republic of Italy	15.18 (11.24 to 20.00)
High systolic blood pressure	Islamic Republic of Iran	15.07 (11.13 to 19.22)
High systolic blood pressure	Australia	14.93 (10.87 to 19.53)
High systolic blood pressure	Republic of Peru	14.86 (10.76 to 19.23)
High systolic blood pressure	Islamic Republic of Afghanistan	14.80 (10.28 to 20.45)
High systolic blood pressure	Republic of Yemen	14.64 (10.73 to 19.49)
High systolic blood pressure	Republic of the Philippines	14.49 (10.67 to 19.23)
High systolic blood pressure	Hellenic Republic	14.48 (10.49 to 18.92)
High systolic blood pressure	Palestine	14.47 (10.14 to 19.00)
High systolic blood pressure	Republic of Zambia	14.24 (9.88 to 18.77)
High systolic blood pressure	Canada	14.19 (10.08 to 19.22)
High systolic blood pressure	United States of America	14.19 (10.04 to 18.67)
High systolic blood pressure	Democratic People's Republic of Korea	14.16 (9.76 to 18.76)
High systolic blood pressure	Kingdom of Cambodia	14.14 (9.52 to 19.65)
High systolic blood pressure	Greenland	14.00 (9.31 to 19.21)
High systolic blood pressure	Taiwan (Province of China)	13.98 (9.72 to 18.84)
High systolic blood pressure	Republic of the Marshall Islands	13.96 (9.61 to 18.72)
High systolic blood pressure	Republic of Tunisia	13.84 (9.62 to 18.49)
High systolic blood pressure	Republic of Kiribati	13.82 (9.52 to 18.70)
High systolic blood pressure	Republic of Cuba	13.65 (9.33 to 18.16)
High systolic blood pressure	Kingdom of Thailand	13.63 (9.78 to 17.80)
High systolic blood pressure	Republic of Suriname	13.35 (9.14 to 18.23)
High systolic blood pressure	Solomon Islands	12.80 (8.75 to 17.95)
High systolic blood pressure	Federal Democratic Republic of Ethiopia	12.58 (8.41 to 17.44)
High systolic blood pressure	Plurinational State of Bolivia	12.52 (8.60 to 17.70)
High systolic blood pressure	Republic of Ecuador	12.27 (8.33 to 16.53)
High systolic blood pressure	Federated States of Micronesia	12.16 (8.01 to 16.96)
High systolic blood pressure	Republic of Korea	11.55 (7.86 to 15.68)
High systolic blood pressure	Republic of Singapore	11.01 (7.35 to 15.23)
High systolic blood pressure	Federal Democratic Republic of Nepal	10.46 (6.99 to 14.30)

High systolic blood pressure	Independent State of Papua New Guinea	9.69 (6.11 to 13.73)

Lead exposure

Risk factor	Location	Percent contribution to ASDR (%)
Lead exposure	Federal Democratic Republic of Nepal	2.13 (-0.28 to 5.28)
Lead exposure	Republic of Yemen	1.99 (-0.28 to 4.68)
Lead exposure	Islamic Republic of Afghanistan	1.96 (-0.27 to 4.81)
Lead exposure	Republic of Guatemala	1.92 (-0.28 to 4.85)
Lead exposure	Federal Republic of Somalia	1.76 (-0.25 to 4.28)
Lead exposure	Kingdom of Bhutan	1.75 (-0.24 to 4.33)
Lead exposure	Republic of Haiti	1.71 (-0.24 to 4.33)
Lead exposure	Arab Republic of Egypt	1.69 (-0.23 to 4.29)
Lead exposure	Republic of Honduras	1.69 (-0.24 to 4.04)
Lead exposure	Federal Democratic Republic of Ethiopia	1.67 (-0.23 to 4.24)
Lead exposure	People's Republic of Bangladesh	1.66 (-0.22 to 4.09)
Lead exposure	Islamic Republic of Iran	1.62 (-0.22 to 3.97)
Lead exposure	Republic of Chad	1.62 (-0.22 to 3.95)
Lead exposure	Republic of the Niger	1.54 (-0.24 to 3.84)
Lead exposure	Republic of El Salvador	1.54 (-0.22 to 3.83)
Lead exposure	Burkina Faso	1.52 (-0.21 to 3.81)
Lead exposure	Dominican Republic	1.51 (-0.21 to 3.87)
Lead exposure	Republic of India	1.38 (-0.19 to 3.52)
Lead exposure	Republic of Mali	1.37 (-0.21 to 3.41)
Lead exposure	Saint Vincent and the Grenadines	1.35 (-0.19 to 3.38)
Lead exposure	Republic of Guinea	1.35 (-0.19 to 3.33)
Lead exposure	Islamic Republic of Pakistan	1.34 (-0.19 to 3.35)
Lead exposure	Palestine	1.33 (-0.18 to 3.34)
Lead exposure	Republic of Cuba	1.33 (-0.17 to 3.50)
Lead exposure	Republic of Nicaragua	1.28 (-0.19 to 3.21)
Lead exposure	Republic of Malta	1.27 (-0.18 to 3.28)
Lead exposure	Republic of Sudan	1.26 (-0.19 to 3.15)
Lead exposure	Plurinational State of Bolivia	1.23 (-0.15 to 3.11)
Lead exposure	Kingdom of Lesotho	1.23 (-0.16 to 3.19)
Lead exposure	Syrian Arab Republic	1.23 (-0.16 to 3.06)
Lead exposure	Republic of Suriname	1.19 (-0.15 to 3.07)
Lead exposure	Republic of Mozambique	1.18 (-0.18 to 2.76)
Lead exposure	Republic of Burundi	1.18 (-0.17 to 2.97)
Lead exposure	Republic of South Sudan	1.14 (-0.15 to 2.85)
Lead exposure	Republic of Guyana	1.13 (-0.15 to 2.84)
Lead exposure	Kingdom of Cambodia	1.12 (-0.15 to 2.83)
Lead exposure	Republic of Tunisia	1.12 (-0.15 to 2.94)
Lead exposure	Lao People's Democratic Republic	1.12 (-0.15 to 2.86)

Lead exposure	Jamaica	1.11 (-0.14 to 2.85)
Lead exposure	Republic of Rwanda	1.11 (-0.15 to 2.77)
Lead exposure	Republic of Guinea-Bissau	1.09 (-0.15 to 2.76)
Lead exposure	United Mexican States	1.08 (-0.15 to 2.78)
Lead exposure	Democratic Republic of Timor-Leste	1.07 (-0.15 to 2.64)
Lead exposure	Central African Republic	1.07 (-0.14 to 2.72)
Lead exposure	State of Eritrea	1.06 (-0.14 to 2.73)
Lead exposure	Republic of Cameroon	1.05 (-0.15 to 2.60)
Lead exposure	Republic of Uganda	1.04 (-0.14 to 2.57)
Lead exposure	Grenada	1.04 (-0.14 to 2.73)
Lead exposure	People's Democratic Republic of Algeria	1.03 (-0.14 to 2.71)
Lead exposure	Republic of Liberia	1.03 (-0.15 to 2.73)
Lead exposure	Republic of Malawi	1.02 (-0.15 to 2.56)
Lead exposure	Republic of Zambia	1.02 (-0.13 to 2.58)
Lead exposure	Republic of the Gambia	1.01 (-0.13 to 2.52)
Lead exposure	Saint Lucia	1.00 (-0.14 to 2.59)
Lead exposure	Republic of Benin	0.99 (-0.14 to 2.50)
Lead exposure	Kingdom of Eswatini	0.99 (-0.14 to 2.53)
Lead exposure	Portuguese Republic	0.99 (-0.13 to 2.47)
Lead exposure	People's Republic of China	0.97 (-0.13 to 2.48)
Lead exposure	Republic of Singapore	0.96 (-0.12 to 2.45)
Lead exposure	Union of the Comoros	0.96 (-0.14 to 2.47)
Lead exposure	Republic of Botswana	0.96 (-0.14 to 2.43)
Lead exposure	Republic of Djibouti	0.96 (-0.13 to 2.37)
Lead exposure	Bolivarian Republic of Venezuela	0.95 (-0.14 to 2.44)
Lead exposure	Hashemite Kingdom of Jordan	0.94 (-0.13 to 2.50)
Lead exposure	Republic of Côte d'Ivoire	0.93 (-0.13 to 2.31)
Lead exposure	Republic of the Union of Myanmar	0.92 (-0.12 to 2.37)
Lead exposure	Democratic Republic of the Congo	0.92 (-0.13 to 2.33)
Lead exposure	Republic of Madagascar	0.92 (-0.12 to 2.35)
Lead exposure	Republic of Peru	0.92 (-0.12 to 2.29)
Lead exposure	Republic of Zimbabwe	0.91 (-0.13 to 2.29)
Lead exposure	Democratic People's Republic of Korea	0.90 (-0.11 to 2.32)
Lead exposure	Republic of Panama	0.90 (-0.12 to 2.31)
Lead exposure	Kingdom of Saudi Arabia	0.90 (-0.13 to 2.24)
Lead exposure	Solomon Islands	0.87 (-0.11 to 2.31)
Lead exposure	Republic of Namibia	0.86 (-0.11 to 2.27)
Lead exposure	Kingdom of Belgium	0.85 (-0.12 to 2.22)
Lead exposure	Sultanate of Oman	0.84 (-0.11 to 2.15)
Lead exposure	Republic of Tajikistan	0.84 (-0.12 to 2.18)
Lead exposure	State of Libya	0.84 (-0.12 to 2.12)
Lead exposure	Taiwan (Province of China)	0.83 (-0.11 to 2.15)
Lead exposure	Republic of Ecuador	0.83 (-0.10 to 2.14)
Lead exposure	Republic of Costa Rica	0.83 (-0.12 to 2.04)

Lead exposure	Republic of Angola	0.81 (-0.12 to 2.14)
Lead exposure	Australia	0.80 (-0.10 to 2.09)
Lead exposure	Togolese Republic	0.80 (-0.11 to 2.00)
Lead exposure	Hellenic Republic	0.79 (-0.10 to 2.05)
Lead exposure	Republic of Iraq	0.79 (-0.11 to 1.95)
Lead exposure	Republic of Korea	0.79 (-0.10 to 2.06)
Lead exposure	Federative Republic of Brazil	0.78 (-0.10 to 2.01)
Lead exposure	Kingdom of Morocco	0.78 (-0.11 to 2.02)
Lead exposure	Republic of Kenya	0.77 (-0.11 to 1.99)
Lead exposure	Eastern Republic of Uruguay	0.77 (-0.10 to 2.00)
Lead exposure	Kingdom of Spain	0.77 (-0.10 to 1.95)
Lead exposure	Democratic Republic of Sao Tome and Principe	0.77 (-0.10 to 2.06)
Lead exposure	Republic of Maldives	0.76 (-0.11 to 1.99)
Lead exposure	Republic of Sierra Leone	0.76 (-0.11 to 1.83)
Lead exposure	Republic of Colombia	0.75 (-0.11 to 1.93)
Lead exposure	Antigua and Barbuda	0.75 (-0.09 to 1.96)
Lead exposure	Republic of Paraguay	0.75 (-0.10 to 1.93)
Lead exposure	Belize	0.73 (-0.10 to 1.89)
Lead exposure	Bosnia and Herzegovina	0.73 (-0.10 to 1.79)
Lead exposure	Saint Kitts and Nevis	0.72 (-0.10 to 1.91)
Lead exposure	Republic of Senegal	0.72 (-0.10 to 1.82)
Lead exposure	Republic of Cabo Verde	0.72 (-0.10 to 1.83)
Lead exposure	Islamic Republic of Mauritania	0.72 (-0.10 to 1.73)
Lead exposure	Republic of Cyprus	0.72 (-0.09 to 1.80)
Lead exposure	New Zealand	0.72 (-0.09 to 1.84)
Lead exposure	State of Kuwait	0.72 (-0.10 to 1.95)
Lead exposure	Commonwealth of Dominica	0.71 (-0.10 to 1.84)
Lead exposure	Kingdom of Bahrain	0.71 (-0.09 to 1.88)
Lead exposure	Kyrgyz Republic	0.67 (-0.09 to 1.77)
Lead exposure	Republic of Turkey	0.67 (-0.09 to 1.77)
Lead exposure	Lebanese Republic	0.67 (-0.09 to 1.72)
Lead exposure	Georgia	0.65 (-0.09 to 1.63)
Lead exposure	Republic of Ghana	0.64 (-0.09 to 1.68)
Lead exposure	Republic of Equatorial Guinea	0.64 (-0.10 to 1.62)
Lead exposure	Socialist Republic of Viet Nam	0.63 (-0.09 to 1.58)
Lead exposure	Mongolia	0.62 (-0.09 to 1.59)
Lead exposure	Brunei Darussalam	0.61 (-0.08 to 1.69)
Lead exposure	Republic of Italy	0.61 (-0.08 to 1.59)
Lead exposure	Swiss Confederation	0.61 (-0.08 to 1.57)
Lead exposure	Republic of the Philippines	0.60 (-0.08 to 1.54)
Lead exposure	Tuvalu	0.59 (-0.08 to 1.37)
Lead exposure	Republic of Albania	0.59 (-0.08 to 1.55)
Lead exposure	Barbados	0.59 (-0.08 to 1.55)
Lead exposure	Republic of Poland	0.59 (-0.08 to 1.52)

Lead exposure	Argentine Republic	0.59 (-0.08 to 1.52)
Lead exposure	Gabonese Republic	0.58 (-0.07 to 1.57)
Lead exposure	Republic of South Africa	0.58 (-0.08 to 1.54)
Lead exposure	Republic of the Congo	0.58 (-0.08 to 1.53)
Lead exposure	United Arab Emirates	0.56 (-0.07 to 1.45)
Lead exposure	Malaysia	0.55 (-0.07 to 1.41)
Lead exposure	Independent State of Papua New Guinea	0.55 (-0.07 to 1.42)
Lead exposure	Republic of Armenia	0.55 (-0.07 to 1.45)
Lead exposure	Federal Republic of Nigeria	0.55 (-0.08 to 1.39)
Lead exposure	United Republic of Tanzania	0.54 (-0.08 to 1.45)
Lead exposure	French Republic	0.54 (-0.08 to 1.41)
Lead exposure	Republic of Indonesia	0.54 (-0.08 to 1.32)
Lead exposure	Republic of Uzbekistan	0.53 (-0.07 to 1.34)
Lead exposure	Republic of Azerbaijan	0.53 (-0.07 to 1.39)
Lead exposure	Kingdom of Thailand	0.52 (-0.07 to 1.38)
Lead exposure	State of Qatar	0.52 (-0.07 to 1.32)
Lead exposure	Republic of Mauritius	0.50 (-0.06 to 1.31)
Lead exposure	Puerto Rico	0.50 (-0.07 to 1.27)
Lead exposure	Ireland	0.49 (-0.07 to 1.26)
Lead exposure	Commonwealth of the Bahamas	0.48 (-0.06 to 1.26)
Lead exposure	North Macedonia	0.48 (-0.06 to 1.27)
Lead exposure	Republic of Vanuatu	0.48 (-0.06 to 1.23)
Lead exposure	United States Virgin Islands	0.48 (-0.06 to 1.26)
Lead exposure	Republic of Iceland	0.47 (-0.06 to 1.23)
Lead exposure	Republic of Kiribati	0.46 (-0.06 to 1.22)
Lead exposure	Republic of Bulgaria	0.46 (-0.06 to 1.20)
Lead exposure	Bermuda	0.44 (-0.06 to 1.15)
Lead exposure	Republic of the Marshall Islands	0.44 (-0.06 to 1.10)
Lead exposure	Kingdom of the Netherlands	0.43 (-0.06 to 1.11)
Lead exposure	United States of America	0.43 (-0.05 to 1.13)
Lead exposure	United Kingdom of Great Britain and Northern Ireland	0.43 (-0.06 to 1.10)
Lead exposure	Democratic Socialist Republic of Sri Lanka	0.43 (-0.06 to 1.12)
Lead exposure	Turkmenistan	0.43 (-0.06 to 1.10)
Lead exposure	Republic of Croatia	0.43 (-0.06 to 1.13)
Lead exposure	Grand Duchy of Luxembourg	0.41 (-0.05 to 1.05)
Lead exposure	Republic of Serbia	0.41 (-0.05 to 1.08)
Lead exposure	Canada	0.41 (-0.05 to 1.12)
Lead exposure	Kingdom of Norway	0.40 (-0.06 to 1.06)
Lead exposure	Republic of Moldova	0.40 (-0.05 to 1.03)
Lead exposure	Romania	0.40 (-0.06 to 1.03)
Lead exposure	State of Israel	0.39 (-0.05 to 1.09)
Lead exposure	Kingdom of Denmark	0.39 (-0.05 to 1.02)
Lead exposure	Republic of Austria	0.39 (-0.05 to 0.97)
Lead exposure	Principality of Monaco	0.39 (-0.05 to 0.98)

Lead exposure	Republic of Trinidad and Tobago	0.38 (-0.05 to 1.03)
Lead exposure	Greenland	0.38 (-0.05 to 0.99)
Lead exposure	Federated States of Micronesia	0.38 (-0.05 to 0.99)
Lead exposure	Principality of Andorra	0.38 (-0.05 to 0.98)
Lead exposure	Republic of Seychelles	0.37 (-0.05 to 0.98)
Lead exposure	Republic of Belarus	0.37 (-0.05 to 1.01)
Lead exposure	Republic of San Marino	0.37 (-0.05 to 0.98)
Lead exposure	Czech Republic	0.37 (-0.05 to 0.94)
Lead exposure	Republic of Nauru	0.37 (-0.05 to 0.82)
Lead exposure	Kingdom of Tonga	0.37 (-0.05 to 0.95)
Lead exposure	Slovak Republic	0.36 (-0.05 to 0.92)
Lead exposure	Montenegro	0.35 (-0.05 to 0.92)
Lead exposure	Hungary	0.35 (-0.05 to 0.91)
Lead exposure	Japan	0.34 (-0.05 to 0.91)
Lead exposure	Independent State of Samoa	0.33 (-0.04 to 0.86)
Lead exposure	Russian Federation	0.33 (-0.04 to 0.85)
Lead exposure	Republic of Slovenia	0.32 (-0.04 to 0.86)
Lead exposure	Republic of Estonia	0.31 (-0.04 to 0.83)
Lead exposure	Federal Republic of Germany	0.31 (-0.04 to 0.80)
Lead exposure	Republic of Latvia	0.31 (-0.04 to 0.80)
Lead exposure	Republic of Fiji	0.30 (-0.04 to 0.76)
Lead exposure	Kingdom of Sweden	0.29 (-0.04 to 0.76)
Lead exposure	Republic of Kazakhstan	0.29 (-0.04 to 0.78)
Lead exposure	Tokelau	0.29 (-0.04 to 0.76)
Lead exposure	Republic of Lithuania	0.28 (-0.04 to 0.74)
Lead exposure	Ukraine	0.28 (-0.04 to 0.71)
Lead exposure	Republic of Chile	0.26 (-0.04 to 0.69)
Lead exposure	Cook Islands	0.22 (-0.03 to 0.58)
Lead exposure	American Samoa	0.21 (-0.03 to 0.53)
Lead exposure	Republic of Finland	0.21 (-0.03 to 0.54)
Lead exposure	Republic of Palau	0.20 (-0.02 to 0.54)
Lead exposure	Republic of Niue	0.20 (-0.03 to 0.51)
Lead exposure	Guam	0.20 (-0.02 to 0.52)
Lead exposure	Northern Mariana Islands	0.19 (-0.02 to 0.50)

Smoking

Risk factor	Location	Percent contribution to ASDR (%)
Smoking	Lebanese Republic	48.47 (41.65 to 55.20)
Smoking	Georgia	48.09 (42.09 to 53.79)
Smoking	Republic of Belarus	47.75 (42.33 to 52.65)
Smoking	Hellenic Republic	46.83 (40.43 to 53.29)
Smoking	Republic of Albania	46.29 (38.73 to 53.73)
Smoking	Hashemite Kingdom of Jordan	46.27 (39.40 to 52.30)

Smoking	Bosnia and Herzegovina	45.50 (38.71 to 52.02)
Smoking	People's Republic of China	44.74 (37.99 to 50.98)
Smoking	Republic of Kiribati	44.63 (37.98 to 51.02)
Smoking	Republic of Iraq	43.90 (37.34 to 50.41)
Smoking	Montenegro	42.75 (35.74 to 49.64)
Smoking	Republic of Croatia	42.66 (36.52 to 49.12)
Smoking	Arab Republic of Egypt	42.37 (36.34 to 48.24)
Smoking	Republic of Bulgaria	42.17 (36.80 to 47.06)
Smoking	Ukraine	41.94 (34.38 to 48.99)
Smoking	Kyrgyz Republic	41.82 (36.70 to 46.54)
Smoking	Republic of Tunisia	41.25 (32.55 to 48.73)
Smoking	Kingdom of Denmark	41.02 (33.96 to 48.51)
Smoking	Palestine	40.64 (34.26 to 47.48)
Smoking	Republic of Paraguay	40.31 (33.01 to 47.72)
Smoking	North Macedonia	40.03 (32.74 to 47.22)
Smoking	Republic of Turkey	39.92 (34.19 to 45.75)
Smoking	Greenland	39.81 (33.19 to 46.92)
Smoking	Republic of Moldova	39.62 (34.18 to 44.99)
Smoking	State of Kuwait	39.50 (32.58 to 46.53)
Smoking	Republic of Serbia	39.41 (33.67 to 45.93)
Smoking	Republic of Armenia	39.26 (34.56 to 43.90)
Smoking	Czech Republic	39.01 (33.12 to 45.36)
Smoking	Republic of Latvia	38.67 (33.36 to 43.94)
Smoking	Republic of Azerbaijan	38.66 (28.06 to 46.99)
Smoking	Republic of Yemen	38.48 (31.21 to 46.37)
Smoking	Republic of Lithuania	38.32 (32.83 to 43.73)
Smoking	Slovak Republic	38.32 (32.29 to 44.72)
Smoking	Russian Federation	38.18 (33.76 to 42.97)
Smoking	Kingdom of Spain	38.06 (31.71 to 44.61)
Smoking	Republic of Cyprus	37.98 (31.08 to 45.19)
Smoking	Syrian Arab Republic	37.80 (31.58 to 43.77)
Smoking	Kingdom of Cambodia	37.35 (28.74 to 46.20)
Smoking	Republic of San Marino	37.17 (29.23 to 45.05)
Smoking	Taiwan (Province of China)	36.92 (31.81 to 41.78)
Smoking	Federal Republic of Germany	36.87 (31.13 to 43.36)
Smoking	Republic of Estonia	36.84 (31.19 to 42.81)
Smoking	United States of America	36.77 (30.52 to 43.28)
Smoking	Hungary	36.74 (31.45 to 41.82)
Smoking	Romania	36.29 (31.43 to 41.25)
Smoking	Republic of Poland	36.21 (30.92 to 42.01)
Smoking	Democratic People's Republic of Korea	36.12 (29.98 to 42.69)
Smoking	Kingdom of Tonga	35.86 (29.18 to 43.27)
Smoking	Eastern Republic of Uruguay	35.85 (30.09 to 41.66)
Smoking	Kingdom of Belgium	35.59 (29.08 to 41.71)

Smoking	Republic of Maldives	35.30 (25.44 to 44.97)
Smoking	Guam	35.22 (29.63 to 41.96)
Smoking	People's Democratic Republic of Algeria	35.18 (27.98 to 41.89)
Smoking	Republic of Austria	35.03 (29.22 to 40.78)
Smoking	Kingdom of Sweden	34.55 (27.82 to 42.26)
Smoking	Grand Duchy of Luxembourg	34.54 (27.97 to 41.70)
Smoking	Federative Republic of Brazil	34.53 (28.61 to 40.69)
Smoking	Dominican Republic	34.45 (28.17 to 41.58)
Smoking	Republic of Italy	34.38 (28.59 to 40.57)
Smoking	Republic of Mauritius	34.37 (29.06 to 39.99)
Smoking	Kingdom of the Netherlands	34.31 (28.10 to 41.57)
Smoking	Federal Democratic Republic of Nepal	34.29 (27.45 to 41.24)
Smoking	State of Libya	33.70 (26.15 to 40.76)
Smoking	Lao People's Democratic Republic	33.69 (26.25 to 40.91)
Smoking	French Republic	33.65 (27.88 to 39.89)
Smoking	Principality of Andorra	33.48 (26.62 to 41.13)
Smoking	Swiss Confederation	33.48 (27.53 to 40.17)
Smoking	Argentine Republic	33.23 (28.02 to 39.05)
Smoking	Republic of Malta	33.05 (27.18 to 39.23)
Smoking	Canada	32.96 (26.67 to 39.38)
Smoking	Republic of Slovenia	32.82 (27.28 to 39.23)
Smoking	Republic of Seychelles	32.78 (24.40 to 41.37)
Smoking	People's Republic of Bangladesh	32.78 (24.90 to 42.37)
Smoking	Mongolia	32.63 (25.52 to 39.50)
Smoking	Socialist Republic of Viet Nam	32.63 (26.72 to 39.42)
Smoking	Republic of Rwanda	32.55 (25.02 to 40.41)
Smoking	Kingdom of Bahrain	32.41 (26.13 to 39.02)
Smoking	Republic of Cuba	32.30 (26.38 to 38.93)
Smoking	Republic of Kazakhstan	31.92 (27.48 to 35.96)
Smoking	United Kingdom of Great Britain and Northern Ireland	31.88 (25.41 to 39.08)
Smoking	Republic of Tajikistan	31.85 (25.71 to 38.30)
Smoking	Republic of the Philippines	31.34 (25.56 to 37.05)
Smoking	Principality of Monaco	31.34 (25.10 to 38.70)
Smoking	Ireland	31.09 (25.01 to 37.61)
Smoking	Republic of Iceland	30.88 (24.63 to 37.85)
Smoking	New Zealand	30.61 (24.55 to 37.20)
Smoking	Turkmenistan	30.59 (25.54 to 35.93)
Smoking	Republic of Suriname	30.37 (24.84 to 36.33)
Smoking	Islamic Republic of Iran	30.30 (24.84 to 35.66)
Smoking	Republic of Costa Rica	30.06 (24.73 to 35.99)
Smoking	Independent State of Samoa	29.97 (23.49 to 36.78)
Smoking	Republic of Korea	29.91 (23.92 to 36.27)
Smoking	Republic of Sudan	29.75 (23.72 to 37.20)
Smoking	Republic of Indonesia	29.73 (21.49 to 37.36)

Smoking	Japan	29.69 (24.82 to 34.90)
Smoking	Portuguese Republic	29.56 (24.70 to 34.39)
Smoking	State of Israel	29.38 (23.84 to 35.21)
Smoking	Malaysia	29.15 (23.49 to 34.88)
Smoking	Republic of the Union of Myanmar	29.00 (23.17 to 35.46)
Smoking	Republic of Finland	28.90 (23.21 to 34.99)
Smoking	Kingdom of Thailand	28.58 (23.40 to 33.90)
Smoking	Kingdom of Lesotho	28,33 (20,47 to 39,85)
Smoking	Democratic Republic of Timor-Leste	28.32 (20.77 to 36.25)
Smoking	Brunei Darussalam	28.26 (22.78 to 35.06)
Smoking	Solomon Islands	27.86 (21.73 to 36.64)
Smoking	Republic of Zimbabwe	27.75 (21.01 to 34.39)
Smoking	Federated States of Micronesia	27.65 (21.96 to 34.11)
Smoking	Republic of Malawi	27.36 (17.96 to 35.84)
Smoking	Republic of Botswana	27.21 (21.71 to 32.68)
Smoking	Puerto Rico	26.87 (21.03 to 33.14)
Smoking	Bermuda	26.71 (20.87 to 33.37)
Smoking	Republic of Nicaragua	26.63 (21.27 to 32.52)
Smoking	State of Qatar	26.58 (20.65 to 34.08)
Smoking	Tuvalu	26.51 (20.88 to 32.98)
Smoking	Cook Islands	26.27 (19.20 to 34.32)
Smoking	American Samoa	26.26 (20.94 to 31.31)
Smoking	Islamic Republic of Afghanistan	26.19 (15.11 to 34.36)
Smoking	Sultanate of Oman	25.99 (19.19 to 33.49)
Smoking	United Republic of Tanzania	25.95 (19.63 to 32.97)
Smoking	Republic of Honduras	25.92 (20.22 to 32.37)
Smoking	Republic of Djibouti	25.90 (19.26 to 32.94)
Smoking	Republic of Chile	25.86 (21.56 to 30.19)
Smoking	Islamic Republic of Pakistan	25.56 (19.30 to 32.94)
Smoking	Republic of Namibia	25.41 (20.14 to 31.11)
Smoking	Republic of Nauru	25.16 (16.99 to 36.14)
Smoking	Jamaica	24.85 (19.90 to 30.62)
Smoking	Republic of Trinidad and Tobago	24.78 (19.81 to 30.10)
Smoking	Republic of Fiji	24.52 (20.28 to 29.61)
Smoking	Republic of Palau	24.25 (19.33 to 30.32)
Smoking	Republic of Niue	23.98 (18.66 to 29.86)
Smoking	Republic of India	23.96 (18.35 to 29.99)
Smoking	Republic of the Marshall Islands	23.86 (18.72 to 31.09)
Smoking	Kingdom of Norway	23.47 (18.58 to 29.50)
Smoking	Northern Mariana Islands	23.38 (18.54 to 28.54)
Smoking	Kingdom of Morocco	23.36 (18.05 to 28.68)
Smoking	United Arab Emirates	23.30 (17.79 to 29.15)
Smoking	Republic of El Salvador	23.18 (18.48 to 28.21)
Smoking	Independent State of Papua New Guinea	23.08 (17.55 to 29.28)

Smoking	Bolivarian Republic of Venezuela	23.05 (18.53 to 27.74)
Smoking	Tokelau	22.86 (17.34 to 29.46)
Smoking	Republic of Peru	22.62 (17.75 to 28.12)
Smoking	Republic of Uzbekistan	22.55 (18.82 to 26.35)
Smoking	Republic of Panama	22.47 (17.99 to 27.66)
Smoking	Antigua and Barbuda	22.47 (17.40 to 28.94)
Smoking	Australia	22.43 (17.56 to 28.05)
Smoking	United Mexican States	21.85 (17.71 to 26.01)
Smoking	Commonwealth of the Bahamas	21.73 (16.86 to 27.62)
Smoking	Republic of Guyana	21.66 (17.00 to 26.65)
Smoking	Democratic Socialist Republic of Sri Lanka	21.38 (17.09 to 26.37)
Smoking	United States Virgin Islands	21.15 (15.41 to 27.57)
Smoking	Saint Vincent and the Grenadines	21.14 (16.63 to 26.18)
Smoking	Kingdom of Saudi Arabia	21.14 (15.14 to 28.32)
Smoking	Belize	21.09 (16.62 to 26.11)
Smoking	Republic of South Africa	20.87 (17.20 to 24.94)
Smoking	Republic of Guatemala	20.83 (16.33 to 25.57)
Smoking	Republic of Angola	20.79 (15.80 to 25.93)
Smoking	Republic of Colombia	20.33 (16.40 to 24.83)
Smoking	Plurinational State of Bolivia	20.17 (15.73 to 25.25)
Smoking	Kingdom of Bhutan	20.13 (13.05 to 27.47)
Smoking	Republic of Vanuatu	19.93 (14.03 to 26.17)
Smoking	Republic of the Congo	19.76 (14.49 to 25.88)
Smoking	Republic of Ecuador	19.75 (15.79 to 23.77)
Smoking	Togolese Republic	19.62 (13.10 to 26.22)
Smoking	Republic of Singapore	19.39 (15.48 to 23.59)
Smoking	Grenada	18.94 (14.82 to 23.40)
Smoking	Republic of Zambia	18.88 (14.21 to 23.60)
Smoking	Barbados	18.74 (14.28 to 23.85)
Smoking	Union of the Comoros	18.68 (12.05 to 25.49)
Smoking	Republic of Mozambique	18.42 (10.65 to 24.98)
Smoking	Commonwealth of Dominica	17.55 (13.45 to 22.49)
Smoking	Saint Kitts and Nevis	17.37 (12.86 to 22.34)
Smoking	Republic of South Sudan	17.20 (8.87 to 25.16)
Smoking	Saint Lucia	16.92 (13.14 to 21.80)
Smoking	Republic of Equatorial Guinea	16.81 (12.21 to 22.00)
Smoking	Republic of Côte d'Ivoire	16.58 (11.69 to 21.91)
Smoking	Republic of the Gambia	16.42 (10.52 to 22.52)
Smoking	Kingdom of Eswatini	16.17 (12.29 to 20.87)
Smoking	Gabonese Republic	15.91 (11.98 to 20.29)
Smoking	Republic of Ghana	15.63 (11.43 to 20.59)
Smoking	Republic of Kenya	15.44 (12.10 to 19.20)
Smoking	Republic of Guinea	15.30 (8.95 to 21.74)
Smoking	Republic of Mali	14.78 (6.03 to 23.64)

Smoking	Republic of Uganda	14.50 (10.53 to 19.09)
Smoking	Republic of Sierra Leone	14.38 (8.82 to 19.60)
Smoking	Republic of Haiti	14.30 (10.12 to 19.43)
Smoking	Central African Republic	14.23 (7.73 to 21.34)
Smoking	Federal Republic of Somalia	13.56 (4.55 to 25.27)
Smoking	Republic of Chad	13.36 (6.94 to 20.27)
Smoking	Republic of Senegal	12.94 (8.12 to 18.06)
Smoking	Republic of Cameroon	12.57 (8.92 to 16.37)
Smoking	Republic of Madagascar	12.50 (8.46 to 16.79)
Smoking	Republic of Burundi	12.24 (6.73 to 17.27)
Smoking	Republic of Cabo Verde	12.05 (8.96 to 15.72)
Smoking	Republic of Guinea-Bissau	11.29 (6.13 to 16.54)
Smoking	Republic of Liberia	11.20 (6.02 to 15.98)
Smoking	Islamic Republic of Mauritania	11.06 (7.40 to 15.57)
Smoking	Democratic Republic of Sao Tome and Principe	10.83 (7.33 to 14.82)
Smoking	Republic of Benin	10.41 (6.36 to 14.55)
Smoking	Democratic Republic of the Congo	10.13 (6.06 to 14.33)
Smoking	State of Eritrea	9.87 (6.00 to 15.05)
Smoking	Burkina Faso	9.81 (4.67 to 15.15)
Smoking	Federal Republic of Nigeria	8.70 (6.03 to 11.84)
Smoking	Federal Democratic Republic of Ethiopia	8.20 (4.95 to 11.10)
Smoking	Republic of the Niger	6.93 (2.19 to 12.38)

Data for Figure 7

Fig 7A. Percentage contribution of risk factors to AA- ASDR in Europe in 2021

Risk factor	Location	Percent contribution to ASDR (%)
Diet high in sodium	Central Europe	1.53 (0.26 to 3.66)
Diet low in fruits	Central Europe	3.86 (2.69 to 5.10)
Diet low in vegetables	Central Europe	1.85 (1.13 to 2.81)
High body-mass index	Central Europe	11.01 (5.89 to 18.69)
High systolic blood pressure	Central Europe	20.29 (15.31 to 25.40)
Lead exposure	Central Europe	0.48 (-0.07 to 1.24)
Smoking	Central Europe	38.06 (32.84 to 43.23)
Diet high in sodium	Eastern Europe	0.70 (0.03 to 2.27)
Diet low in fruits	Eastern Europe	4.65 (3.25 to 6.15)
Diet low in vegetables	Eastern Europe	2.89 (1.87 to 4.15)
High body-mass index	Eastern Europe	11.37 (6.03 to 19.53)
High systolic blood pressure	Eastern Europe	20.66 (15.72 to 25.95)
Lead exposure	Eastern Europe	0.32 (-0.04 to 0.84)
Smoking	Eastern Europe	39.29 (34.77 to 43.90)
Diet high in sodium	Western Europe	0.53 (0.01 to 2.12)
Diet low in fruits	Western Europe	3.06 (2.15 to 4.02)
Diet low in vegetables	Western Europe	2.98 (1.96 to 4.25)
High body-mass index	Western Europe	8.76 (4.63 to 15.32)
High systolic blood pressure	Western Europe	17.40 (13.12 to 22.15)
Lead exposure	Western Europe	0.50 (-0.07 to 1.28)
Smoking	Western Europe	34.82 (29.10 to 41.11)

Fig 7B. Percentage contribution of risk factors to AA-ASDR in Europe grouped by sex in 2021

Risk factor	Sex	Location	Percent contribution to ASDR (%)
Diet high in sodium	Male	Central Europe	1.76 (0.33 to 4.12)
Diet high in sodium	Female	Central Europe	1.07 (0.10 to 2.93)
Diet low in fruits	Male	Central Europe	3.74 (2.59 to 4.96)
Diet low in fruits	Female	Central Europe	3.99 (2.77 to 5.31)
Diet low in vegetables	Male	Central Europe	1.86 (1.13 to 2.81)
Diet low in vegetables	Female	Central Europe	1.84 (1.10 to 2.88)
High body-mass index	Male	Central Europe	10.40 (5.51 to 18.00)
High body-mass index	Female	Central Europe	11.78 (6.32 to 19.60)
High systolic blood pressure	Male	Central Europe	19.83 (15.00 to 24.79)
High systolic blood pressure	Female	Central Europe	20.59 (15.93 to 26.04)
Lead exposure	Male	Central Europe	0.55 (-0.07 to 1.42)
Lead exposure	Female	Central Europe	0.36 (-0.05 to 0.93)
Smoking	Male	Central Europe	44.04 (37.71 to 50.06)
Smoking	Female	Central Europe	22.99 (18.93 to 27.04)
Diet high in sodium	Male	Eastern Europe	0.86 (0.04 to 2.64)
Diet high in sodium	Female	Eastern Europe	0.38 (0.00 to 1.58)
Diet low in fruits	Male	Eastern Europe	4.53 (3.20 to 5.98)
Diet low in fruits	Female	Eastern Europe	4.71 (3.29 to 6.24)
Diet low in vegetables	Male	Eastern Europe	2.88 (1.84 to 4.20)
Diet low in vegetables	Female	Eastern Europe	2.86 (1.82 to 4.18)
High body-mass index	Male	Eastern Europe	10.15 (5.38 to 17.77)
High body-mass index	Female	Eastern Europe	13.22 (7.00 to 22.24)
High systolic blood pressure	Male	Eastern Europe	20.16 (15.35 to 25.50)
High systolic blood pressure	Female	Eastern Europe	21.11 (15.92 to 26.32)
Lead exposure	Male	Eastern Europe	0.35 (-0.05 to 0.92)
Lead exposure	Female	Eastern Europe	0.28 (-0.04 to 0.71)
Smoking	Male	Eastern Europe	53.67 (47.13 to 60.35)
Smoking	Female	Eastern Europe	11.07 (9.14 to 13.40)
Diet high in sodium	Male	Western Europe	0.64 (0.01 to 2.39)
Diet high in sodium	Female	Western Europe	0.31 (0.00 to 1.57)
Diet low in fruits	Male	Western Europe	2.93 (2.07 to 3.86)
Diet low in fruits	Female	Western Europe	3.26 (2.24 to 4.35)
Diet low in vegetables	Male	Western Europe	2.93 (1.93 to 4.17)
Diet low in vegetables	Female	Western Europe	3.05 (1.99 to 4.35)
High body-mass index	Male	Western Europe	8.44 (4.46 to 14.76)
High body-mass index	Female	Western Europe	9.15 (4.75 to 15.83)
High systolic blood pressure	Male	Western Europe	16.87 (12.58 to 21.71)
High systolic blood pressure	Female	Western Europe	18.07 (13.43 to 22.74)
Lead exposure	Male	Western Europe	0.56 (-0.07 to 1.43)

Lead exposure	Female	Western Europe	0.40 (-0.05 to 1.03)
Smoking	Male	Western Europe	39.63 (32.93 to 46.83)
Smoking	Female	Western Europe	23.07 (18.65 to 27.84)

AA, aortic aneurysm; ASDR, age-standardized death rates.

Fig 7C. AA-ASDR attributable to risk factors in Europe grouped by sex from 1990 to 2021

Year	Risk factor	Sex	Location	ASDR per 100,000
1990	Diet high in sodium	Male	Central Europe	0.09 (0.02 to 0.21)
1990	Diet high in sodium	Female	Central Europe	0.02 (0.00 to 0.05)
1990	Diet high in sodium	Both	Central Europe	0.05 (0.01 to 0.11)
2021	Diet high in sodium	Male	Central Europe	0.08 (0.02 to 0.20)
2021	Diet high in sodium	Female	Central Europe	0.02 (0.00 to 0.05)
2021	Diet high in sodium	Both	Central Europe	0.04 (0.01 to 0.11)
1990	Diet low in fruits	Male	Central Europe	0.24 (0.17 to 0.32)
1990	Diet low in fruits	Female	Central Europe	0.08 (0.06 to 0.11)
1990	Diet low in fruits	Both	Central Europe	0.15 (0.10 to 0.20)
2021	Diet low in fruits	Male	Central Europe	0.18 (0.12 to 0.24)
2021	Diet low in fruits	Female	Central Europe	0.07 (0.04 to 0.09)
2021	Diet low in fruits	Both	Central Europe	0.11 (0.08 to 0.15)
1990	Diet low in vegetables	Male	Central Europe	0.15 (0.10 to 0.22)
1990	Diet low in vegetables	Female	Central Europe	0.05 (0.03 to 0.07)
1990	Diet low in vegetables	Both	Central Europe	0.09 (0.06 to 0.13)
2021	Diet low in vegetables	Male	Central Europe	0.09 (0.05 to 0.14)
2021	Diet low in vegetables	Female	Central Europe	0.03 (0.02 to 0.05)
2021	Diet low in vegetables	Both	Central Europe	0.05 (0.03 to 0.08)
1990	High body-mass index	Male	Central Europe	0.47 (0.24 to 0.81)
1990	High body-mass index	Female	Central Europe	0.17 (0.09 to 0.29)
1990	High body-mass index	Both	Central Europe	0.30 (0.16 to 0.50)
2021	High body-mass index	Male	Central Europe	0.49 (0.26 to 0.84)
2021	High body-mass index	Female	Central Europe	0.19 (0.10 to 0.33)
2021	High body-mass index	Both	Central Europe	0.32 (0.17 to 0.56)
1990	High systolic blood pressure	Male	Central Europe	1.15 (0.89 to 1.43)
1990	High systolic blood pressure	Female	Central Europe	0.39 (0.30 to 0.50)
1990	High systolic blood pressure	Both	Central Europe	0.70 (0.54 to 0.87)
2021	High systolic blood pressure	Male	Central Europe	0.94 (0.69 to 1.20)
2021	High systolic blood pressure	Female	Central Europe	0.34 (0.25 to 0.46)
2021	High systolic blood pressure	Both	Central Europe	0.59 (0.45 to 0.76)
1990	Lead exposure	Male	Central Europe	0.03 (0.00 to 0.07)
1990	Lead exposure	Female	Central Europe	0.01 (0.00 to 0.01)
1990	Lead exposure	Both	Central Europe	0.01 (0.00 to 0.04)
2021	Lead exposure	Male	Central Europe	0.03 (0.00 to 0.07)
2021	Lead exposure	Female	Central Europe	0.01 (0.00 to 0.02)

2021	Lead exposure	Both	Central Europe	0.01 (0.00 to 0.04)
1990	Smoking	Male	Central Europe	2.75 (2.40 to 3.11)
1990	Smoking	Female	Central Europe	0.40 (0.34 to 0.47)
1990	Smoking	Both	Central Europe	1.38 (1.21 to 1.55)
2021	Smoking	Male	Central Europe	2.09 (1.77 to 2.46)
2021	Smoking	Female	Central Europe	0.38 (0.29 to 0.49)
2021	Smoking	Both	Central Europe	1.11 (0.93 to 1.30)
1990	Diet high in sodium	Male	Eastern Europe	0.04 (0.00 to 0.13)
1990	Diet high in sodium	Female	Eastern Europe	0.01 (0.00 to 0.02)
1990	Diet high in sodium	Both	Eastern Europe	0.02 (0.00 to 0.06)
2021	Diet high in sodium	Male	Eastern Europe	0.06 (0.00 to 0.18)
2021	Diet high in sodium	Female	Eastern Europe	0.01 (0.00 to 0.04)
2021	Diet high in sodium	Both	Eastern Europe	0.03 (0.00 to 0.09)
1990	Diet low in fruits	Male	Eastern Europe	0.24 (0.17 to 0.32)
1990	Diet low in fruits	Female	Eastern Europe	0.08 (0.06 to 0.11)
1990	Diet low in fruits	Both	Eastern Europe	0.14 (0.10 to 0.18)
2021	Diet low in fruits	Male	Eastern Europe	0.30 (0.20 to 0.41)
2021	Diet low in fruits	Female	Eastern Europe	0.10 (0.07 to 0.14)
2021	Diet low in fruits	Both	Eastern Europe	0.18 (0.12 to 0.24)
1990	Diet low in vegetables	Male	Eastern Europe	0.13 (0.08 to 0.19)
1990	Diet low in vegetables	Female	Eastern Europe	0.04 (0.03 to 0.06)
1990	Diet low in vegetables	Both	Eastern Europe	0.07 (0.05 to 0.10)
2021	Diet low in vegetables	Male	Eastern Europe	0.19 (0.12 to 0.28)
2021	Diet low in vegetables	Female	Eastern Europe	0.06 (0.04 to 0.09)
2021	Diet low in vegetables	Both	Eastern Europe	0.11 (0.07 to 0.16)
1990	High body-mass index	Male	Eastern Europe	0.32 (0.18 to 0.56)
1990	High body-mass index	Female	Eastern Europe	0.16 (0.08 to 0.26)
1990	High body-mass index	Both	Eastern Europe	0.22 (0.12 to 0.36)
2021	High body-mass index	Male	Eastern Europe	0.66 (0.34 to 1.14)
2021	High body-mass index	Female	Eastern Europe	0.29 (0.15 to 0.49)
2021	High body-mass index	Both	Eastern Europe	0.44 (0.23 to 0.73)
1990	High systolic blood pressure	Male	Eastern Europe	0.92 (0.69 to 1.17)
1990	High systolic blood pressure	Female	Eastern Europe	0.34 (0.26 to 0.41)
1990	High systolic blood pressure	Both	Eastern Europe	0.53 (0.41 to 0.67)
2021	High systolic blood pressure	Male	Eastern Europe	1.32 (0.96 to 1.67)
2021	High systolic blood pressure	Female	Eastern Europe	0.46 (0.33 to 0.59)
2021	High systolic blood pressure	Both	Eastern Europe	0.79 (0.59 to 1.00)
1990	Lead exposure	Male	Eastern Europe	0.01 (0.00 to 0.04)
1990	Lead exposure	Female	Eastern Europe	0.00 (0.00 to 0.01)
1990	Lead exposure	Both	Eastern Europe	0.01 (0.00 to 0.02)
2021	Lead exposure	Male	Eastern Europe	0.02 (0.00 to 0.06)
2021	Lead exposure	Female	Eastern Europe	0.01 (0.00 to 0.02)

2021	Lead exposure	Both	Eastern Europe	0.01 (0.00 to 0.03)
1990	Smoking	Male	Eastern Europe	2.54 (2.25 to 2.82)
1990	Smoking	Female	Eastern Europe	0.14 (0.11 to 0.16)
1990	Smoking	Both	Eastern Europe	0.96 (0.86 to 1.06)
2021	Smoking	Male	Eastern Europe	3.50 (2.97 to 4.02)
2021	Smoking	Female	Eastern Europe	0.24 (0.19 to 0.32)
2021	Smoking	Both	Eastern Europe	1.50 (1.28 to 1.72)
2021	Diet high in sodium	Male	Western Europe	0.03 (0.00 to 0.09)
2021	Diet high in sodium	Female	Western Europe	0.00 (0.00 to 0.02)
2021	Diet high in sodium	Both	Western Europe	0.01 (0.00 to 0.05)
1990	Diet high in sodium	Male	Western Europe	0.04 (0.00 to 0.16)
1990	Diet high in sodium	Female	Western Europe	0.01 (0.00 to 0.03)
1990	Diet high in sodium	Both	Western Europe	0.02 (0.00 to 0.08)
1990	Diet low in fruits	Male	Western Europe	0.34 (0.24 to 0.46)
1990	Diet low in fruits	Female	Western Europe	0.11 (0.08 to 0.15)
1990	Diet low in fruits	Both	Western Europe	0.20 (0.14 to 0.27)
2021	Diet low in fruits	Male	Western Europe	0.12 (0.08 to 0.16)
2021	Diet low in fruits	Female	Western Europe	0.05 (0.03 to 0.07)
2021	Diet low in fruits	Both	Western Europe	0.08 (0.05 to 0.10)
1990	Diet low in vegetables	Male	Western Europe	0.32 (0.21 to 0.46)
1990	Diet low in vegetables	Female	Western Europe	0.10 (0.07 to 0.14)
1990	Diet low in vegetables	Both	Western Europe	0.19 (0.12 to 0.27)
2021	Diet low in vegetables	Male	Western Europe	0.12 (0.08 to 0.17)
2021	Diet low in vegetables	Female	Western Europe	0.05 (0.03 to 0.07)
2021	Diet low in vegetables	Both	Western Europe	0.08 (0.05 to 0.11)
1990	High body-mass index	Male	Western Europe	0.69 (0.37 to 1.16)
1990	High body-mass index	Female	Western Europe	0.21 (0.11 to 0.36)
1990	High body-mass index	Both	Western Europe	0.40 (0.22 to 0.68)
2021	High body-mass index	Male	Western Europe	0.33 (0.17 to 0.58)
2021	High body-mass index	Female	Western Europe	0.14 (0.07 to 0.24)
2021	High body-mass index	Both	Western Europe	0.23 (0.12 to 0.39)
1990	High systolic blood pressure	Male	Western Europe	1.95 (1.49 to 2.43)
1990	High systolic blood pressure	Female	Western Europe	0.60 (0.46 to 0.75)
1990	High systolic blood pressure	Both	Western Europe	1.13 (0.86 to 1.41)
2021	High systolic blood pressure	Male	Western Europe	0.67 (0.50 to 0.85)
2021	High systolic blood pressure	Female	Western Europe	0.27 (0.20 to 0.35)
2021	High systolic blood pressure	Both	Western Europe	0.45 (0.33 to 0.57)
1990	Lead exposure	Male	Western Europe	0.04 (-0.01 to 0.10)
1990	Lead exposure	Female	Western Europe	0.01 (0.00 to 0.02)
1990	Lead exposure	Both	Western Europe	0.02 (0.00 to 0.05)
2021	Lead exposure	Male	Western Europe	0.02 (0.00 to 0.06)
2021	Lead exposure	Female	Western Europe	0.01 (0.00 to 0.02)

2021	Lead exposure	Both	Western Europe	0.01 (0.00 to 0.03)
1990	Smoking	Male	Western Europe	4.32 (3.70 to 4.96)
1990	Smoking	Female	Western Europe	0.72 (0.59 to 0.87)
1990	Smoking	Both	Western Europe	2.15 (1.83 to 2.48)
2021	Smoking	Male	Western Europe	1.57 (1.30 to 1.87)
2021	Smoking	Female	Western Europe	0.35 (0.27 to 0.43)
2021	Smoking	Both	Western Europe	0.89 (0.74 to 1.07)

AA, aortic aneurysm; ASDR, age-standardized death rates.

Data for Figure 8

Fig 8A. Percentage contribution of risk factors to AA-ASDR in Asia in 2021

Risk factor	Location	Percent contribution to ASDR (%)
Diet high in sodium	Central Asia	0.77 (0.03 to 2.59)
Diet low in fruits	Central Asia	3.73 (2.60 to 4.99)
Diet low in vegetables	Central Asia	0.87 (0.50 to 1.41)
High body-mass index	Central Asia	9.87 (5.44 to 16.87)
High systolic blood pressure	Central Asia	20.82 (15.91 to 26.16)
Lead exposure	Central Asia	0.50 (-0.07 to 1.29)
Smoking	Central Asia	34.08 (29.64 to 38.25)
Diet high in sodium	East Asia	2.05 (0.39 to 4.80)
Diet low in fruits	East Asia	3.20 (2.17 to 4.30)
Diet low in vegetables	East Asia	0.52 (0.26 to 0.88)
High body-mass index	East Asia	5.69 (3.13 to 9.71)
High systolic blood pressure	East Asia	15.69 (11.28 to 20.21)
Lead exposure	East Asia	0.96 (-0.13 to 2.44)
Smoking	East Asia	43.57 (37.28 to 49.59)
Diet high in sodium	Japan	1.05 (0.06 to 3.16)
Diet low in fruits	Japan	4.11 (2.78 to 5.46)
Diet low in vegetables	Japan	1.29 (0.72 to 2.04)
High body-mass index	Japan	4.57 (2.49 to 7.25)
High systolic blood pressure	Japan	17.64 (13.11 to 22.97)
Lead exposure	Japan	0.34 (-0.05 to 0.91)
Smoking	Japan	29.69 (24.82 to 34.90)
Diet high in sodium	Republic of Korea	1.99 (0.25 to 5.12)
Diet low in fruits	Republic of Korea	2.96 (2.07 to 3.93)
Diet low in vegetables	Republic of Korea	1.71 (1.03 to 2.66)
High body-mass index	Republic of Korea	3.78 (2.01 to 6.14)
High systolic blood pressure	Republic of Korea	11.55 (7.86 to 15.68)
Lead exposure	Republic of Korea	0.79 (-0.10 to 2.06)
Smoking	Republic of Korea	29.91 (23.92 to 36.27)
Diet high in sodium	Singapore	1.63 (0.12 to 4.41)
Diet low in fruits	Singapore	1.87 (1.21 to 2.56)
Diet low in vegetables	Singapore	1.64 (0.96 to 2.51)
High body-mass index	Singapore	4.61 (2.43 to 7.86)
High systolic blood pressure	Singapore	11.01 (7.35 to 15.23)
Lead exposure	Singapore	0.96 (-0.12 to 2.45)
Smoking	Singapore	19.39 (15.48 to 23.59)
Diet high in sodium	South Asia	0.71 (0.02 to 2.62)
Diet low in fruits	South Asia	5.53 (3.84 to 7.22)
Diet low in vegetables	South Asia	4.23 (2.88 to 5.86)
High body-mass index	South Asia	3.24 (1.84 to 5.26)

High systolic blood pressure	South Asia	16.27 (12.19 to 20.76)
Lead exposure	South Asia	1.42 (-0.19 to 3.58)
Smoking	South Asia	25.18 (19.66 to 31.60)
Diet high in sodium	Southeast Asia	1.24 (0.13 to 3.35)
Diet low in fruits	Southeast Asia	3.61 (2.48 to 4.88)
Diet low in vegetables	Southeast Asia	4.51 (3.03 to 6.30)
High body-mass index	Southeast Asia	3.85 (2.13 to 6.13)
High systolic blood pressure	Southeast Asia	19.09 (14.31 to 24.02)
Lead exposure	Southeast Asia	0.59 (-0.08 to 1.53)
Smoking	Southeast Asia	29.26 (24.25 to 34.17)

AA, aortic aneurysm; ASDR, age-standardized death rates.

Fig 8B. Percentage contribution of risk factors to AA-ASDR in Asia grouped by sex in 2021

Risk factor	Sex	Location	Percent contribution to ASDR (%)
Diet high in sodium	Male	Central Asia	0.95 (0.06 to 2.90)
Diet high in sodium	Female	Central Asia	0.52 (0.00 to 2.04)
Diet low in fruits	Male	Central Asia	3.59 (2.51 to 4.79)
Diet low in fruits	Female	Central Asia	3.86 (2.66 to 5.14)
Diet low in vegetables	Male	Central Asia	0.93 (0.53 to 1.49)
Diet low in vegetables	Female	Central Asia	0.78 (0.42 to 1.32)
High body-mass index	Male	Central Asia	8.23 (4.52 to 14.15)
High body-mass index	Female	Central Asia	12.18 (6.64 to 20.84)
High systolic blood pressure	Male	Central Asia	20.07 (15.25 to 25.33)
High systolic blood pressure	Female	Central Asia	21.65 (16.17 to 27.51)
Lead exposure	Male	Central Asia	0.55 (-0.07 to 1.45)
Lead exposure	Female	Central Asia	0.42 (-0.06 to 1.15)
Smoking	Male	Central Asia	50.54 (44.07 to 56.73)
Smoking	Female	Central Asia	4.67 (3.65 to 5.85)
Diet high in sodium	Male	East Asia	2.19 (0.41 to 5.07)
Diet high in sodium	Female	East Asia	1.61 (0.27 to 4.08)
Diet low in fruits	Male	East Asia	3.20 (2.16 to 4.35)
Diet low in fruits	Female	East Asia	3.10 (2.07 to 4.27)
Diet low in vegetables	Male	East Asia	0.55 (0.27 to 0.96)
Diet low in vegetables	Female	East Asia	0.53 (0.26 to 0.91)
High body-mass index	Male	East Asia	5.27 (2.90 to 9.12)
High body-mass index	Female	East Asia	6.52 (3.55 to 10.97)
High systolic blood pressure	Male	East Asia	15.17 (10.81 to 20.05)
High systolic blood pressure	Female	East Asia	16.88 (11.49 to 22.81)
Lead exposure	Male	East Asia	1.05 (-0.14 to 2.66)
Lead exposure	Female	East Asia	0.78 (-0.10 to 1.97)
Smoking	Male	East Asia	55.28 (48.99 to 60.71)
Smoking	Female	East Asia	11.04 (8.28 to 13.96)
Diet high in sodium	Male	Japan	1.19 (0.08 to 3.32)
Diet high in sodium	Female	Japan	0.86 (0.02 to 2.81)
Diet low in fruits	Male	Japan	4.22 (2.92 to 5.69)
Diet low in fruits	Female	Japan	3.86 (2.61 to 5.22)
Diet low in vegetables	Male	Japan	1.24 (0.63 to 2.03)
Diet low in vegetables	Female	Japan	1.34 (0.69 to 2.25)
High body-mass index	Male	Japan	4.55 (2.46 to 7.37)
High body-mass index	Female	Japan	4.45 (2.30 to 7.45)
High systolic blood pressure	Male	Japan	17.86 (13.28 to 23.53)
High systolic blood pressure	Female	Japan	17.19 (11.77 to 23.51)
Lead exposure	Male	Japan	0.35 (-0.04 to 0.95)

Lead exposure	Female	Japan	0.33 (-0.05 to 0.86)
Smoking	Male	Japan	42.02 (35.73 to 48.55)
Smoking	Female	Japan	11.64 (8.77 to 15.14)
Diet high in sodium	Male	Republic of Korea	2.16 (0.28 to 5.47)
Diet high in sodium	Female	Republic of Korea	1.82 (0.19 to 4.86)
Diet low in fruits	Male	Republic of Korea	2.85 (1.94 to 3.84)
Diet low in fruits	Female	Republic of Korea	3.02 (2.09 to 4.20)
Diet low in vegetables	Male	Republic of Korea	1.62 (0.92 to 2.58)
Diet low in vegetables	Female	Republic of Korea	1.82 (1.03 to 2.88)
High body-mass index	Male	Republic of Korea	3.29 (1.78 to 5.55)
High body-mass index	Female	Republic of Korea	4.16 (2.19 to 7.05)
High systolic blood pressure	Male	Republic of Korea	10.55 (6.91 to 14.78)
High systolic blood pressure	Female	Republic of Korea	12.35 (7.79 to 17.73)
Lead exposure	Male	Republic of Korea	0.90 (-0.11 to 2.36)
Lead exposure	Female	Republic of Korea	0.70 (-0.09 to 1.84)
Smoking	Male	Republic of Korea	44.52 (36.54 to 52.34)
Smoking	Female	Republic of Korea	12.61 (8.59 to 18.38)
Diet high in sodium	Male	Singapore	1.81 (0.13 to 4.97)
Diet high in sodium	Female	Singapore	1.34 (0.07 to 3.96)
Diet low in fruits	Male	Singapore	1.83 (1.20 to 2.55)
Diet low in fruits	Female	Singapore	1.89 (1.20 to 2.69)
Diet low in vegetables	Male	Singapore	1.69 (0.98 to 2.57)
Diet low in vegetables	Female	Singapore	1.56 (0.86 to 2.48)
High body-mass index	Male	Singapore	4.69 (2.51 to 8.20)
High body-mass index	Female	Singapore	4.41 (2.24 to 7.63)
High systolic blood pressure	Male	Singapore	10.76 (7.11 to 15.62)
High systolic blood pressure	Female	Singapore	11.19 (6.49 to 16.48)
Lead exposure	Male	Singapore	1.03 (-0.13 to 2.63)
Lead exposure	Female	Singapore	0.88 (-0.12 to 2.26)
Smoking	Male	Singapore	25.85 (20.80 to 31.19)
Smoking	Female	Singapore	7.27 (4.84 to 10.49)
Diet high in sodium	Male	South Asia	0.83 (0.03 to 2.94)
Diet high in sodium	Female	South Asia	0.53 (0.00 to 2.14)
Diet low in fruits	Male	South Asia	5.41 (3.76 to 7.06)
Diet low in fruits	Female	South Asia	5.68 (3.94 to 7.42)
Diet low in vegetables	Male	South Asia	4.09 (2.79 to 5.67)
Diet low in vegetables	Female	South Asia	4.43 (3.02 to 6.14)
High body-mass index	Male	South Asia	2.84 (1.63 to 4.57)
High body-mass index	Female	South Asia	3.85 (2.12 to 6.44)
High systolic blood pressure	Male	South Asia	15.57 (11.63 to 19.92)
High systolic blood pressure	Female	South Asia	17.34 (13.04 to 21.92)
Lead exposure	Male	South Asia	1.54 (-0.21 to 3.87)

Lead exposure	Female	South Asia	1.24 (-0.18 to 3.11)
Smoking	Male	South Asia	35.88 (30.17 to 42.21)
Smoking	Female	South Asia	8.47 (6.47 to 11.09)
Diet high in sodium	Male	Southeast Asia	1.45 (0.17 to 3.82)
Diet high in sodium	Female	Southeast Asia	0.91 (0.05 to 2.74)
Diet low in fruits	Male	Southeast Asia	3.43 (2.34 to 4.61)
Diet low in fruits	Female	Southeast Asia	3.83 (2.58 to 5.27)
Diet low in vegetables	Male	Southeast Asia	4.39 (2.95 to 6.11)
Diet low in vegetables	Female	Southeast Asia	4.67 (3.13 to 6.61)
High body-mass index	Male	Southeast Asia	3.19 (1.77 to 5.18)
High body-mass index	Female	Southeast Asia	4.83 (2.61 to 7.84)
High systolic blood pressure	Male	Southeast Asia	18.25 (13.47 to 23.34)
High systolic blood pressure	Female	Southeast Asia	20.18 (15.14 to 25.80)
Lead exposure	Male	Southeast Asia	0.68 (-0.09 to 1.75)
Lead exposure	Female	Southeast Asia	0.44 (-0.06 to 1.15)
Smoking	Male	Southeast Asia	42.36 (36.33 to 48.85)
Smoking	Female	Southeast Asia	7.42 (5.74 to 9.44)

AA, aortic aneurysm; ASDR, age-standardized death rates.

Fig 8C. AA-ASDR attributable to risk factors in Asia grouped by sex from 1990 to 2021

Year	Risk factors	Sex	Location	ASDR per 100,000
2021	Diet high in sodium	Male	Central Asia	0.03 (0.00 to 0.09)
2021	Diet high in sodium	Female	Central Asia	0.01 (0.00 to 0.02)
2021	Diet high in sodium	Both	Central Asia	0.02 (0.00 to 0.05)
1990	Diet high in sodium	Male	Central Asia	0.02 (0.00 to 0.05)
1990	Diet high in sodium	Female	Central Asia	0.00 (0.00 to 0.02)
1990	Diet high in sodium	Both	Central Asia	0.01 (0.00 to 0.03)
1990	Diet low in fruits	Male	Central Asia	0.07 (0.05 to 0.10)
1990	Diet low in fruits	Female	Central Asia	0.03 (0.02 to 0.05)
1990	Diet low in fruits	Both	Central Asia	0.05 (0.03 to 0.07)
2021	Diet low in fruits	Male	Central Asia	0.11 (0.08 to 0.15)
2021	Diet low in fruits	Female	Central Asia	0.05 (0.03 to 0.07)
2021	Diet low in fruits	Both	Central Asia	0.07 (0.05 to 0.10)
1990	Diet low in vegetables	Male	Central Asia	0.03 (0.02 to 0.05)
1990	Diet low in vegetables	Female	Central Asia	0.01 (0.01 to 0.02)
1990	Diet low in vegetables	Both	Central Asia	0.02 (0.01 to 0.03)
2021	Diet low in vegetables	Male	Central Asia	0.03 (0.02 to 0.05)
2021	Diet low in vegetables	Female	Central Asia	0.01 (0.01 to 0.02)
2021	Diet low in vegetables	Both	Central Asia	0.02 (0.01 to 0.03)
1990	High body-mass index	Male	Central Asia	0.10 (0.05 to 0.17)
1990	High body-mass index	Female	Central Asia	0.06 (0.03 to 0.11)
1990	High body-mass index	Both	Central Asia	0.08 (0.04 to 0.14)
2021	High body-mass index	Male	Central Asia	0.25 (0.14 to 0.43)
2021	High body-mass index	Female	Central Asia	0.15 (0.08 to 0.25)
2021	High body-mass index	Both	Central Asia	0.20 (0.11 to 0.34)
1990	High systolic blood pressure	Male	Central Asia	0.28 (0.21 to 0.38)
1990	High systolic blood pressure	Female	Central Asia	0.13 (0.09 to 0.17)
1990	High systolic blood pressure	Both	Central Asia	0.19 (0.14 to 0.25)
2021	High systolic blood pressure	Male	Central Asia	0.62 (0.46 to 0.80)
2021	High systolic blood pressure	Female	Central Asia	0.26 (0.19 to 0.35)
2021	High systolic blood pressure	Both	Central Asia	0.41 (0.31 to 0.54)
1990	Lead exposure	Male	Central Asia	0.01 (0.00 to 0.02)
1990	Lead exposure	Female	Central Asia	0.00 (0.00 to 0.01)
1990	Lead exposure	Both	Central Asia	0.00 (0.00 to 0.01)
2021	Lead exposure	Male	Central Asia	0.02 (0.00 to 0.04)
2021	Lead exposure	Female	Central Asia	0.01 (0.00 to 0.01)
2021	Lead exposure	Both	Central Asia	0.01 (0.00 to 0.03)
1990	Smoking	Male	Central Asia	0.77 (0.65 to 0.95)
1990	Smoking	Female	Central Asia	0.04 (0.03 to 0.05)
1990	Smoking	Both	Central Asia	0.33 (0.28 to 0.40)

2021 Smoking	Male	Central Asia	1.56 (1.30 to 1.83)
2021 Smoking	Female	Central Asia	0.06 (0.04 to 0.08)
2021 Smoking	Both	Central Asia	0.67 (0.56 to 0.79)
1990 Diet high in sodium	Male	East Asia	0.01 (0.00 to 0.03)
1990 Diet high in sodium	Female	East Asia	0.01 (0.00 to 0.01)
1990 Diet high in sodium	Both	East Asia	0.01 (0.00 to 0.02)
2021 Diet high in sodium	Male	East Asia	0.02 (0.00 to 0.04)
2021 Diet high in sodium	Female	East Asia	0.00 (0.00 to 0.01)
2021 Diet high in sodium	Both	East Asia	0.01 (0.00 to 0.03)
1990 Diet low in fruits	Male	East Asia	0.02 (0.01 to 0.03)
1990 Diet low in fruits	Female	East Asia	0.01 (0.01 to 0.02)
1990 Diet low in fruits	Both	East Asia	0.01 (0.01 to 0.02)
2021 Diet low in fruits	Male	East Asia	0.03 (0.01 to 0.04)
2021 Diet low in fruits	Female	East Asia	0.01 (0.00 to 0.01)
2021 Diet low in fruits	Both	East Asia	0.02 (0.01 to 0.02)
1990 Diet low in vegetables	Male	East Asia	0.01 (0.01 to 0.02)
1990 Diet low in vegetables	Female	East Asia	0.01 (0.00 to 0.01)
1990 Diet low in vegetables	Both	East Asia	0.01 (0.01 to 0.01)
2021 Diet low in vegetables	Male	East Asia	0.00 (0.00 to 0.01)
2021 Diet low in vegetables	Female	East Asia	0.00 (0.00 to 0.00)
2021 Diet low in vegetables	Both	East Asia	0.00 (0.00 to 0.00)
1990 High body-mass index	Male	East Asia	0.01 (0.01 to 0.02)
1990 High body-mass index	Female	East Asia	0.01 (0.00 to 0.02)
1990 High body-mass index	Both	East Asia	0.01 (0.00 to 0.02)
2021 High body-mass index	Male	East Asia	0.04 (0.02 to 0.07)
2021 High body-mass index	Female	East Asia	0.02 (0.01 to 0.03)
2021 High body-mass index	Both	East Asia	0.03 (0.01 to 0.05)
1990 High systolic blood pressure	Male	East Asia	0.05 (0.03 to 0.08)
1990 High systolic blood pressure	Female	East Asia	0.04 (0.02 to 0.06)
1990 High systolic blood pressure	Both	East Asia	0.05 (0.03 to 0.06)
2021 High systolic blood pressure	Male	East Asia	0.12 (0.08 to 0.18)
2021 High systolic blood pressure	Female	East Asia	0.04 (0.03 to 0.06)
2021 High systolic blood pressure	Both	East Asia	0.08 (0.05 to 0.11)
1990 Lead exposure	Male	East Asia	0.01 (0.00 to 0.01)
1990 Lead exposure	Female	East Asia	0.00 (0.00 to 0.01)
1990 Lead exposure	Both	East Asia	0.00 (0.00 to 0.01)
2021 Lead exposure	Male	East Asia	0.01 (0.00 to 0.02)
2021 Lead exposure	Female	East Asia	0.00 (0.00 to 0.01)
2021 Lead exposure	Both	East Asia	0.00 (0.00 to 0.01)
1990 Smoking	Male	East Asia	0.26 (0.18 to 0.37)
1990 Smoking	Female	East Asia	0.04 (0.03 to 0.06)
1990 Smoking	Both	East Asia	0.15 (0.11 to 0.20)

2021 Smoking	Male	East Asia	0.43 (0.31 to 0.56)
2021 Smoking	Female	East Asia	0.03 (0.02 to 0.04)
2021 Smoking	Both	East Asia	0.22 (0.16 to 0.28)
1990 Diet high in sodium	Male	Japan	0.06 (0.01 to 0.15)
1990 Diet high in sodium	Female	Japan	0.02 (0.00 to 0.05)
1990 Diet high in sodium	Both	Japan	0.04 (0.01 to 0.09)
2021 Diet high in sodium	Male	Japan	0.08 (0.01 to 0.22)
2021 Diet high in sodium	Female	Japan	0.03 (0.00 to 0.11)
2021 Diet high in sodium	Both	Japan	0.05 (0.00 to 0.16)
1990 Diet low in fruits	Male	Japan	0.22 (0.16 to 0.29)
1990 Diet low in fruits	Female	Japan	0.09 (0.06 to 0.12)
1990 Diet low in fruits	Both	Japan	0.14 (0.10 to 0.19)
2021 Diet low in fruits	Male	Japan	0.28 (0.19 to 0.38)
2021 Diet low in fruits	Female	Japan	0.14 (0.09 to 0.20)
2021 Diet low in fruits	Both	Japan	0.21 (0.14 to 0.28)
1990 Diet low in vegetables	Male	Japan	0.11 (0.07 to 0.16)
1990 Diet low in vegetables	Female	Japan	0.04 (0.03 to 0.07)
1990 Diet low in vegetables	Both	Japan	0.07 (0.04 to 0.10)
2021 Diet low in vegetables	Male	Japan	0.08 (0.04 to 0.13)
2021 Diet low in vegetables	Female	Japan	0.05 (0.03 to 0.09)
2021 Diet low in vegetables	Both	Japan	0.07 (0.04 to 0.11)
1990 High body-mass index	Male	Japan	0.16 (0.09 to 0.27)
1990 High body-mass index	Female	Japan	0.08 (0.04 to 0.14)
1990 High body-mass index	Both	Japan	0.12 (0.06 to 0.19)
2021 High body-mass index	Male	Japan	0.30 (0.16 to 0.49)
2021 High body-mass index	Female	Japan	0.17 (0.08 to 0.28)
2021 High body-mass index	Both	Japan	0.23 (0.12 to 0.37)
1990 High systolic blood pressure	Male	Japan	1.01 (0.77 to 1.26)
1990 High systolic blood pressure	Female	Japan	0.41 (0.31 to 0.51)
1990 High systolic blood pressure	Both	Japan	0.66 (0.50 to 0.82)
2021 High systolic blood pressure	Male	Japan	1.18 (0.89 to 1.56)
2021 High systolic blood pressure	Female	Japan	0.64 (0.41 to 0.92)
2021 High systolic blood pressure	Both	Japan	0.89 (0.64 to 1.18)
1990 Lead exposure	Male	Japan	0.01 (0.00 to 0.04)
1990 Lead exposure	Female	Japan	0.01 (0.00 to 0.01)
1990 Lead exposure	Both	Japan	0.01 (0.00 to 0.02)
2021 Lead exposure	Male	Japan	0.02 (0.00 to 0.06)
2021 Lead exposure	Female	Japan	0.01 (0.00 to 0.03)
2021 Lead exposure	Both	Japan	0.02 (0.00 to 0.05)
1990 Smoking	Male	Japan	2.47 (2.12 to 2.81)
1990 Smoking	Female	Japan	0.28 (0.22 to 0.36)
1990 Smoking	Both	Japan	1.17 (1.01 to 1.34)

2021 Smoking	Male	Japan	2.78 (2.34 to 3.26)
2021 Smoking	Female	Japan	0.43 (0.31 to 0.59)
2021 Smoking	Both	Japan	1.50 (1.25 to 1.78)
2021 Diet high in sodium	Male	Republic of Korea	0.05 (0.01 to 0.15)
2021 Diet high in sodium	Female	Republic of Korea	0.03 (0.00 to 0.07)
2021 Diet high in sodium	Both	Republic of Korea	0.04 (0.00 to 0.10)
1990 Diet high in sodium	Male	Republic of Korea	0.04 (0.00 to 0.12)
1990 Diet high in sodium	Female	Republic of Korea	0.02 (0.00 to 0.07)
1990 Diet high in sodium	Both	Republic of Korea	0.03 (0.00 to 0.08)
1990 Diet low in fruits	Male	Republic of Korea	0.09 (0.05 to 0.16)
1990 Diet low in fruits	Female	Republic of Korea	0.06 (0.04 to 0.11)
1990 Diet low in fruits	Both	Republic of Korea	0.08 (0.04 to 0.12)
2021 Diet low in fruits	Male	Republic of Korea	0.07 (0.05 to 0.10)
2021 Diet low in fruits	Female	Republic of Korea	0.05 (0.03 to 0.07)
2021 Diet low in fruits	Both	Republic of Korea	0.06 (0.04 to 0.08)
1990 Diet low in vegetables	Male	Republic of Korea	0.03 (0.01 to 0.06)
1990 Diet low in vegetables	Female	Republic of Korea	0.02 (0.01 to 0.04)
1990 Diet low in vegetables	Both	Republic of Korea	0.02 (0.01 to 0.04)
2021 Diet low in vegetables	Male	Republic of Korea	0.04 (0.02 to 0.06)
2021 Diet low in vegetables	Female	Republic of Korea	0.03 (0.01 to 0.05)
2021 Diet low in vegetables	Both	Republic of Korea	0.03 (0.02 to 0.05)
1990 High body-mass index	Male	Republic of Korea	0.07 (0.02 to 0.14)
1990 High body-mass index	Female	Republic of Korea	0.05 (0.02 to 0.09)
1990 High body-mass index	Both	Republic of Korea	0.06 (0.02 to 0.11)
2021 High body-mass index	Male	Republic of Korea	0.08 (0.04 to 0.15)
2021 High body-mass index	Female	Republic of Korea	0.06 (0.03 to 0.11)
2021 High body-mass index	Both	Republic of Korea	0.07 (0.03 to 0.13)
1990 High systolic blood pressure	Male	Republic of Korea	0.35 (0.18 to 0.59)
1990 High systolic blood pressure	Female	Republic of Korea	0.26 (0.15 to 0.41)
1990 High systolic blood pressure	Both	Republic of Korea	0.30 (0.17 to 0.45)
2021 High systolic blood pressure	Male	Republic of Korea	0.26 (0.15 to 0.39)
2021 High systolic blood pressure	Female	Republic of Korea	0.18 (0.10 to 0.29)
2021 High systolic blood pressure	Both	Republic of Korea	0.22 (0.14 to 0.31)
1990 Lead exposure	Male	Republic of Korea	0.02 (0.00 to 0.06)
1990 Lead exposure	Female	Republic of Korea	0.01 (0.00 to 0.03)
1990 Lead exposure	Both	Republic of Korea	0.01 (0.00 to 0.04)
2021 Lead exposure	Male	Republic of Korea	0.02 (0.00 to 0.06)
2021 Lead exposure	Female	Republic of Korea	0.01 (0.00 to 0.03)
2021 Lead exposure	Both	Republic of Korea	0.02 (0.00 to 0.04)
1990 Smoking	Male	Republic of Korea	1.31 (0.75 to 2.00)
1990 Smoking	Female	Republic of Korea	0.28 (0.16 to 0.45)
1990 Smoking	Both	Republic of Korea	0.65 (0.39 to 0.97)

2021 Smoking	Male	Republic of Korea	1.10 (0.81 to 1.43)
2021 Smoking	Female	Republic of Korea	0.19 (0.11 to 0.30)
2021 Smoking	Both	Republic of Korea	0.57 (0.43 to 0.72)
2021 Diet high in sodium	Male	Singapore	0.05 (0.00 to 0.14)
2021 Diet high in sodium	Female	Singapore	0.02 (0.00 to 0.06)
2021 Diet high in sodium	Both	Singapore	0.04 (0.00 to 0.10)
1990 Diet high in sodium	Male	Singapore	0.08 (0.01 to 0.20)
1990 Diet high in sodium	Female	Singapore	0.02 (0.00 to 0.06)
1990 Diet high in sodium	Both	Singapore	0.05 (0.01 to 0.12)
1990 Diet low in fruits	Male	Singapore	0.14 (0.10 to 0.19)
1990 Diet low in fruits	Female	Singapore	0.05 (0.03 to 0.08)
1990 Diet low in fruits	Both	Singapore	0.09 (0.06 to 0.12)
2021 Diet low in fruits	Male	Singapore	0.05 (0.03 to 0.07)
2021 Diet low in fruits	Female	Singapore	0.03 (0.02 to 0.04)
2021 Diet low in fruits	Both	Singapore	0.04 (0.03 to 0.06)
1990 Diet low in vegetables	Male	Singapore	0.12 (0.08 to 0.18)
1990 Diet low in vegetables	Female	Singapore	0.04 (0.03 to 0.07)
1990 Diet low in vegetables	Both	Singapore	0.08 (0.05 to 0.12)
2021 Diet low in vegetables	Male	Singapore	0.05 (0.03 to 0.07)
2021 Diet low in vegetables	Female	Singapore	0.02 (0.01 to 0.04)
2021 Diet low in vegetables	Both	Singapore	0.04 (0.02 to 0.05)
1990 High body-mass index	Male	Singapore	0.14 (0.07 to 0.24)
1990 High body-mass index	Female	Singapore	0.04 (0.02 to 0.07)
1990 High body-mass index	Both	Singapore	0.09 (0.05 to 0.15)
2021 High body-mass index	Male	Singapore	0.13 (0.07 to 0.23)
2021 High body-mass index	Female	Singapore	0.07 (0.03 to 0.12)
2021 High body-mass index	Both	Singapore	0.10 (0.05 to 0.17)
1990 High systolic blood pressure	Male	Singapore	0.71 (0.52 to 0.92)
1990 High systolic blood pressure	Female	Singapore	0.28 (0.18 to 0.39)
1990 High systolic blood pressure	Both	Singapore	0.47 (0.34 to 0.61)
2021 High systolic blood pressure	Male	Singapore	0.31 (0.19 to 0.45)
2021 High systolic blood pressure	Female	Singapore	0.17 (0.10 to 0.26)
2021 High systolic blood pressure	Both	Singapore	0.24 (0.15 to 0.33)
1990 Lead exposure	Male	Singapore	0.04 (-0.01 to 0.11)
1990 Lead exposure	Female	Singapore	0.01 (0.00 to 0.03)
1990 Lead exposure	Both	Singapore	0.03 (0.00 to 0.07)
2021 Lead exposure	Male	Singapore	0.03 (0.00 to 0.08)
2021 Lead exposure	Female	Singapore	0.01 (0.00 to 0.04)
2021 Lead exposure	Both	Singapore	0.02 (0.00 to 0.05)
1990 Smoking	Male	Singapore	1.58 (1.30 to 1.88)
1990 Smoking	Female	Singapore	0.17 (0.11 to 0.25)
1990 Smoking	Both	Singapore	0.81 (0.67 to 0.97)

2021 Smoking	Male	Singapore	0.74 (0.59 to 0.92)
2021 Smoking	Female	Singapore	0.11 (0.07 to 0.16)
2021 Smoking	Both	Singapore	0.42 (0.33 to 0.52)
2021 Diet high in sodium	Male	South Asia	0.01 (0.00 to 0.05)
2021 Diet high in sodium	Female	South Asia	0.00 (0.00 to 0.02)
2021 Diet high in sodium	Both	South Asia	0.01 (0.00 to 0.03)
1990 Diet high in sodium	Male	South Asia	0.01 (0.00 to 0.02)
1990 Diet high in sodium	Female	South Asia	0.00 (0.00 to 0.02)
1990 Diet high in sodium	Both	South Asia	0.00 (0.00 to 0.02)
1990 Diet low in fruits	Male	South Asia	0.04 (0.02 to 0.10)
1990 Diet low in fruits	Female	South Asia	0.04 (0.02 to 0.08)
1990 Diet low in fruits	Both	South Asia	0.04 (0.02 to 0.07)
2021 Diet low in fruits	Male	South Asia	0.08 (0.05 to 0.16)
2021 Diet low in fruits	Female	South Asia	0.05 (0.03 to 0.08)
2021 Diet low in fruits	Both	South Asia	0.07 (0.04 to 0.11)
1990 Diet low in vegetables	Male	South Asia	0.04 (0.02 to 0.09)
1990 Diet low in vegetables	Female	South Asia	0.03 (0.01 to 0.07)
1990 Diet low in vegetables	Both	South Asia	0.03 (0.02 to 0.06)
2021 Diet low in vegetables	Male	South Asia	0.06 (0.04 to 0.12)
2021 Diet low in vegetables	Female	South Asia	0.04 (0.02 to 0.07)
2021 Diet low in vegetables	Both	South Asia	0.05 (0.03 to 0.08)
1990 High body-mass index	Male	South Asia	0.01 (0.00 to 0.03)
1990 High body-mass index	Female	South Asia	0.01 (0.00 to 0.03)
1990 High body-mass index	Both	South Asia	0.01 (0.01 to 0.02)
2021 High body-mass index	Male	South Asia	0.04 (0.02 to 0.09)
2021 High body-mass index	Female	South Asia	0.03 (0.02 to 0.07)
2021 High body-mass index	Both	South Asia	0.04 (0.02 to 0.07)
1990 High systolic blood pressure	Male	South Asia	0.11 (0.05 to 0.25)
1990 High systolic blood pressure	Female	South Asia	0.11 (0.05 to 0.25)
1990 High systolic blood pressure	Both	South Asia	0.11 (0.06 to 0.18)
2021 High systolic blood pressure	Male	South Asia	0.24 (0.14 to 0.41)
2021 High systolic blood pressure	Female	South Asia	0.16 (0.09 to 0.24)
2021 High systolic blood pressure	Both	South Asia	0.20 (0.13 to 0.30)
1990 Lead exposure	Male	South Asia	0.01 (0.00 to 0.04)
1990 Lead exposure	Female	South Asia	0.01 (0.00 to 0.03)
1990 Lead exposure	Both	South Asia	0.01 (0.00 to 0.03)
2021 Lead exposure	Male	South Asia	0.02 (0.00 to 0.07)
2021 Lead exposure	Female	South Asia	0.01 (0.00 to 0.03)
2021 Lead exposure	Both	South Asia	0.02 (0.00 to 0.05)
1990 Smoking	Male	South Asia	0.36 (0.17 to 0.81)
1990 Smoking	Female	South Asia	0.07 (0.03 to 0.14)
1990 Smoking	Both	South Asia	0.22 (0.13 to 0.44)

2021 Smoking	Male	South Asia	0.57 (0.35 to 1.00)
2021 Smoking	Female	South Asia	0.08 (0.04 to 0.12)
2021 Smoking	Both	South Asia	0.31 (0.20 to 0.52)
2021 Diet high in sodium	Male	Southeast Asia	0.03 (0.00 to 0.08)
2021 Diet high in sodium	Female	Southeast Asia	0.01 (0.00 to 0.03)
2021 Diet high in sodium	Both	Southeast Asia	0.02 (0.00 to 0.05)
1990 Diet high in sodium	Male	Southeast Asia	0.03 (0.00 to 0.06)
1990 Diet high in sodium	Female	Southeast Asia	0.01 (0.00 to 0.03)
1990 Diet high in sodium	Both	Southeast Asia	0.02 (0.00 to 0.04)
1990 Diet low in fruits	Male	Southeast Asia	0.06 (0.04 to 0.09)
1990 Diet low in fruits	Female	Southeast Asia	0.04 (0.02 to 0.06)
1990 Diet low in fruits	Both	Southeast Asia	0.05 (0.03 to 0.07)
2021 Diet low in fruits	Male	Southeast Asia	0.07 (0.04 to 0.10)
2021 Diet low in fruits	Female	Southeast Asia	0.04 (0.02 to 0.05)
2021 Diet low in fruits	Both	Southeast Asia	0.05 (0.03 to 0.07)
1990 Diet low in vegetables	Male	Southeast Asia	0.07 (0.04 to 0.10)
1990 Diet low in vegetables	Female	Southeast Asia	0.04 (0.02 to 0.07)
1990 Diet low in vegetables	Both	Southeast Asia	0.05 (0.03 to 0.08)
2021 Diet low in vegetables	Male	Southeast Asia	0.09 (0.06 to 0.13)
2021 Diet low in vegetables	Female	Southeast Asia	0.04 (0.03 to 0.06)
2021 Diet low in vegetables	Both	Southeast Asia	0.06 (0.04 to 0.09)
1990 High body-mass index	Male	Southeast Asia	0.02 (0.01 to 0.04)
1990 High body-mass index	Female	Southeast Asia	0.02 (0.01 to 0.03)
1990 High body-mass index	Both	Southeast Asia	0.02 (0.01 to 0.04)
2021 High body-mass index	Male	Southeast Asia	0.06 (0.03 to 0.11)
2021 High body-mass index	Female	Southeast Asia	0.04 (0.02 to 0.07)
2021 High body-mass index	Both	Southeast Asia	0.05 (0.03 to 0.09)
1990 High systolic blood pressure	Male	Southeast Asia	0.21 (0.14 to 0.32)
1990 High systolic blood pressure	Female	Southeast Asia	0.14 (0.09 to 0.21)
1990 High systolic blood pressure	Both	Southeast Asia	0.17 (0.12 to 0.24)
2021 High systolic blood pressure	Male	Southeast Asia	0.37 (0.26 to 0.51)
2021 High systolic blood pressure	Female	Southeast Asia	0.19 (0.13 to 0.25)
2021 High systolic blood pressure	Both	Southeast Asia	0.26 (0.19 to 0.35)
1990 Lead exposure	Male	Southeast Asia	0.01 (0.00 to 0.02)
1990 Lead exposure	Female	Southeast Asia	0.00 (0.00 to 0.01)
1990 Lead exposure	Both	Southeast Asia	0.01 (0.00 to 0.02)
2021 Lead exposure	Male	Southeast Asia	0.01 (0.00 to 0.04)
2021 Lead exposure	Female	Southeast Asia	0.00 (0.00 to 0.01)
2021 Lead exposure	Both	Southeast Asia	0.01 (0.00 to 0.02)
1990 Smoking	Male	Southeast Asia	0.67 (0.50 to 0.99)
1990 Smoking	Female	Southeast Asia	0.09 (0.07 to 0.13)
1990 Smoking	Both	Southeast Asia	0.35 (0.26 to 0.49)

2021	Smoking	Male	Southeast Asia	0.85 (0.66 to 1.08)
2021	Smoking	Female	Southeast Asia	0.07 (0.05 to 0.09)
2021	Smoking	Both	Southeast Asia	0.41 (0.32 to 0.51)

AA, aortic aneurysm; ASDR, age-standardized death rates.
